# Electrochemical Studies of the Cycloaddition Activity of Bismuth(III) Acetylides Towards Organic Azides Under Copper(I)-Catalyzed Conditions

**DOI:** 10.3389/fchem.2022.830237

**Published:** 2022-02-25

**Authors:** Antonina L. Nazarova, Billal Zayat, Valery V. Fokin, Sri R. Narayan

**Affiliations:** ^1^ Department of Chemistry, Loker Hydrocarbon Research Institute, University of Southern California, Los Angeles, CA, United States; ^2^ Bridge Institute, USC Michelson Center for Convergent Bioscience, University of Southern California, Los Angeles, CA, United States

**Keywords:** cyclic voltammetry, mechanistic studies, bismuth(III) acetylides, reaction kinetic analysis, copper(I)catalysis, ordinary differential equations, NMR reaction profiling, copper-mediated 1,3 cycloaddition

## Abstract

Time-dependent monitoring of the reactive intermediates provides valuable information about the mechanism of a synthetic transformation. However, the process frequently involves intermediates with short lifetimes that significantly challenge the accessibility of the desired kinetic data. We report *in situ* cyclic voltammetry (CV) and nuclear magnetic resonance (NMR) spectroscopy studies of the cycloaddition reaction of organobismuth(III) compounds with organic azides under the copper(I)-catalyzed conditions. A series of bismuth(III) acetylides carrying diphenyl sulfone scaffolds have been synthesized to study the underlying electronic and steric effects of the tethered moieties capable of transannular oxygen O···Bi interactions and *para*-functionality of the parent phenylacetylene backbones. While belonging to the family of copper-catalyzed azide-alkyne cycloaddition reactions, the reaction yielding 5-bismuth(III)-triazolide is the sole example of a complex catalytic transformation that features activity of bismuth(III) acetylides towards organic azides under copper(I)-catalyzed conditions. Stepwise continuous monitoring of the copper(I)/copper(0) redox activity of the copper(I) catalyst by cyclic voltammetry provided novel insights into the complex catalytic cycle of the bismuth(III)-triazolide formation. From CV-derived kinetic data, reaction rate parameters of the bismuth(III) acetylides coordination to the copper(I) catalyst (K_A_) and equilibrium concentration of the copper species [cat]_eq._ are compared with the overall 5-bismuth(III)-triazolide formation rate constant k_obs_ obtained by ^1^H-NMR kinetic analysis.

## Graphical Abstract

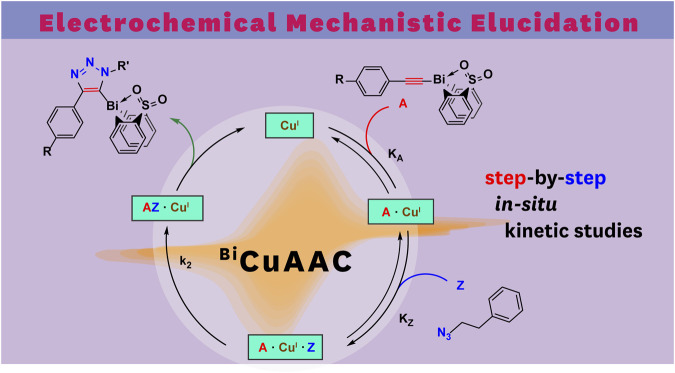

## Introduction

**FIGURE 1:**
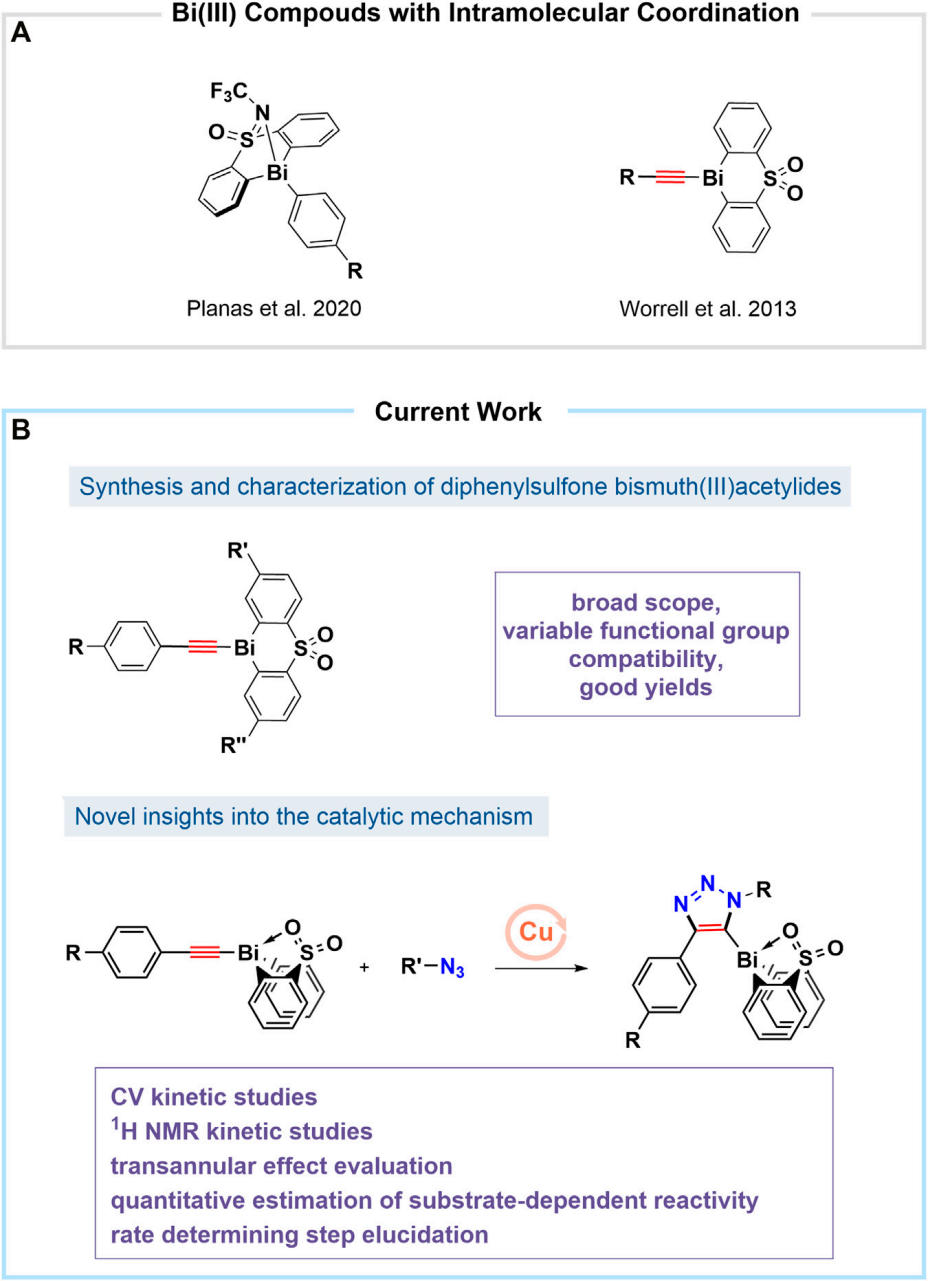
**(A)** Recent reports on organobismuth(III) compounds with tethered sulfonyl-linked bis-anionic ligands (Worrell et al., 2013a; Planas et al., 2020); **(B)** This work: 1) Synthesis of various bismuth(III) acetylides with diphenyl sulfone ligand derivatization and *para*-functionalization of the acetylenic unit 2) Mechanistic studies using cyclic voltammetry and NMR spectroscopy.

With an increased interest in the area of novel, non-toxic, and biocompatible nanomaterials, bismuth (Bi)-doped systems have become important in the area of near-infrared (NIR)-emitters and drug-delivery materials (Laguta and Razdobreev, 2018; Liu et al., 2018; Szostak et al., 2019; Orellana-Tavra et al., 2020). Bismuth has a negligibly low level of toxicity and carcinogenicity as compared to its highly-abundant neighbors in the periodic table (tin, lead, antimony, arsenic) (Spafford et al., 2008; Zhang et al., 2009; Mohan, 2010). However, biochemical and industrial applications involving bismuth-containing organic compounds remain limited (Luan et al., 2011; Ramler et al., 2019). Presently, commercially-available bismuth(III) compounds are typically inorganic salts used for instance as Lewis acid catalysts in organometallic reactions or those used for the preparation of nanoparticles (Suzuki et al., 2001; Rabin et al., 2006; Brown and Goforth, 2012; Bi et al., 2018). Recently the coordination with electron donors such as sulfur (S) and nitrogen (N) was reported to increase the thermal, air, and hydrolytic stability of bismuth in the oxidation states of (III) and (V), creating new opportunities for its applications (Raţ et al., 2013; Toma et al., 2016; Toma et al., 2017). Also, recently the bismuth(III)/(V) couple has been reported to show redox activity when participating in bimolecular interactions in solution (Inani et al., 1990). The investigation of the redox characteristics as well as the role of transition metals involved in catalytic processes through reaction kinetic profiling remains well established in organometallic chemistry (Jutand, 2008; Blackmond, 2015; Easter and Blum, 2019). Herein, we report a mechanistic study of the reactivity of *para*-phenyl substituted bismuth(III) acetylides in the copper(I)-catalyzed cycloaddition reactions with organic azides (Worrell et al., 2013a). Reaction progress experiments were performed using cyclic voltammetry (CV) and nuclear magnetic resonance (^1^H NMR) spectroscopy. To exploit bismuth’s coordination chemistry by modifying its electronic effects and geometry, we have focused our study on sulfonyl-merged bis-anionic aryl-tethered scaffolds (Ohkata et al., 1989; Suzuki et al., 2001; Nazarova and Fokin, 2018; Planas et al., 2020). By altering the electron-withdrawing nature of the sulfone moiety and the ability of the oxygen atom to intramolecularly coordinate to the bismuth center, we studied a variety of sulfone-type ligands that influence the bismuth(III) acetylides activity under copper(I)-catalyzed conditions (Figure 1).

## Materials and Methods

### Cyclic Voltammetry

All electrochemical measurements were performed inside a glovebox in a nitrogen atmosphere. Anhydrous DMSO was used as the solvent. A 3-electrode glass cell consisting of a glassy carbon working electrode (BASi, MF-2012), copper foil counter and reference electrodes, and copper(I) trifluoromethanesulfonate was used for the measurements. Electrochemical data was collected on a multi-channel potentiostat (AMETEK Scientific Instruments, VersaSTAT 4). Voltammograms were recorded between −0.3 and 0.8 V vs Cu^+^/Cu at a scan rate of 100 mV s^−1^.

#### Cyclic Voltammetry Kinetic Studies: Bismuth(III)-Acetylide–Copper(I) Catalyst π-Complex Formation

Stock solutions of the corresponding bismuth(III) acetylides (75.0 mM), (2-azidoethyl)benzene (135 mM), and copper(I) trifluoromethanesulfonate toluene complex (16.24 mM) were prepared in anhydrous DMSO. 0.5 ml of the copper(I) trifluoromethanesulfonate toluene complex stock solution and 6.0 ml of DMSO were transferred into the 3-electrode cell and stirred (final concentration of the copper(I) trifluoro-methane-sulfonate toluene complex was 1.25 mM). Three CV scan cycles were carried out to ensure the reproducibility of the current response and the stability of the copper(I) trifluoromethanesulfonate complex. No additional supporting electrolyte was used.

The CV scans for kinetic studies were performed in the same 3-electrode cell with a glassy carbon working electrode, and copper foils as counter and reference electrodes. Bismuth(III) acetylide stock solution in anhydrous DMSO (0.5 ml) was introduced to the electrochemical cell (final concentrations were 1.16 mM of the copper(I) trifluoromethanesulfonate toluene complex and 5.35 mM of the bismuth(III) acetylide) and stirred for half a minute prior to the start of the CV scans.

#### Cyclic Voltammetry Kinetic Studies: Azide Insertion/Reductive Elimination Step

A solution of the (2-azidoethyl)benzene in anhydrous DMSO (0.5 ml) was introduced to an electrochemical cell after the acetylide and copper catalyst mixture attained equilibrium (General Procedure A in the Supporting Information, final concentrations were 1.08 mM of the copper(I) trifluoromethanesulfonate toluene complex, 5.0 mM of the bismuth(III) acetylide, 9.0 mM of the azide). The mixture was stirred for half a minute before the start of the electrochemistry data acquisition. Continuous electrochemical data collection between −0.3 and 0.8 V vs Cu^+^/Cu at a scan rate of 100 mV s^−1^ was performed until no further changes in the redox peaks were observed. To study the electrochemical activity on the bismuth center of the bismuth(III) acetylides, CV responses were collected between −0.3 and 1.7 V vs Cu^+^/Cu at a scan rate of 100 mV s^−1^.

### ^1^H NMR and IR Spectroscopy and X-Ray Analysis

NMR spectra were recorded on Varian Mercury 400, Varian VNMRS-500, or Varian VNMRS-600 spectrometer. Chemical shifts were referenced to residual solvent signals (Chloroform-*d*: δ(^1^H) = 7.26 ppm, δ(^13^C) = 77.16 ppm, DMSO-d6: δ(^1^H) = 2.5 ppm, δ(^13^C) = 39.51) as an internal reference. ^19^F NMR spectra were externally referenced to 80% CFCl_3_ in chloroform-*d*. The following abbreviations were used to describe NMR signal multiplicities: s = singlet, d = doublet, t = triplet, q = quartet, quin = quintet, m = multiplet, b = broad. Infrared spectra were recorded in the range 4,000–400 cm^−1^ on a Bruker Alpha spectrometer using a diamond ATR unit. IR intensities are described as vw (very weak), w (weak), m (medium), s (strong), vs (very strong). X-ray crystallographic analysis was performed at UCSD on a Bruker Apex II Ultra2 CCD diffractometer equipped with Mo Kα radiation. Yields refer to chromatographically and spectroscopically (^1^H NMR) pure materials unless otherwise is stated.

### ^1^H NMR Kinetic Studies: 5-Bismuth(III) Triazolide[X] Formation

Stock solutions for the corresponding bismuth(III) acetylides (0.100 M), (2-azidoethyl)benzene (0.688 M), 1,4-dimethoxy-benzene (internal standard) (54 mM) and copper(I) trifluoromethanesulfonate toluene complex (20 mM) were prepared in DMSO-d6. Of these stock solutions: 200 μl of the alkyne, 50 μl of the azide, and 50 μl of the reference stock solutions were transferred to an NMR tube and diluted with 450 μl of DMSO-d6 capped with a gas-tight rubber NMR septum and agitated. Lastly, 50 μl of the copper(I) trifluoro-methane-sulfonate toluene complex catalyst solution was added to the NMR tube and immediately agitated. The total volume of the reaction mixture in the NMR tube for one experiment was 800 μl.

Kinetic NMR measurements were recorded on a Varian VNMRS-600 spectrometer. The sample spinning rate was 20 Hz. ^1^H-NMR data were collected with the following acquisition parameters: 1 transient (scan), 5 s relaxation delay time, acquisition time of 5.824 s. Between the spectra was a 1 s pre-acquisition delay. Data was collected at 60.0°C. The temperature was calibrated against a temperature standard (ethylene glycol). Each experiment was performed twice. All reagents were dried before use. All manipulations were performed in the dry nitrogen atmosphere of a glovebox. Reaction conversions were integrated relative to an internal reference standard.

## Results and Discussion

### Synthesis and Characterization of Diphenyl Sulfone Bismuth(III) Acetylides

**FIGURE 2:**
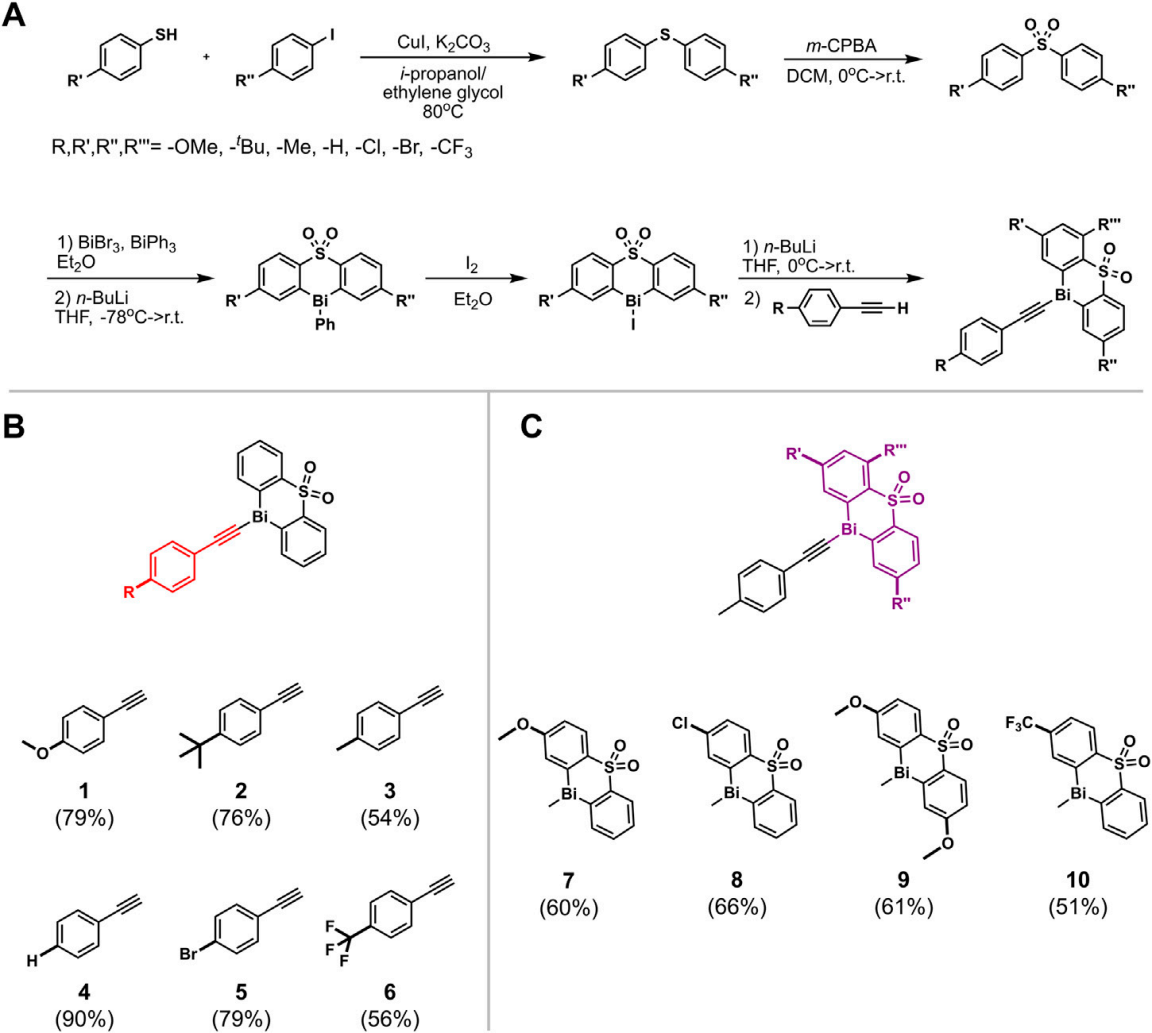
**(A)** Synthetic route for the synthesis of diphenyl sulfone bismuth(III) acetylides (a) CuI, K_2_CO_3_, *i*-propanol/ethylene glycol, 80°C; (b) *m*-CPBA, DCM, MgSO_4_ 10 eq., 0°C –> r.t.; (c) 1) BiBr_3_, BiPh_3_, Et_2_O, 2) *n*-BuLi, THF, −78°C –> r. t.; (d) I_2_, Et_2_O, r. t.; (e) 1) *n*-BuLi, THF, −78°C –> r. t. 2) *para*-substituted phenylacetylene; **(B)** diphenyl sulfone bismuth(III) acetylides; **(C)** 1-ethynyl-4-methylbenzene bismuth(III) acetylides with functionalized diphenyl sulfone ligands.

The rational design of the bismuth(III) acetylides was a crucial factor for gaining insights into the catalytic cycle. Two types of derivatization strategies were applied in the synthesis of the organobismuth acetylides: 1) diphenyl sulfone ligand derivatization with different functional groups; and 2) *para*-position phenylacetylene substitution with electron-deficient, neutral, and electron-rich functional groups (Suzuki et al., 2001). To study factors influencing the electronic environment of the bismuth(III) center, acetylides **A[1]** through **A[6]** were prepared using a synthetic protocol adapted from a previously reported procedure for acetylide **A[3]** containing a diphenyl sulfone scaffold as the tethered ligand (Figure 2B) (Suzuki et al., 1992). Acetylides **A[7]** through **A[10]** were synthesized by coupling thiols with aryl iodides and subsequent oxidation of the diphenyl sulfides (Figure 2C) (Kwong and Buchwald, 2002; Thomas et al., 2011). Thus, copper-catalyzed C(aryl)-S bond formation between aryl iodides and aryl sulfides, followed by the oxidation of the sulfide with *m*-CPBA, metalation with *n*-butyllithium, and subsequent treatment with *in situ* generated Bi(Br)_2_Ph yielded triphenyl bismuth derivatives (Figure 2A). Iodination of the triphenyl bismuth species followed by C(sp)-Bi coupling provided the bismuth(III) acetylides **A[7–10]**. While these substrates have not been used for the *in situ* kinetic studies, they were used for comparisons of the solid-state structures.

Strong transannular N→Bi interactions in “butterfly-like” bisorganobismuth(III) bromides were reported as a key factor for their catalytic activity in the oxidation of thiophenols (Toma et al., 2017). To study the bismuth–oxygen intermolecular interactions, the solid-state structures of all synthesized bismuth(III) acetylides were determined by single-crystal X-ray crystallography as well as by the means of solid-state vibrational (IR) and solution phase ^1^H, ^13^C, and ^19^F NMR spectroscopy. The correlation between spectroscopic variables and the effects of systematic variation of substituents is an established approach to examine the electronic nature of aryl compounds (Exner and Boček, 1967; Bazan et al., 2000). A linear dependence on the substituent and its electronic origin is typically observed (Paul et al., 2002) and has been reported for other metal acetylides, such as uranium(VI) (Mullane et al., 2019) and iron(II) (Costuas et al., 2004) acetylides, and 1-alkynyl-pyridines (Yamaguchi et al., 2011). However, the vibrational analysis of the acetylenic C(sp)-C(sp) bonds for compounds **A[1]-[10]** revealed that the corresponding absorbance bands exhibit no linear free energy dependence relative to the Hammett constants. (Supplementary Table S3 in the Supporting Information).

**FIGURE 3:**
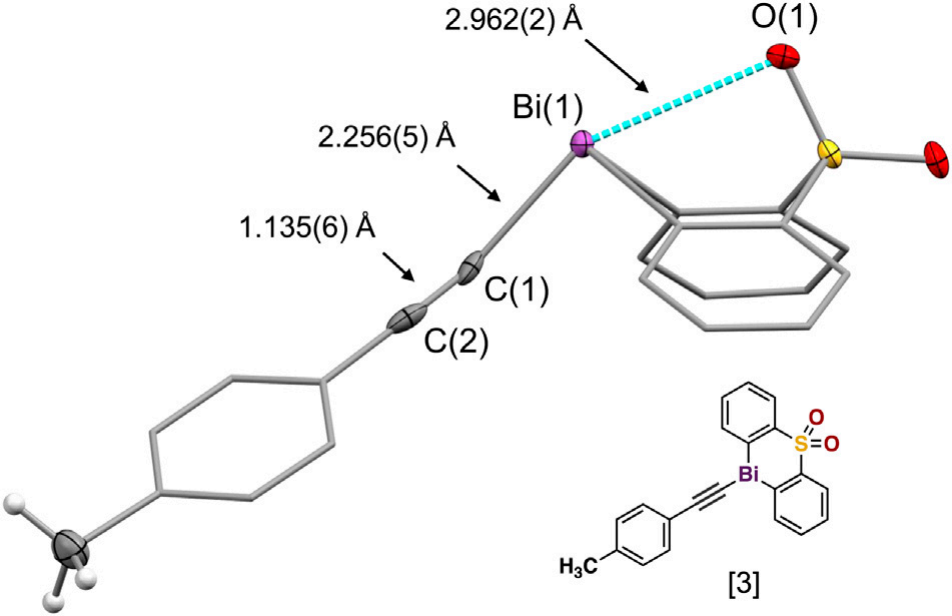
Crystal structure of the bismuth(III) acetylide [3] with labeled Bi(1)-C(1), C(1)≡C(2) and C(2)-C(sp^2^) bonds and transannular Bi(1)-O(1) interaction distances [Å].

Our comprehensive X-ray crystallographic characterization revealed the acetylide structure with the bismuth center in a tethered geometry is influenced not only by the *para*-substituents but also by the axial oxygen atom of the sulfonyl group (Figure 3). Our crystallographic analysis of the *para*-substituted diphenyl sulfone bismuth(III) acetylides **A[1]** to **A[6]**, in particular, the distance of the transannular interactions between Bi(1) and O(1) as well as the C(sp)-C(sp) triple bond distance did not follow the expected linear trend (Hammett trend) (Ohkata et al., 1989) (Table 1). The intramolecular bismuth-oxygen(1) distance in bismuth(III) acetylides with electron-withdrawing substituents in the diphenyl sulfone scaffold, chloro- **A[8]** and trifluoro-methyl- **A[10],** is 2.878(3) Å and 2.874(2) Å that is significantly shorter than the Bi(1)-O(1) distances observed for electron-donating substituted diphenyl sulfone scaffolds, methoxy-**A[7]** and bismethoxy-**A[9]** (2.908(3) Å and 3.035(3) Å, respectively). The complete crystallographic details are given in the Supporting Information.

**TABLE 1:** Selected bond and transannular Bi(1)-O(1) interaction distances of all synthesized bismuth(III) acetylides.

Bi(III) acetylide	O· ·Bi/Å	Bi(1)–C(1)	C(1)≡C(2)	C(2)-C(sp^2^)
*para*-phenyl substituents				
[1]	2.896(2)	2.243(3)	1.180(3)	1.453(3)
[2]	2.998(2)	2.225(3)	1.195(5)	1.453(5)
[3]	2.962(2)	2.256(4)	1.135(6)	1.483(6)
[4]	2.936(2)	2.236(3)	1.202(5)	1.449(5)
[5]	2.972(2)	2.231(4)	1.175(6)	1.458(6)
[6]	2.940(2)	2.210(5)	1.222(6)	1.434(6)
Diphenyl sulfone ligand substituents				
[7]	2.874(2)	2.230(3)	1.211(3)	1.440(3)
[8]	2.878(3)	2.230(4)	1.196(5)	1.444(4)
[9]	2.908(3)	2.221(4)	1.153(6)	1.477(7)
[10]	3.035(3)	2.209(4)	1.198(6)	1.447(6)

### Kinetic Cyclic Voltammetry for Mechanism Elucidation

The electronic functionality of triarylbismuth(V) ligands was reported to directly influence their reactivity (Barton and Finet, 1987; Abramovitch et al., 1988; Combes and Finet, 1999; Finet, 2002). With this in mind, we analyzed the reactivity trends of differently functionalized bismuth(III) acetylides towards organic azides in the copper(I) catalyzed cycloaddition reaction. Being eager to study substrate-dependent reactivity features in the mechanism, we made an effort for stepwise kinetic studies using cyclic voltammetry (CV) (Elgrishi et al., 2017; Graham, 2017; Sandford et al., 2019; Savéant and Costentin, 2019). While commonly used as an electro-analytical method, cyclic voltammetry allowed the direct investigation of the intermolecular transannular effect on bismuth(III) acetylide reactivity towards triazolide formation as well as the redox activity of the catalytic intermediates.

**FIGURE 4:**
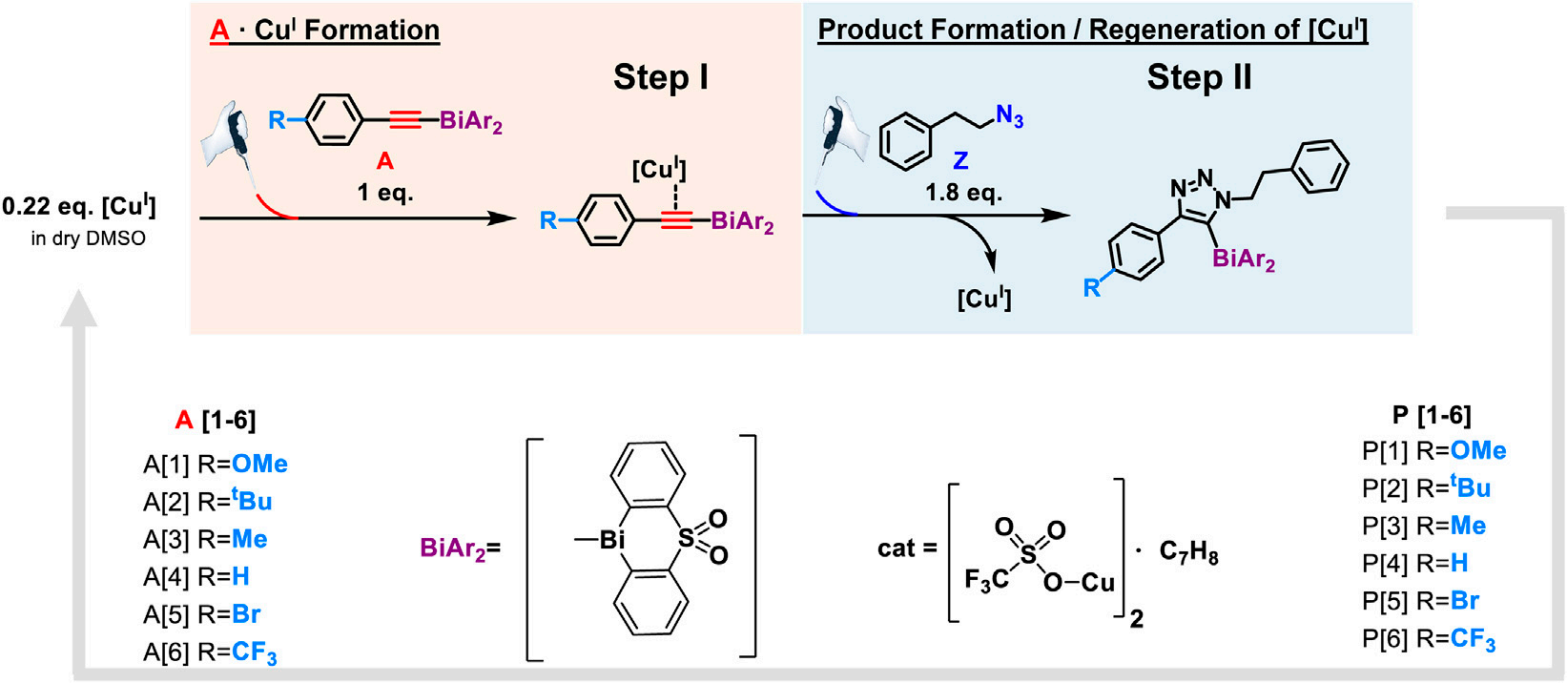
Reaction scheme of kinetic studies of copper(I)-catalyzed reaction of 5-bismuth(III) triazolides formation with cyclic voltammetry technique.

A substantial drawback of the copper(I) system is its air sensitivity; therefore, all electrochemical experiments were performed in the nitrogen atmosphere of a glovebox. Following our developed CV kinetic analysis protocol, we were able to stepwise monitor the redox activity of copper(I) after adding first the acetylide and then the organic azide reactant to access quantitative kinetic parameters of the catalytic cycle (Figure 4). Each electrochemical kinetic experiment was initiated by redox studies of a DMSO solution containing only the copper(I) trifluoromethanesulfonate toluene complex catalyst. The mechanism of the copper(I) catalyzed azide bismuth(III) acetylide cycloaddition has been postulated to initiate with a π-intermediate complex formation of copper(I) and the bismuth(III) acetylide (Worrell et al., 2013a). In a subsequent step, the azide ligation/migratory insertion occurs and reductive elimination of copper(I) yielding the 5-bismuth(III)-triazolide (Worrell et al., 2013a; Worrell et al., 2013b). The kinetics of the two independent steps: 1) π-intermediate complex of copper(I) and the bismuth(III) acetylide [A(X)·cat] formation, 2) product formation/regeneration of (Cu^I^), were monitored by the subsequent addition of the reactants. First, a bismuth(III) acetylide was added and the kinetics of the π-complex formation was monitored by continuously recording cyclic voltammograms until no further changes in the CV responses could be detected. Subsequently, the organic azide was introduced to the mixture and the reaction progress was again monitored by continuously recording cyclic voltammograms.

**SCHEME 1:**
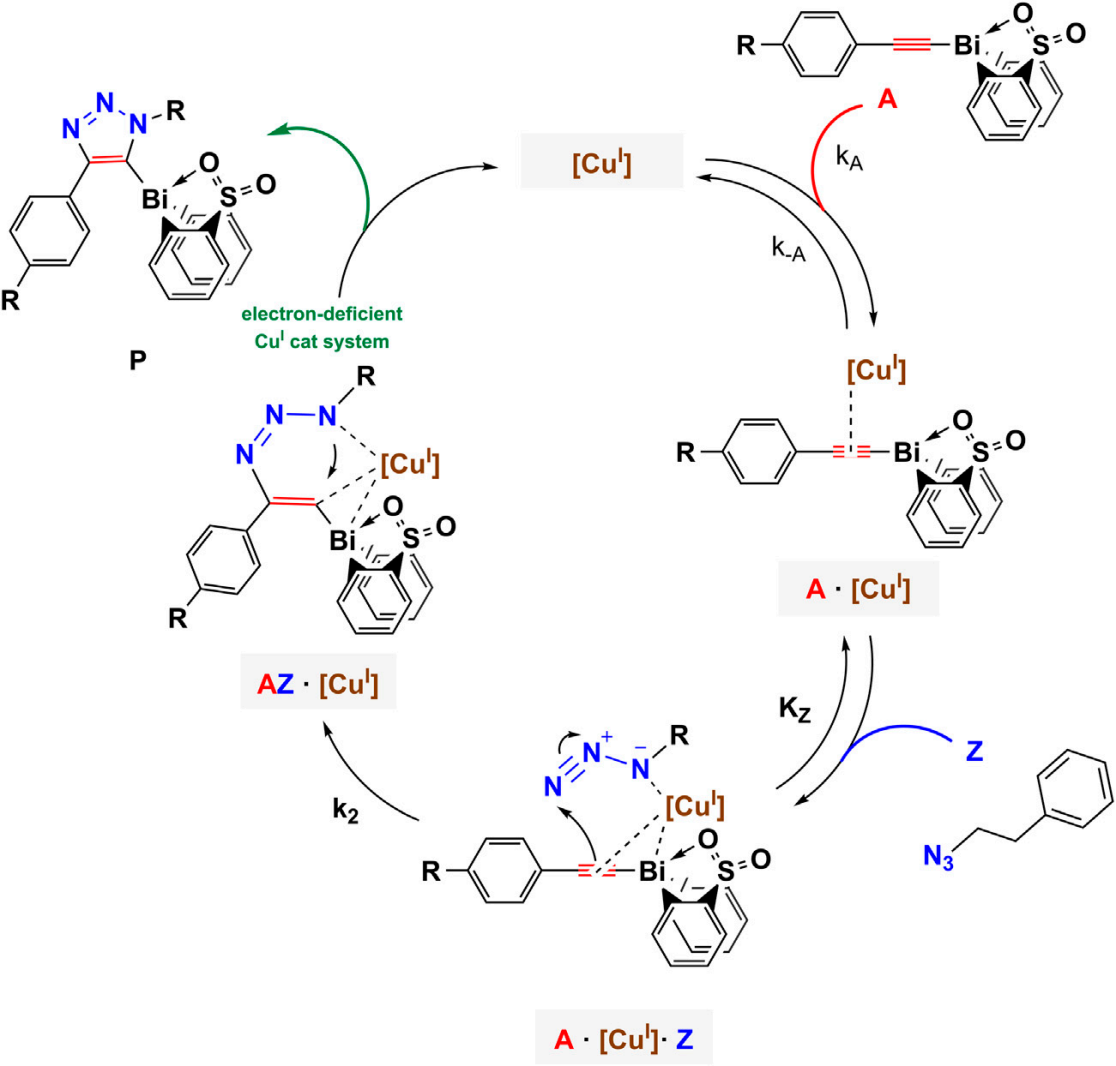
Proposed mechanistic model of azide-bismuth(III) acetylide copper(I)-catalyzed cycloaddition reaction. The electronic nature of the alkyne substrate is expected to directly influence the kinetics of the catalytic steps.

Using *in situ* electrochemistry as an electroanalytical tool provided vital information on the formation of intermediates, which is critical for understanding the catalytic transformation, as well as the ability to dissect the reaction rate constants and parameters from *in situ* experimental data (Scheme 1) (Sandford et al., 2019).

All bismuth(III) acetylides used for the kinetic studies carried the same sterically demanding diphenyl sulfone ligand. Therefore, no significant differences in the steric environment of the copper species in the copper(I)-bismuth(III) acetylide complexes are expected. As for other (metal) acetylides, coordination of the azide to copper(I) in the copper(I)-bismuth(III) acetylide complex is expected to primarily depend on the electronic nature of the copper(I) species (Partyka et al., 2009; Zhou et al., 2010; Yamada et al., 2016; Chung et al., 2018; Ma and Ding, 2020). Therefore, the formation of the first σ-bond between the β-carbon [C(2)] of the acetylide and the terminal nitrogen [N(3)] of the azide should determine the rate-limiting step of the overall transformation (Worrell et al., 2013a).

**FIGURE 5:**
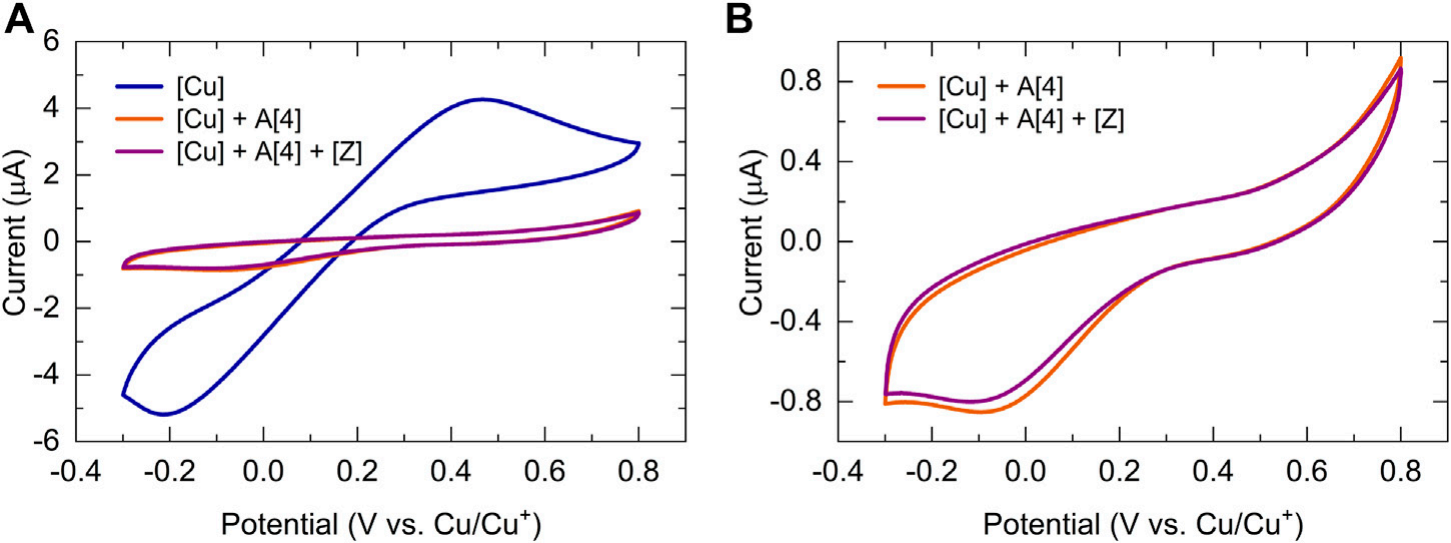
Cyclic voltammogram of bismuth(III) acetylides in the presence of the copper(I) trifluoromethanesulfonate toluene complex at 25°C in dry DMSO recorded at 100 mV/sec. Voltammograms of the **(A)** copper(I) trifluoromethanesulfonate catalyst (blue), after the addition of acetylide (orange), and after stepwise addition of acetylide and azide (purple); **(B)** copper(I) trifluoromethanesulfonate catalyst after the addition of acetylide (orange), and after stepwise addition of acetylide and azide (purple). Conditions: 3-electrode cell with glassy carbon as the working electrode, copper foils as counter, and reference electrodes.

To develop a quantitative model of such a complex catalytic reaction, rate constants, and substituent effects need to be determined. To verify that the CV response is chemically reversible and thus the catalyst and the solvent are suitable for the CV timescale, each kinetic electrochemical experiment was first initiated with cyclic voltammetry studies of a solution of the copper(I) trifluoromethanesulfonate toluene complex catalyst. The cyclic voltammograms of copper(I) complex (1.25 mM, 8.12 μmol) dissolved in dry DMSO (6.5 ml) recorded at 100 mV/s revealed that the reduction of Cu(I) to Cu(0) occurred at around −0.2 V versus the Cu^+^/Cu^0^ reference electrode (Figure 5 **A**, blue). To collect the first set of quantitative kinetic data, we injected an almost five-fold excess of bismuth(III) acetylide, A(X), into a 1.25 mM catalyst solution. The final concentration of the bismuth(III) acetylide substrate was 5.35 mM. The formation of the [A(X)·cat] complex was monitored by continuously recording cyclic voltammograms until no further changes in the CV responses were detected.

Similar cyclic voltammetry studies of Cu(I) complexes with various triazolylamine ligands for the copper(I) catalyzed azide-alkyne cycloaddition reaction have been reported while studying reversible couple characteristics of Cu^I/II^ (Hong et al., 2008). Upon binding to the π-system of the bismuth(III) acetylide, the electrode potentials during the reduction of Cu(I) were found to slightly shift towards the higher potentials for the bismuth(III) substrates. On the contrary, the electrode potentials during the oxidation of Cu(0) to Cu(I) remained identical and stable throughout the reaction. The final CV response after the formation of the [A(X)·cat] complex results in a lowering of the Cu(I) concentration and a consequent decrease in the observed current for the reduction of Cu(I). The copper(I)-bismuth(III) acetylide complex itself is not easily reduced.

**FIGURE 6:**
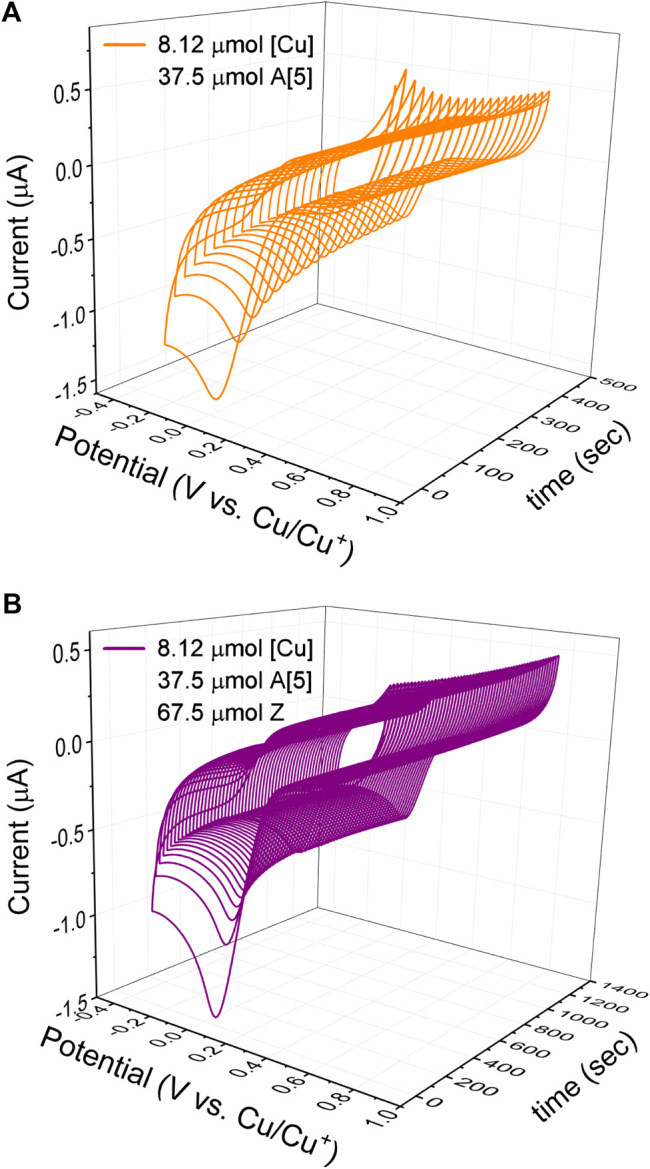
Cyclic voltammetry kinetic studies of the copper(I) catalyst in the 5-bismuth(III) triazolide formation at 25°C in dry DMSO recorded at 100 mV/sec. **(A)** Kinetic studies of [A [5]·cat] complex formation of the copper(I) catalyst and bismuth(III) acetylide (A [5]); **(B)** Kinetic studies of the azide ligation/migratory insertion and 5-bismuth(III)-triazolide, product formation. Conditions: 3-electrode cell with glassy carbon as the working electrode, copper foils as counter and reference electrodes.

The steady decrease in the reduction current for Cu(I) after the addition of bismuth(III) acetylide is consistent with the formation of the copper(I)-bismuth(III) acetylide complex. When the redox cycle indicated the complete formation of the [A(X)·cat] complex, the azide reactant was injected and the processes on Cu(I) were monitored by continuously recording cyclic voltammograms (Figure 6B). Immediately after injection, the characteristic yellow color of the [A(X)·cat] complex began to fade and the recorded current increased due to a sudden release of free copper-catalyst. The electrochemical monitoring was continued until no further changes in current were recorded and the current stabilized at a certain value. The cyclic voltammograms of copper(I), after the formation of the copper-bismuth(III) acetylide complex, and after the azide addition/product formation are shown in Figure 5.

### Kinetic Model Parameter Estimation From Experimental Data

**SCHEME 2:**
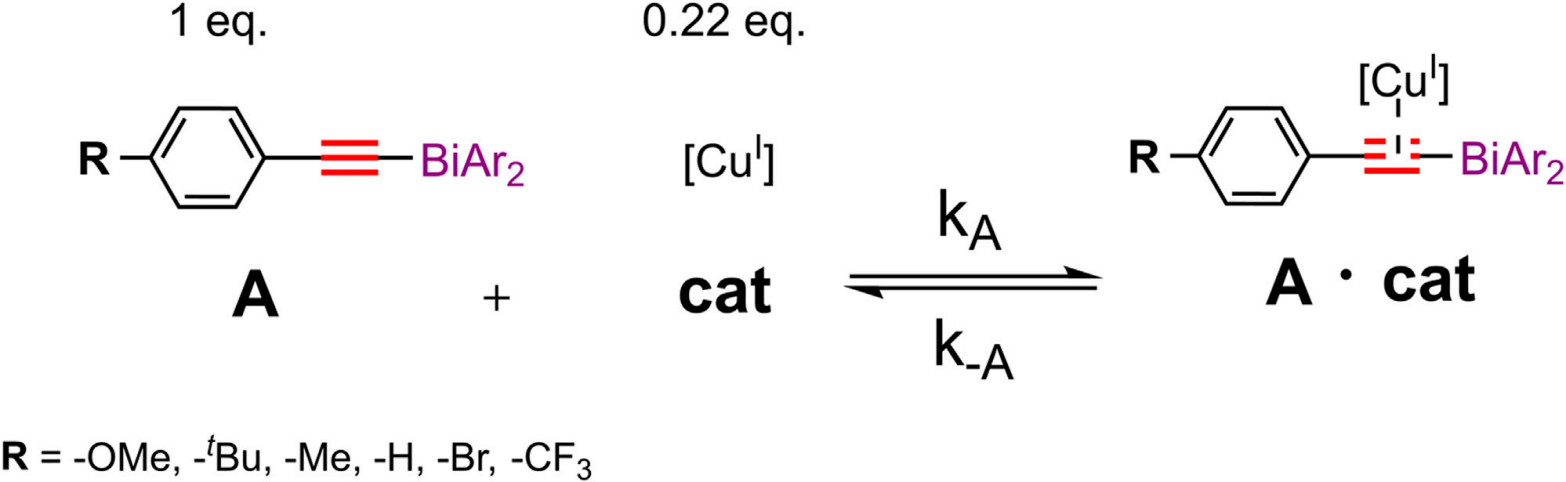
Reaction scheme of the first step, the π-intermediate complex formation of the copper(I) catalyst and bismuth(III) acetylide.

Cyclic voltammetry is a unique tool to extract reaction kinetic parameters, i.e. rate constants by detecting changes in peak currents or potentials versus time (Elgrishi et al., 2016; Hammerich and Speiser, 2016; Passard et al., 2016; Rountree et al., 2016; Haque et al., 2021). *In situ* cyclic voltammetry data was used to determine changes in the free copper(I) concentration, which allowed for the calculation of the kinetic parameters of the bismuth(III) acetylide coordination reactions during 1,2,3-triazolide **P[X]** formation. Since the reaction rate is a function of multiple parameters, a direct (assumption-free) mechanistic analysis would allow accurate and reliable accounting of all factors that influence the evolution of the rate-determining changes throughout the reaction (Ašperger, 2003; Peccoud et al., 2010). By direct analysis, one implies subsuming concentration variations of all involved catalytic species and reactants. Thus, kinetic profiles of concentration versus time for the stepwise experiments can be formulated in form of ordinary differential rate equations (ODEs). For the direct and reverse rate constant elucidation and their impact on the equilibrium processes, ODEs were formulated to solve the mechanistic model of the copper(I)-catalyzed formation of 5-bismuth(III) triazolides (Scheme 2). The catalytic process was investigated stepwise: first, the acetylide **A[X]** addition, and second, the addition of the azide **Z** reactant. Therefore, we were able to quantify the kinetics of the first step, the copper(I)-bismuth(III) acetylide complex formation (Figure 4, red). For simplicity, we denote the copper trifluoromethanesulfonate benzene complex catalyst (Cu^I^) hereafter as (cat).

Considering the equilibrium process of the intermediate [A·cat] formation from [cat] and the bismuth(III) acetylide in the theoretical model (Eq. 1):

$$-\frac{d\left[cat\right]}{dt}=k_{A}\left[A\right]\left[cat\right]-k_{-A}\left[A\cdot cat\right]$$ (1)

Applying the conservation law for the catalyst and bismuth(III) acetylide components yields Equations 2, 3:

$${\left[cat\right]}_{0}=\left[cat\right]+\left[A\cdot cat\right]$$ (2)

$${\left[A\right]}_{0}=\left[A\right]+\left[A\cdot cat\right],$$ (3)

where ${\left[cat\right]}_{0}$ and ${\left[A\right]}_{0}$ are the initial concentrations of the bismuth(III) acetylide and copper(I) catalyst.

Considering the law of conservation of mass, Equations 2, 3, the concentration of the catalyst can be expressed as Eq. 4: (Kaps and Rentrop, 1984; Butcher, 2016; Stone et al., 2018)

$$\frac{d\left[cat\right]}{dt}=-k_{A}\left({\left[A\right]}_{0}-{\left[cat\right]}_{0}+\left[cat\right]\right)\left[\text{cat}\right]+k_{-A}\left({\left[cat\right]}_{0}-\left[cat\right]\right)$$ (4)

The integration was done using a self-developed computer program relying on the fourth-order classical Runge-Kutta method (Runge, 1895; Press and Teukolsky, 1992). The solution of Eq. 4 was found via the grid search using metrics of the LMS optimization:

$$\underset{k_{A},k_{-A}}{\min}\overset{n}{\underset{i=1}{\sum }}{\left({\left[cat\right]}_{exp,i}-{\left[cat\right]}_{i}\right)}^{2},$$ (5)

where the concentration of the catalyst, [cat]_exp_, is derived from experimental kinetic data, and values of [cat] are obtained from Eq. 4, *n* is a number of the experimentally derived concentration values at *n* time intervals.

Based on the Randles-Sevcik equation (Hickey et al., 2019), the peak current in a cyclic voltammogram is directly proportional to a concentration of the species that participate in the electron transfer, Equation 6:

$$i_{p}={\left(2.69\cdot 10\right)}^{5}{\sqrt{n}}^{3}A\sqrt{D}\sqrt{\nu }C,$$ (6)

where $i_{p}$ is the peak current (in our case of reduction current), *n* is the number of participating electrons, *A* is the electrode area, *D* is a diffusion coefficient, ν is the scan rate, *C* is the unknown concentration of the (cat) species at a time corresponding to a certain peak current, $i_{p}$ .

From the first set of kinetic data in Figure 6A for the bismuth(III) acetylide addition, we derived a linear proportionality coefficient α for each kinetic experiment of the six different acetylides while keeping the value of the initial copper(I) catalyst concentration constant as given by Equation 7:

$$i_{p}=\alpha {\left[cat\right]}_{0},$$ (7)

where $i_{p}$ was denoted as the peak of the anode current and ${\left[cat\right]}_{0}$ is the initial concentration of the catalyst.

The concentration of (cat) over time was obtained from $i_{p}$ versus time data from the kinetic CV experiments using Eq. 7. The complete details on the individual [cat] over time studies for all six bismuth(III) acetylides are given in the Supporting Information (Supplementary Figure S3A–F). The equilibrium constant $K_{A}$ was determined from Eq. 4 while having results on separate rate constants of the direct and reverse reaction of the [A·cat] formation. As the rate of the [cat] coordinating to the bismuth(III) acetylide equals zero at equilibrium, we can solve the quadratic equation (Eq. 4) for the equilibrium concentration (cat)_eq._ (the corresponding algebraic expressions are given in the Supporting Information, Supplementary Equation S4).

**FIGURE 7:**
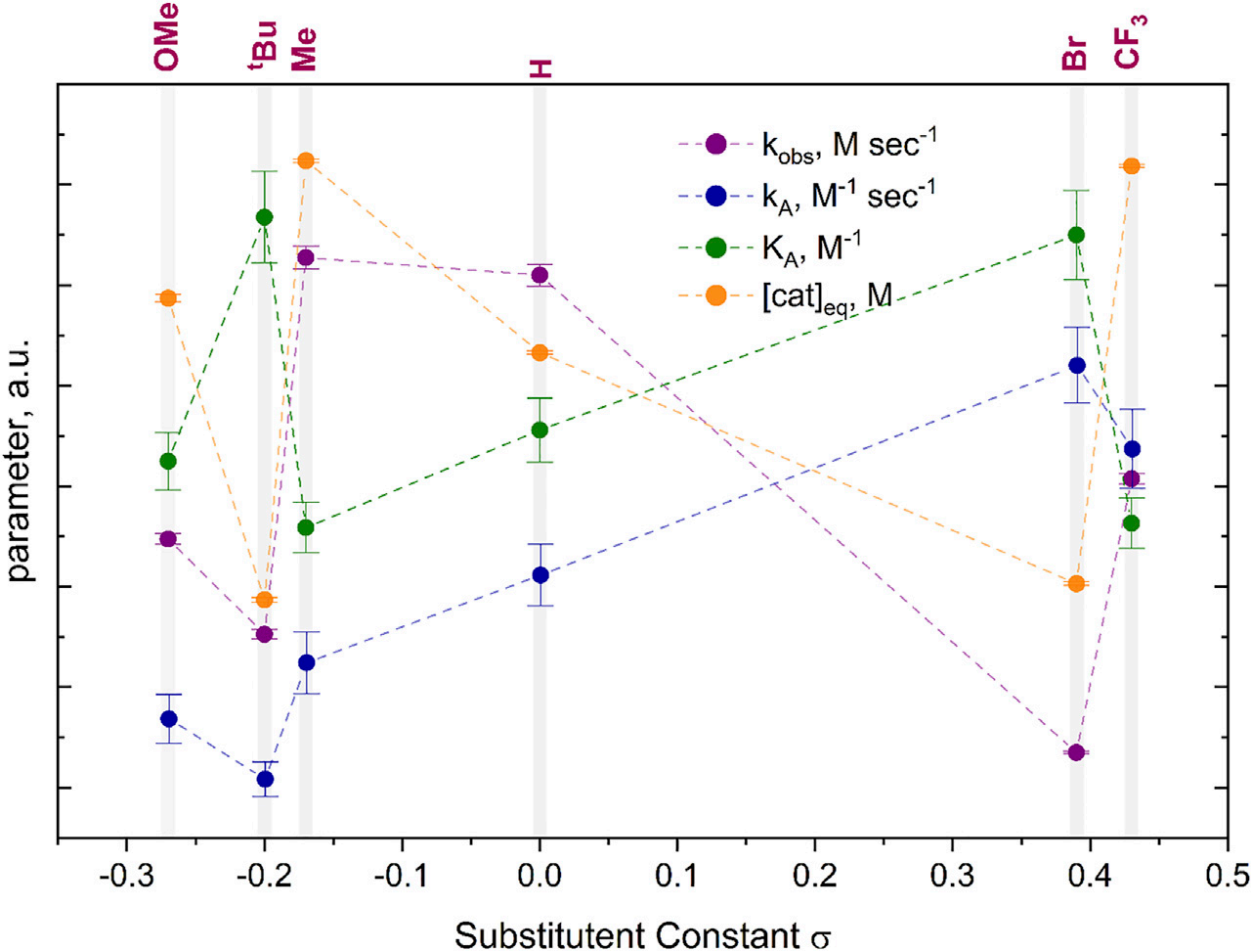
Kinetic parameters K_A,_ k_obs,_ $k_{A}$ and [cat]_eq._ were obtained from cyclic voltammogram and ^1^H NMR kinetic studies for each of the six studied bismuth(III) acetylides. The electrochemically determined equilibrium constant of the [A·cat] complex formation K_A_ (green) is inversely correlated to the overall rate constant determined by ^1^H NMR kinetic experiments of the 5-bismuth(III)-triazolide formation k_obs_ (purple) and equilibrium catalyst concentration [cat]_eq._ (orange) plotted vs the Hammett sigma values of the six studied para-substituents.

The forward reaction rate parameter $k_{A}$ for the (A·cat) complex formation of the differently substituted bismuth(III) acetylides follow a non-linear trend in activity when compared to the corresponding equilibrium concentration (cat)_eq._ values. The reaction equilibrium parameter $K_{A}$ for the (A·cat) complex formation was found to be inversely correlated in terms of values when compared to the corresponding equilibrium concentrations (cat)_eq._ (Figure 7). Indeed, the more the equilibrium is shifted in the forward direction, the more (A·cat) species are formed, thus the lower would be the equilibrium concentration of the free catalyst (cat). This confirms our assumption that the activity of bismuth(III) acetylides of the copper(I)-catalyzed cycloaddition with organic azides is not solely directed by *para*-phenyl substituents as has been reported for proto and iodoalkynes (Chung et al., 2017; Nazarova and Fokin, 2017; Nazarova and Fokin, 2019).

After determining the equilibrium constant, $K_{A}$ , of the π-coordination of the bismuth(III) acetylides to the copper(I) catalyst, we applied the same approach to the estimation of the azide ligation/migratory insertion rate parameters. The direct investigation of the azide coordination/insertion dependent rate constants is not possible without being able to detect the azide (Z) consumption separately. The addition of the azide to a solution of the (A·cat) π-complex (stabilized CV response) resulted in an immediate increase in the reduction current. Before the addition of the azide, the mixture contained dominantly the (A·cat) species. Upon addition, the product, 5-bismuth(III) triazolide, was formed and a large amount of free copper catalyst was released at once causing the sudden increase in peak current (Figure 6B). Thereafter, the peak current returned to the current levels observed for the (A·cat) complex formation. This makes the copper(I)–bismuth(III) acetylide complex formation the faster process. Subsequently, we can conclude that the azide ligation/migratory insertion is the rate-determining step (RDS), as a continuous accumulation of the (A·cat) complex was observed. While the coordination of the ɑ-nitrogen (N1) to copper(I) is influenced primarily by the electronic environment of the copper(I) species, the formation of the first covalent bond between acetylene β-carbon and terminal nitrogen of the azide moiety is also expected to influence the kinetics of the reaction (Scheme 1).

Overall, the rate constants for the formation of the π-complex of copper(I) for all six studied bismuth(III) acetylides were found to confirm the reactivity trend determined in the ^1^H NMR kinetic studies. While previous studies of (metal)acetylides in the copper(I)-catalyzed cycloaddition with azides followed a linear (Hammett) reactivity trend, (Brown and Widenhoefer, 2011; Powers et al., 2015), the observation of a non-Hammett-dependent reactivity of the bismuth(III) acetylides indicates that, apart from *para*-phenyl substituents, other effects such as transannular electron density donation of oxygen to the bismuth center play a role in the control of the reaction kinetics. In the solid-state, the transannular Bi(1)-O(1) distance for the least reactive acetylide, the *para*-bromo substituted **A[5]**, is one of the largest, whereas the more reactive substrates, **A[3]**, **A[4]**, and **A[6]**, have shorter Bi(1)-O(1) bond distances. We believe that such perturbations not only affect the electron density of the C(β)-acetylene carbon but subsequently also influence the electronic environment of the copper(I) species in the (A·cat) complex. This causes different affinities of copper(I) species towards N→Cu(I) ligation (Scheme 1). This supports our previous conclusion of the overall reaction to be azide-dependent.

**FIGURE 8:**
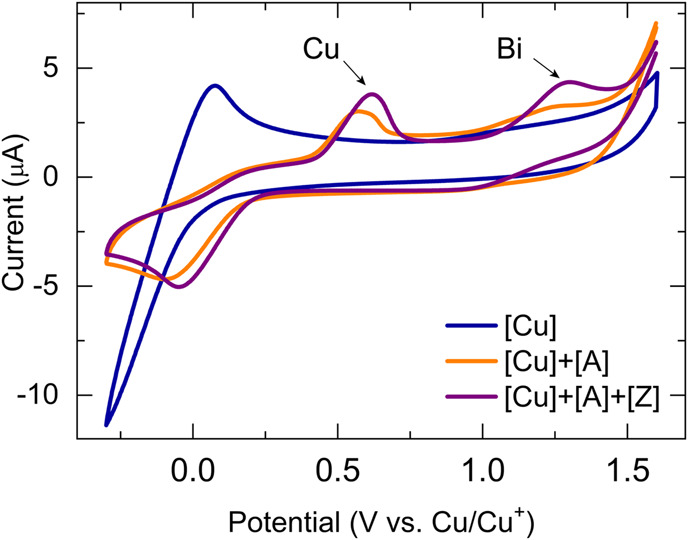
Cyclic voltammogram kinetic studies of bismuth(III) acetylide in the presence of the copper(I) trifluoromethanesulfonate toluene complex at 25°C in dry DMF at 100 mV/sec. Conditions: 3-electrode cell with glassy carbon as the working electrode, copper foils as counter, and reference electrodes.

Increasing the potential window past the Cu(I)/Cu(0) redox pair allowed the observation of electrochemically relevant processes at the bismuth center in the bismuth(III) acetylides (Figure 8). As expected, the formation of the copper(I)–bismuth(III) acetylide complex also impacts the electronic environment of the bismuth(III) center.

Our detailed mechanistic modeling performed in the context of CV independent reactivity experiments revealed the electronic effects of *para*-phenyl functionalization together with transannular interaction determine substrates rate parameters and reactivity. Since the reaction rate is influenced by multiple parameters, a direct (assumption-free) mechanistic analysis allowed the accurate and reliable examination of all factors that influence the complex catalytic formation of 5-bismuth(III)-triazolides.

### Kinetic ^1^H NMR Spectroscopic Experiments Supporting the CV-Derived Quantitative Mechanistic Model

**SCHEME 3:**
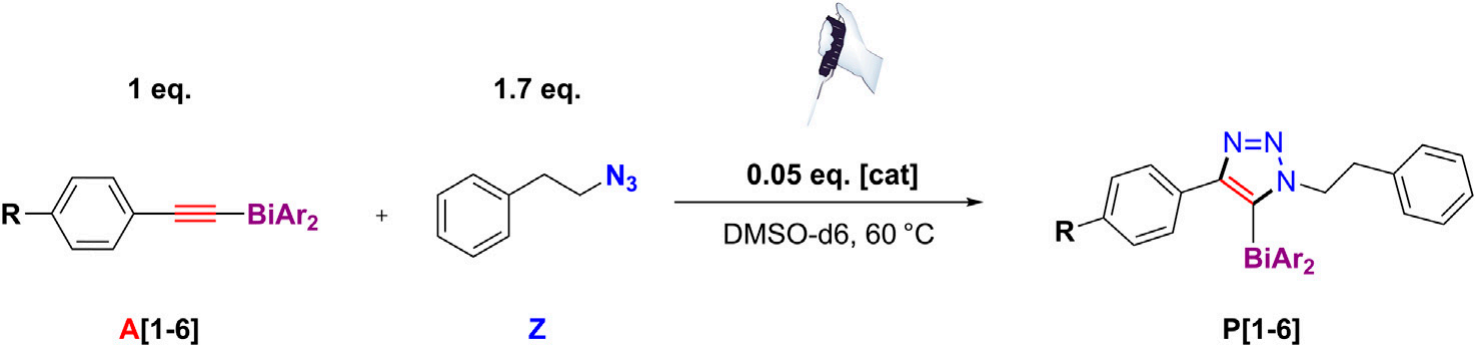
Reaction scheme of the *in situ* ^1^H NMR spectroscopy kinetic studies of copper(I)-catalyzed cycloaddition reaction of bismuth(III) acetylides with an organic azide.

To confirm the kinetic model derived from cyclic voltammetry, the reactant consumption and product formation during the catalytic transformation was continuously monitored via *in situ* ^1^H NMR spectroscopy. An NMR tube was loaded with one of the six different bismuth(III) acetylides, **A[X]**, excess of (2-azidoethyl)benzene, and the copper(I) trifluoromethanesulfonate toluene complex [cat], according to the reaction shown in Scheme 3.

^1^H NMR kinetic studies were performed in deuterated dimethyl sulfoxide due to its high boiling point to achieve better signal separation (Socrates, 1967; Kaufman et al., 1982; von Harbou et al., 2017). A positive side-effect of recording the spectra at elevated temperatures was the elimination of apical and equatorial ligand rotations due to Berry pseudo rotation (BPR) and Turnstile rotation (TR) processes on the NMR time scale, allowing precise peak integration. At room temperature, these processes cause a broadening of the ortho-hydrogens in the diphenyl sulfone scaffold which makes accurate signal integration difficult (see Supplementary Figure S6 in the Supporting Information) (Ugi et al., 2002). Our NMR-spectroscopic kinetic investigations match the conclusion made in the cyclic voltammetry studies, i.e. the transformation to be predominantly or exclusively azide-substrate dependent. However, the atypical reactive trend was also found in the ^1^H NMR kinetic study and is in good agreement with the overall trend imposed by the equilibrium concentrations ${\left[cat\right]}_{eq.}$ (Table 2; Figure 7). During competing experiments with terminal acetylenes, the exclusive formation of bismuth(III)-triazolides was observed. This indicates a copper(I) catalyst preference for bismuth(III) acetylides. Catalyst robustness experiments showed that this is also true in the presence of residual moisture (Supplementary Figures S7, S8, and S9 in the Supporting Information).

**TABLE 2:** Rate parameters k_A_, k_−A_, K_A_, [cat]_eq._, derived from the cyclic voltammetry kinetic studies at 25°C in dry DMSO at 100 mV/sec and k_obs_, obtained by ^1^H kinetic NMR at 60°C in dry DMSO-d6, with corresponding standard deviations.

Entry	σ_*para*_	k_A,_ M^−1^ sec^−1^	σ(k_A),_ M^−1^ sec^−1^	k_obs_·10^−5^, M·sec^−1^	σ(k_obs_)·10^−7^, M·sec^−1^	K_A_·10^3^, M^−1^	σ(K_A_)·10^3^, M^−1^	[cat]_eq_·10^−5^, M	σ([cat]_eq_)·10^−5^, M
[1], R = OMe	−0.27	2.39	0.0598	1.19	0.70	0.85	0.0215	24.32	0.033
[2], R = ^*t*^Bu	−0.20	1.95	0.0415	0.81	0.63	1.40	0.0344	16.32	0.020
[3], R = Me	−0.17	2.80	0.0754	2.31	1.53	0.70	0.0189	27.97	0.016
[4], R = H	0.00	3.44	0.0749	2.24	1.47	0.92	0.0240	22.88	0.015
[5], R = Br	0.39	4.97	0.0923	0.34	0.17	1.36	0.0337	16.75	0.016
[6], R = CF_3_	0.43	4.36	0.0965	1.43	0.70	0.71	0.0190	27.83	0.013

**FIGURE 9:**
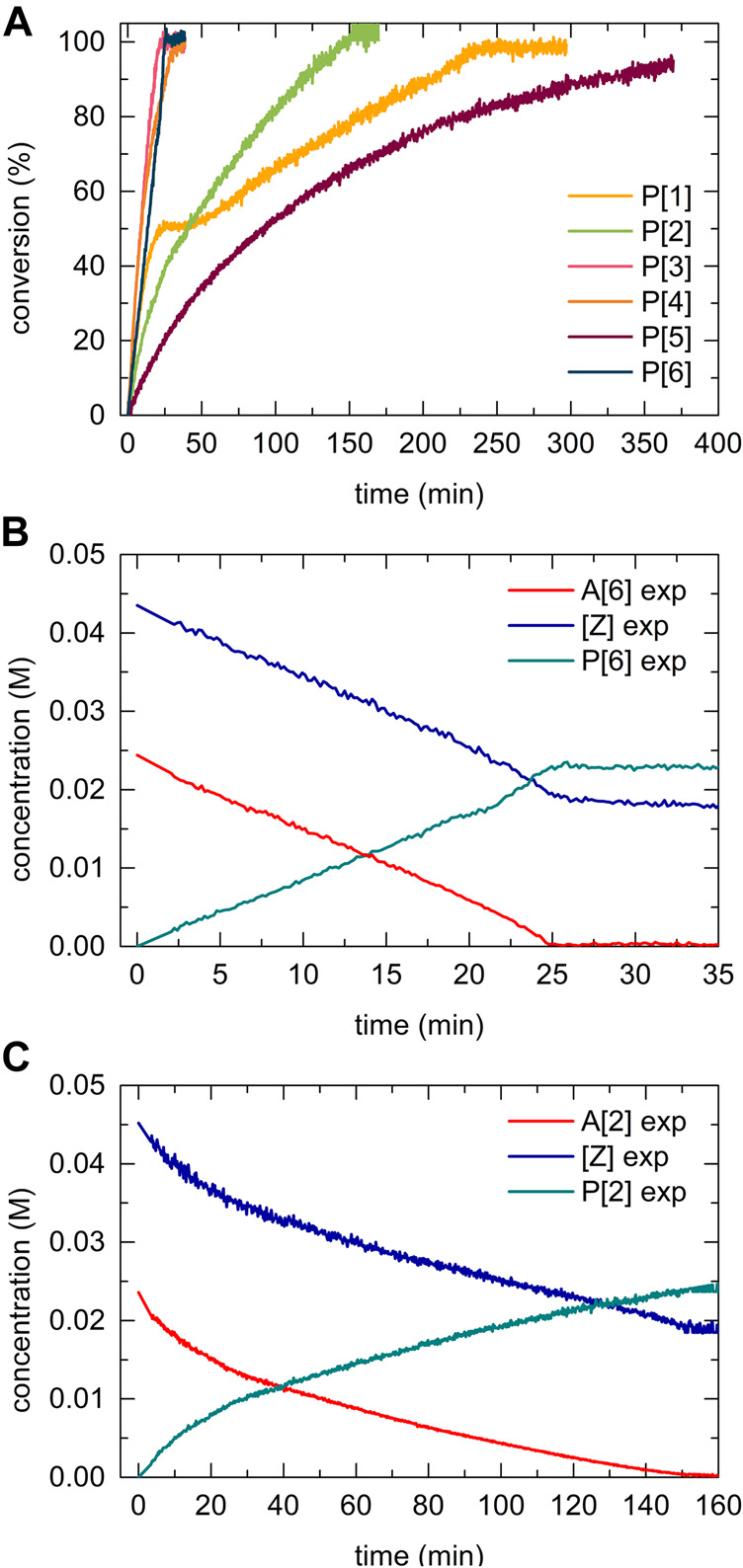
Kinetic profiles of independent experiments of copper(I)-catalyzed cycloaddition reaction of bismuth(III) acetylides with an organic azide. Conditions: A [X] = 25mM; [Z] = 43 mM [cat] = 1.25 mM; 0.8 ml volume DMSO-d6; T = 59.5°C. **(A)** Standard reaction conversion vs. time **(C)** Concentration vs. time for trifluoromethyl functionalized substrate A [6], linear dependence reveals “zero-order” kinetics; **(B)** Concentration vs. time for *tert*-butyl functionalized substrate A [2], exponential dependence reveals positive value rate-order “first-order” kinetics.

A drastic difference in the reaction rate for the formation of **P[3]**, **P[4]**, and **P[6]** (methyl-, proto-, and trifluoromethyl-substituted) compared to the other 5-bismuth(III)-1,2,3-triazolides, **P[1]**, **P[2]**, and **P[5]**, was observed (Figure 9A). The derived concentration vs times curves demonstrated either a linear or exponential dependence for reactant consumption or production formation. Exemplary, the kinetic profiles for trifluoromethyl **A[6]** and *tert*-butyl **A[2]** substituted bismuth(III) acetylides are shown in Figure 9B,C, respectively. This allowed the differentiation between zero-order kinetics and positive value rate orders for fast- or slow-reacting acetylides, respectively.

**FIGURE 10:**
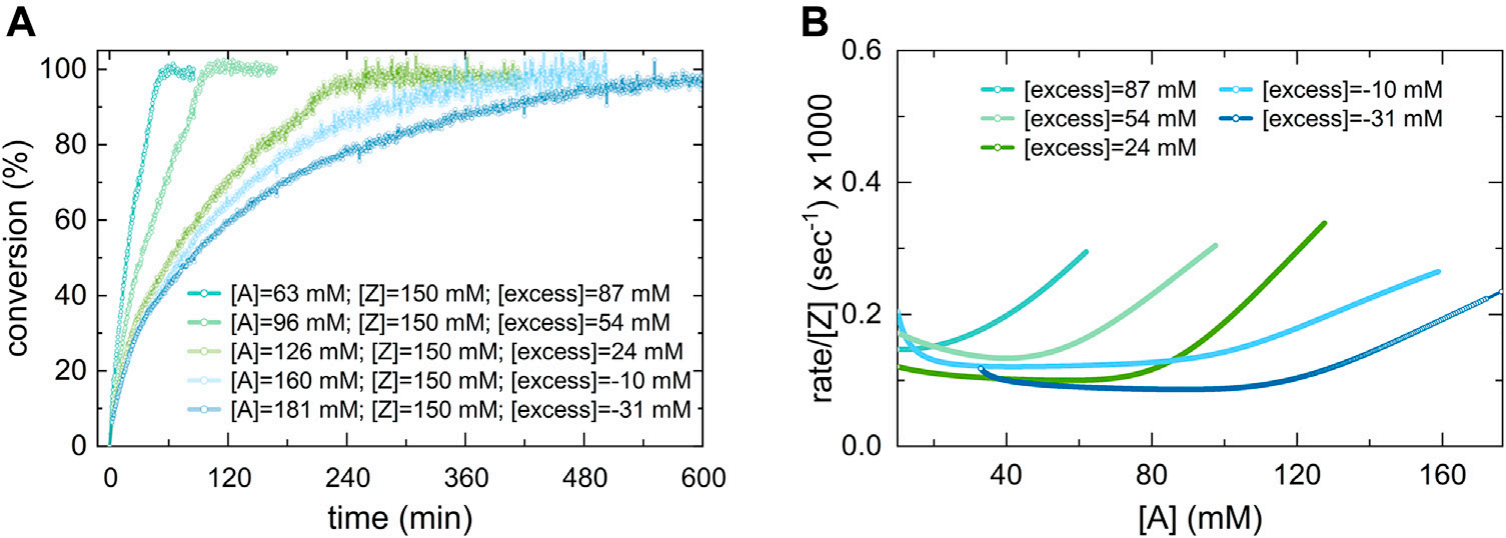
Kinetic profile of different-excess-experiments of the copper(I) catalyzed 5-bismuth(III) triazolide formation using A [2]. **(A)** Variable [A] concentration experiment, conversion vs. time. **(B)** rate/[Z] vs. A [2], the overlapping of the [excess] trials in the region of higher conversions signalizes a near to zero-type order in acetylide [A] substrate. Whereas, in the initial kinetic regime (at low conversions) the acetylide [A] shows non-zero rate order.

Utilizing the reaction progress kinetic analysis approach (Blackmond, 2005), various (“excess”) experiments were performed to derive the rate order and estimate whether we observe a rate-determining step shift for *para*-substituted acetylide substrates as previously reported for terminal alkynes in the copper(I)-catalyzed azide alkyne cycloaddition (CuAAC) reaction (Seath et al., 2017). For the *para*-*tert*-butyl substituted acetylide (2), the “excess” value was defined as the difference between the concentrations of azide and acetylide (2) reactants at the initial time, t = 0 (Figure 10A):

$${\left[Z\right]}_{x}={\left[A\right]}_{x}+\left["excess"\right]$$ (8)

Normalized data representation for all experiments indicates that the reaction rate law exhibits non-zero bismuth(III) acetylide dependency at low conversion (beginning of the reaction) (Figure 10B). Towards the end of the reaction, zero-order or acetylide-independent but azide-dependent kinetics were found (Figure 10B). The highly reactive trifluoromethyl-*para*-substituted acetylide (6) proceeds with azide coordination as the turn over limiting step (zero-order in alkyne substrate) during the entire course of the reaction which results in the linear dependency of the concentration over time (Figure 9B).

Similar to the stepwise kinetic CV studies, the ^1^H NMR monitoring revelated that the overall reactivity is independent of the substituent constants (Hammett sigma values). However, two clusters of substrates with different reactivity trends were identified. We hypothesize that a combination of *para*-substituent functionality and transannular coordination of the oxygen of the diphenyl sulfone scaffold influences the electron density and reactivity of the acetylenic triple bond making it more or less prone for the coordination to copper(I) in the first step.

In addition, the ^1^H NMR kinetic study showed that the overall reaction rates (*k*_*obs*_) are directly correlating with the electronic environment of copper(I) in the (A·cat) complex as found electrochemically by the means of the equilibrium catalyst concentration [(cat)_eq_.]. However, the equilibrium parameter of the π-complex formation between bismuth(III) acetylide and copper(I) catalyst exhibits an inverse correlation with *k*_*obs*_. This indicates a slow 5-bismuth(III)-triazolide formation for cases where the coordination of the bismuth(III)-acetylide to copper(I) is fast and *vice versa.* Indeed, the higher is the affinity of the bismuth(III) acetylide to copper(I), the weaker the coordination of the azide during the ligation/migratory insertion event, thus exhibiting lower overall reaction rates.

## Conclusion

The mechanistic elucidation of the copper(I) catalyzed cycloaddition reaction of bismuth(III) acetylides and organic azides was accomplished by kinetic *in situ* electrochemical (CV) as well as ^1^H NMR spectroscopic techniques. Novel insights into the catalytic model of the 5-bismuth(III) triazolide formation were gained. A non-Hammett-dependent reactivity trend of the bismuth(III) acetylides was observed. We attribute the unexpected non-linear reactivity trend to the more complex nature of the bismuth acetylides that is not solely influenced by Hammett substituent effects. In bismuth(III) acetylides, the reactivity of the C(sp)C(sp) triple bond is affected by various factors including transannular donation from sulfonyl oxygen to the bismuth(III) center and electron-withdrawing or -donating functional groups in *para* position of the phenyl ring.

The CV kinetic experiments enabled the stepwise investigation of the catalytic cycle. Electrochemical monitoring of the formation of the putative intermediate complex of the copper(I) catalyst and the bismuth(III) acetylides and the computed equilibrium catalyst concentration [(cat)_eq._] were used to categorize the different acetylide substrates by reactivity. The azide ligation/migratory insertion was found to be the rate-determining step. The rate-determining step is influenced by both, the tendency of the ɑ-nitrogen (N(1) of the azide to ligate with the copper metal and the ability of the azide to form the first covalent bond between the β-carbon of the acetylide and terminal nitrogen [N(3)].

The equilibrium constant of the (A·cat) complex formation, $K_{A}$ , correlates with the equilibrium concentrations (cat)_eq._ trend, revealing the faster the formation of the (A·cat) intermediate the lower is the residual amount of free copper(I) catalyst species.

The experimentally derived reaction rate constants of the corresponding π-intermediate equilibrium constant (*K*_*A*_) and the triazolide formation (*k*_*obs*_) determined utilizing kinetic cyclic voltammetry and nuclear magnetic resonance studies are inversely correlated. Thus, the stronger the coordination of the copper(I) and bismuth(III) acetylide the weaker the azide ligation to copper(I), which results in slower overall reaction rates. All our findings as well as the results of the quantitative modeling support the proposed mechanistic model of the copper-mediated 5-bismuth(III)-1,2,3-triazolide formation.

## Data Availability

The original contributions presented in the study are included in the article Supplementary Materials.
