# Supplementary material for: Electrochemical Studies of the Cycloaddition Activity of Bismuth(III) Acetylides Towards Organic Azides Under Copper(I)-Catalyzed Conditions
Source: Front Chem. 2022 Feb 25;10:830237. doi: 10.3389/fchem.2022.830237 (PMC9531323; doi:10.3389/fchem.2022.830237)
Supplement: Supplementary file 1 [file DataSheet1.pdf]

Supplementary Material

1. 1. Cyclic Voltammetric (CV) kinetic studies........3

1.1 General information 3

1.2 Experimental details........ 3

1.3 Data analysis 3

1.4 General procedure A – Determination of the rate constant of bismuth(III)-acetylide – copper(I) complex formation 4

1.5 General procedure B – Determination of the apparent rate constant of the bismuth(III) triazolide[X] formation 7

1.5.1 Determination of reaction rate parameters........11

1.5.2 Derivatization of the catalyst concentration at the equilibrium ([cat]eq.)........15

1.5.3 Standart deviations 15

2. 2. ^{1}H NMR kinetic reactivity studies 17

2.1 General information 17

2.2 Experimental details........ 17

2.3 Data analysis 17

2.4 General procedure C – Independent bismuth(III)-acetylide reactivity experiments...... 18

2.4.1 Determination of reaction rate parameters........21

2.5 General procedure D - Rate orders experiments........ 24

2.6 Dynamic NMR spectroscopy 30

2.7 General Procedure E - Protoalkyne/ bismuth(III) acetylide competitive experiments .. 32

2.8 Moisture compatibility experiments........ 34

2.9 General procedure F – catalyst robustness experiments 35

3. 3. Synthesis and characterization of bismuth(III)-acetylides and 5-bismuth-1,2,3-triazolides37

3.1 General Procedure 1. Synthesis of diphenyl sulfides........ 39

3.2 General Procedure 2. Synthesis of sulfonyldibenzenes 43

3.3 General Procedure 3. Synthesis of 10-phenyl-10H-dibenzo[b,e][1,4]thiabismine 5,5-dioxides 48

3.4 General Procedure 4. Synthesis of 10-iodo-10H-dibenzo[b,e][1,4]thiabismine 5,5-dioxides 53

3.5 General Procedure 5. Synthesis of bismuth(III) acetylides with a diphenyl sulfone scaffold 58
3.6 General Procedure 6. Synthesis of 5-bismuth(III) triazolides 68
4. 4. X-Ray Crystallographic Details 74
5. 5. ^{1}H, ^{13}C and ^{19}F NMR Spectra 131
6. 6. References 175

1. Cyclic Voltammetric (CV) kinetic studies

1.1 General information

Anhydrous DMSO was purchased from MilliporeSigma and used without further purification. Copper(I) trifluoromethanesulfonate toluene complex (≥99.7%) was purchased from Sigma-Aldrich.

1.2 Experimental details

All glassware was oven-dried (140°C) and purged by vacuum-N_2 cycles in the antechamber of the glovebox before use. All electrochemical measurements were performed inside a glovebox under a nitrogen atmosphere. Anhydrous DMSO served as the solvent. A 3-electrode glass cell with a glassy carbon working electrode (BASi, MF-2012), copper foil as the counter and reference electrodes, and copper(I) triflate in anhydrous DMSO) was used for the measurements.

Electrochemical data was collected on a multi-channel potentiostat (AMETEK Scientific Instruments, VersaSTAT 4). Voltammogram at a scan rate of 100 mV s^{-1} was performed on the copper compound between -0.3 V and 0.8 V vs. Cu^+/Cu^0.

^{1}H NMR spectra were obtained on a Varian 600 MHz. Chemical shifts were reported in ppm referenced at 2.5 ppm of DMSO-d6 solvent.

1.3 Data analysis

Kinetic cyclic voltammetry measurements were processed as current versus time dependences. Graphical interpretation of processed experimental data was performed in Origin 9.0 data analysis and graphing software package (OriginLab Corporation) or Microsoft Excel 2016.

1.4 General procedure A – Determination of the rate constant of bismuth(III)-acetylide – copper(I) complex formation

Independent reactivity experiments: Stock solutions of the corresponding bismuth(III) acetylides (0.075 M), and copper(I) triflate (0.016 M) was prepared in anhydrous DMSO. 0.5 mL of the copper(I) triflate toluene complex stock solution and 6.0 mL of DMSO were transferred into the 3-electrode cell, agitated, and used to performed cyclic voltammetry studies of three redox cycles (to ensure reproducibility of the data and the stability of copper(I) triflate complex).

Kinetic CV measurements were recorded in the same 3-electrode cell with a glassy carbon working electrode, copper foils as counter, and reference electrodes. A solution of the bismuth(III) acetylide[X] stock solution in anhydrous DMSO (0.5 mL) was introduced into the electrochemical cell and agitated for some time before the start of the electrochemistry data acquisition. Continuous electrochemical data collection at 100 mV s^{-1} was performed until no further changes in the redox cycles were observed.

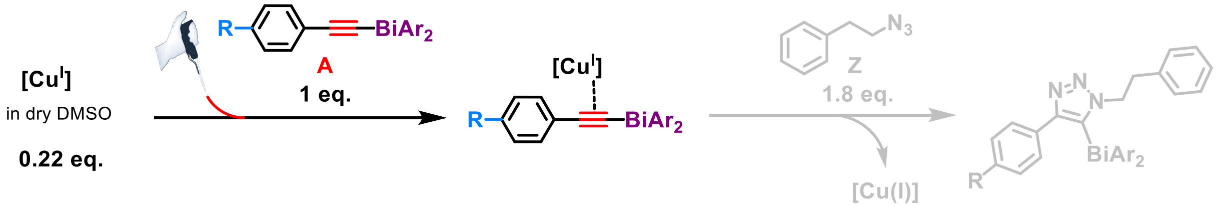

Table S1. Reactions conditions of experiments following general procedure A.

Entry	A[X], mM	AX, µmol	[cat], mM	cat, µmol	T, ˚C
**1**	[1], 5.35	[1], 37.5	1.16	8.12	28.0
**2**	[2], 5.35	[2], 37.5			
**3**	[3], 5.35	[3], 37.5			
**4**	[4], 5.35	[4], 37.5			
**5**	[5], 5.35	[5], 37.5			
**6**	[6], 5.35	[6], 37.5			

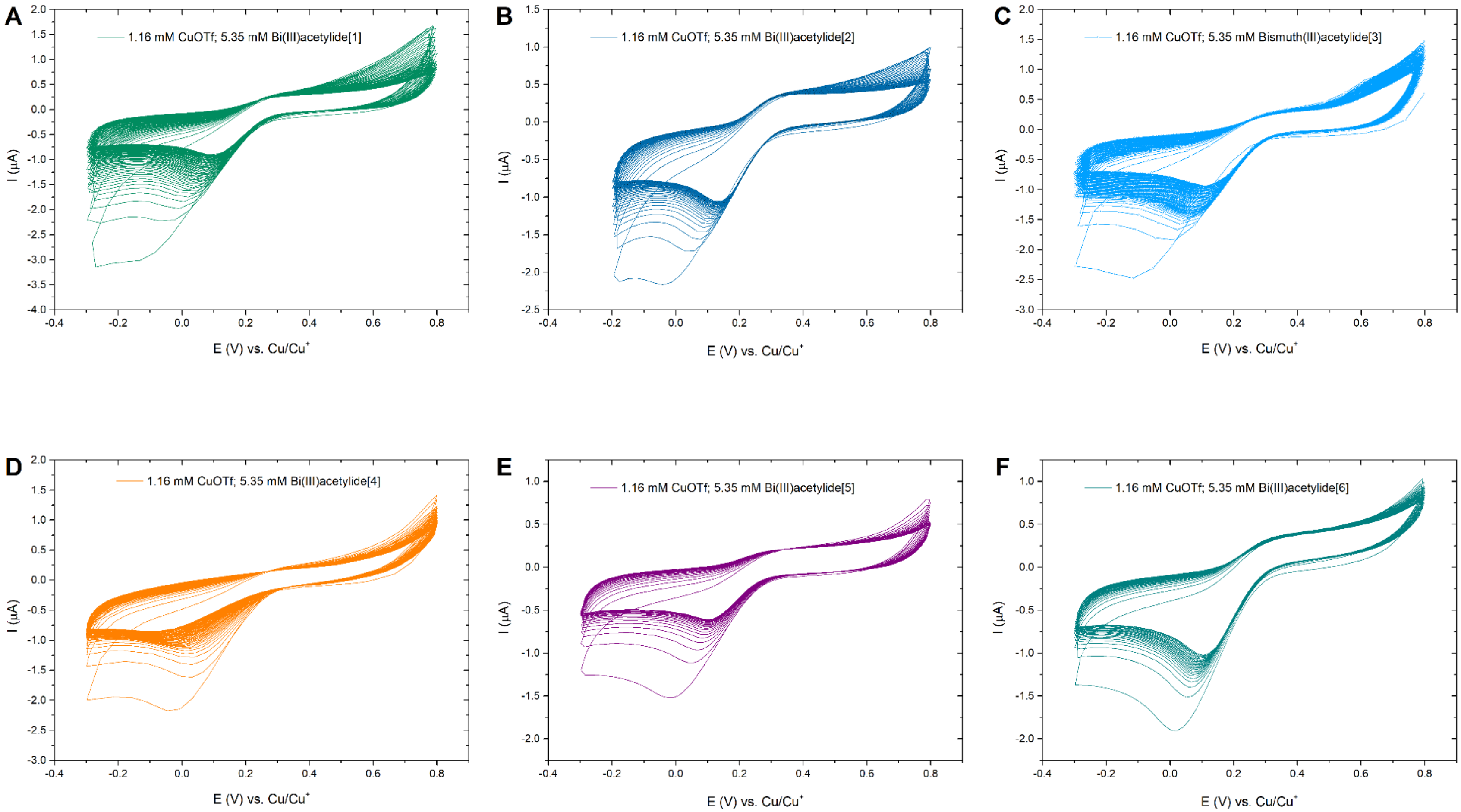

Figure S1. Kinetic profiles A-F of the cyclic voltammograms were obtained by following general procedure A.

1.5 General procedure B – Determination of the apparent rate constant of the bismuth(III) triazolide[X] formation

Kinetic CV experiments were recorded in a 3-electrode cell with a glassy carbon working electrode, copper foils as counter and reference electrodes. A solution of the (2-azidoethyl)benzene (0.135M) in anhydrous DMSO (0.5 mL) was introduced to an electrochemical cell with already preformed π-intermediate (general procedure A). The mixture was agitated for some time before the start of the electrochemistry data acquisition. Continuous electrochemical data collection at 100 mV s^{-1} was performed until no further changes in the redox cycles were observed. Electrochemical measurements were recorded at 100 mV s^{-1} between −0.3 V and 0.8 V vs. Cu^{+}/Cu. An aliquot sample was taken and analyzed by ^{1}H NMR to confirm the conversion rate of the reaction.

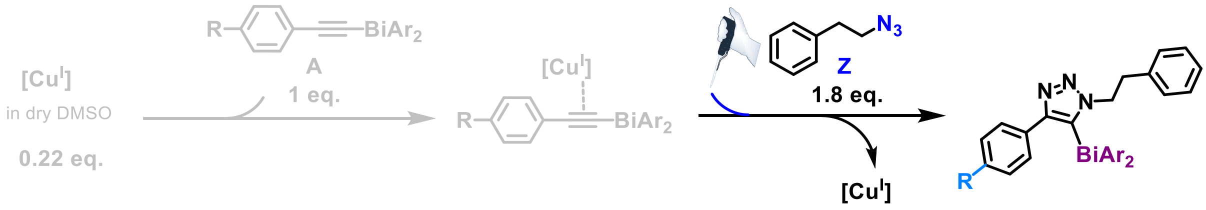

Table S2. Reactions conditions of experiments following general procedure B.

Entry	A[X], mM	AX, µmol	[Z], mM	Z, µmol	[cat], mM	cat, µmol	[ex], mM	ex, µmol	T,˚C
**1**	[1], 5.0	[1], 37.5	9.0	67.5					
**2**	[2], 5.0	[2], 37.5	9.0	67.5					
**3**	[3], 5.0	[3], 37.5	9.0	67.5	1.08	8.12	4.0	30.0	28.0
**4**	[4], 5.0	[4], 37.5	9.0	67.5					
**5**	[5], 5.0	[5], 37.5	9.0	67.5					
**6**	[6], 5.0	[6], 37.5	9.0	67.5					

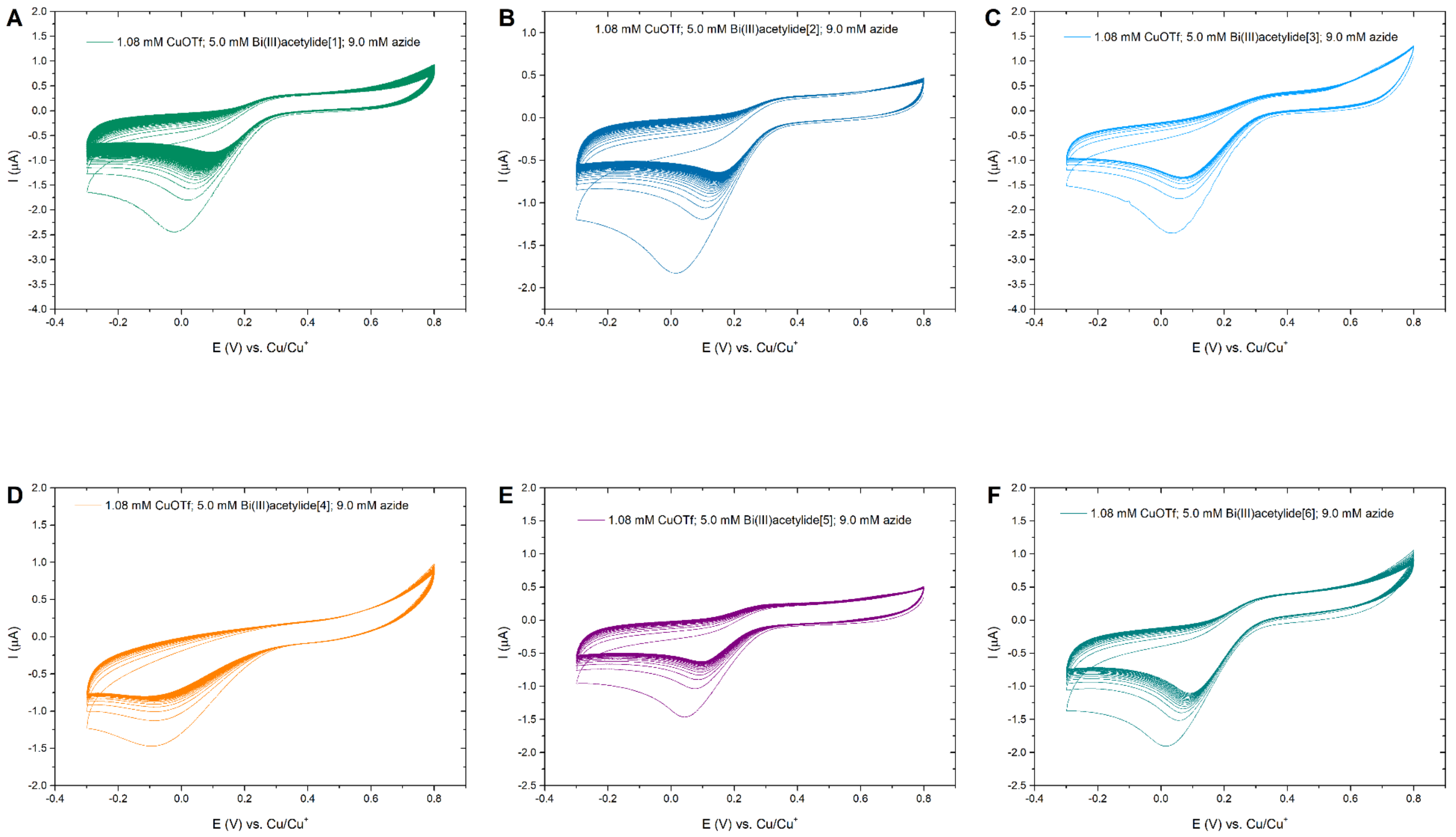

Figure S2. Kinetic profiles A-F of the cyclic voltammograms were obtained by following general procedure B.

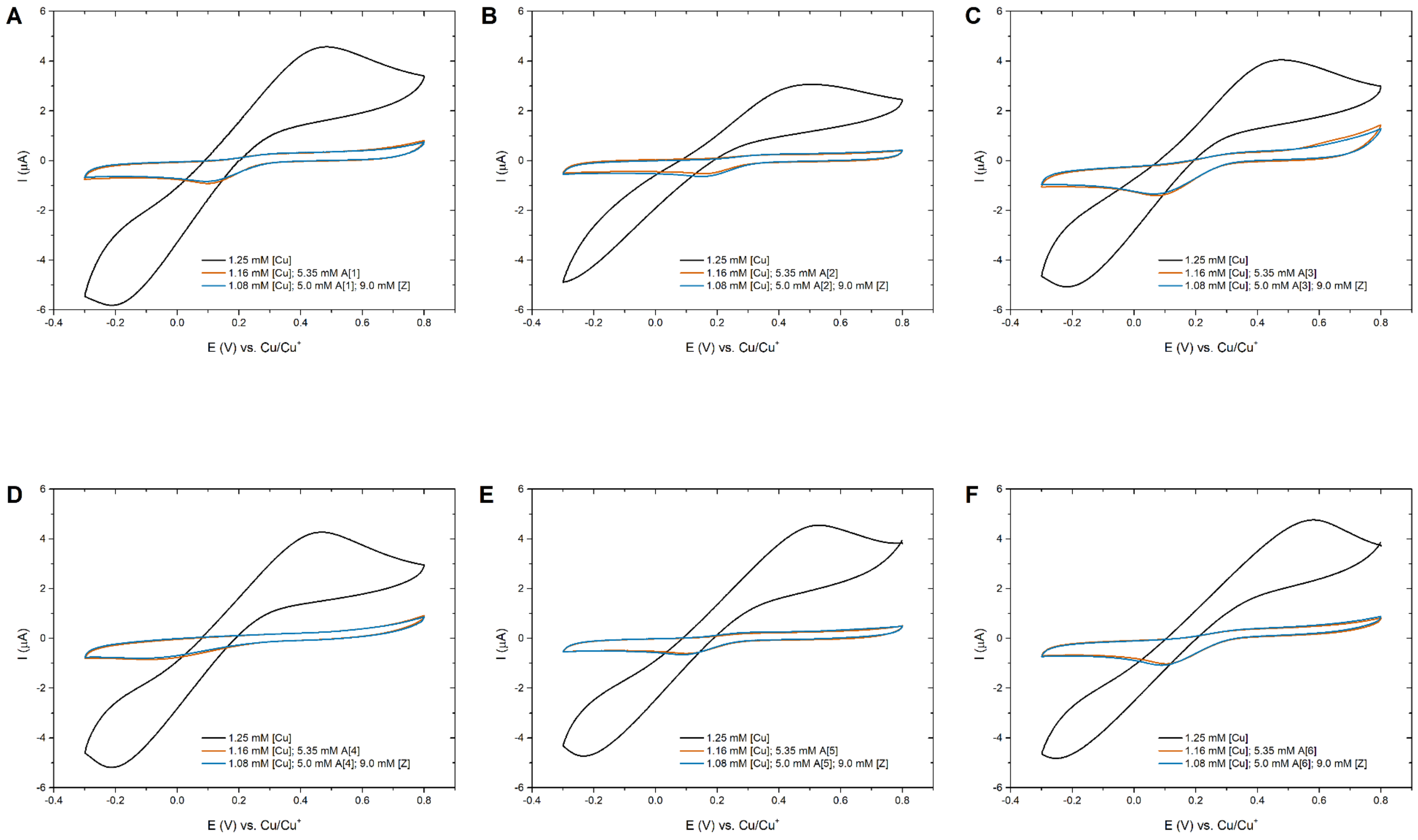

Figure S3. Final cyclic voltammograms A-F were obtained after the kinetic studies by following general procedures A and B.

1.5.1 Determination of reaction rate parameters

The development and validation of the theoretical kinetic model of the catalytic reaction were achieved using self-developed program (C++).

Rate of [1]

Kinetic data was obtained following general procedures A and B using 10-((4-methoxyphenyl)ethynyl)-10H-dibenzo[b,e][1,4]thiabismine 5,5-dioxide (bismuth(III) acetylide[1]). The equilibrium rate constant of the [A·cat] complex formation was calculated as a quotient of the forward (k_A) and reverse (k_{-A}) rate constants. Following Eq.S4 and Eq.6: k_A = 2.39 (M^{-1} sec^{-1}), k_{-A} = 2.81E-3(sec^{-1}), $[cat]_{eq.}$ = 24.32E-5(M).

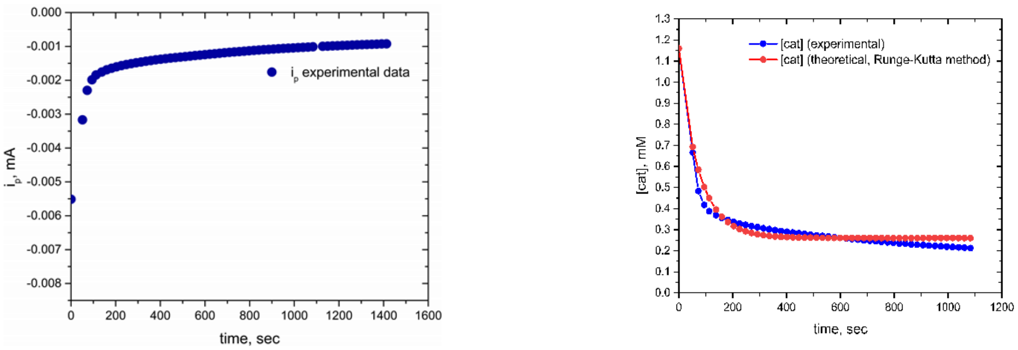

Figure S3a. Concentration and current changes of the Cu^+/Co^0 redox pair for the General Procedure A. Table S1, entry 1.

Rate of [2]

Kinetic data was obtained following general procedures A and B using 10-((4-(tert-butyl)phenyl)ethynyl)-10H-dibenzo[b,e][1,4]thiabismine 5,5-dioxide (bismuth(III) acetylide[2]). The equilibrium rate constant of the [A·cat] complex formation was calculated as a quotient of the forward (k_A) and reverse (k_{-A}) rate constants. Following Eq.S4 and Eq.6: k_A = 1.95 (M^{-1} sec^{-1}), k_{-A} =1.39E-3(sec^{-1}), $[cat]_{eq.}$ = 16.32E-5(M).

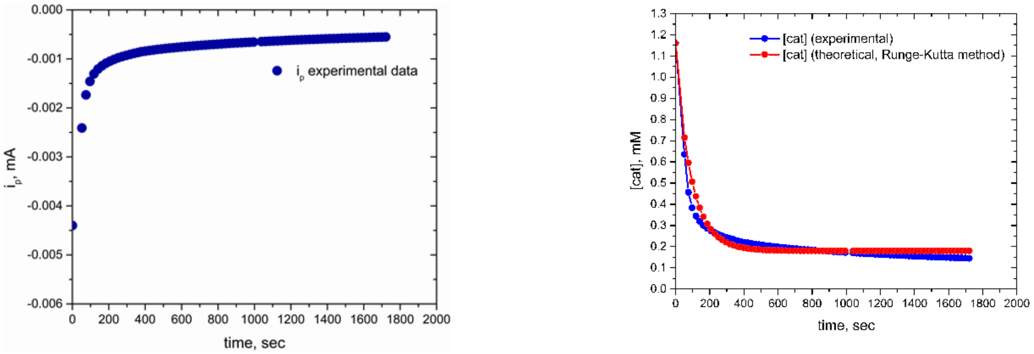

Figure S3b. Concentration and current changes of the Cu^{+}/Co^{0} redox pair for the General Procedure A. Table S1, entry 2.

Rate of [3]

Kinetic data was obtained following general procedures A and B using 10-(p-tolylethynyl)-10H-dibenzo[b,e][1,4]thiabismine 5,5-dioxide (bismuth(III) acetylide[3]). The equilibrium rate constant of the [A·cat] complex formation was calculated as a quotient of the forward (k_A) and reverse (k_{-A}) rate constants. Following Eq.S4, from Eq. 6: $k_A = 2.80$ (M^{-1} sec^{-1}), k_{-A} =3.97E-3(sec^{-1}), $[cat]_{eq.}$ = 27.97E-5(M).

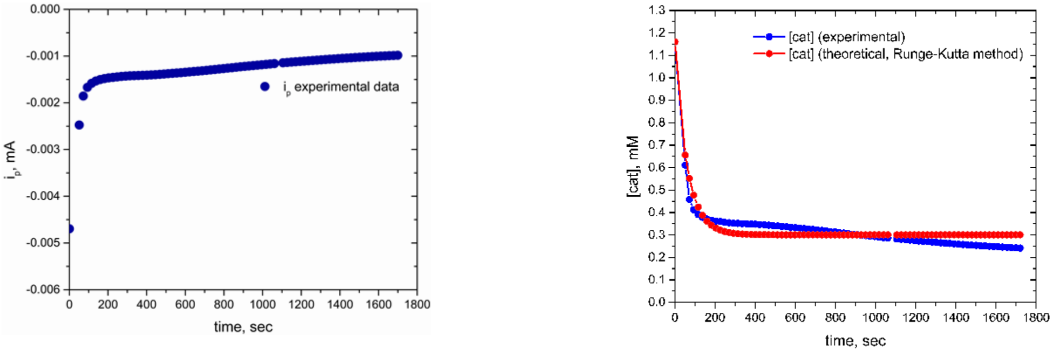

Figure S3c. Concentration and current changes of the Cu^{+}/Co^{0} redox pair for the General Procedure A. Table S1, entry 3.

Rate of [4]

Kinetic data was obtained following general procedures A and B using 10-(phenylethynyl)-10H-dibenzo[b,e][1,4]thiabismine 5,5-dioxide (bismuth(III) acetylide[4]). The equilibrium rate constant of the [A·cat] complex formation was calculated as a quotient of the forward (k_A) and reverse (k_{-A}) rate constants. Following Eq.S4, from Eq.6: k_A = 3.44 (M^{-1} sec^{-1}), k_{-A} =3.73E-3(sec^{-1}), $[cat]_{eq.}$ = 22.88E-5(M).

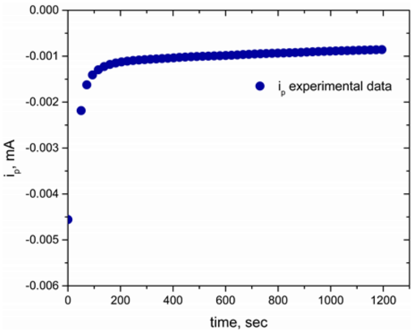

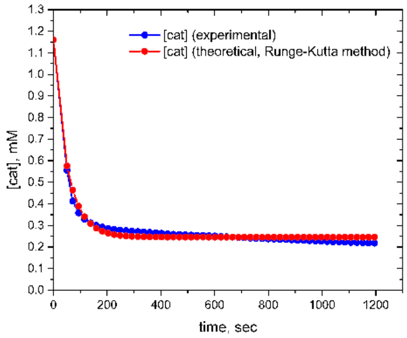

Figure S3d. Concentration and current changes of the Cu^+/Co^0 redox pair for the General Procedure A. Table S1, entry 4.

Rate of [5]

Kinetic data was obtained following general procedures A and B using 10-((4-bromophenyl)ethynyl)-10H-dibenzo[b,e][1,4]thiabismine 5,5-dioxide (bismuth(III) acetylide[5]). The equilibrium rate constant of the [A·cat] complex formation was calculated as a quotient of the forward (k_A) and reverse (k_{-A}) rate constants. Following Eq.S4, from Eq. 6: k_A = 4.97 (M^{-1} sec^{-1}), k_{-A} =3.65E-3(sec^{-1}), $[cat]_{eq.}$ = 16.75E-5(M).

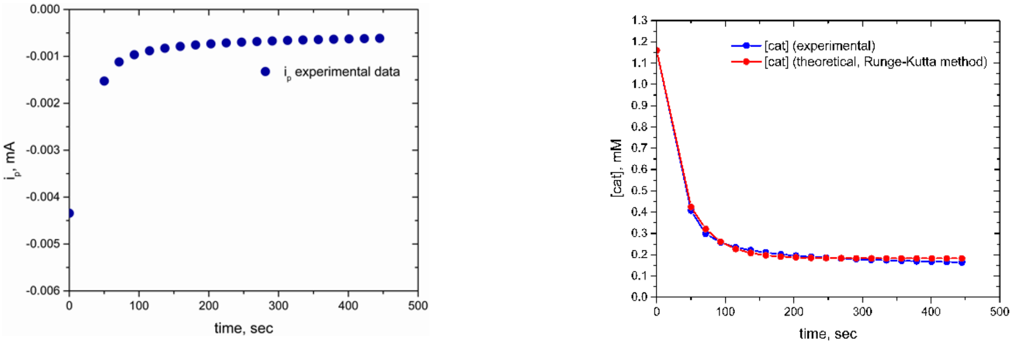

Figure S3e. Concentration and current changes of the Cu^{+}/Co^{0} redox pair for the General Procedure A. Table S1, entry 5.

Rate of [6]

Kinetic data was obtained following general procedures A and B using 10-((4-bromophenyl)ethynyl)-10H-dibenzo[b,e][1,4]thiabismine 5,5-dioxide (bismuth(III) acetylide[6]). The equilibrium rate constant of the [A·cat] complex formation was calculated as a quotient of the forward (k_A) and reverse (k_{-A}) rate constants. Following Eq.S4, from Eq.6: k_A = 4.36 (M^{-1} sec^{-1}), k_{-A} =6.15E-3(sec^{-1}), $[cat]_{eq.}$ = 27.83E-4(M).

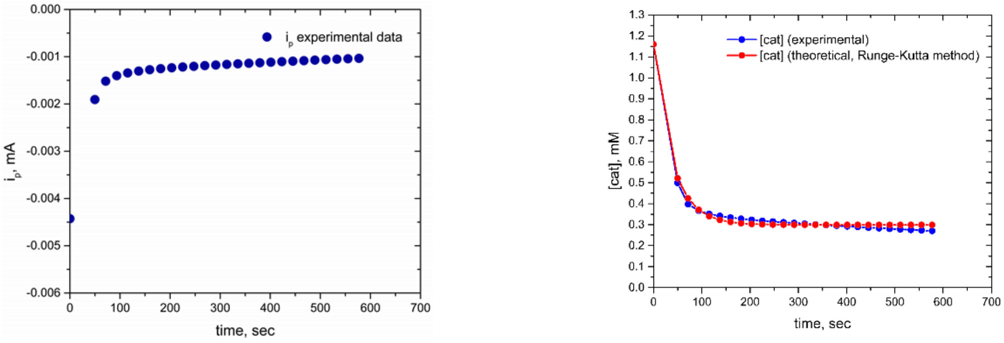

Figure S3f. Concentration and current changes of the Cu^{+}/Co^{0} redox pair for the General Procedure A. Table S1, entry 6.

1.5.2 Derivatization of the catalyst concentration at the equilibrium ([cat]eq.)

When reaching equilibrium conditions (General Procedure A):

$$\frac{d[cat]}{dt} = 0 \tag{Eq.S1}$$

From Eq. (2) at the equilibrium conditions:

$$\frac{d[cat]}{dt} = 0 = -k_A([A]_0 - [cat]_0 + [cat])[\mathrm{cat}] + k_{-A}([cat]_0 - [cat]) \tag{Eq.S2}$$

The equilibrium concentration $[\mathrm{cat}]_{\mathrm{eq.}}$ can be determined as a solution to the following quadratic expression:

$$k_A[cat]^2_{eq.} + (k_{-A} + k_A([A]_0 - [cat]_0))[cat]_{eq.} - k_{-A}[cat]_0 = 0 \tag{Eq.S3}$$

The positive value solution for the equilibrium concentration $[\mathrm{cat}]_{\mathrm{eq.}}$ is defined as:

$$[cat]_{eq.} = -{}^1/_2\left({}^{k_{-A}}/_{k_A} + [A]_0 - [cat]_0\right) + ({}^1/_4\left({}^{k_{-A}}/_{k_A} + [A]_0 - [cat]_0\right)^2 + {}^{k_{-A}}/_{k_A}[cat]_0)^{1/2} \tag{Eq.S4}$$

1.5.3 Standart deviations

The standard deviations were calculated for each sample taking into account a 0.01 mL volume error when dissolving and the $5\cdot10^{-5}$ g accuracy of the balance weighing.

Table S3a. Standard deviations for initial concentrations $[\mathrm{cat}]_o$ and $[\mathrm{A}]_o$.

Entry	$[\mathrm{cat}]_o\cdot10^{-3}$,M	$\sigma([\mathrm{cat}]_o)\cdot10^{-5}$, M	$[\mathrm{A}]_o\cdot10^{-3}$,M	$\sigma([\mathrm{A}]_o)\cdot10^{-5}$,·M
[1], R = OMe	1.16	1.80	5.35	5.50
[2], R = ^{t}Bu				5.48
[3], R = Me				5.51
[4], R = H				5.52
[5], R = Br				5.47
[6], R = CF_3				5.48

The estimation of the standard deviations for the rate parameters k_A, k_A, K_A, and $[cat]_{eq.}$ derived from electrochemical experimental data was done following the reported procedure by Alper et al..[1] Thus, the repetitive model simulations have been accomplished using Monte Carlo generated noise consisting of normal-distributed values with zero mean and appropriate variance determined by standard deviations for $[cat]_0$, $[A]_0$. The Box-Muller method was applied to obtain Gaussian-distributed values.[2]

Table S3b. Best fit parameter values k_A, k_A, K_A, and $[cat]_{eq.}$ and their standart deviations σ(k_A), σ(k_{-A}),·σ(K_A) and σ($[cat]_{eq.}$). The confidence intervals should be taken as 2×σ to satisfy the 95% level of confidence.

Entry	k_A, M^{-1} sec^{-1}	σ(k_A), M^{-1} sec^{-1}	$k_{-A}\cdot10^{-3}$, sec^{-1}	σ(k_{-A})$\cdot10^{-3}$, sec^{-1}	$K_A\cdot10^{3}$, M^{-1}	σ(K_A)$\cdot10^{3}$, M^{-1}	$[cat]_{eq}\cdot10^{-5}$, M	σ($[cat]_{eq.}$)$\cdot10^{-5}$, M
[1], R = OMe	2.39	0.0598	2.81	0.009	0.85	0.0215	24.32	0.033
[2], R = ^{t}Bu	1.95	0.0415	1.39	0.007	1.40	0.0344	16.32	0.020
[3], R = Me	2.80	0.0754	3.97	0.012	0.70	0.0189	27.97	0.016
[4], R = H	3.44	0.0749	3.73	0.019	0.92	0.0240	22.88	0.015
[5], R = Br	4.97	0.0923	3.65	0.025	1.36	0.0337	16.75	0.016
[6], R = CF_3	4.36	0.0965	6.15	0.031	0.71	0.0190	27.83	0.013

2. ^{1}H NMR kinetic reactivity studies

2.1 General information

DMSO-d6 was purchased in ampules from Cambridge Isotope Laboratories, Inc., opened inside a glovebox, and stored over molecular sieves. Copper(I) trifluoromethanesulfonate toluene complex (≥99.7%) was purchased from Sigma-Aldrich. 1,4-dimethoxybenzene and 1,2,3,4,5,6-hexamethyl benzene were obtained from commercial sources (Sigma-Aldrich).

2.2 Experimental details

Kinetic experiments were carried out in 5mm thin wall precision NMR tubes (7”, 600 MHz, 535-PP-7, Wilmad LabGlass). All glassware was oven-dried (140 C) and purged by three vacuum-N_2 cycles in the antechamber of the glovebox before use. Stock solutions of corresponding bismuth(III) acetylides, (2-azidoethyl)benzene, and copper(I) trifluoromethanesulfonate toluene complex were prepared using 1 mL volumetric flasks volume and stored in LCMS-capped vials for the duration of one batch of experiments. ^{1}H NMR spectra were recorded on a Varian VNMRS-600 spectrometer. Chemical shifts are reported in ppm referenced to the residual peak of DMSO-d5 at 2.5 ppm.

2.3 Data analysis

For manipulation and analysis of the NMR data and signal peak integration, MestReNova (Version 9.0.0, Mestrelab Research S.L.) was used. The substrates rate orders from the excess experiments were derived in the frames of the first kinetic regime (initial rates).

2.4 General procedure C – Independent bismuth(III)-acetylide reactivity experiments

For independent reactivity experiments: Stock solutions for the corresponding bismuth(III) acetylides (0.100 M), (2-azidoethyl)benzene (0.688 M), 1,4-dimethoxybenzene (internal standard) (54 mM) and copper(I) triflate (20 mM) were prepared in DMSO-d6. Of these stock solutions: 200 μL of the alkyne, 50 μL of the azide, and 50 μL of the reference stock solutions were transferred to an NMR tube and diluted with 450 μL of DMSO-d6 capped with a gas-tight rubber NMR septum and agitated. Lastly, 50 μL of the copper(I) trifluoromethanesulfonate toluene complex catalyst solution was added to the NMR tube and immediately agitated. The total volume in the NMR tube for one experiment was 800 μL.

Kinetic NMR measurements were recorded on a Varian VNMRS-600 spectrometer. The sample spinning rate was 20 Hz. ^{1}H-NMR data were collected with the following acquisition parameters: 1 transient (scans), 5 sec. relaxation delay time, acquisition time of 5.824 sec. Between the spectra was a 1 sec pre-acquisition delay. Data was collected at 60.0°C. The temperature was calibrated against a temperature standard (ethylene glycol). Each experiment was performed twice. All reagents were dried before use. All manipulations were performed in the dry nitrogen atmosphere of a glove box. Reaction conversions were integrated relative to an internal reference standard.

Table S4. Comparison of acetylene group C(1)≡C(2) IR stretching vibration frequencies of the derivatized bismuth(III) acetylides.

**Bismuth(III)-acetylide**	**IR* (υ[cm^{-1}])**	**Bismuth(III)-acetylide**	**IR* (υ[cm^{-1}])**
**para-phenyl substituted bismuth(III)acetylides**			
[1], R = OMe	2099	[2], R = ^{t}Bu	2108
[3], R = Me	2099	[4], R = H	2111
[5], R = Br	2105	[6], R = CF_3	2114
**diphenylsulfone substituted bismuth(III)acetylides**			
[7], R' = CF_3	2045	[8], R' = Cl	2098
[9], R' = OMe	2107	[10], R' = OMe, OMe	2051

* FT-IR spectra were taken in the mid-infrared (MIR) range of 400–4000 cm^{-1} using attenuated total reflectance (ATR).

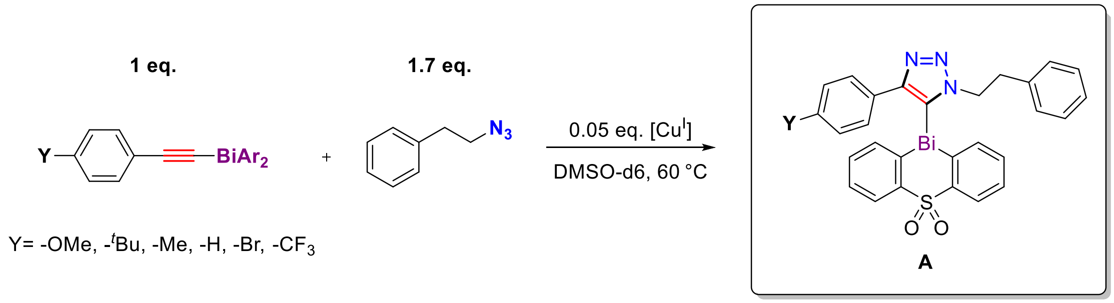

Table S5 Reactions conditions of experiments following general procedure C. Independent bismuth(III)-acetylide reactivity experiment.

Entry	A [X], mM	[Z], mM	[cat], mM	[ref], mM	[ex],mM	Time (avg), sec	T, ˚C	Conv, %
**1**	[1], 25	43				14400		100.0
**2**	[2], 25	43				9300		100.0
**3**	[3], 25	43	1.25	3.375	18	1350	60.0	100.0
**4**	[4], 25	43				2100		100.0
**5**	[5], 25	43				22200		94.6
**6**	[6], 25	43				1510		100.0

2.4.1 Determination of reaction rate parameters

Rate of [1]

Kinetic data was obtained by following general procedure C; using 10-((4-methoxyphenyl)ethynyl)-10H-dibenzo[b,e][1,4]thiabismine 5,5-dioxide (bismuth(III) acetylide[1]). The rate constant k = 1.19E-5(0.70E-7) was derived as the slope of the initial rate curve.

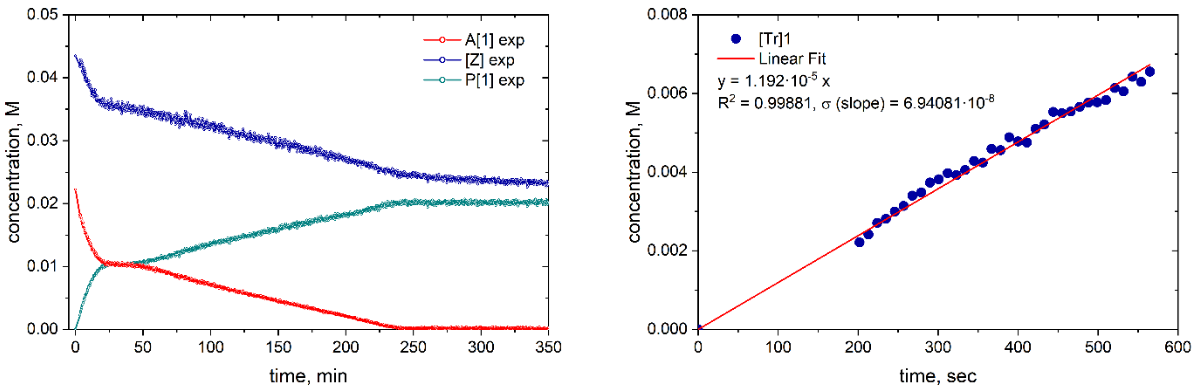

Figure S4a. Concentration in substrates [A] and [Z] and the product [P] for the General Procedure C. Table S4, entry 1.

Rate of [2]

Kinetic data was obtained by following general procedure C; using 10-((4-(*tert*-butyl)phenyl)ethynyl)-10H-dibenzo[b,e][1,4]thiabismine 5,5-dioxide (bismuth(III) acetylide[2]). The rate constant k=0.81E-5 (0.63E-7) was derived as the slope of the initial rate curve.

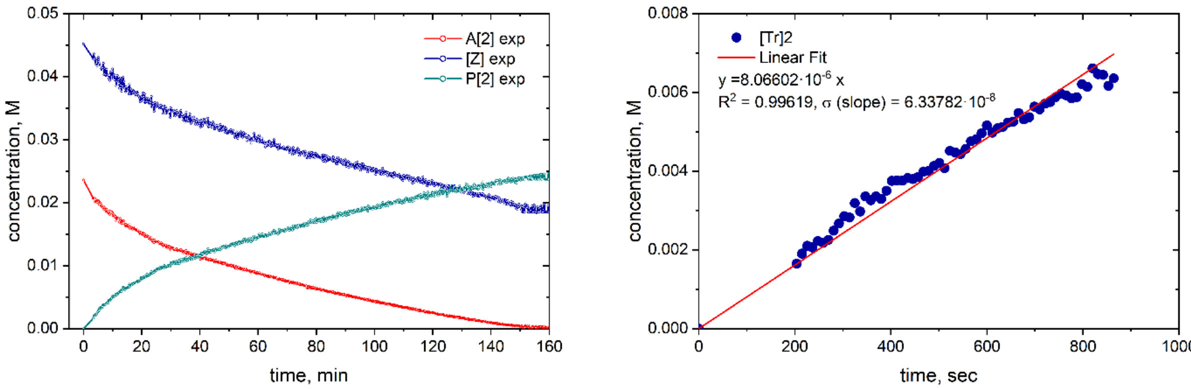

Figure S4b. Concentration in substrates [A] and [Z] and the product [P] for the General Procedure C. Table S4, entry 2.

Rate of [3]

Kinetic data was obtained by following general procedure C; using 10-(*p*-tolylethynyl)-10H-dibenzo[b,e][1,4]thiabismine 5,5-dioxide (bismuth(III) acetylide[3]). The rate constant k=2.31E-5 (1.53E-7) was derived as the slope of the initial rate curve.

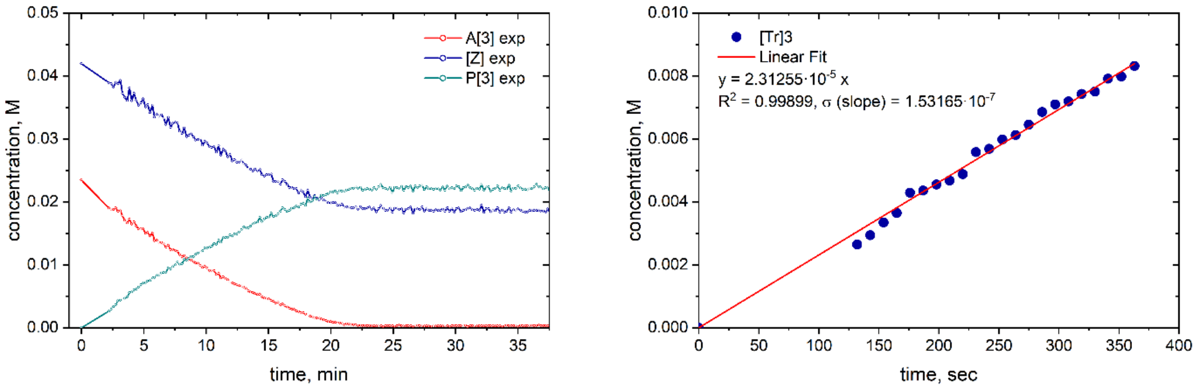

Figure S4c. Concentration in substrates [A] and [Z] and the product [P] for the General Procedure C. Table 4, entry 3.

Rate of [4]

Kinetic data was obtained by following general procedure C; using 10-(phenylethynyl)-10H-dibenzo[b,e][1,4]thiabismine 5,5-dioxide (bismuth(III) acetylide[4]). The rate constant k=2.24E-5 (1.47E-7) was derived as the slope of the initial rate curve.

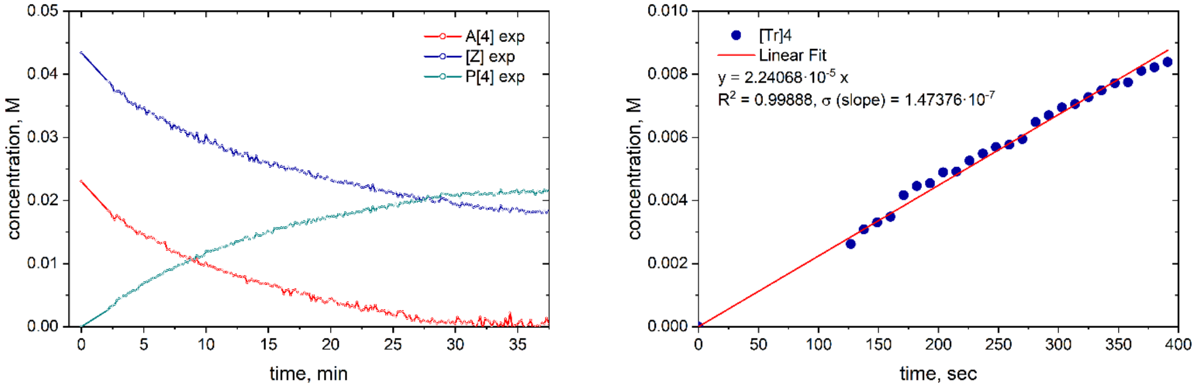

Figure S4d. Concentration in substrates [A] and [Z] and the product [P] for the General Procedure C. Table S4, entry 4.

Rate of [5]

Kinetic data was obtained by following general procedure C; using 10-((4-bromophenyl)ethynyl)-10H-dibenzo[b,e][1,4]thiabismine 5,5-dioxide (bismuth(III) acetylide[5]). The rate constant k=0.34E-5 (0.17E-7) was derived as the slope of the initial rate curve.

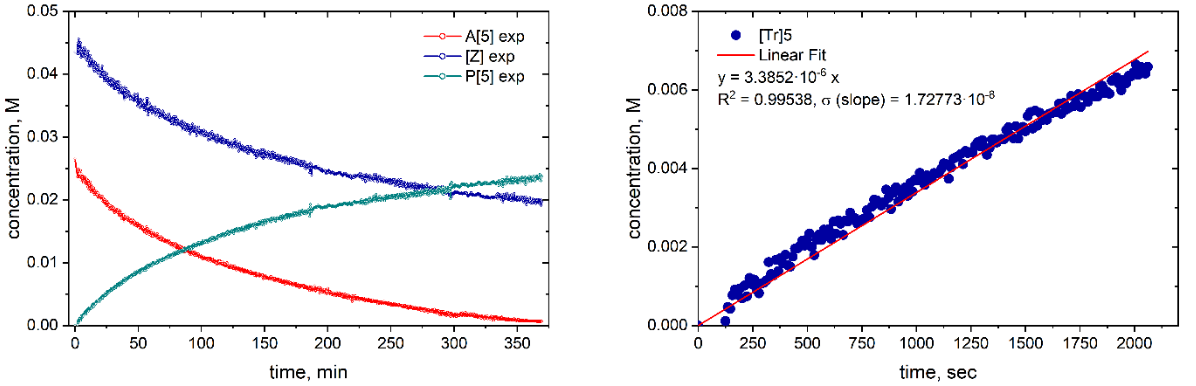

Figure S4e. Concentration in substrates [A] and [Z] and the product [P] for the General Procedure C. Table S4, entry 5.

Rate of [6]

Kinetic data was obtained by following general procedure C; using 10-((4-bromophenyl)ethynyl)-10H-dibenzo[b,e][1,4]thiabismine 5,5-dioxide (bismuth(III) acetylide[6]). The rate constant k=1.43E-5 (0.70E-7) was derived as the slope of the initial rate curve.

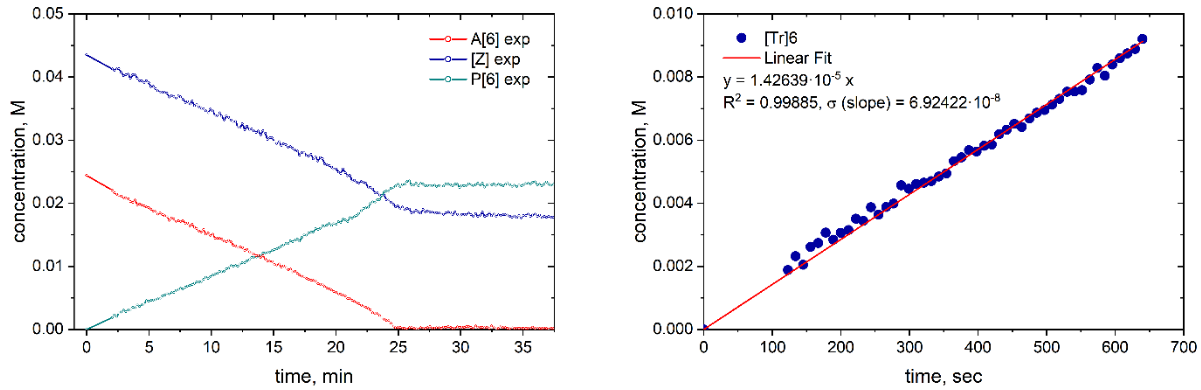

Figure S4f. Concentration in substrates [A] and [Z] and the product [P] for the General Procedure C. Table S4, entry 6.

2.5 General procedure D - Rate orders experiments

<u>Evaluation of 1-bismuth(III) acetylide component rate order</u>: Stock solutions of (2-azidoethyl)benzene (0.6 M), of bismuth(III) acetylide[2], 1,2,3,4,5,6-hexamethylbenzene (0.230 M, as internal standard) and copper(I) triflate (0.300 M) were prepared in DMSO-d6. Of these stock solutions: *x* μL of the bismuth(III) acetylide, 200 μL of azide, 50 μL of reference stock solution were solutions were transferred to an NMR tube and diluted with (150+180+200-*x*) μL of DMSO-d6 agitated and closed with a gas-tight rubber NMR septum. Lastly, 20 μL of the copper(I) trifluoromethanesulfonate toluene complex catalyst solution was added to the NMR tube and immediately agitated. The total volume in the NMR tube for one experiment was 800 μL.

Kinetic NMR measurements were recorded on a Varian VNMRS-600 spectrometer. The sample spinning rate was 20 Hz. ^{1}H-NMR data were collected with the following acquisition parameters: 3 transients (scans), 5 sec. relaxation delay time, acquisition time of 2.7263 sec. Between the spectra was a 1 sec pre-acquisition delay. Data was collected at 69.0˚C. The temperature was calibrated against a temperature standard (ethylene glycol). Each experiment was performed twice. All reagents were dried before use. All manipulations were performed in the dry nitrogen

atmosphere of a glove box. Reaction conversions were integrated relative to an internal reference standard.

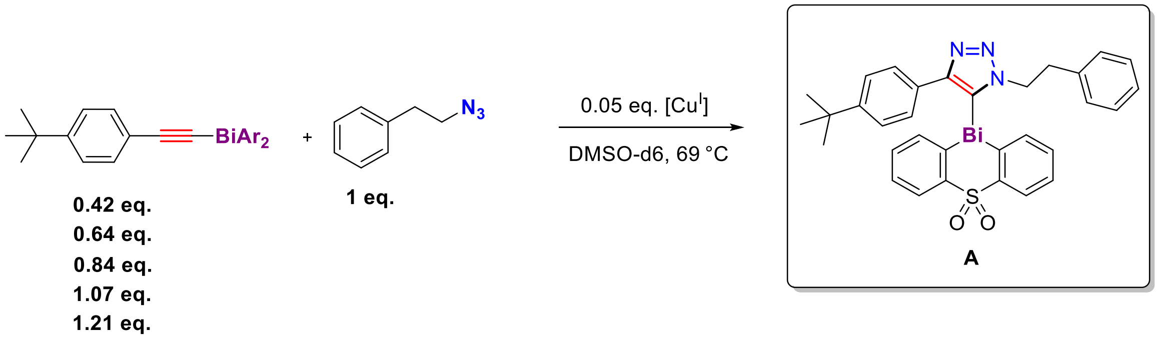

Table S6. Reactions conditions of experiments following general procedure D. Variable [Alkyne] concentration experiment. Kinetic profiles are shown in Figure S5.

Entry	A[2], M	[Z], M	[cat], mM	[excess], M	Time (avg), sec	Conv, %
**1**	0.063	0.150		0.087	3300	100.0
**2**	0.096	0.150		0.054	6400	100.0
**3**	0.126	0.150	7.5	0.024	16000	100.0
**4**	0.160	0.150		-0.10	30000	100.0
**5**	0.181	0.150		-0.31	45000	100.0

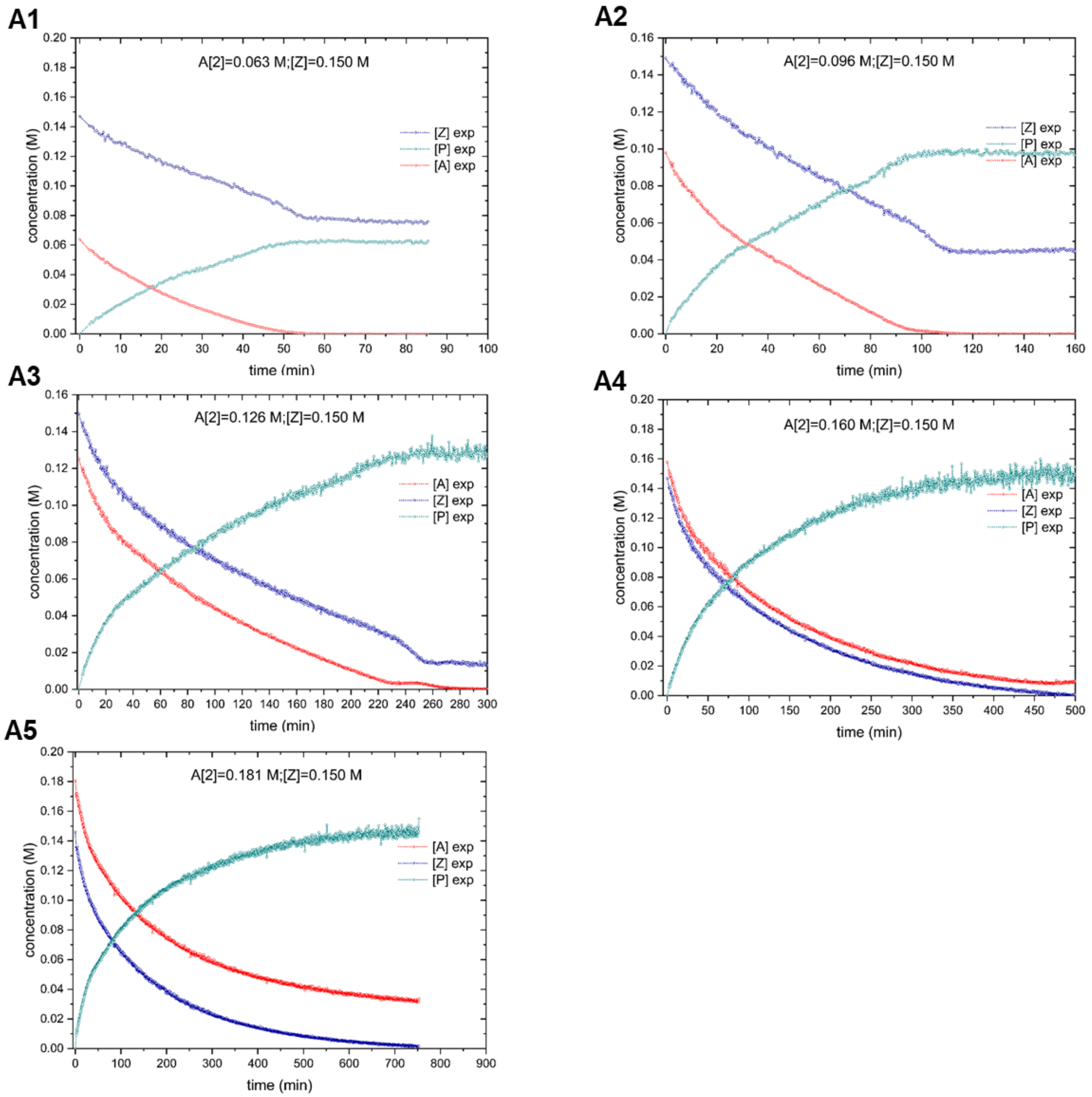

Figure S5. Kinetic profiles of reactions studied following general procedure D.

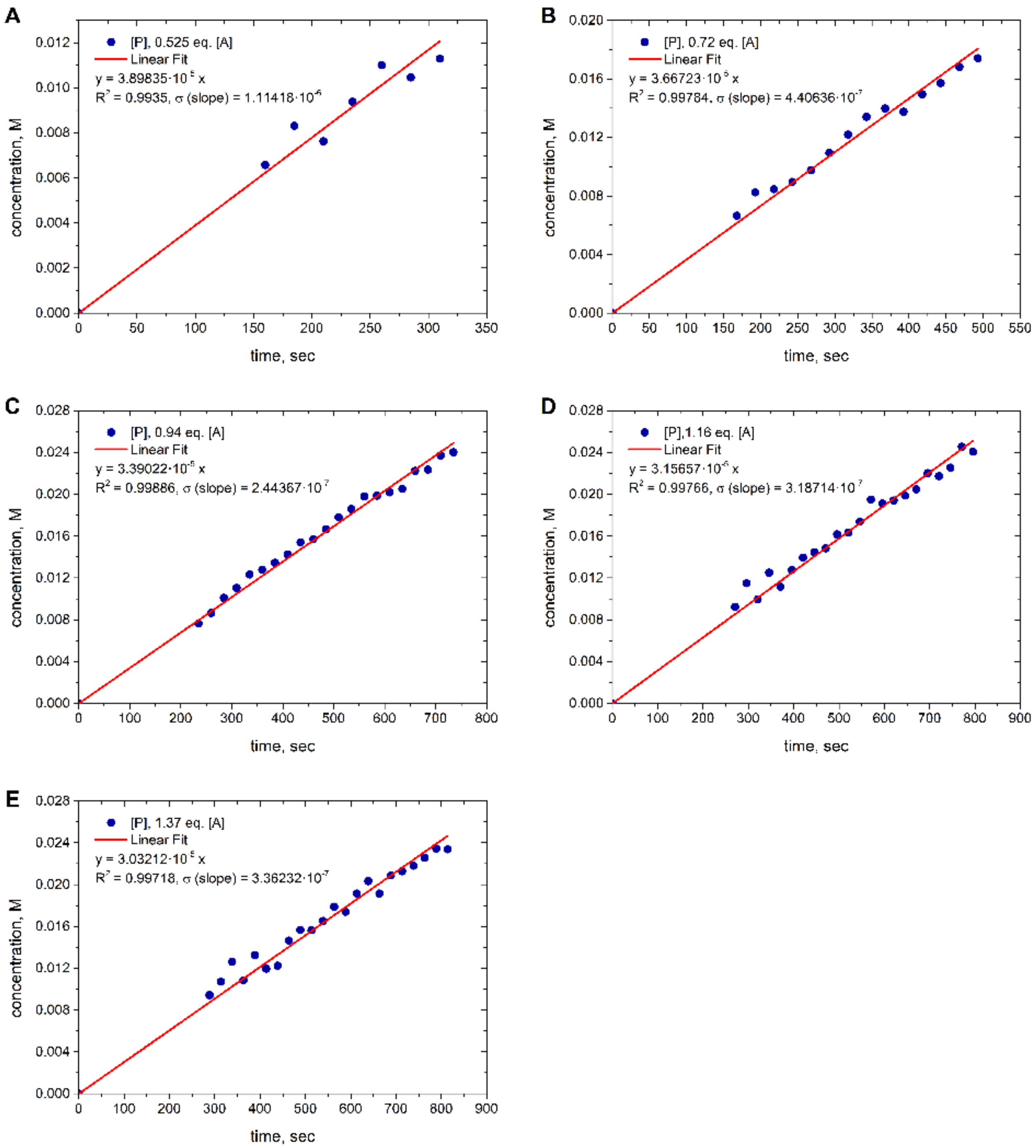

Figure S5a. Substrate rate order kinetic study performed following general procedure D. Method of initial rates. (A-E) Product P[2] concentration vs. time.

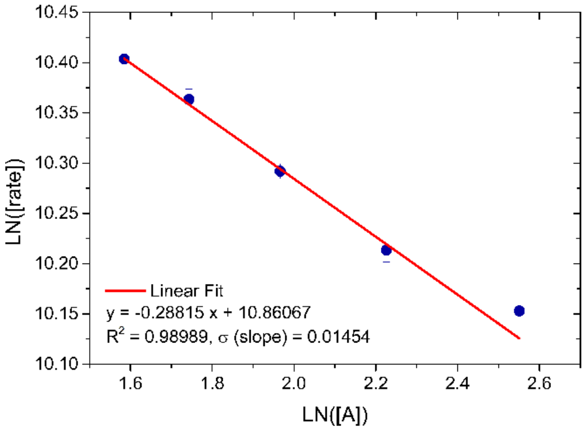

Figure S5b. Substrate rate order kinetic study performed following general procedure D. Method of initial rates. LN(rate) vs. LN([A]).

2.6 Dynamic NMR spectroscopy

The experiment was performed by recording ^{1}H-NMR of one identical sample at different temperatures. No quantitative interpretation of the changes in the spectra was performed. Bismuth(III) triazolide [2] in DMF-d7 was chosen as a representative sample. A restricted rotation around the C(sp^2)-Bi bond was confirmed by the variability of resonances for slow, intermediate, and fast exchange regions. At low temperatures all aromatic protons of the diphenyl sulfone unit exhibit different unique chemical shifts. As the temperature gradually raises, a similar observation was made for the two methylene groups of the ‘azido’ fragment. Close to room temperature these signals are broad and overlapping. The existence of two structural isomers is supported by the fact of two characteristic singlets representing the ^{t}Bu substituent.

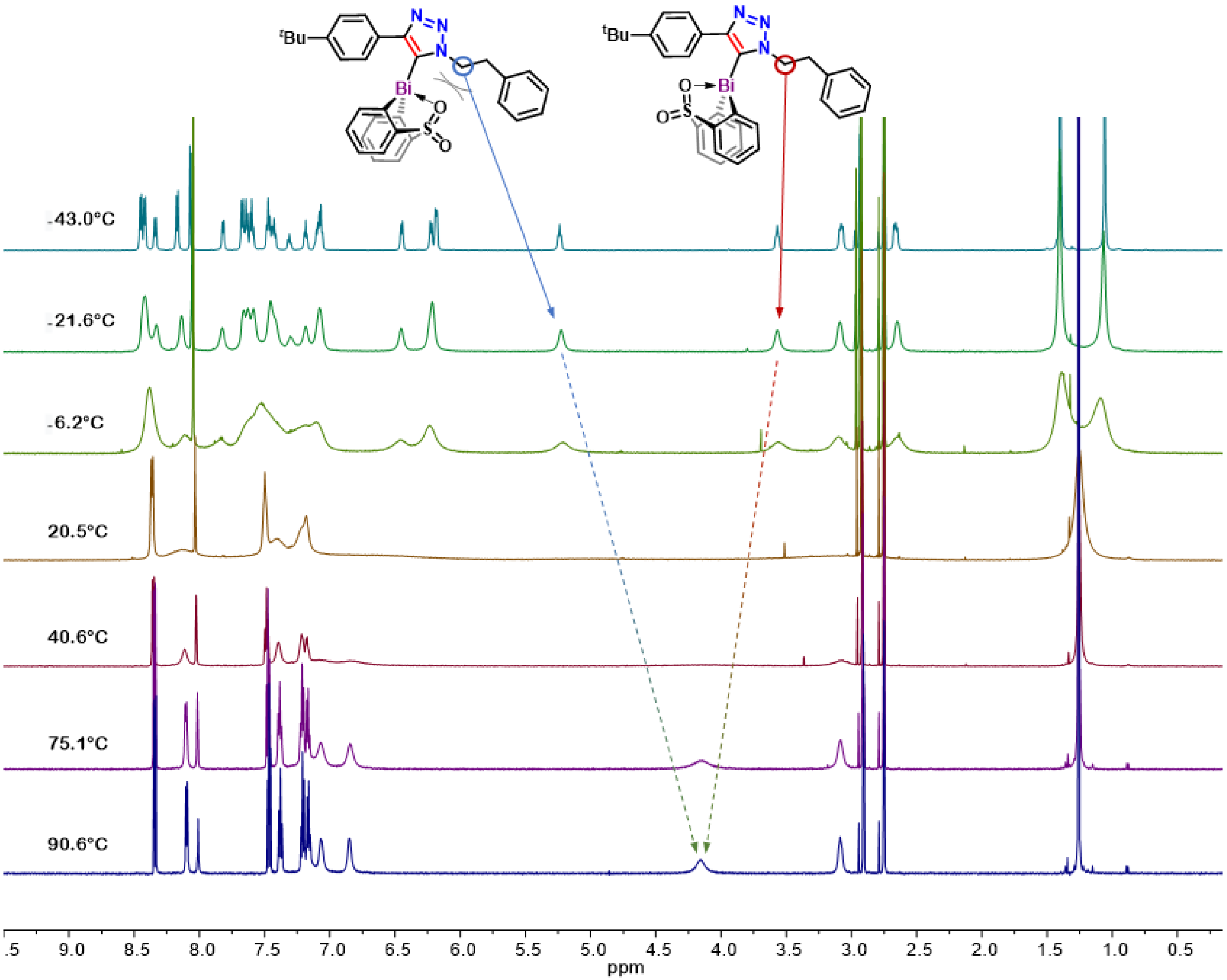

Figure S6. ^{1}H NMR signal coalescence/decoalescence demonstrate intermolecular processes of bond rotations as a function of temperature for 5-bismuth(III) triazole[2].

2.7 General Procedure E - Protoalkyne/ bismuth(III) acetylide competitive experiments

Evaluation of catalytic activity in the presence of terminal acetylenes: Stock solutions of the corresponding bismuth(III) acetylides (60 mM), terminal alkyne (60 mM), (2-azidoethyl)benzene (60 mM), 1,4-dimethoxybenzene (12 mM, internal standard) and copper(I) triflate (5.0 mM) were prepared in DMSO-d6. Of these stock solutions: 200 μL of bismuth(III)-acetylide, 200 μL of terminal alkyne, and 50 μL of reference the stock solutions were transferred into an NMR tube, diluted with 300 μL of DMSO-d6 agitated and closed with a gas-tight rubber NMR septum. Lastly, 50 μL of the copper(I) trifluoromethanesulfonate toluene complex catalyst solution was added to the NMR tube and immediately agitated. The total volume in the NMR tube for one experiment was 800 μL.

Kinetic NMR measurements were recorded on a Varian VNMRS-600 spectrometer. The sample spinning rate was 20 Hz. ^{1}H-NMR data were collected with the following acquisition parameters: 1 transient (scan), 5 sec. relaxation delay time, acquisition time of 5.824 sec. Between the spectra was a 1 sec pre-acquisition delay. Data was collected at 27.0˚C. After about 1 hour of data acquisition, the temperature was raised to 69.0˚C. After which 200 μL of the (2-azidoethyl)benzene stock solution were injected and immediately agitated. The total volume inside the NMR tube experiment increased to 1 mL. Temperatures were calibrated against a temperature standard (ethylene glycol). Each experiment was performed twice. All reagents were dried before use. All manipulations were performed in the dry nitrogen atmosphere of a glove box. Reaction conversions were integrated relative to an internal reference standard.

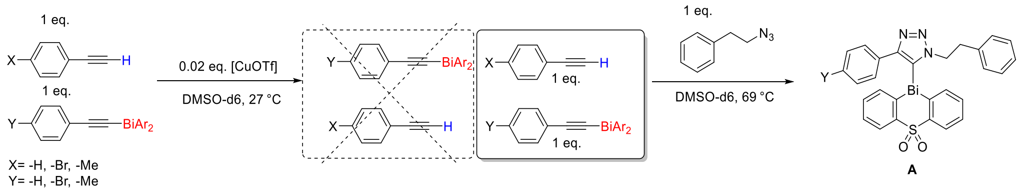

Table S7. Reactions conditions of experiments following general procedure E. Kinetic profiles are shown in **Figure S7**.

Entry	A[X], mM	A[Y],M	[Z], M	[cat], mM	yield A, %
**1**	[-Br], 12	[-H], 12	12	0.250	100.0
**2**	[-H], 12	[-Br], 12			58.33
**3**	[-Me], 12	[-H], 12			100.0
**4**	[-H], 12	[-Me], 12			100.0

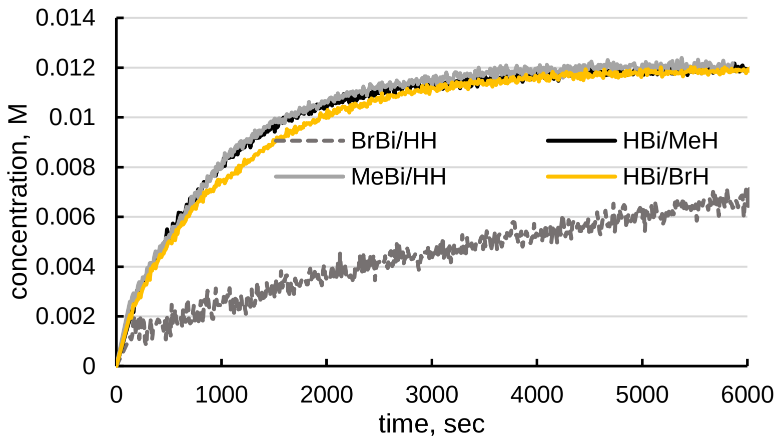

Figure S7. Kinetic profiles of reactions studied following general procedure E.

2.8 Moisture compatibility experiments

Table S8. Moisture compatibility experiment. Reaction conditions of experiments following general procedure C.

№	A [6], mM	[Z], mM	[cat], mM	Water, %	[Ref], mM	Time, sec	T, ˚C	Conv, %
**1**	25	43	1.25	2% H_2O	3.375	1509	60	100.0
**2**				12% H_2O		1685		100.0

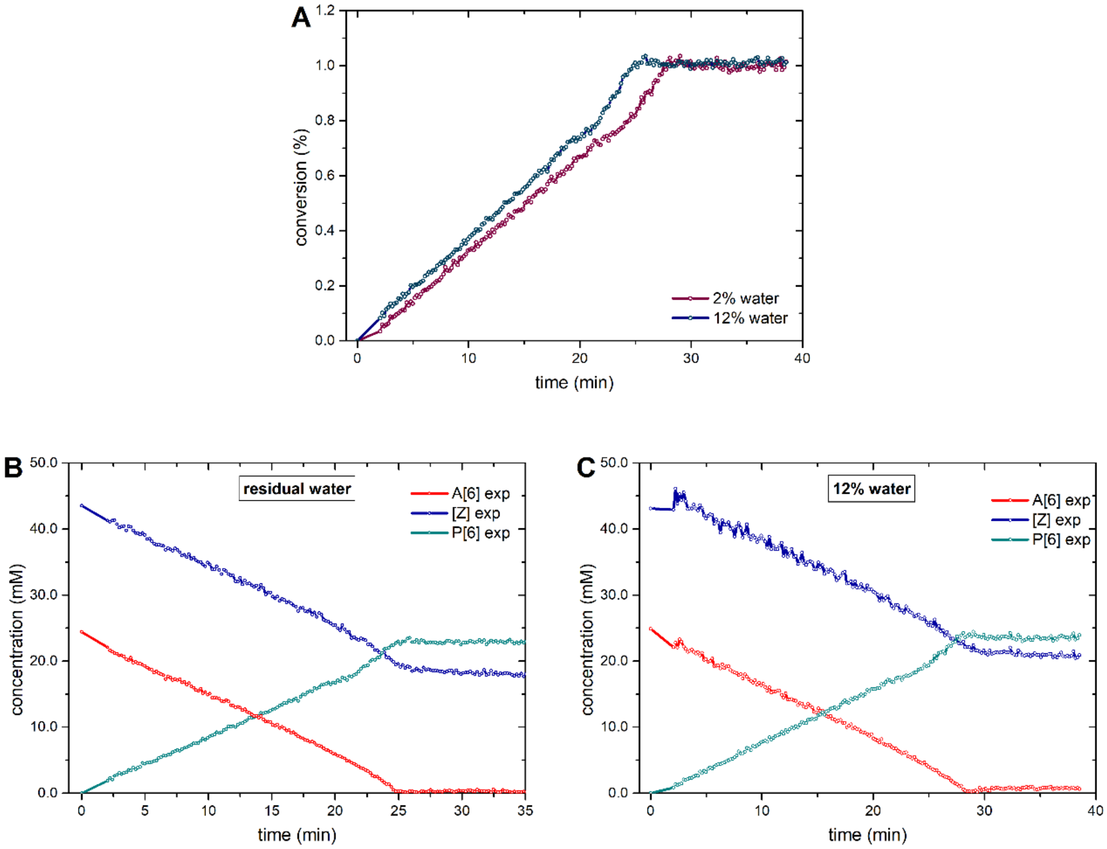

Figure S8. Kinetic profiles of reactions studied following general procedure C. (A) Conversion profiles of reactions (Table S7)**,** entries 1 and 2; (B) dry and (C) ‘wet’ DMSO-d6 as solvent.

2.9 General procedure F – catalyst robustness experiments

Evaluation of catalyst rate order: Stock solutions of bismuth(III)-acetylide[4] (60 mM), of 2-azidoethylbenzene (60 mM), 1,4-dimethoxybenzene (25 mM) and copper(I) triflate (6.2 mM) were prepared in DMSO-d6. Of these stock solutions: 160 μL of bismuth(III)-acetylide, 200 μL of azide, 100 μL of reference stock solution transferred to an NMR tube, diluted with 240 μL (entry 1) or 140 μL (entry 2) of pure DMSO-d6 agitated and closed with a gas-tight rubber NMR septum. Lastly, 100 μL (entry 1) or 200 μL (entry 2) of the copper(I) trifluoromethanesulfonate toluene complex catalyst solution was added to the NMR tube and immediately agitated. The total volume in the NMR tube for one experiment was 800 μL.

Kinetic NMR measurements were recorded on a Varian VNMRS-600 spectrometer. The sample spinning rate was 20 Hz. ^{1}H-NMR data were collected with the following acquisition parameters: 2 transients (scans), 5 sec. relaxation delay time, acquisition time of 5.824 sec. Between the spectra was a 1 sec pre-acquisition delay. Data was collected either collected at 60.0°C. The temperature was calibrated against a temperature standard (ethylene glycol). Each experiment was performed twice. All reagents were dried before use. All manipulations were performed in the dry nitrogen atmosphere of a glove box. Reaction conversions were integrated relative to an internal reference standard.

Table S9. Catalyst robustness experiments. Reactions conditions of the experiments following general procedure F.

Entry	A [4], M	[Z], M	[cat], mM	[Ref], mM	[excess], M	Time (averaged), sec	T,°C	Conv, %
**1**	0.012	0.015	0.775	3.1	0.003	4436	60	100.0

**2**	1.550	2336	100.0

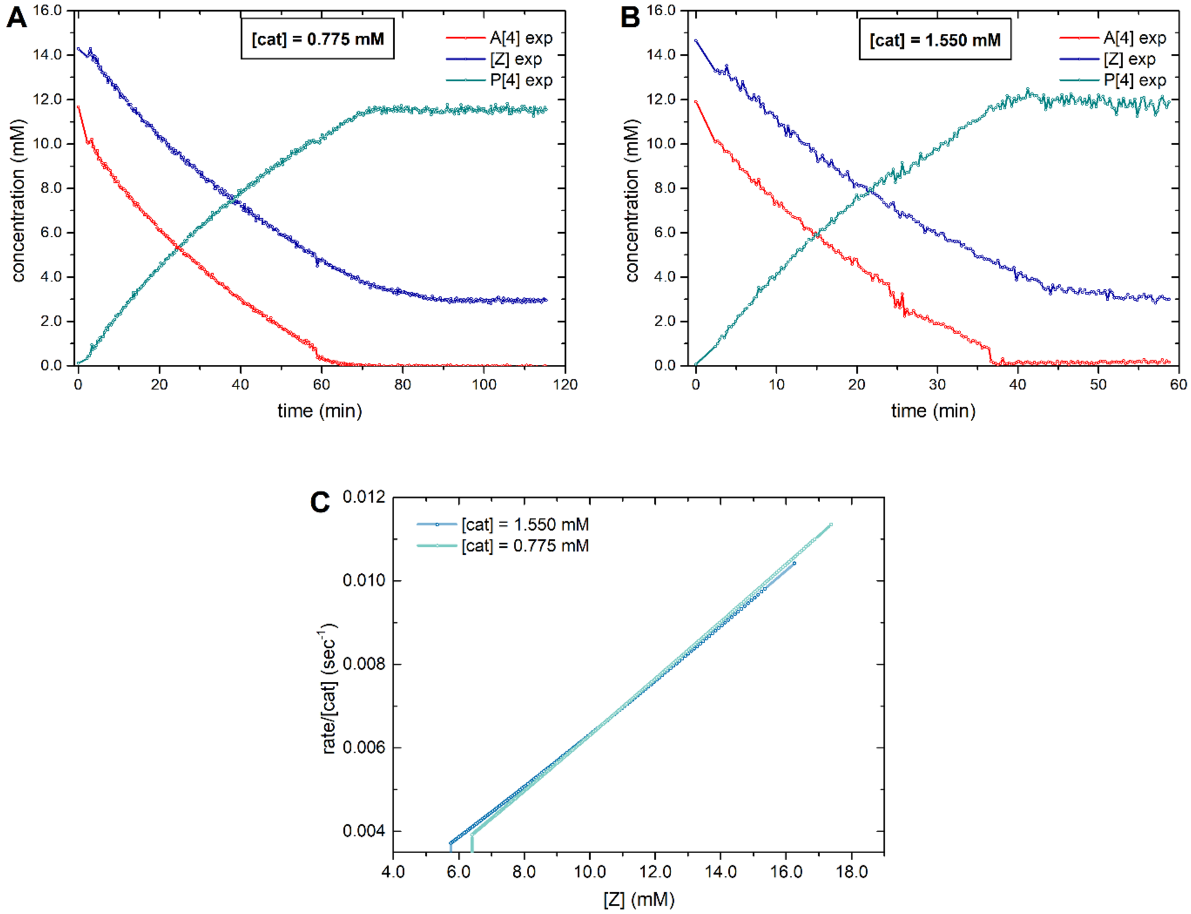

Figure S9. Kinetic profiles for experiments performed following general procedure F. (A) [cat]=0.775 mM; (B) [cat]=1.550 mM, (C) rate/[cat] vs. [Z].

3. Synthesis and characterization of bismuth(III)-acetylides and 5-bismuth-1,2,3-triazolides

All reactions were carried out under a nitrogen atmosphere with dry solvents ensuring anhydrous reaction conditions unless otherwise stated. Dry tetrahydrofuran (THF), acetone, diethyl ether (Et_2O), and methylene chloride (CH_2Cl_2) were obtained by passing commercially available pre-dried, oxygen-free formulations through activated alumina columns. Chemical reagents were purchased at the highest commercially available purity and used without further purification unless otherwise stated. Copper(I) iodide (98% purity), bismuth(III) bromide (98% purity), copper(I) trifluoromethanesulfonate toluene complex (90% technical grade) were purchased from Sigma-Aldrich. Triphenyl bismuth (99+% purity) and 3-chloroperbenzoic acid (<77% purity) were purchased from Alfa Aesar. Anhydrous potassium carbonate and ethylene glycol were purchased from Fischer Scientific. LCMS grade 2-propanol, hexane, and ethyl acetate were purchased from Fischer Scientific and used directly without drying or degassing. Aryl thiols and aryl halides were used as received and without additional purification. Silica gel (230-400 mesh) was purchased from Merck. Reactions progress was monitored both by thin-layer chromatography (TLC) on 0.25 mm E. Merck silica gel plates (60F-254) using UV light as a visualizing agent and by Agilent LC/MSD as well as Agilent HPLC/DAD spectrometer. E. Merck silica gel (60, particle size 0.040–0.063 mm) was used for flash column chromatography. NMR spectra were recorded on Varian Mercury 400, Varian VNMRS-500, or Varian VNMRS-600 spectrometer. Chemical shifts were referenced to residual solvent signals (Chloroform-d: δH = 7.26 ppm, δC = 77.16 ppm) as an internal reference. ^{19}F NMR spectra were externally referenced to 80% $CFCl_3$ in chloroform-*d*. The following abbreviations were used to describe NMR signal multiplicities: s = singlet, d =

doublet, t = triplet, q = quartet, quin = quintet, m = multiplet, b = broad. Infrared spectra were recorded in the range 4000-400 cm^{-1} on Bruker Alpha spectrometer using a diamond ATR unit. IR intensities are described as vw (very weak), w (weak), m (medium), s (strong), vs (very strong). X-ray crystallographic analysis was performed at UCSD on a Bruker Apex II Ultra2 CCD diffractometer equipped with Mo Kα radiation (crystallographic details for all structures are given in section 4). ORTEP drawings were prepared using the Mercury CSD program.[3] Further crystallographic details can be obtained from the Cambridge Crystallographic Data Centre (CCDC, 12 Union Road, Cambridge CB21EZ, UK (Fax: (+44) 1223-336-033; e-mail: deposit@ccdc.cam.ac.uk) on quoting the deposition no CCDC 2124668-2124680. Yields refer to chromatographically and spectroscopically (^{1}H NMR) pure materials unless otherwise is stated.

3.1 General Procedure 1. Synthesis of diphenyl sulfides

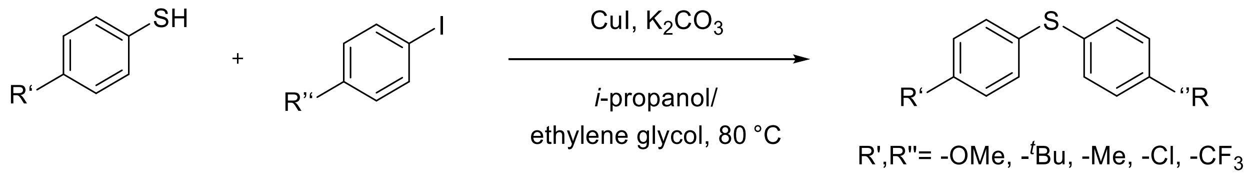

Adapted from a procedure of S. L. Buchwald and co-workers[4].

(4-methoxyphenyl)(phenyl) sulfide 1. A dry 12-mL screw-capped glass vial sealed with rubber septum was purged with dry nitrogen, equipped with a magnetic stirring bar, and charged with copper(I) iodide (70.0 mg, 0.35 mmol), potassium carbonate (1.95 g, 14.0 mmol), and 1-iodo-4-methoxybenzene (1.30 g, 7.0 mmol). The vial and its contents were exposed to three vacuum-nitrogen cycles. Subsequently, 2-propanol (7.0 mL) and ethylene glycol (0.77 mL) were added together with the 4-methoxybenzenethiol (0.77 mg, 7.0 mmol) with a syringe. The stirred reaction mixture was heated up to 80°C and kept at this temperature overnight. The reaction mixture was allowed to cool to room temperature, filtered, and concentrated to give yellow liquid, which was purified by flashed column chromatography to yield a pale-yellow liquid as a product (yield: 0.65 g, 43 %). NMR characterization data were consistent with those previously reported[5].

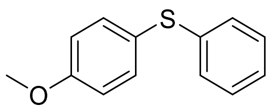

11

phenyl(p-tolyl) sulfide. Synthesized from 4-methylbenzenethiol (0.87 g, 7.0 mmol) and iodobenzene (1.43 g, 7.0 mmol) following general procedure 1, 1.08 g, 77 %, overnight, colorless liquid. NMR characterization data is consistent with those previously reported[5]

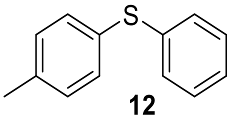

12

(4-chlorophenyl)(phenyl) sulfide. Synthesized from benzenethiol (0.77 g, 7.0 mmol) and 1-chloro-4-iodobenzene (1.97 g, 7.0 mmol) using the general procedure 1, 1.5 g, 97 %, overnight, colorless liquid. NMR characterization data is consistent with those previously reported[6].

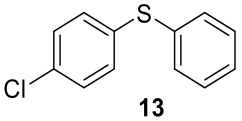

13

phenyl(4-(trifluoromethyl)phenyl) sulfide. Synthesized from benzenethiol (0.77 g, 7.0 mmol) and 1-iodo-4-(trifluoromethyl)benzene (1.90 g, 7.0 mmol) following general procedure 1, 0.88 g, 50 %, overnight, colorless liquid:

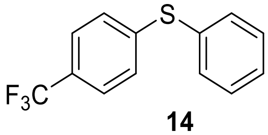

14

IR (υ[cm-1]) 3089 (vw), 3050 (vw), 2921 (vw), 1926 (vw), 1602 (m), 1582 (m), 1568 (w), 1496 (vw), 1477 (m), 1440 (m), 1401 (m), 1320 (s), 1189 (w), 1161 (s), 1127 (s), 1106 (vs), 1094 (vs), 1061 (vs), 1024 (m), 1011 (s), 997 (m), 959 (m), 908 (m), 833 (vs), 778 (w), 743 (vs), 727 (s), 695 (s), 687 (s), 631 (m), 615 (w), 592 (m), 522 (m), 494 (m), 480 (s), 435 (m), 403 (w).

^{1}H NMR (500 MHz, Chloroform-d) δ 7.50 – 7.46 (m, 4H), 7.42 – 7.36 (m, 3H), 7.28 (d, J = 0.9 Hz, 2H).

^{13}C NMR (126 MHz, Chloroform-d) δ 142.97, 133.68, 132.65, 129.82, 128.66, 128.18 (q, J = 32.5 Hz), 125.95 (q, J = 3.8 Hz) , 124.37 (q, J = 273.7 Hz).

^{19}F NMR (470 MHz, Chloroform-d) δ -62.51.

(4-methoxyphenyl)(4-(trifluoromethyl)phenyl) sulfide. Synthesized from 4-methoxybenzenethiol (0.98 g, 7.0 mmol) and 1-iodo-4-(trifluoromethyl)benzene (1.90 g, 7.0 mmol) following general procedure 1, 1.20 g, 60 %, overnight, colorless liquid:

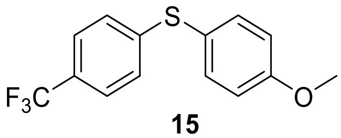

15

IR (υ[cm-1]) 3086 (vw), 3029 (w), 2973 (w), 2948 (w), 2846 (w), 2084 (vw), 2054 (vw), 1923 (w), 1670 (vw), 1601 (m), 1588 (m), 1569 (m), 1490 (m), 1466 (m), 1451 (m), 1441 (m), 1400 (m), 1356 (vw), 1327 (s), 1288 (m), 1252 (m), 1189 (m), 1165 (s), 1107 (vs), 1082 (vs), 1060 (s), 1030 (s), 1009 (s), 957 (m), 830 (vs), 811 (s), 799 (m), 776 (m), 724 (m), 699 (m), 644 (m), 627 (m), 590 (m), 532 (m), 505 (m), 492 (m), 430 (m).

^{1}H NMR (500 MHz, Chloroform-d) δ 7.47 (d, J = 8.9 Hz, 2H), 7.44 (d, J = 8.2 Hz, 2H), 7.14 (d, J = 8.2 Hz, 2H), 6.96 (d, J = 8.9 Hz, 2H), 3.85 (s, 3H).

^{13}C NMR (126 MHz, Chloroform-d) δ 160.77, 144.97, 136.85, 127.35 (q, J = 32.7 Hz), 126.55, 125.77 (q, J = 3.8 Hz), 124.32 (q, J = 271.7 Hz), 121.82, 115.52, 55.57.

^{19}F NMR (470 MHz, Chloroform-d) δ -62.39.

bis(4-(trifluoromethyl)phenyl) sulfide. Synthesized from 4-(trifluoromethyl)benzenethiol (0.89 g, 5.0 mmol) and 1-iodo-4-(trifluoromethyl)benzene (1.36 g, 5.0 mmol) following general procedure 1, 1.21 g, 75 %, overnight, colorless solid:

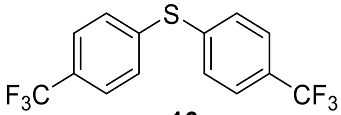

16

IR (υ[cm-1]) 3094 (vw), 3063 (vw), 2924 (vw), 1604 (m), 1574 (w), 1496 (w), 1401 (m), 1318 (vs), 1162 (s), 1119 (vs), 1103 (vs), 1082 (s), 1060 (vs), 1012 (s), 951 (w), 824 (s), 778 (m), 724 (w), 699 (m), 632 (w), 590 (m), 515 (m), 486 (m), 414 (w).

^{1}H NMR (500 MHz, Chloroform-d) δ 7.60 (d, J = 8.2 Hz, 1H), 7.46 (d, J = 8.5 Hz, 1H).

^{13}C NMR (126 MHz, Chloroform-d) δ 139.68, 131.17, 129.85 (q, J = 32.8 Hz), 126.42 (q, J = 3.8 Hz), 124.06 (q, J = 272.0 Hz).

^{19}F NMR (470 MHz, Chloroform-d) δ -62.77.

3.2 General Procedure 2. Synthesis of sulfonyldibenzenes

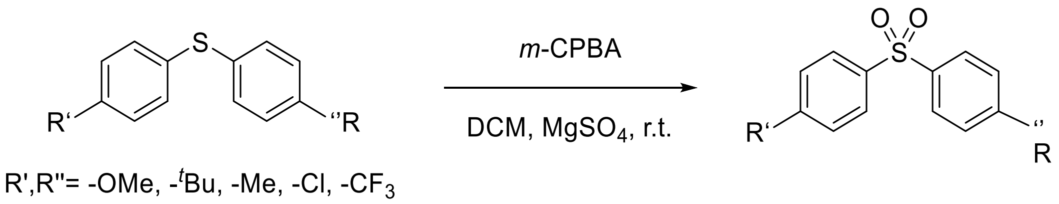

Adapted from a procedure of J. Thomas and co-workers[7].

1-methoxy-4-(phenylsulfonyl)benzene. To a 50 mL round-bottom flask equipped with a magnetic stir bar, 1.00g (4.62 mmol, 1.00 eq.) of (4-methoxyphenyl)(phenyl) sulfide in 25 mL of reagent grade DCM were added. To the clear solution, 5.56 g (46.2 mmol, 10.0 eq.) of anhydrous $MgSO_4$ were added followed by the slow addition of 2.40g of *m*-CPBA (oxidizing reagent) (13.86 mmol, 3.00 eq.). The reaction mixture was stirred at room temperature for 8 hours. Complete conversion of starting material was ensured monitoring of the reaction progress by TLC (hexane, Rf(product)=0.5, Rf (reagent)=0.4). The reaction mixture was transferred to a 250 mL separatory funnel and washed with sat. $NaHCO_3$ (2×40 mL) to ensure complete removal of any residual *m*-CPBA. The organic phase was dried over $MgSO_4$, filtered, and concentrated *in vacuo* to yield a pale yellow oil. The crude material was purified by flash column chromatography using hexane as eluent to give 1-methoxy-4-(phenylsulfonyl)benzene as a pale yellow solid (0.70 g, 98% yield).

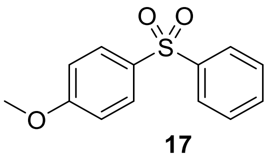

17

IR (υ[cm-1]) 3076 (w), 2847 (w), 2655 (w), 2595 (w), 2550 (w), 1689 (m), 1591 (m), 1574 (m), 1497 (m), 1468 (w), 1446 (m), 1416 (m), 1298 (s), 1261 (s), 1192 (w), 1183 (w), 1148 (s), 1105 (s), 1071 (m), 1018 (m), 997 (m), 914 (m), 897 (m), 850 (m), 833 (m), 803 (m), 748 (s), 730 (s), 719 (s), 711 (m), 687 (m), 667 (m), 656 (m), 627 (m), 575 (s), 554 (vs), 493 (m), 451 (w), 416 (w).

^{1}H NMR (500 MHz, Chloroform-d) δ 7.92 (d, J = 8.3 Hz, 2H), 7.88 (d, J = 8.2 Hz, 2H), 7.56 – 7.51 (m, 1H), 7.48 (t, J = 7.5 Hz, 2H), 6.96 (d, J = 9.0 Hz, 2H), 3.84 (s, 3H).

^{13}C NMR (126 MHz, Chloroform-d) δ 163.51, 142.52, 133.28, 132.96, 130.04, 129.33, 127.46, 114.65, 55.79.

1-chloro-4-(phenylsulfonyl)benzene. Synthesized from (4-chlorophenyl)(phenyl) sulfide (0.80 g, 3.60 mmol) following general procedure 2, 0.91 g, 98%, 4 hours, colorless solid. NMR characterization data is consistent with those previously reported[8].

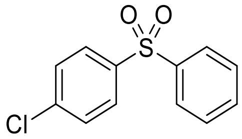

18

IR (υ[cm-1]) 3091 (w), 3068 (w), 2956 (w), 2956 (w), 2923 (w), 2853 (w), 1693 (m), 1596 (w), 1576 (m), 1474 (m), 1446 (m), 1417 (w), 1390 (m), 1319 (m), 1308 (s), 1279 (m), 1263 (m), 1176 (w), 1152 (s), 1106 (m), 1084 (s), 1069 (s), 1049 (m), 1026 (m), 1011 (m), 998 (m), 953 (w), 932 (w), 897 (w), 851 (w), 833 (m), 823 (m), 765 (m), 747 (vs), 718 (s), 700 (m), 685 (s), 653 (w), 608 (vs), 564 (vs), 521 (m), 496 (s), 486 (m), 467 (m), 435 (m).

1-(phenylsulfonyl)-4-(trifluoromethyl)benzene. Synthesized from phenyl(4-(trifluoromethyl)phenyl) sulfide (0.88 g, 3.40 mmol) following general procedure 2, 0.89 g, 91%, 4 hours, colorless solid.

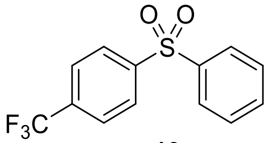

19

IR (υ[cm-1]) 3108 (vw), 3090 (vw), 3063 (vw), 1608 (vw), 1583 (vw), 1500 (vw), 1477 (vw), 1446 (w), 1404 (m), 1317 (s), 1296 (m), 1145 (s), 1106 (s), 1071 (s), 1058 (s), 1017 (m), 998 (m), 978 (w), 967 (w), 928 (w), 846 (m), 839 (m), 787 (m), 758 (m), 742 (m), 720 (s), 700 (s), 684 (s), 593 (vs), 557 (s), 497 (w), 480 (w), 456 (w), 421 (m).

^{1}H NMR (500 MHz, Chloroform-d) δ 8.07 (d, J = 8.2 Hz, 2H), 7.96 (d, J = 7.6 Hz, 2H), 7.75 (d, J = 8.2 Hz, 2H), 7.63 – 7.56 (m, 1H), 7.53 (t, J = 7.6 Hz, 2H).

^{13}C NMR (126 MHz, Chloroform-d) δ 145.33, 140.65, 134.89 (q, J=33.2 Hz), 133.89, 129.64, 128.28, 127.98, 126.54 (q, J=3.7 Hz), 123.20 (q, J=273.5 Hz).

4,4'-sulfonylbis(methoxybenzene). Synthesized from 4.4'-sulfonyldiphenol (0.50 g, 2.0 mmol) and iodomethane (2.84 g, 20.0 mmol) following a literature procedure[9], 0.54 g, 97 %, 4 hours, colorless solid:

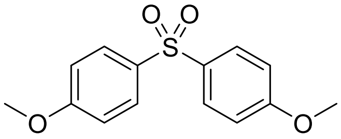

20

IR (υ[cm-1]) 2996 (w), 2982 (w), 2949 (w), 2921 (w), 2901 (w), 2841 (w), 1592 (m), 1576 (m), 1496 (m), 1458 (s), 1437 (s), 1386 (vs), 1317 (s), 1295 (s), 1253 (vs), 1185 (m), 1178 (m), 1145 (s), 1103 (s), 1020 (s), 973 (m), 939 (w), 846 (s), 829 (m), 819 (m), 801 (s), 717 (m), 677 (s), 642 (w), 630 (m), 623 (w), 566 (s), 547 (vs), 484 (m), 411 (vw).

^{1}H NMR (500 MHz, Chloroform-d) δ 7.85 (d, J = 9.1 Hz, 4H), 6.95 (d, J = 9.0 Hz, 4H), 3.83 (s, 6H).

^{13}C NMR (126 MHz, Chloroform-d) δ 163.22, 134.13, 129.66, 114.55, 55.76.

1-methoxy-4-((4-(trifluoromethyl)phenyl)sulfonyl)benzene. Synthesized from (4-methoxyphenyl)(4-(trifluoromethyl)phenyl) sulfide (1.20 g, 3.70 mmol) following general procedure 2, 1.16 g, 99 %, 4 hours, colorless solid:

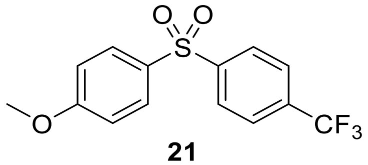

21

IR (υ[cm-1]) 3101 (vw), 2955 (vw), 2850 (vw), 1591 (m), 1575 (m), 1497 (m), 1463 (w), 1445 (w), 1415 (w), 1403 (m), 1322 (s), 1302 (m), 1261 (s), 1188 (m), 1155 (s), 1122 (s), 1105 (vs), 1074 (m), 1058 (vs), 1014 (s), 959 (w), 944 (w), 838 (m), 801 (m), 734 (w), 721 (s), 706 (s), 661 (s), 627 (w), 590 (vs), 549 (s), 515 (m), 479 (m), 424 (s).

^{1}H NMR (500 MHz, Chloroform-d) δ 8.04 (d, J = 9.0 Hz, 2H), 7.89 (d, J = 8.7 Hz, 2H), 7.74 (d, J = 8.9 Hz, 2H), 6.99 (d, J = 8.8 Hz, 2H), 3.85 (s, 3H).

^{13}C NMR (126 MHz, Chloroform-d) δ 164.00, 146.13, 134.65 (q, J = 33.1 Hz), 132.11, 130.31, 127.99, 126.50 (q, J = 3.8 Hz), 123.26 (q, J = 273.0 Hz), 114.92, 55.86.

4,4'-sulfonylbis((trifluoromethyl)benzene). Synthesized from bis(4-(trifluoromethyl)phenyl) sulfide (1.0 g, 3.10 mmol) following general procedure 2, 0.55 g, 50 %, 4 hours, colorless solid:

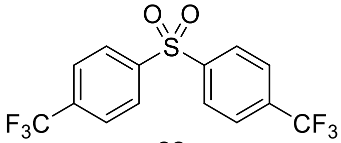

22

IR (υ[cm-1]) 3105 (vw), 3056 (vw), 2927 (vw), 1609 (vw), 1406 (m), 1316 (s), 1158 (s), 1129 (vs), 1105 (vs), 1073 (s), 1058 (vs), 1015 (s), 962 (m), 840 (s), 790 (m), 734 (m), 716 (s), 703 (s), 676 (w), 616 (s), 600 (s), 560 (s), 499 (w), 471 (w), 427 (s).

^{1}H NMR (500 MHz, Chloroform-d) δ 8.08 (d, J = 8.2 Hz, 4H), 7.81 (d, J = 8.4 Hz, 4H).

^{13}C NMR (126 MHz, Chloroform-d) δ 144.34, 135.59 (q, J = 33.2 Hz), 128.60, 126.85 (q, J = 3.7 Hz), 123.11 (q, J = 273.2 Hz).

^{19}F NMR (470 MHz, Chloroform-d) δ -63.34.

3.3 General Procedure 3. Synthesis of 10-phenyl-10H-dibenzo[b,e][1,4]thiabismine 5,5-dioxides

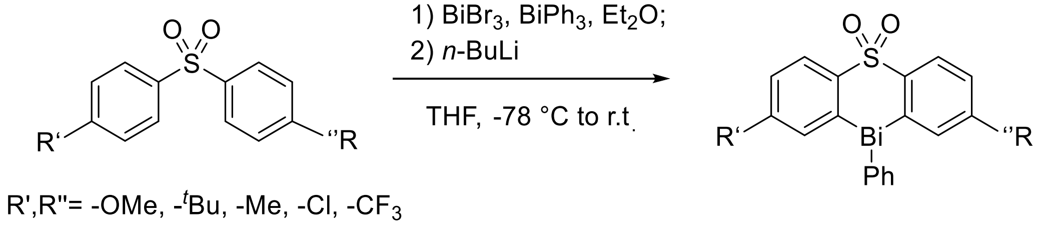

Adapted from a procedure of Suzuki H. and co-workers[10].

2-methoxy-10-phenyl-10H-dibenzo[b,e][1,4]thiabismine 5,5-dioxide. Synthesized from 1-methoxy-4-(phenylsulfonyl)benzene (0.65 g, 2.60 mmol) following a literature procedure[10], 1.23 g, 87 %, 4 hours, colorless solid:

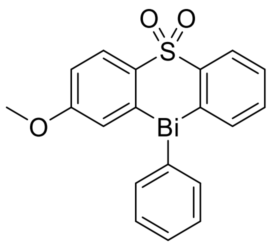

23

IR (υ[cm-1]) 3047 (w), 3005 (vw), 2958 (w), 2922 (m), 2851 (w), 1720 (w), 1567 (s), 1496 (vw), 1460 (m), 1427 (m), 1291 (s), 1265 (s), 1236 (m), 1221 (s), 1183 (w), 1145 (s), 1116 (s), 1094 (s), 1074 (s), 1025 (s), 1011 (s), 996 (m), 909 (w), 874 (m), 828 (m), 760 (m), 727 (vs), 715 (s), 706 (m), 695 (s), 661 (m), 650 (s), 582 (s), 561 (vs), 543 (s), 513 (m), 495 (m), 483 (m), 460 (m), 445 (m), 431 (m).

^{1}H NMR (500 MHz, Chloroform-d) δ 8.34 (d, J = 7.7 Hz, 1H), 8.32 (d, J = 8.6 Hz, 1H), 7.85 (d, J = 7.2 Hz, 1H), 7.79 (d, J = 7.9 Hz, 2H), 7.47 – 7.40 (m, 2H), 7.42 – 7.35 (m, 3H), 7.33 (t, J = 7.4 Hz, 1H), 6.85 (dd, J = 8.6, 2.6 Hz, 1H), 3.69 (s, 3H).

^{13}C NMR (126 MHz, Chloroform-d) δ 167.88, 166.20, 163.65, 160.47, 142.74, 138.80, 137.61, 133.32, 133.22, 130.99, 129.11, 128.73, 128.24, 126.73, 123.51, 112.90, 55.68.

10-phenyl-10H-dibenzo[b,e][1,4]thiabismine 5,5-dioxide. Synthesized from diphenyl sulfone (1.8 g, 8.5 mmol) following general procedure 3, 2.07 g, 63 %, overnight, colorless solid. NMR characterization data is consistent with those previously reported[11].

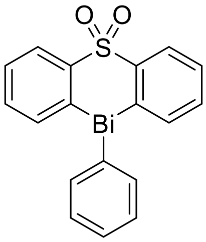

24

2-chloro-10-phenyl-10H-dibenzo[b,e][1,4]thiabismine 5,5-dioxide. Synthesized from 1-chloro-4-(phenylsulfonyl)benzene (0.78 g, 3.10 mmol) following general procedure 3, 0.79 g, 47 %, overnight, colorless solid:

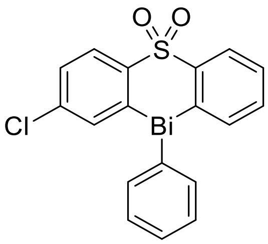

25

IR (υ[cm-1]) 3056 (w), 3039 (w), 2978 (vw), 2932 (vw), 1651 (vw), 1548 (m), 1474 (w), 1427 (m), 1362 (w), 1298 (s), 1284 (m), 1249 (m), 1150 (s), 1122 (m), 1086 (s), 1075 (s), 1055 (m), 1012 (m), 996 (m), 953 (w), 912 (w), 877 (w), 851 (w), 821 (m), 780 (m), 754 (m), 728 (s), 711 (m), 694 (s), 649 (m), 638 (m), 612 (vs), 568 (vs), 527 (m), 502 (m), 476 (m), 467 (m), 442 (m), 426 (m).

^{1}H NMR (500 MHz, Chloroform-d) δ 8.37 (d, J = 7.6 Hz, 1H), 8.30 (d, J = 8.3 Hz, 1H), 7.87 (d, J = 7.0 Hz, 1H), 7.82 (d, J = 2.0 Hz, 1H), 7.78 (d, J = 7.9 Hz, 2H), 7.47 (t, J = 7.7 Hz, 2H), 7.45 – 7.34 (m, 4H).

^{13}C NMR (126 MHz, Chloroform-d) δ 166.45, 159.23, 141.67, 140.90, 140.15, 138.74, 137.74, 137.32, 133.82, 131.29, 129.04, 128.51, 128.48, 128.41, 127.41.

10-phenyl-2-(trifluoromethyl)-10H-dibenzo[b,e][1,4]thiabismine 5,5-dioxide. Synthesized from 1-(phenylsulfonyl)-4-(trifluoromethyl)benzene (2.44 g, 8.52 mmol) following general procedure 3, 1.22 g, 25 %, overnight, colorless solid:

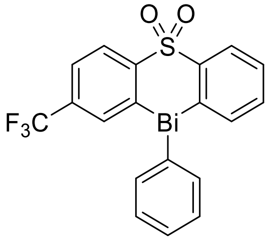

26

IR (υ[cm-1]) 3069 (w), 3052 (w), 1954 (w), 1911 (vw), 1852 (vw), 1786 (vw), 1587 (w), 1562 (m), 1475 (w), 1447 (w), 1427 (m), 1383 (m), 1320 (s), 1304 (vs), 1288 (s), 1256 (m), 1154 (s), 1118 (vs), 1097 (s), 1079 (s), 1064 (vs), 1022 (m), 1012 (m), 996 (m), 964 (m), 909 (w), 898 (m),

855 (w), 834 (m), 802 (m), 766 (m), 741 (m), 724 (s), 709 (s), 699 (s), 651 (m), 638 (w), 622 (m), 601 (s), 559 (s), 513 (m), 492 (w), 469 (m), 450 (w), 438 (m), 428 (m), 404 (w).

^{1}H NMR (500 MHz, Chloroform-*d*) δ 8.47 (d, *J* = 8.1 Hz, 1H), 8.40 (dd, *J* = 7.6, 1.5 Hz, 1H), 8.09 (s, 1H), 7.90 (dd, *J* = 7.2, 1.3 Hz, 1H), 7.76 (dd, *J* = 8.0, 1.4 Hz, 2H), 7.67 (d, *J* = 8.1 Hz, 1H), 7.50 – 7.33 (m, 5H).

^{13}C NMR (126 MHz, Chloroform-d) δ 166.48, 159.71, 145.43, 141.01, 138.67, 137.82, 134.80 (q, J = 32.4 Hz), 134.50 (q, J = 3.6 Hz), 134.02, 131.31, 129.15, 128.62, 127.79, 127.35, 127.20, 125.49 (q, J = 3.7 Hz), 123.55 (q, J = 273.2 Hz).

^{19}F NMR (470 MHz, Chloroform-d) δ -62.92.

2,8-dimethoxy-10-phenyl-10H-dibenzo[b,e][1,4]thiabismine 5,5-dioxide. Synthesized from 4,4'-sulfonylbis(methoxybenzene) (1.0 g, 3.60 mmol) following general procedure 3, 1.20 g, 59 %, overnight, colorless solid:

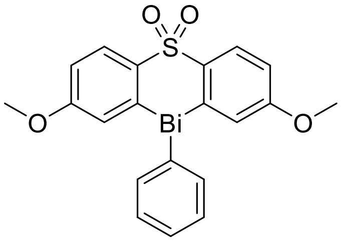

27

IR (υ[cm-1]) 3107 (w), 3059 (w), 2934 (w), 1609 (w), 1593 (w), 1570 (m), 1497 (w), 1460 (w), 1427 (w), 1406 (m), 1314 (s), 1265 (m), 1221 (m), 1156 (s), 1128 (vs), 1104 (vs), 1074 (s), 1058 (s), 1015 (s), 963 (m), 839 (s), 791 (m), 734 (m), 716 (s), 704 (m), 680 (m), 654 (w), 616 (m), 600 (m), 560 (s), 503 (w), 472 (w), 452 (vw), 427 (m).

^{1}H NMR (500 MHz, Chloroform-d) δ 8.28 (d, J = 8.6 Hz, 2H), 7.81 (d, J = 7.9 Hz, 2H), 7.44 (t, J = 7.6 Hz, 2H), 7.39 – 7.35 (m, 3H), 6.84 (dd, J = 8.6, 2.5 Hz, 2H), 3.69 (s, 6H).

^{13}C NMR (126 MHz, Chloroform-d) δ 172.32, 163.52, 160.04, 138.80, 134.23, 131.00, 128.76, 128.59, 123.50, 112.75, 55.68.

2,4-dimethoxy-10-phenyl-10H-dibenzo[b,e][1,4]thiabismine 5,5-dioxide. Synthesized from 2,4-dimethoxy-1-(phenylsulfonyl)benzene (0.80 g, 2.80 mmol) following general procedure 3, 0.75 g, 47 % , 4 hours, colorless solid:

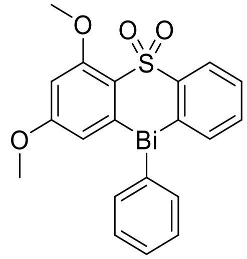

28

IR (υ[cm-1]) 3086 (vw), 3051 (w), 3033 (w), 2999 (w), 2980 (w), 2941 (w), 2829 (w), 2067 (vw), 1955 (vw), 1878 (vw), 1820 (vw), 1574 (m), 1556 (s), 1473 (w), 1462 (m), 1445 (m), 1419 (m), 1399 (m), 1298 (s), 1272 (vs), 1208 (s), 1181 (m), 1173 (m), 1142 (vs), 1109 (s), 1083 (m), 1067 (s), 1029 (s), 1009 (s), 995 (m), 951 (m), 923 (m), 854 (s), 845 (s), 754 (m), 728 (vs), 708 (s), 698 (s), 636 (s), 607 (s), 587 (vs), 576 (s), 537 (m), 512 (s), 484 (m), 449 (m), 426 (m), 414 (m).

^{1}H NMR (400 MHz, Chloroform-d) δ 8.35 (d, J = 6.4 Hz, 1H), 7.75 (d, J = 7.3 Hz, 1H), 7.65 (dd, J = 7.9, 1.5 Hz, 2H), 7.54 (d, J = 2.2 Hz, 1H), 7.37 – 7.20 (m, 5H), 6.54 (d, J = 2.2 Hz, 1H), 3.82 (s, 3H), 3.25 (s, 3H).

^{13}C NMR (101 MHz, Chloroform-d) δ 164.39, 162.32, 162.13, 157.19, 143.07, 141.64, 138.06, 137.90, 137.04, 133.27, 130.29, 128.02, 127.67, 126.57, 104.29, 103.03, 55.94, 55.57.

3.4 General Procedure 4. Synthesis of 10-iodo-10H-dibenzo[b,e][1,4]thiabismine 5,5-dioxides

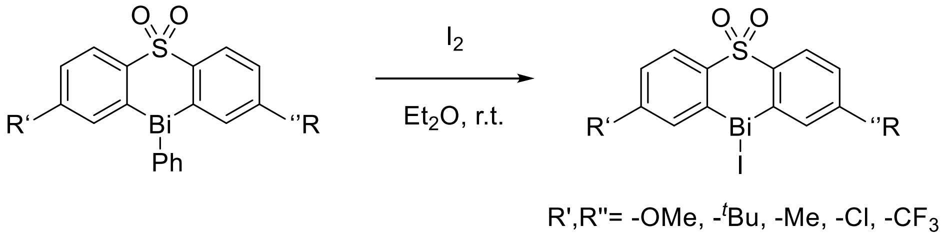

Adapted from a procedure of H. Suzuki and co-workers[10].

10-iodo-2-methoxy-10H-dibenzo[b,e][1,4]thiabismine 5,5-dioxide. Synthesized from 2-methoxy-10-phenyl-10H-dibenzo[b,e][1,4]thiabismine 5,5-dioxide (0.06 g, 11.4 mmol) following a literature procedure[10], 0.61 g, 10.8 mmol, 92 %, 4 hours, off-white solid:

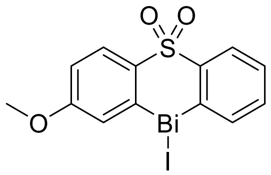

29

IR (υ[cm-1]) 2969 (m), 2953 (m), 2928 (m), 1592 (s), 1551 (s), 1496 (m), 1470 (m), 1445 (m), 1425 (s), 1384 (vs), 1267 (s), 1198 (m), 1145 (s), 1106 (m), 1090 (m), 1070 (m), 1017 (m), 962 (w), 898 (w), 874 (w), 831 (m), 803 (w), 760 (m), 731 (s), 684 (m), 671 (m), 658 (m), 577 (m), 513 (s), 430 (w), 408 (w).

^{1}H NMR (500 MHz, Chloroform-d) δ 9.21 – 9.18 (m, 1H), 8.81 (d, J = 2.4 Hz, 1H), 8.27 (d, J = 7.6 Hz, 1H), 8.24 (d, J = 8.4 Hz, 1H), 7.60 (t, J = 7.4 Hz, 1H), 7.47 (t, J = 7.6 Hz, 1H), 6.91 (dd, J = 8.5, 2.4 Hz, 1H), 3.89 (s, 3H).

^{13}C NMR (126 MHz, Chloroform-d) δ 166.31, 165.77, 163.62, 141.19, 140.29, 135.75, 131.12, 129.68, 128.84, 127.21, 126.39, 114.23, 56.02.

10-iodo-10H-dibenzo[b,e][1,4]thiabismine 5,5-dioxide. Synthesized from 10-phenyl-10H-dibenzo[b,e][1,4]thiabismine 5,5-dioxide (3.310 g, 6.0 mmol) following general procedure 4, 2.07 g, 63 %, 4 hours, colorless solid. NMR characterization data is consistent with those previously reported[11].

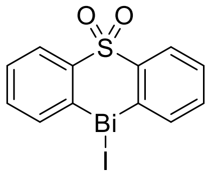

30

2-chloro-10-iodo-10H-dibenzo[b,e][1,4]thiabismine 5,5-dioxide. Synthesized from 2-chloro-10-phenyl-10H-dibenzo[b,e][1,4]thiabismine 5,5-dioxide (0.65 g, 1.11 mmol) following general procedure 4, 0.66 g, 69 %, 4 hours, off-white solid:

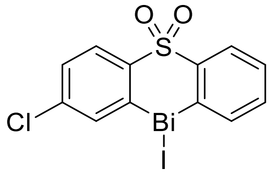

31

IR (υ[cm-1]) 3076 (w), 3046 (vw), 3031 (vw), 1547 (m), 1437 (w), 1423 (m), 1362 (w), 1302 (s), 1285 (m), 1248 (m), 1146 (m), 1135 (m), 1116 (m), 1087 (s), 1066 (s), 1024 (m), 1007 (m), 957 (w), 884 (m), 832 (m), 781 (m), 760 (s), 728 (m), 709 (m), 648 (m), 636 (w), 612 (s), 567 (vs), 524 (m), 500 (m), 483 (m), 463 (s), 424 (m).

^{1}H NMR (500 MHz, Chloroform-d) δ 9.23 (d, J = 7.4 Hz, 1H), 9.13 (dd, J = 2.0, 1.0 Hz, 1H), 8.30 (d, J = 7.7 Hz, 1H), 8.21 (d, J = 8.2 Hz, 1H), 7.65 (t, J = 7.4 Hz, 1H), 7.54 – 7.48 (m, 1H), 7.45 – 7.42 (m, 1H).

^{13}C NMR (126 MHz, Chloroform-d) δ 163.84, 157.42, 143.99, 140.65, 140.54, 140.11, 138.41, 136.28, 129.22, 129.10, 128.70, 127.86.

10-iodo-2-(trifluoromethyl)-10H-dibenzo[b,e][1,4]thiabismine 5,5-dioxide. Synthesized from 10-phenyl-2-(trifluoromethyl)-10H-dibenzo[b,e][1,4]thiabismine 5,5-dioxide (1.0 g, 1.75 mmol) following general procedure 4, 0.46 g, 42 %, 4 hours, off-white solid:

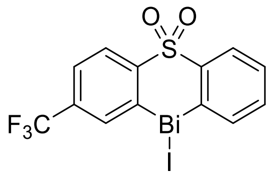

32

IR (υ[cm-1]) 3105 (vw), 3062 (vw), 3031 (vw), 1561 (w), 1440 (w), 1425 (w), 1390 (w), 1311 (vs), 1294 (m), 1254 (m), 1173 (s), 1148 (s), 1118 (vs), 1093 (m), 1073 (m), 1064 (s), 1029 (m), 1010 (m), 985 (w), 971 (w), 894 (m), 878 (w), 844 (m), 823 (w), 802 (w), 762 (m), 738 (m), 723 (m), 707 (m), 652 (w), 638 (w), 623 (m), 601 (m), 588 (m), 558 (s), 526 (w), 513 (m), 491 (w), 470 (m), 442 (m), 428 (m).

^{1}H NMR (500 MHz, Chloroform-d) δ 9.41 (s, 1H), 9.28 – 9.21 (m, 1H), 8.41 – 8.29 (m, 2H), 7.75 (d, J = 7.9 Hz, 1H), 7.67 (tt, J = 7.5, 1.5 Hz, 1H), 7.56 – 7.49 (m, 1H).

^{13}C NMR (126 MHz, Chloroform-d) δ 143.63, 140.51, 137.56, 136.36 129.07, 128.10, 127.38, 126.05, 123.36 (q. J = 274 Hz), 77.25, 76.99, 76.74.

^{19}F NMR (470 MHz, Chloroform-d) δ -62.81.

10-iodo-2,8-dimethoxy-10H-dibenzo[b,e][1,4]thiabismine 5,5-dioxide. Synthesized from 2,8-dimethoxy-10-phenyl-10H-dibenzo[b,e][1,4]thiabismine 5,5-dioxide (0.56 g, 1.78 mmol) following general procedure 4, 0.56 g, 51%, 4 hours, colorless solid:

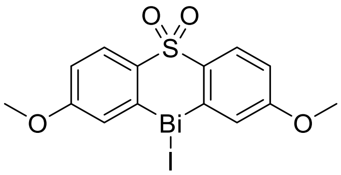

33

IR (υ[cm-1]) 3083 (w), 3011 (w), 2968 (w), 2930 (w), 2829 (w), 1561 (s), 1472 (w), 1452 (m), 1424 (m), 1400 (m), 1317 (m), 1294 (s), 1269 (s), 1223 (vs), 1189 (m), 1178 (m), 1157 (m), 1139 (m), 1124 (s), 1102 (s), 1071 (s), 1020 (s), 954 (m), 884 (m), 876 (m), 819 (vs), 712 (m), 681 (s), 652 (m), 636 (m), 581 (m), 567 (vs), 548 (s), 539 (vs), 504 (s), 466 (m), 435 (m).

^{1}H NMR (600 MHz, Chloroform-d) δ 8.79 (d, J = 2.4 Hz, 2H), 8.20 (d, J = 8.6 Hz, 2H), 6.89 (dd, J = 8.5, 2.5 Hz, 2H), 3.89 (s, 6H).

^{13}C NMR (151 MHz, Chloroform-d) δ 166.14, 165.60, 132.21, 129.12, 126.33, 114.08, 56.00.

10-iodo-2-methoxy-8-(trifluoromethyl)-10H-dibenzo[b,e][1,4]thiabismine 5,5-dioxide. Synthesized from 2-methoxy-10-phenyl-8-(trifluoromethyl)-10H-dibenzo[b,e][1,4]thiabismine 5,5-dioxide (0.40 g, 0.67 mmol) following general procedure 4, 0.16 g, 10.8 mmol, 37%, 4 hours, off-white solid:

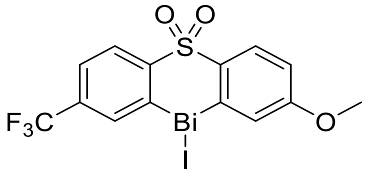

34

IR (υ[cm-1]) 3107 (w), 3015 (w), 2969 (w), 2934 (w), 2833 (w), 1566 (m), 1455 (m), 1425 (m), 1397 (m), 1381 (m), 1318 (s), 1302 (s), 1271 (s), 1255 (s), 1227 (s), 1177 (m), 1145 (s), 1122 (vs), 1103 (s), 1075 (s), 1064 (vs), 1019 (s), 1006 (s), 974 (m), 950 (m), 893 (m), 882 (m), 849 (m), 826 (m), 817 (s), 802 (m), 731 (w), 717 (s), 708 (m), 664 (m), 652 (s), 621 (m), 594 (s), 558 (m), 548 (s), 516 (m), 494 (m), 474 (m), 440 (m), 421 (w), 411 (w).

^{1}H NMR (500 MHz, Chloroform-d) δ 9.39 (s, 1H), 8.84 (d, J = 2.5 Hz, 1H), 8.34 (d, J = 8.2 Hz, 1H), 8.26 (d, J = 8.6 Hz, 1H), 7.73 (d, J = 8.5 Hz, 1H), 6.94 (dd, J = 8.6, 2.4 Hz, 1H), 3.91 (s, 3H).

^{13}C NMR (126 MHz, Chloroform-d) δ 166.72, 166.21, 163.06, 137.58 (q, J = 3.6 Hz), 137.07 (q, J = 33.0 Hz), 130.26, 130.16, 126.98, 126.70, 126.16 (q, J = 3.7 Hz), 123.44 (q, J = 272.8 Hz), 114.52, 56.11.

10-iodo-2,4-dimethoxy-10H-dibenzo[b,e][1,4]thiabismine 5,5-dioxide. Synthesized from 2,4-dimethoxy-10-phenyl-10H-dibenzo[b,e][1,4]thiabismine 5,5-dioxide (0.75 g, 1.33 mmol) following general procedure 4, 0.8 g, 98 %, 4 hours, olive solid:

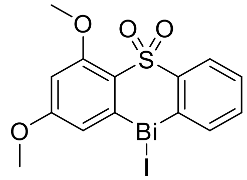

35

IR (υ[cm-1]) 3079 (w), 2998 (vw), 2954 (w), 2929 (w), 2828 (w), 1575 (m), 1554 (m), 1463 (m), 1456 (m), 1421 (m), 1397 (m), 1309 (s), 1278 (vs), 1251 (m), 1212 (s), 1189 (m), 1180 (m), 1160 (m), 1139 (s), 1108 (s), 1083 (m), 1073 (m), 1038 (s), 1027 (m), 1004 (m), 944 (m), 931 (m), 838 (s), 756 (m), 724 (s), 696 (m), 635 (m), 602 (vs), 572 (s), 541 (m), 511 (s), 473 (m), 449 (w), 432 (m).

^{1}H NMR (500 MHz, Chloroform-d) δ 10.33 (d, J = 7.7 Hz, 1H), 8.04 – 7.96 (m, 1H), 7.81 (dd, J = 2.7, 1.0 Hz, 1H), 7.68 (d, J = 4.8 Hz, 2H), 6.66 (d, J = 1.7 Hz, 1H), 3.97 (s, 3H), 3.87 (s, 3H).

^{13}C NMR (126 MHz, Chloroform-d) δ 161.84, 159.81, 148.80, 142.97, 142.39, 140.57, 130.45, 129.61, 110.15, 108.33, 103.94, 57.34, 56.40.

3.5 General Procedure 5. Synthesis of bismuth(III) acetylides with a diphenyl sulfone scaffold

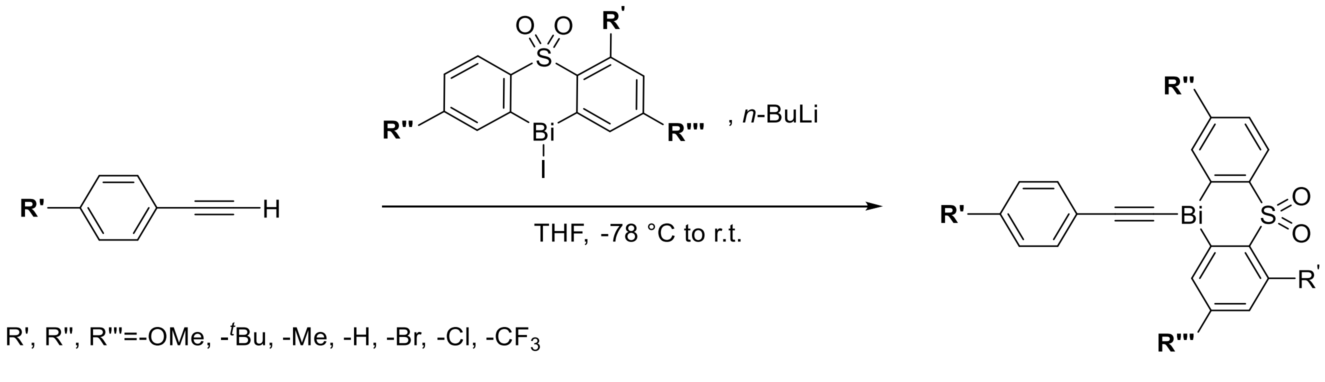

Adapted from a procedure of H Suzuki and co-workers[10].

10-((4-methoxyphenyl)ethynyl)-10H-dibenzo[b,e][1,4]thiabismine 5,5-dioxide. Synthesized from 1-ethynyl-4-methoxybenzene and **30** (0.90 g, 1.63 mmol) following a literature procedure[10], 0.72 g, 79%, 4 hours, colorless solid:

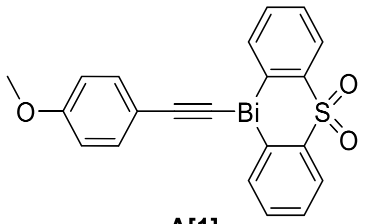

A[1]

IR (υ[cm-1]) 3036 (w), 2972 (w), 2939 (w), 2844 (w), 2099 (m), 1601 (m), 1561 (m), 1504 (m), 1459 (m), 1438 (m), 1285 (s), 1248 (s), 1208 (m), 1182 (m), 1171 (m), 1164 (m), 1143 (s), 1114 (m), 1085 (m), 1069 (m), 1025 (s), 1010 (m), 951 (m), 830 (s), 807 (m), 757 (m), 737 (s), 715 (s), 700 (m), 637 (m), 587 (vs), 563 (vs), 536 (m), 510 (m), 466 (m), 423 (m).

^{1}H NMR (500 MHz, Chloroform-d) δ 8.74 (d, J = 7.5 Hz, 2H), 8.33 (d, J = 7.7 Hz, 2H), 7.52 (t, J = 7.4 Hz, 2H), 7.46 – 7.36 (m, 4H), 6.83 (d, J = 8.2 Hz, 2H), 3.81 (s, 3H).

^{13}C NMR (126 MHz, Chloroform-d) δ 160.60, 159.91, 141.21, 138.19, 134.17, 133.71, 128.53, 127.65, 115.61, 114.06, 112.85, 83.62, 55.43.

10-((4-(tert-butyl)phenyl)ethynyl)-10H-dibenzo[b,e][1,4]thiabismine 5,5-dioxide.

Synthesized from 1-ethynyl-4-tert-butylbenzene and **30** (1.0 g, 1.81 mmol) following general procedure 5, 0.80 g, 76%, 4 hours, colorless solid:

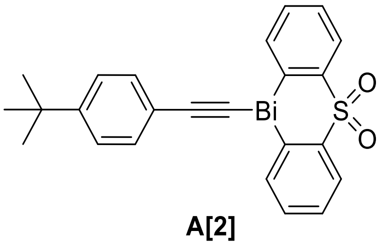

A[2]

IR (υ[cm-1]) 3044 (vs), 2960 (vs), 2108 (vs), 1562 (vs), 1501 (vs), 1461 (vs), 1438 (vs), 1393 (vs), 1364 (vs), 1301 (s), 1287 (s), 1268 (vs), 1251 (vs), 1209 (vs), 1183 (vs), 1147 (s), 1133 (vs), 1117 (vs), 1106 (vs), 1086 (vs), 1072 (vs), 1012 (vs), 952 (vs), 874 (vs), 831 (vs), 793 (vs), 759 (vs), 737 (s), 715 (vs), 701 (vs), 654 (vs), 637 (vs), 586 (s), 561 (s), 511 (s), 464 (s), 423 (vs).

^{1}H NMR (500 MHz, Chloroform-*d*) δ 8.74 (d, *J* = 7.2 Hz, 2H), 8.33 (d, *J* = 7.7 Hz, 2H), 7.51 (t, *J* = 7.4 Hz, 2H), 7.45 – 7.37 (m, 4H), 7.32 (d, *J* = 8.2 Hz, 2H), 1.30 (s, 9H).

^{13}C NMR (126 MHz, Chloroform-d) δ 160.66, 152.01, 141.19, 138.21, 134.19, 131.97, 128.53, 127.66, 125.45, 120.46, 112.80, 83.96, 34.95, 31.31.

10-(p-tolylethynyl)-10H-dibenzo[b,e][1,4]thiabismine 5,5-dioxide. Synthesized from 1-ethynyl-4-methylbenzene and **30** (0.77 g, 1.39 mmol) following general procedure 5, 0.41 g, 54%, 4 hours, colorless solid:

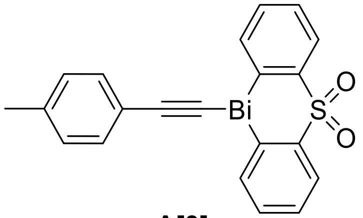

A[3]

IR (υ[cm-1]) 3025 (w), 2956 (w), 2924 (w), 2099 (m), 1603 (w), 1562 (m), 1503 (m), 1436 (m), 1375 (w), 1285 (s), 1250 (m), 1201 (m), 1178 (m), 1163 (m), 1144 (s), 1117 (m), 1105 (m), 1086

(m), 1069 (m), 1027 (m), 1010 (m), 941 (m), 833 (w), 811 (m), 756 (s), 739 (s), 715 (s), 700 (m), 637 (m), 586 (vs), 563 (vs), 529 (m), 511 (m), 486 (m), 463 (m), 415 (m).

^{1}H NMR (500 MHz, Chloroform-d) δ 8.75 (d, J = 7.4 Hz, 2H), 8.33 (d, J = 7.7 Hz, 2H), 7.52 (t, J = 7.4 Hz, 2H), 7.43 (t, J = 7.6 Hz, 2H), 7.35 (d, J = 7.6 Hz, 2H), 7.11 (d, J = 7.9 Hz, 2H), 2.35 (s, 3H).

^{13}C NMR (126 MHz, Chloroform-d) δ 160.64, 141.20, 138.85, 138.20, 134.20, 132.11, 129.84, 129.19, 128.54, 127.67, 120.42, 112.89, 21.66.

10-(phenylethynyl)-10H-dibenzo[b,e][1,4]thiabismine 5,5-dioxide. Synthesized from ethynylbenzene and **30** (1.0 g, 1.81 mmol) following general procedure 5, 0.85 g, 90%, 4 hours, colorless solid:

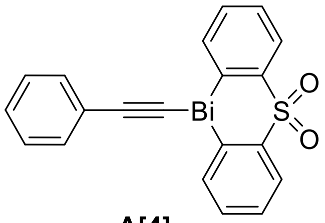

A[4]

IR (υ[cm-1]) 3054 (vw), 3036 (vw), 2111 (w), 1634 (vw), 1596 (w), 1561 (w), 1486 (m), 1437 (m), 1299 (s), 1287 (m), 1249 (m), 1204 (m), 1177 (w), 1165 (w), 1147 (s), 1133 (m), 1116 (m), 1087 (m), 1071 (m), 1026 (m), 1011 (m), 951 (w), 919 (w), 875 (w), 844 (w), 800 (w), 785 (m), 758 (s), 738 (s), 712 (m), 692 (m), 636 (m), 585 (vs), 562 (vs), 538 (m), 530 (m), 509 (m), 465 (m), 424 (m).

^{1}H NMR (500 MHz, Chloroform-*d*) δ 8.75 (d, *J* = 7.3 Hz, 2H), 8.33 (d, *J* = 7.7 Hz, 2H), 7.52 (t, *J* = 7.4 Hz, 2H), 7.49 – 7.40 (m, 4H), 7.32 – 7.29 (m, 3H).

^{13}C NMR (126 MHz, Chloroform-d) δ 160.66, 141.19, 140.45, 138.18, 134.23, 132.20, 128.95, 128.58, 128.44, 127.70, 123.51, 112.53.

10-((4-bromophenyl)ethynyl)-10H-dibenzo[b,e][1,4]thiabismine 5,5-dioxide. Synthesized from 1-bromo-4-ethynylbenzene and **30** (1.0 g, 1.81 mmol) following general procedure 5, 0.86 g, 79%, 4 hours, colorless solid:

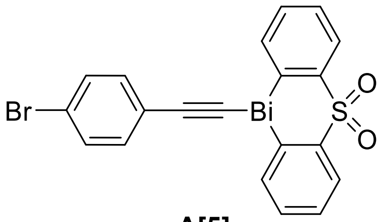

A[5]

IR (υ[cm-1]) 3056 (vw), 3038 (vw), 2105 (w), 1636 (w), 1582 (w), 1562 (w), 1482 (m), 1466 (w), 1438 (m), 1391 (w), 1285 (s), 1250 (m), 1200 (m), 1177 (w), 1165 (w), 1143 (m), 1118 (m), 1106 (m), 1086 (m), 1069 (m), 1028 (m), 1009 (m), 943 (w), 819 (m), 785 (w), 759 (m), 741 (s), 715 (m), 700 (m), 637 (m), 598 (m), 586 (vs), 563 (vs), 525 (m), 512 (m), 463 (m), 421 (w), 408 (w).

^{1}H NMR (500 MHz, Chloroform-d) δ 8.73 (d, J = 7.3 Hz, 2H), 8.34 (d, J = 7.7 Hz, 2H), 7.53 (t, J = 7.4 Hz, 2H), 7.44 (t, J = 7.6 Hz, 4H), 7.31 (d, J = 7.7 Hz, 2H).

^{13}C NMR (126 MHz, Chloroform-d) δ 160.66, 141.17, 138.18, 134.28, 133.60, 131.71, 128.65, 127.78, 127.71, 122.86, 122.48, 115.55, 111.21.

10-((4-bromophenyl)ethynyl)-10H-dibenzo[b,e][1,4]thiabismine 5,5-dioxide. Synthesized from 1-ethynyl-4-(trifluoromethyl)benzene and **30** (1.0 g, 1.81 mmol) following general procedure 5, 0.60 g, 56 %, 4 hours, colorless solid:

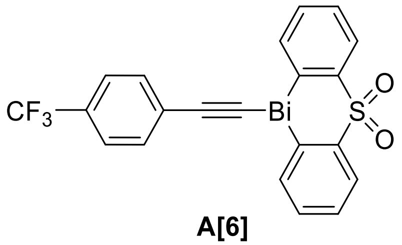

A[6]

IR (υ[cm-1]) 3056 (vw), 2960 (vw), 2921 (vw), 2114 (vw), 1609 (m), 1562 (w), 1509 (vw), 1437 (w), 1427 (w), 1403 (w), 1319 (s), 1299 (m), 1285 (s), 1254 (m), 1207 (m), 1179 (m), 1166 (m), 1151 (s), 1123 (s), 1103 (s), 1087 (m), 1064 (s), 1027 (m), 1014 (m), 946 (w), 869 (vw), 845 (m), 838 (m), 815 (w), 769 (m), 760 (m), 737 (s), 713 (m), 700 (m), 638 (w), 596 (m), 585 (vs), 564 (vs), 523 (m), 514 (m), 464 (s), 422 (m).

^{1}H NMR (500 MHz, Chloroform-*d*) δ 8.74 (d, *J* = 7.3 Hz, 2H), 8.35 (d, *J* = 7.7 Hz, 2H), 7.58 – 7.51 (m, 6H), 7.44 (t, *J* = 7.6 Hz, 2H).

^{13}C NMR (126 MHz, Chloroform-d) δ 160.80, 141.13, 138.08, 134.31, 132.37, 130.17 (q, J = 32.6 Hz), 128.68, 127.80, 127.33, 125.35 (q, J = 3.5 Hz), 123.97 (q, J = 272.3 Hz), 115.72, 110.58.

^{19}F NMR (470 MHz, Chloroform-d) δ -62.85.

2,8-dimethoxy-10-(p-tolylethynyl)-10H-dibenzo[b,e][1,4]thiabismine 5,5-dioxide.

Synthesized from 1-ethynyl-4-methylbenzene and **33** (0.50 g, 0.82 mmol) following general procedure 5, 0.30 g,0.550 mmol, 61 %, overnight, pale yellow solid:

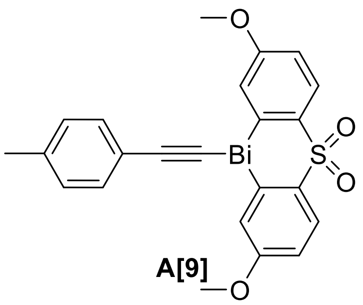

A[9]

IR (υ[cm-1]) 3062 (w), 2937 (w), 2838 (w), 2051 (vw), 1592 (m), 1573 (m), 1494 (s), 1457 (m), 1438 (m), 1414 (m), 1318 (m), 1308 (m), 1292 (s), 1256 (vs), 1176 (m), 1146 (vs), 1102 (vs), 1072 (s), 1014 (s), 831 (m), 804 (s), 717 (m), 679 (s), 637 (m), 626 (m), 553 (vs), 486 (m), 436 (w), 414 (vw).

^{1}H NMR (500 MHz, Chloroform-d) δ 8.30 (d, J = 2.5 Hz, 2H), 8.22 (d, J = 8.6 Hz, 2H), 7.36 (d, J = 7.8 Hz, 2H), 7.11 (d, J = 7.8 Hz, 2H), 6.86 (dd, J = 8.6, 2.5 Hz, 2H), 3.84 (s, 6H), 2.35 (s, 3H).

^{13}C NMR (126 MHz, Chloroform-d) δ 164.22, 162.21, 138.85, 133.46, 132.05, 129.23, 129.01, 123.81, 120.45, 113.49, 113.15, 110.15, 55.84, 21.66.

2-chloro-10-(p-tolylethynyl)-10H-dibenzo[b,e][1,4]thiabismine 5,5-dioxide. Synthesized from 1-ethynyl-4-methylbenzene and **31** (0.20 g, 0.34 mmol) following general procedure 5, 0.13 g, 66%, overnight, pale-yellow solid:

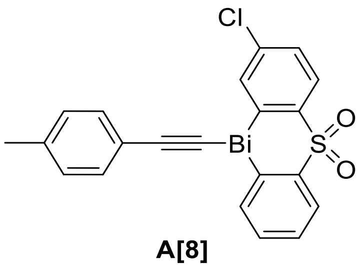

A[8]

IR (υ[cm-1]) 2957 (m), 2920 (vs), 2851 (s), 2098 (w), 1710 (w), 1601 (m), 1548 (m), 1504 (w), 1461 (m), 1439 (w), 1376 (w), 1305 (m), 1285 (m), 1248 (m), 1147 (m), 1117 (m), 1086 (s), 1071 (s), 1010 (m), 956 (w), 915 (vw), 884 (w), 854 (vw), 818 (m), 779 (m), 761 (s), 728 (m), 711 (m), 650 (w), 638 (w), 611 (vs), 568 (vs), 526 (m), 501 (w), 477 (m), 465 (m), 420 (w).

^{1}H NMR (500 MHz, Chloroform-d) δ 8.75 (d, J = 7.3 Hz, 1H), 8.71 (d, J = 2.0 Hz, 1H), 8.32 (d, J = 7.7 Hz, 1H), 8.24 (d, J = 8.2 Hz, 1H), 7.54 (t, J = 7.3 Hz, 1H), 7.44 (t, J = 7.6 Hz, 1H), 7.40 (dd, J = 8.3, 2.0 Hz, 1H), 7.37 (d, J = 7.7 Hz, 2H), 7.13 (d, J = 8.6 Hz, 2H), 2.36 (s, 3H).

^{13}C NMR (126 MHz, Chloroform-d) δ 161.88, 161.16, 141.75, 141.04, 139.42, 139.07, 138.24, 138.10, 134.45, 132.14, 129.25, 128.74, 120.14, 113.80, 113.16, 21.68.

2-trifluoro-10-(p-tolylethynyl)-10H-dibenzo[b,e][1,4]thiabismine 5,5-dioxide. Synthesized from 1-ethynyl-4-methylbenzene and **32** (0.20 g, 0.32 mmol) following general procedure 5, 0.10 g, 51%, overnight, pale-yellow solid:

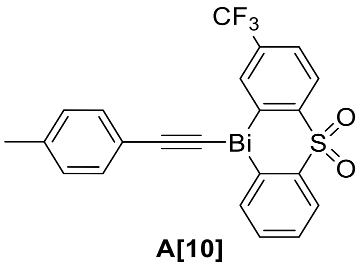

A[10]

IR (υ[cm-1]) 3099 (w), 3081 (w), 3062 (w), 2045 (vw), 1607 (vw), 1582 (vw), 1476 (w), 1446 (m), 1403 (m), 1321 (vs), 1294 (s), 1156 (vs), 1122 (s), 1104 (vs), 1071 (s), 1060 (vs), 1016 (m), 997 (m), 962 (w), 849 (m), 838 (m), 787 (w), 764 (m), 740 (m), 720 (vs), 701 (m), 689 (m), 604 (s), 593 (s), 557 (s), 422 (m).

^{1}H NMR (500 MHz, Chloroform-d) δ 9.02 (s, 1H), 8.76 (d, J = 7.3 Hz, 1H), 8.41 (d, J = 8.0 Hz, 1H), 8.35 (d, J = 7.5 Hz, 1H), 7.70 (d, J = 8.0 Hz, 1H), 7.56 (t, J = 7.4 Hz, 1H), 7.46 (t, J = 7.5 Hz, 1H), 7.34 (d, J = 7.8 Hz, 2H), 7.12 (d, J = 7.8 Hz, 2H), 2.36 (s, 3H).

^{13}C NMR (126 MHz, Chloroform-d) δ 161.60, 161.51, 144.74, 140.40, 139.13, 138.33, 135.38, 135.18 (q, J=3.27 Hz), 134.69, 132.07, 129.27, 128.86, 128.23, 127.53, 125.78 (q, J=3.67 Hz), 120.03, 113.88, 77.16, 21.68.

^{19}F NMR (470 MHz, Chloroform-d) δ -62.80.

2-methoxy-10-(p-tolylethynyl)-10H-dibenzo[b,e][1,4]thiabismine 5,5-dioxide. Synthesized from 1-ethynyl-4-methylbenzene and **29** (0.47 g, 0.80 mmol) following general procedure 5, 0.35 g, 60%, overnight, colorless solid:

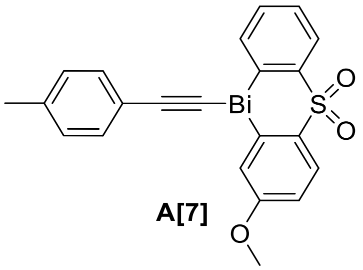

A[7]

IR (υ[cm-1]) 3063 (w), 2943 (w), 2846 (w), 2107 (w), 1591 (m), 1571 (m), 1497 (m), 1459 (m), 1446 (m), 1312 (s), 1297 (s), 1262 (vs), 1229 (m), 1192 (w), 1183 (w), 1148 (vs), 1105 (vs), 1070 (s), 1017 (s), 997 (m), 931 (w), 862 (w), 833 (s), 821 (m), 803 (m), 764 (m), 755 (m), 730 (s), 710 (m), 687 (m), 657 (m), 627 (m), 575 (s), 554 (vs), 485 (m), 454 (m), 424 (w).

^{1}H NMR (500 MHz, Chloroform-*d*) δ 8.72 (d, *J* = 6.1 Hz, 1H), 8.32 (d, *J* = 2.5 Hz, 1H), 8.29 (d, *J* = 6.4 Hz, 1H), 8.26 (d, *J* = 8.6 Hz, 1H), 7.92 (d, *J* = 7.0 Hz, 0.5H), 7.88 (d, *J* = 8.8 Hz, 0.5H), 7.50

(t, *J* = 8.2 Hz, 1H), 7.41 (td, *J* = 7.6, 1.2 Hz, 1.5H), 7.35 (d, *J* = 7.9 Hz, 2H), 7.11 (d, *J* = 7.8 Hz, 2H), 6.96 (d, *J* = 8.8 Hz, 0.5H), 6.87 (dd, *J* = 8.6, 2.5 Hz, 1H), 3.84 (s, 3H), 2.35 (s, 3H).

^{13}C NMR (126 MHz, Chloroform-d) δ 164.36, 162.60, 160.29, 142.14, 138.85, 138.11, 133.96, 132.08, 130.03, 129.53, 129.33, 129.21, 128.48, 127.46, 127.15, 123.80, 120.43, 114.64, 113.64, 113.01, 55.85, 21.66.

3.6 General Procedure 6. Synthesis of 5-bismuth(III) triazolides

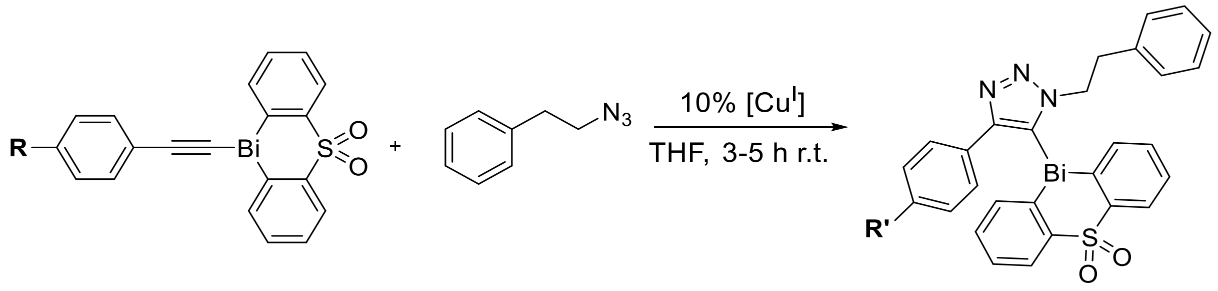

R =-OMe, -^{t}Bu, -Me, -H, -Br, -CF_3

Adapted from a procedure of B. T. Worrell and co-workers[11].

10-(4-(4-methoxyphenyl)-1-phenethyl-1H-1,2,3-triazol-5-yl)-10H-dibenzo[b,e][1,4]thiabismine 5,5-dioxide. To a 50 mL round-bottom flask equipped with a magnetic stir bar, 0.139 g (0.25 mmol, 1.00 equiv.) of 10-((4-methoxyphenyl)ethynyl)-10H-dibenzo[b,e][1,4]thiabismine 5,5-dioxide **A[1]** and 0.055 g (0.37 mmol, 1.50 equiv.) of (2-azidoethyl)benzene in 3 mL of reagent grade dry THF were added. 1 mL of a copper(I) trifluoromethanesulfonate toluene complex solution in dry THF (0.05 mmol, 0.2 equiv.) was injected directly into the clear solution of **A[1]** and (2-azidoethyl)benzene. Immediately, the solution turned bright yellow (for all substrates) signalizing the formation of bismuth(III)-acetylenide-Cu(I) π-complex. The reaction mixture was stirred for 2-3 hours at room temperature. After this time, the reaction mixture was dissolved with reagent grade DCM (30 mL), transferred to a 250 mL separatory funnel, and washed with birne:NH_4OH(10%) solution (5:1) 2×30 mL NH_4OH to ensure complete removal of any residual catalytic species. The organic phase was dried over $MgSO_4$, filtered, and concentrated *in vacuo*. The residue was dissolved in 1 mL of dry DCM and added dropwise to 200 mL of vigorously stirred hexanes. The formed precipitate was filtered

and dried in a vacuum to yield a colorless solid (0.113 g, 64 %). *Due to the mediocre performance of recrystallization as a purification technique, the observable yields are considered to be higher***.**

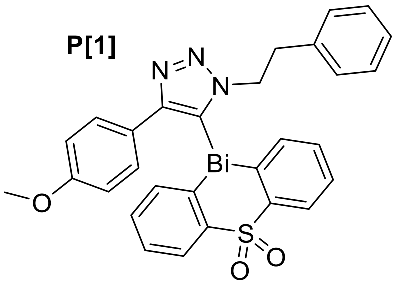

IR (υ[cm-1]) 3032 (w), 2991 (w), 2957 (w), 2929 (w), 2851 (w), 2834 (w), 1614 (m), 1576 (w), 1561 (w), 1536 (m), 1498 (m), 1440 (m), 1427 (m), 1398 (w), 1336 (w), 1306 (m), 1290 (m), 1243 (s), 1197 (w), 1174 (m), 1152 (s), 1120 (m), 1106 (m), 1087 (m), 1073 (m), 1037 (m), 1023 (m), 1011 (m), 982 (m), 950 (w), 909 (w), 876 (vw), 834 (m), 810 (m), 775 (w), 759 (m), 734 (s), 717 (m), 696 (s), 669 (m), 636 (w), 614 (m), 586 (vs), 564 (vs), 547 (m), 532 (m), 512 (m), 498 (m), 462 (m), 426 (w).

^{1}H NMR (500 MHz, Chloroform-d) δ 8.53 (d, J = 7.7 Hz, 2H), 7.97 (s, 2H), 7.51 (t, J = 7.9 Hz, 2H), 7.41 (s, 2H), 7.26 (s, 4H), 6.72 (s, 5H), 3.88 (s, 5H), 3.11 (s, 2H).

^{13}C NMR (126 MHz, Chloroform-d) δ 159.54, 157.08, 156.44, 152.42, 141.10, 138.05, 137.30, 134.01, 129.55, 129.23, 128.73, 128.60, 127.78, 127.05, 124.89, 113.82, 77.16, 55.39, 53.34, 36.74.

10-(4-(4-(tert-butyl)phenyl)-1-phenethyl-1H-1,2,3-triazol-5-yl)-10H-dibenzo[b,e][1,4]thiabismine 5,5-dioxide. Synthesized from (2-azidoethyl)benzene and **A2** (0.146 g, 0.25 mmol) following general procedure 6, 0.127 g, 70 %, 2 hours, colorless solid:

A[2]

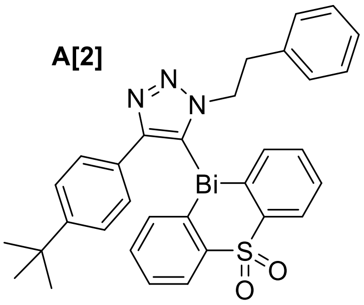

IR (υ[cm-1]) 3062 (vw), 3034 (w), 2950 (m), 2903 (w), 2866 (w), 1561 (w), 1538 (vw), 1494 (w), 1472 (m), 1458 (m), 1442 (m), 1427 (m), 1404 (m), 1365 (m), 1333 (w), 1305 (s), 1289 (m), 1270 (m), 1256 (m), 1228 (w), 1199 (m), 1178 (m), 1153 (m), 1134 (m), 1119 (m), 1106 (m), 1088 (m), 1074 (m), 1051 (w), 1027 (m), 1011 (m), 983 (m), 912 (w), 889 (w), 878 (w), 842 (m), 764 (m), 740 (s), 715 (m), 697 (s), 637 (m), 585 (vs), 576 (s), 565 (vs), 518 (m), 509 (m), 501 (m), 461 (m), 426 (w).

^{1}H NMR (500 MHz, Chloroform-d) δ 8.40 (d, J = 7.7 Hz, 2H), 7.85 (s, 2H), 7.37 (t, J = 7.5 Hz, 2H), 7.29-7.27 (m, 2H), 7.12 (s, 6H), 6.55 (s, 2H), 3.82 (s, 2H), 2.97 (s, 2H), 1.28 (s, 9H).

^{13}C NMR (126 MHz, Chloroform-d) δ 156.95, 156.59, 152.39, 150.98, 141.04, 137.88, 137.17, 133.90, 129.32, 129.22, 128.60, 128.45, 127.93, 127.67, 126.91, 125.24, 77.02, 53.21, 36.49, 34.52, 31.26.

10-(1-phenethyl-4-(p-tolyl)-1H-1,2,3-triazol-5-yl)-10H-dibenzo[b,e][1,4]thiabismine 5,5-dioxide. Synthesized from (2-azidoethyl)benzene and **A[3]** (0.135, 0.25 mmol) following general procedure 6, 0.121 g, 71 %, 4 hours, colorless solid:

P[3]

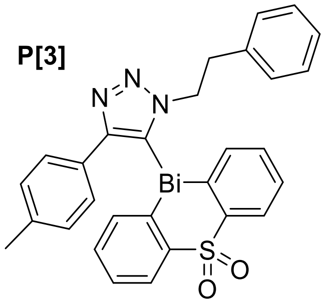

IR (υ[cm-1]) 3060 (vw), 3033 (w), 2960 (w), 2921 (w), 2852 (w), 1561 (w), 1538 (vw), 1497 (w), 1457 (m), 1425 (m), 1409 (w), 1397 (m), 1332 (vw), 1308 (m), 1289 (m), 1253 (m), 1194 (m),

1181 (w), 1151 (s), 1135 (m), 1120 (m), 1107 (m), 1087 (m), 1073 (m), 1029 (m), 1011 (m), 984 (m), 964 (w), 949 (w), 909 (w), 900 (w), 876 (w), 839 (w), 823 (m), 797 (w), 780 (m), 759 (m), 733 (s), 716 (m), 694 (s), 669 (w), 636 (m), 612 (w), 587 (vs), 564 (vs), 542 (m), 510 (m), 479 (w), 462 (m), 425 (w), 408 (w).

^{1}H NMR (500 MHz, Chloroform-d) δ 8.54 (s, 2H), 8.00 (s, 2H), 7.67 – 7.28 (m, 7H), 7.19 – 6.22 (m, 6H), 3.95 (s, 2H), 3.09 (s, 2H), 2.43 (s, 3H).

^{13}C NMR (126 MHz, Chloroform-d) δ 157.28, 156.71, 152.80, 141.05, 138.01, 137.88, 137.23, 133.98, 129.45, 129.13, 128.64, 128.50, 128.22, 127.71, 126.95, 77.37, 77.16, 53.25, 36.59, 21.20.

10-(1-phenethyl-4-phenyl-1H-1,2,3-triazol-5-yl)-10H-dibenzo[b,e][1,4]thiabismine 5,5-dioxide. Synthesized from (2-azidoethyl)benzene and **A[4]** (0.138 g, 0.25 mmol) following general procedure 6, 0.10 g, 59 %, 4 hours, colorless solid:

P[4]

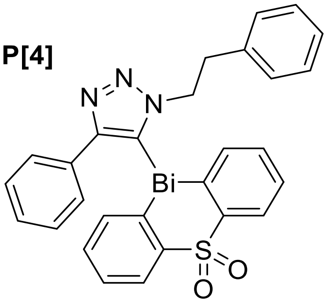

IR (υ[cm-1]) 3062 (w), 3026 (w), 2950 (w), 1605 (w), 1581 (w), 1562 (w), 1497 (w), 1483 (w), 1447 (m), 1427 (m), 1395 (w), 1372 (w), 1298 (s), 1286 (m), 1251 (m), 1223 (m), 1179 (m), 1151 (s), 1106 (m), 1087 (m), 1073 (m), 1050 (m), 1026 (m), 1013 (m), 999 (m), 977 (m), 912 (w), 845 (w), 760 (s), 727 (s), 714 (m), 697 (vs), 690 (vs), 637 (m), 586 (vs), 562 (vs), 517 (m), 508 (m), 462 (m), 427 (w), 404 (vw).

^{1}H NMR (500 MHz, Chloroform-d) δ 8.53 (d, J = 7.8 Hz, 2H), 8.00 (s, 2H), 7.51 (t, J = 8.0 Hz, 2H), 7.41 (s, 3H), 7.36-7.11 (m, 6H), 6.70 (s, 3H), 3.98 (s, 2H), 3.11 (s, 2H).

^{13}C NMR (126 MHz, Chloroform-d) δ 157.20, 156.67, 152.74, 141.10, 137.99, 137.24, 134.06, 132.37, 129.32, 128.71, 128.56, 128.40, 128.33, 128.08, 127.80, 127.03, 77.16, 53.34, 36.66.

10-(4-(4-bromophenyl)-1-phenethyl-1H-1,2,3-triazol-5-yl)-10H-dibenzo[b,e][1,4]thiabismine 5,5-dioxide. Synthesized from (2-azidoethyl)benzene and **A[5]** (0.151 g, 0.25 mmol) following general procedure 6, 0.075 g, 40 %, 2 hours, pale-yellow solid:

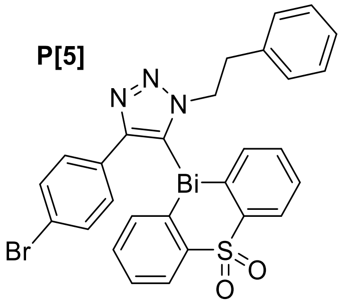

IR (υ[cm-1]) 3118 (vw), 3091 (vw), 3055 (w), 2918 (vw), 2855 (vw), 1599 (w), 1564 (w), 1498 (w), 1481 (w), 1454 (m), 1402 (w), 1364 (w), 1334 (w), 1301 (m), 1288 (m), 1254 (m), 1222 (m), 1178 (w), 1148 (s), 1133 (m), 1117 (m), 1106 (m), 1088 (m), 1070 (m), 1051 (m), 1028 (m), 1011 (m), 977 (m), 838 (m), 824 (m), 756 (m), 739 (s), 712 (m), 697 (s), 637 (m), 584 (vs), 564 (vs), 507 (m), 480 (m), 461 (m), 443 (m), 423 (w).

^{1}H NMR (500 MHz, Chloroform-d) δ 8.38 (d, J = 7.7 Hz, 2H), 7.68 (s, 2H), 7.38 (t, J = 7.4 Hz, 2H), 7.23 – 7.15 (m, 4H), 7.08 – 6.42 (m, 7H), 4.33 (s, 2H), 3.18 (s, 2H).

13C NMR (126 MHz, Chloroform-d) δ 157.26, 154.98, 152.45, 141.67, 140.85, 137.41, 134.01, 131.28, 131.04, 129.53, 129.29, 129.05, 128.97, 127.91, 127.45, 121.99, 77.16, 53.75, 37.28.

10-(1-phenethyl-4-(4-(trifluoromethyl)phenyl)-1H-1,2,3-triazol-5-yl)-10H-dibenzo[b,e][1,4]thiabismine 5,5-dioxide. Synthesized from (2-azidoethyl)benzene and **A[6]** (0.297 g, 0.5 mmol) following general procedure 5, 0.298 g, 80 %, 3 hours, colorless solid:

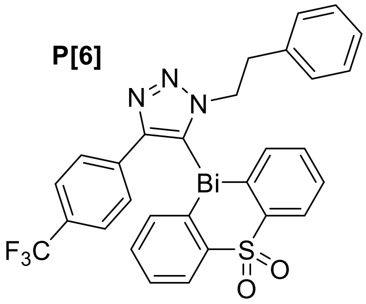

IR (υ[cm-1]) 3036 (w), 2941 (w), 2873 (vw), 1618 (w), 1562 (w), 1493 (w), 1457 (w), 1436 (w), 1427 (w), 1413 (w), 1323 (s), 1310 (s), 1256 (m), 1196 (w), 1168 (m), 1152 (s), 1134 (s), 1106 (s), 1088 (s), 1063 (s), 1029 (m), 1011 (m), 983 (m), 915 (w), 878 (w), 850 (m), 781 (w), 761 (m), 737 (s), 714 (m), 699 (s), 665 (m), 636 (m), 608 (w), 586 (vs), 564 (vs), 509 (m), 488 (w), 463 (m), 452 (m), 425 (w).

^{1}H NMR (600 MHz, Chloroform-d) δ 8.33 (d, J = 7.8 Hz, 2H), 7.71 – 7.55 (m, 2H), 7.30 (t, J = 7.6 Hz, 2H), 7.20 (t, J = 7.4 Hz, 2H), 7.15 (t, J = 7.3 Hz, 3H), 7.13 – 7.06 (m, 6H), 4.58 (s, 2H), 3.23 (s, 2H).

^{13}C NMR (126 MHz, Chloroform-d) δ 157.65, 154.29, 152.81, 140.70, 137.98, 137.40, 135.87, 133.89, 129.29, 129.05, 127.99, 127.74, 127.47, 127.29 (q, J=4.9 Hz), 124.53, 123.94 (q, J=272.1 Hz), 77.16, 53.78, 37.35.

^{19}F NMR (470 MHz, Chloroform-d) δ -63.00.

4. X-Ray Crystallographic Details

X-Ray Crystallographic Analysis of bismuth(III) acetylide A[1]

A clear colourless blade-like specimen of $C_{21}H_{15}BiO_3S$, approximate dimensions 0.016 mm x 0.143 mm x 0.226 mm, was used for the X-ray crystallographic analysis. The X-ray intensity data were measured on a Bruker APEX DUO system equipped with a TRIUMPH curved-crystal monochromator and a MoKα fine-focus tube (λ = 0.71073 Å).
The total exposure time was 14.00 hours. The frames were integrated with the Bruker SAINT software package using a SAINT V8.34A (Bruker AXS, 2013) algorithm. The integration of the data using a triclinic unit cell yielded a total of 22944 reflections to a maximum θ angle of 31.44° (0.68 Å resolution), of which 5725 were independent (average redundancy 4.008, completeness = 93.6%, R_{int} = 3.31%, R_{sig} = 3.01%) and 5300 (92.58%) were greater than $2\sigma(F^2)$. The final cell constants of a = 8.5311(12) Å, b = 9.7298(13) Å, c = 12.6454(17) Å, α = 69.541(2)°, β = 75.212(2)°, γ = 71.666(2)°, volume = 921.0(2) $Å^3$, are based upon the refinement of the XYZ-centroids of 9959 reflections above 20 σ(I) with 4.613° < 2θ < 62.75°. Data were corrected for absorption effects using the multi-scan method (SADABS). The ratio of minimum to maximum apparent transmission was 0.738. The calculated minimum and maximum transmission coefficients (based on crystal size) are 0.2180 and 0.8600.

The structure was solved and refined using the Bruker SHELXTL Software Package, using the space group *P*-1, with Z = 2 for the formula unit, $C_{21}H_{15}BiO_3S$. The final anisotropic full-matrix least-squares refinement on F^2 with 236 variables converged at R1 = 1.92%, for the observed data and wR2 = 4.28% for all data. The goodness-of-fit was 1.057. The largest peak in the final difference electron density synthesis was 1.128 e-/$Å^3$ and the largest hole was -1.282 e-/$Å^3$ with an RMS deviation of 0.131 e-/$Å^3$. On the basis of the final model, the calculated density was 2.006 g/cm3 and F(000), 528 e-.

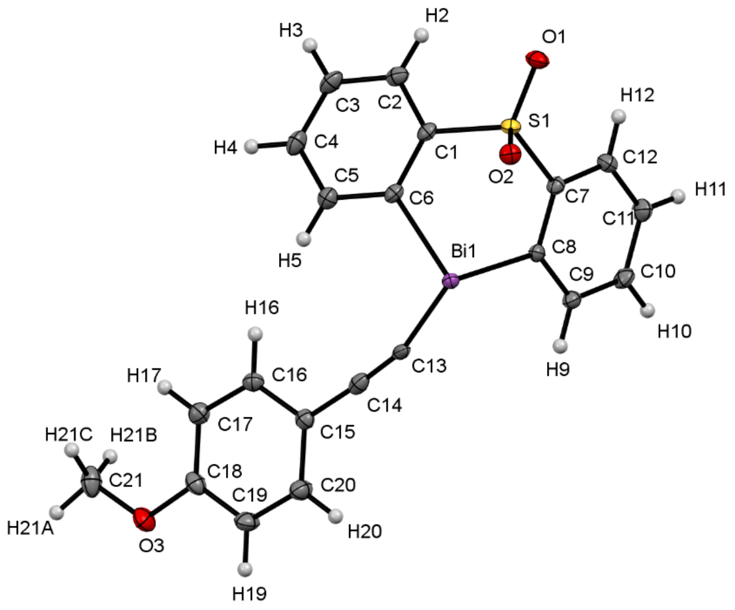

Figure S.1 Asymmetric unit in the crystal structure of compound A[1].

Table S.10 Crystal data and structure refinement for compound A[1].

Chemical formula	C21H15BiO3S
Formula weight	556.37 g/mol
Temperature	100(2) K
Wavelength	0.71073 Å
Crystal size	0.016 x 0.143 x 0.226 mm
Crystal habit	clear colourless blade
Crystal system	triclinic
Space group	*P* -1
Unit cell dimensions	a = 8.5311(12) Å α = 69.541(2)°
	b = 9.7298(13) Å β = 75.212(2)°
	c = 12.6454(17) Å γ = 71.666(2)°
Volume	921.0(2) Å3
Z	2
Density (calculated)	2.006 g/cm3
Absorption coefficient	9.703 mm-1
F(000)	528
Diffractometer	Bruker APEX DUO
Radiation source	fine-focus tube, MoKα
Theta range for data collection	1.74 to 31.44°
Index ranges	-12<=h<=12, -13<=k<=14, -18<=l<=18
Reflections collected	22944
Independent reflections	5725 [R(int) = 0.0331]
Coverage of independent reflections	93.6%
Absorption correction	multi-scan
Max. and min. transmission	0.8600 and 0.2180
Structure solution technique	direct methods
Structure solution program	SHELXTL XT 2014/4 (Bruker AXS, 2014)
Refinement method	Full-matrix least-squares on F2
Refinement program	SHELXTL XL 2014/7 (Bruker AXS, 2014)
Function minimized	Σ w(Fo2 - Fc2)2
Data / restraints / parameters	5725 / 0 / 236
Goodness-of-fit on F2	1.057
Δ/σmax	0.001
Final R indices	5300 data; I>2σ(I) R1 = 0.0192, wR2 = 0.0417
	all data R1 = 0.0229, wR2 = 0.0428
Weighting scheme	w=1/[σ2(Fo2)+(0.0199P)2+0.2424P]
where P=(Fo2+2Fc2)/3	
Largest diff. peak and hole	1.128 and -1.282 eÅ-3
R.M.S. deviation from mean	0.131 eÅ-3

Table S.11 Bond lengths [Å] and angles [°] for compound A[7].

C1-C2	1.391(3)	C1-C6	1.395(3)
C1-S1	1.768(2)	C2-C3	1.391(4)
C2-H2	0.95	C3-C4	1.392(4)
C3-H3	0.95	C4-C5	1.396(3)

C4-H4	0.95	C5-C6	1.393(3)
C5-H5	0.95	C6-Bi1	2.277(2)
C7-C12	1.388(3)	C7-C8	1.393(3)
C7-S1	1.773(2)	C8-C9	1.398(3)
C8-Bi1	2.287(2)	C9-C10	1.394(4)
C9-H9	0.95	C10-C11	1.396(4)
C10-H10	0.95	C11-C12	1.387(3)
C11-H11	0.95	C12-H12	0.95
C13-C14	1.180(4)	C13-Bi1	2.243(2)
C14-C15	1.453(3)	C15-C16	1.396(3)
C15-C20	1.406(3)	C16-C17	1.388(3)
C16-H16	0.95	C17-C18	1.389(4)
C17-H17	0.95	C18-O3	1.368(3)
C18-C19	1.391(4)	C19-C20	1.388(4)
C19-H19	0.95	C20-H20	0.95
C21-O3	1.437(3)	C21-H21A	0.98
C21-H21B	0.98	C21-H21C	0.98
O1-S1	1.4399(18)	O2-S1	1.4586(17)
C2-C1-C6	123.1(2)	C2-C1-S1	119.83(19)
C6-C1-S1	117.05(17)	C1-C2-C3	118.2(2)
C1-C2-H2	120.9	C3-C2-H2	120.9
C2-C3-C4	120.0(2)	C2-C3-H3	120.0
C4-C3-H3	120.0	C3-C4-C5	120.8(2)
C3-C4-H4	119.6	C5-C4-H4	119.6
C6-C5-C4	120.3(2)	C6-C5-H5	119.9
C4-C5-H5	119.9	C5-C6-C1	117.6(2)
C5-C6-Bi1	123.36(18)	C1-C6-Bi1	119.00(17)
C12-C7-C8	123.5(2)	C12-C7-S1	119.31(18)
C8-C7-S1	117.14(18)	C7-C8-C9	117.3(2)
C7-C8-Bi1	118.75(17)	C9-C8-Bi1	123.99(17)
C10-C9-C8	120.2(2)	C10-C9-H9	119.9
C8-C9-H9	119.9	C9-C10-C11	120.9(2)
C9-C10-H10	119.6	C11-C10-H10	119.6
C12-C11-C10	119.9(2)	C12-C11-H11	120.1
C10-C11-H11	120.1	C11-C12-C7	118.2(2)
C11-C12-H12	120.9	C7-C12-H12	120.9
C14-C13-Bi1	160.3(2)	C13-C14-C15	176.5(3)
C16-C15-C20	118.4(2)	C16-C15-C14	119.1(2)
C20-C15-C14	122.4(2)	C17-C16-C15	121.4(2)
C17-C16-H16	119.3	C15-C16-H16	119.3
C16-C17-C18	119.5(2)	C16-C17-H17	120.3
C18-C17-H17	120.3	O3-C18-C17	124.3(2)
O3-C18-C19	115.5(2)	C17-C18-C19	120.2(2)
C20-C19-C18	120.2(2)	C20-C19-H19	119.9
C18-C19-H19	119.9	C19-C20-C15	120.3(2)
C19-C20-H20	119.8	C15-C20-H20	119.8
O3-C21-H21A	109.5	O3-C21-H21B	109.5

H21A-C21-H21B	109.5	O3-C21-H21C	109.5
H21A-C21-H21C	109.5	H21B-C21-H21C	109.5
C18-O3-C21	117.0(2)	O1-S1-O2	118.52(11)
O1-S1-C1	110.79(11)	O2-S1-C1	106.15(11)
O1-S1-C7	110.19(11)	O2-S1-C7	105.66(11)
C1-S1-C7	104.51(11)	C13-Bi1-C6	89.61(8)
C13-Bi1-C8	94.75(8)	C6-Bi1-C8	85.94(8)

X-Ray Crystallographic Analysis of bismuth(III) acetylide A[2]

The single-crystal X-ray diffraction studies were carried out on a Bruker Kappa Apex II CCD diffractometer equipped with Mo K_{α} radiation (λ = 0.71073). A 0.180 x 0.125 x 0.060 mm colorless crystal was mounted on a Cryoloop with Paratone oil. Data were collected in a nitrogen gas stream at 100(2) K using ϕ and ϖ scans. Crystal-to-detector distance was 40 mm using exposure time 15s with a scan width of 0.60°. Data collection was 100.0% complete to 25.242° in θ. A total of 24144 reflections were collected covering the indices, -12<=h<=12, -10<=k<=10, -30<=l<=30. 4322 reflections were found to be symmetry independent, with a Rint of 0.0350. Indexing and unit cell refinement indicated a Primitive, Monoclinic lattice. The space group was found to be P21/c. The data were integrated using the Bruker SAINT Software program and scaled using the SADABS software program. Solution by direct methods (SHELXT) produced a complete phasing model consistent with the proposed structure. All nonhydrogen atoms were refined anisotropically by full-matrix least-squares (SHELXL-2014). All carbon bonded hydrogen atoms were placed using a riding model. Their positions were constrained relative to their parent atom using the appropriate HFIX command in SHELXL-2014.

Notes: Proposed structure in agreement with model derived from diffraction data. Excellent data and refinement Disorder on the t-Bu group, SADI, RIGU…

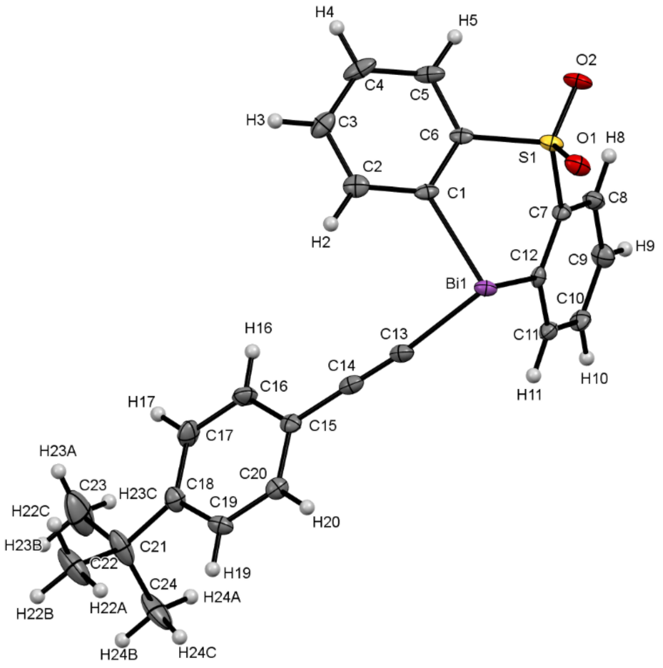

Figure S.11 Asymmetric unit in the crystal structure of compound A[2].

Table S.12 Crystal data and structure refinement for A[2].

Empirical formula	C24 H21 Bi O2 S
Molecular formula	C24 H21 Bi O2 S
Formula weight	582.45
Temperature	100.0 K
Wavelength	0.71073 Å

Crystal system	Monoclinic
Space group	P 1 21/c 1
Unit cell dimensions	a = 10.2166(5) Å □□ 90°.
	b = 8.6731(4) Å □□□97.570(2)°.
	c = 24.1194(11) Å □□□ 90°.
Volume	2118.58(17) Å^3
Z	4
Density (calculated)	1.826 Mg/m^3
Absorption coefficient	8.437 mm^{-1}
F(000)	1120
Crystal size	0.18 x 0.125 x 0.06 mm^3
Crystal color, habit	colorless plank
Theta range for data collection	2.011 to 26.371°.
Index ranges	-12<=h<=12, -10<=k<=10, -30<=l<=30
Reflections collected	24144
Independent reflections	4322 [R(int) = 0.0350]
Completeness to theta = 25.242°	100.0 %
Absorption correction	Semi-empirical from equivalents
Max. and min. transmission	0.2607 and 0.1603
Refinement method	Full-matrix least-squares on F^2
Data / restraints / parameters	4322 / 76 / 296
Goodness-of-fit on F^2	1.028
Final R indices [I>2sigma(I)]	R1 = 0.0198, wR2 = 0.0336
R indices (all data)	R1 = 0.0321, wR2 = 0.0364
Largest diff. peak and hole	0.481 and -0.415 e.Å^{-3}

Table S.13 Bond lengths [Å] and angles [°] for A[2].

Bi(1)-C(1)	2.277(3)
Bi(1)-C(12)	2.274(3)
Bi(1)-C(13)	2.225(3)
S(1)-O(1)	1.453(2)
S(1)-O(2)	1.440(2)
S(1)-C(6)	1.769(3)
S(1)-C(7)	1.766(3)
C(1)-C(2)	1.389(4)
C(1)-C(6)	1.390(4)
C(2)-H(2)	0.9500
C(2)-C(3)	1.391(4)
C(3)-H(3)	0.9500
C(3)-C(4)	1.392(5)
C(4)-H(4)	0.9500
C(4)-C(5)	1.382(5)
C(5)-H(5)	0.9500
C(5)-C(6)	1.391(4)
C(7)-C(8)	1.387(4)

C(7)-C(12)	1.399(4)
C(8)-H(8)	0.9500
C(8)-C(9)	1.390(4)
C(9)-H(9)	0.9500
C(9)-C(10)	1.389(4)
C(10)-H(10)	0.9500
C(10)-C(11)	1.387(4)
C(11)-H(11)	0.9500
C(11)-C(12)	1.385(4)
C(13)-C(14)	1.195(4)
C(14)-C(15)	1.453(4)
C(15)-C(16)	1.397(4)
C(15)-C(20)	1.395(4)
C(16)-H(16)	0.9500
C(16)-C(17)	1.384(4)
C(17)-H(17)	0.9500
C(17)-C(18)	1.399(5)
C(18)-C(19)	1.400(4)
C(18)-C(21)	1.535(7)
C(18)-C(21B)	1.531(7)
C(19)-H(19)	0.9500
C(19)-C(20)	1.382(4)
C(20)-H(20)	0.9500
C(21)-C(22)	1.533(7)
C(21)-C(23)	1.526(7)
C(21)-C(24)	1.535(7)
C(22)-H(22A)	0.9800
C(22)-H(22B)	0.9800
C(22)-H(22C)	0.9800
C(23)-H(23A)	0.9800
C(23)-H(23B)	0.9800
C(23)-H(23C)	0.9800
C(24)-H(24A)	0.9800
C(24)-H(24B)	0.9800
C(24)-H(24C)	0.9800
C(21B)-C(22B)	1.538(7)
C(21B)-C(23B)	1.529(7)
C(21B)-C(24B)	1.530(7)
C(22B)-H(22D)	0.9800
C(22B)-H(22E)	0.9800
C(22B)-H(22F)	0.9800
C(23B)-H(23D)	0.9800
C(23B)-H(23E)	0.9800
C(23B)-H(23F)	0.9800
C(24B)-H(24D)	0.9800
C(24B)-H(24E)	0.9800
C(24B)-H(24F)	0.9800

C(12)-Bi(1)-C(1)	85.87(11)
C(13)-Bi(1)-C(1)	90.39(11)
C(13)-Bi(1)-C(12)	93.72(11)
O(1)-S(1)-C(6)	105.64(14)
O(1)-S(1)-C(7)	106.87(14)
O(2)-S(1)-O(1)	119.31(13)
O(2)-S(1)-C(6)	109.76(14)
O(2)-S(1)-C(7)	109.92(14)
C(7)-S(1)-C(6)	104.24(14)
C(2)-C(1)-Bi(1)	122.4(2)
C(2)-C(1)-C(6)	118.0(3)
C(6)-C(1)-Bi(1)	119.5(2)
C(1)-C(2)-H(2)	119.9
C(1)-C(2)-C(3)	120.2(3)
C(3)-C(2)-H(2)	119.9
C(2)-C(3)-H(3)	119.8
C(2)-C(3)-C(4)	120.3(3)
C(4)-C(3)-H(3)	119.8
C(3)-C(4)-H(4)	119.7
C(5)-C(4)-C(3)	120.6(3)
C(5)-C(4)-H(4)	119.7
C(4)-C(5)-H(5)	121.0
C(4)-C(5)-C(6)	118.0(3)
C(6)-C(5)-H(5)	121.0
C(1)-C(6)-S(1)	118.0(2)
C(1)-C(6)-C(5)	122.8(3)
C(5)-C(6)-S(1)	119.1(2)
C(8)-C(7)-S(1)	118.8(2)
C(8)-C(7)-C(12)	122.6(3)
C(12)-C(7)-S(1)	118.6(2)
C(7)-C(8)-H(8)	120.8
C(7)-C(8)-C(9)	118.3(3)
C(9)-C(8)-H(8)	120.8
C(8)-C(9)-H(9)	119.9
C(10)-C(9)-C(8)	120.2(3)
C(10)-C(9)-H(9)	119.9
C(9)-C(10)-H(10)	119.8
C(11)-C(10)-C(9)	120.3(3)
C(11)-C(10)-H(10)	119.8
C(10)-C(11)-H(11)	119.5
C(12)-C(11)-C(10)	120.9(3)
C(12)-C(11)-H(11)	119.5
C(7)-C(12)-Bi(1)	118.8(2)
C(11)-C(12)-Bi(1)	123.6(2)
C(11)-C(12)-C(7)	117.6(3)
C(14)-C(13)-Bi(1)	170.3(3)
C(13)-C(14)-C(15)	178.1(4)
C(16)-C(15)-C(14)	120.1(3)

C(20)-C(15)-C(14)	121.8(3)
C(20)-C(15)-C(16)	118.1(3)
C(15)-C(16)-H(16)	119.7
C(17)-C(16)-C(15)	120.6(3)
C(17)-C(16)-H(16)	119.7
C(16)-C(17)-H(17)	119.0
C(16)-C(17)-C(18)	121.9(3)
C(18)-C(17)-H(17)	119.0
C(17)-C(18)-C(19)	116.7(3)
C(17)-C(18)-C(21)	123.6(5)
C(17)-C(18)-C(21B)	120.7(5)
C(19)-C(18)-C(21)	118.9(5)
C(19)-C(18)-C(21B)	122.5(5)
C(18)-C(19)-H(19)	119.1
C(20)-C(19)-C(18)	121.9(3)
C(20)-C(19)-H(19)	119.1
C(15)-C(20)-H(20)	119.6
C(19)-C(20)-C(15)	120.8(3)
C(19)-C(20)-H(20)	119.6
C(22)-C(21)-C(18)	104.6(13)
C(22)-C(21)-C(24)	112.1(13)
C(23)-C(21)-C(18)	112.4(7)
C(23)-C(21)-C(22)	108.5(11)
C(23)-C(21)-C(24)	108.7(8)
C(24)-C(21)-C(18)	110.6(8)
C(21)-C(22)-H(22A)	109.5
C(21)-C(22)-H(22B)	109.5
C(21)-C(22)-H(22C)	109.5
H(22A)-C(22)-H(22B)	109.5
H(22A)-C(22)-H(22C)	109.5
H(22B)-C(22)-H(22C)	109.5
C(21)-C(23)-H(23A)	109.5
C(21)-C(23)-H(23B)	109.5
C(21)-C(23)-H(23C)	109.5
H(23A)-C(23)-H(23B)	109.5
H(23A)-C(23)-H(23C)	109.5
H(23B)-C(23)-H(23C)	109.5
C(21)-C(24)-H(24A)	109.5
C(21)-C(24)-H(24B)	109.5
C(21)-C(24)-H(24C)	109.5
H(24A)-C(24)-H(24B)	109.5
H(24A)-C(24)-H(24C)	109.5
H(24B)-C(24)-H(24C)	109.5
C(18)-C(21B)-C(22B)	114.7(15)
C(23B)-C(21B)-C(18)	113.1(8)
C(23B)-C(21B)-C(22B)	109.7(12)
C(23B)-C(21B)-C(24B)	108.0(9)
C(24B)-C(21B)-C(18)	105.2(8)

C(24B)-C(21B)-C(22B)	105.5(14)
C(21B)-C(22B)-H(22D)	109.5
C(21B)-C(22B)-H(22E)	109.5
C(21B)-C(22B)-H(22F)	109.5
H(22D)-C(22B)-H(22E)	109.5
H(22D)-C(22B)-H(22F)	109.5
H(22E)-C(22B)-H(22F)	109.5
C(21B)-C(23B)-H(23D)	109.5
C(21B)-C(23B)-H(23E)	109.5
C(21B)-C(23B)-H(23F)	109.5
H(23D)-C(23B)-H(23E)	109.5
H(23D)-C(23B)-H(23F)	109.5
H(23E)-C(23B)-H(23F)	109.5
C(21B)-C(24B)-H(24D)	109.5
C(21B)-C(24B)-H(24E)	109.5
C(21B)-C(24B)-H(24F)	109.5
H(24D)-C(24B)-H(24E)	109.5
H(24D)-C(24B)-H(24F)	109.5
H(24E)-C(24B)-H(24F)	109.5

X-Ray Crystallographic Analysis of bismuth(III) acetylide A[3]

The single-crystal X-ray diffraction studies were carried out on a Bruker APEX II ULTRA CCD diffractometer equipped with Mo Kα radiation (λ = 0.71073 Å). Crystals of the subject compound were used as received. A 0.220 x 0.160 x 0.110 mm colorless block crystal was mounted on a Cryoloop with Paratone N oil. Data were collected in a nitrogen gas stream at 100(2) K using ϕ and ϖ scans. Crystal-to-detector distance was 40 mm using an exposure time of 5 seconds with a scan width of 1.5°. Data collection was 99.9% complete to 25.242° in θ. A total of 11108 reflections were collected. 4447 reflections were found to be symmetry independent, with a Rint of 0.0314. Indexing and unit cell refinement indicated a Primitive Triclinic lattice. The space group was found to be P-1. The data were integrated using the Bruker SAINT Software program and scaled using the SADABS software program. Solution by direct methods (SHELXT) produced a complete phasing model consistent with the proposed structure. All non-hydrogen atoms were refined anisotropically by full-matrix least-squares (SHELXL-2014). All carbon bonded hydrogen atoms were placed using a riding model. Their positions were constrained relative to their parent atom using the appropriate HFIX command in SHELXL-2014.
Notes: Excellent data and refinement. There is one molecule per asymmetric unit.

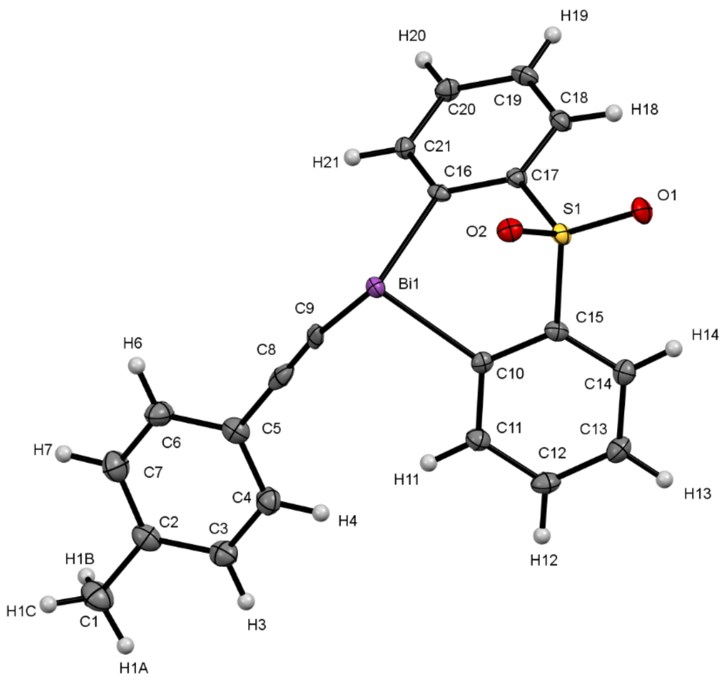

Figure S.2 Asymmetric unit in the crystal structure of compound A[3].

Table S.14 Crystal data and structure refinement for compound A[3].

Empirical formula	C21 H15 Bi O2 S
Formula weight	540.37
Temperature	100.0 K
Wavelength	0.71073 Å
Crystal system	Triclinic
Space group	P-1

Unit cell dimensions	a = 8.4656(5) Å α= 70.606(2)°.
	b = 9.4514(5) Å β= 87.545(2)°.
	c = 12.2813(6) Å γ= 75.602(2)°.
Volume	896.93(8) Å3
Z	2
Density (calculated)	2.001 Mg/m3
Absorption coefficient	9.956 mm-1
F(000)	512
Crystal size	0.22 x 0.16 x 0.11 mm3
Theta range for data collection	1.760 to 28.292°.
Index ranges	-11<=h<=11, -12<=k<=12, -16<=l<=16
Reflections collected	11108
Independent reflections	4447 [R(int) = 0.0314]
Completeness to theta = 25.242°	99.9 %
Absorption correction	Semi-empirical from equivalents
Max. and min. transmission	0.7457 and 0.5814
Refinement method	Full-matrix least-squares on F2
Data / restraints / parameters	4447 / 0 / 227
Goodness-of-fit on F2	1.038
Final R indices [I>2sigma(I)]	R1 = 0.0205, wR2 = 0.0463
R indices (all data)	R1 = 0.0242, wR2 = 0.0472
Extinction coefficient	n/a
Largest diff. peak and hole	1.429 and -0.802 e.Å-3

Table S.15 Bond lengths [Å] and angles [°] for compound A[3].

Bi(1)-C(9)	2.256(3)
Bi(1)-C(10)	2.274(3)
Bi(1)-C(16)	2.275(3)
S(1)-O(1)	1.445(2)
S(1)-O(2)	1.459(2)
S(1)-C(15)	1.767(3)
S(1)-C(17)	1.771(3)
C(1)-H(1A)	0.9800
C(1)-H(1B)	0.9800
C(1)-H(1C)	0.9800
C(1)-C(2)	1.520(4)
C(2)-C(3)	1.391(5)
C(2)-C(7)	1.391(5)
C(3)-H(3)	0.9500
C(3)-C(4)	1.387(4)
C(4)-H(4)	0.9500
C(4)-C(5)	1.400(5)
C(5)-C(6)	1.396(5)
C(5)-C(8)	1.483(5)
C(6)-H(6)	0.9500
C(6)-C(7)	1.377(4)

C(7)-H(7)	0.9500
C(8)-C(9)	1.135(5)
C(10)-C(11)	1.391(4)
C(10)-C(15)	1.399(4)
C(11)-H(11)	0.9500
C(11)-C(12)	1.397(5)
C(12)-H(12)	0.9500
C(12)-C(13)	1.392(4)
C(13)-H(13)	0.9500
C(13)-C(14)	1.392(4)
C(14)-H(14)	0.9500
C(14)-C(15)	1.381(4)
C(16)-C(17)	1.390(4)
C(16)-C(21)	1.399(4)
C(17)-C(18)	1.393(4)
C(18)-H(18)	0.9500
C(18)-C(19)	1.384(4)
C(19)-H(19)	0.9500
C(19)-C(20)	1.395(4)
C(20)-H(20)	0.9500
C(20)-C(21)	1.395(4)
C(21)-H(21)	0.9500
C(9)-Bi(1)-C(10)	91.74(11)
C(9)-Bi(1)-C(16)	94.53(11)
C(10)-Bi(1)-C(16)	86.35(11)
O(1)-S(1)-O(2)	118.94(13)
O(1)-S(1)-C(15)	110.15(14)
O(1)-S(1)-C(17)	109.64(14)
O(2)-S(1)-C(15)	106.19(14)
O(2)-S(1)-C(17)	106.05(14)
C(15)-S(1)-C(17)	104.92(14)
H(1A)-C(1)-H(1B)	109.5
H(1A)-C(1)-H(1C)	109.5
H(1B)-C(1)-H(1C)	109.5
C(2)-C(1)-H(1A)	109.5
C(2)-C(1)-H(1B)	109.5
C(2)-C(1)-H(1C)	109.5
C(3)-C(2)-C(1)	121.4(3)
C(7)-C(2)-C(1)	120.6(3)
C(7)-C(2)-C(3)	117.9(3)
C(2)-C(3)-H(3)	119.2
C(4)-C(3)-C(2)	121.6(3)
C(4)-C(3)-H(3)	119.2
C(3)-C(4)-H(4)	120.0
C(3)-C(4)-C(5)	119.9(3)
C(5)-C(4)-H(4)	120.0
C(4)-C(5)-C(8)	120.2(3)

C(6)-C(5)-C(4)	118.5(3)
C(6)-C(5)-C(8)	121.2(3)
C(5)-C(6)-H(6)	119.6
C(7)-C(6)-C(5)	120.8(3)
C(7)-C(6)-H(6)	119.6
C(2)-C(7)-H(7)	119.4
C(6)-C(7)-C(2)	121.2(3)
C(6)-C(7)-H(7)	119.4
C(9)-C(8)-C(5)	178.3(4)
C(8)-C(9)-Bi(1)	161.7(3)
C(11)-C(10)-Bi(1)	123.0(2)
C(11)-C(10)-C(15)	117.3(3)
C(15)-C(10)-Bi(1)	119.7(2)
C(10)-C(11)-H(11)	119.8
C(10)-C(11)-C(12)	120.5(3)
C(12)-C(11)-H(11)	119.8
C(11)-C(12)-H(12)	119.6
C(13)-C(12)-C(11)	120.9(3)
C(13)-C(12)-H(12)	119.6
C(12)-C(13)-H(13)	120.2
C(12)-C(13)-C(14)	119.5(3)
C(14)-C(13)-H(13)	120.2
C(13)-C(14)-H(14)	120.7
C(15)-C(14)-C(13)	118.6(3)
C(15)-C(14)-H(14)	120.7
C(10)-C(15)-S(1)	116.9(2)
C(14)-C(15)-S(1)	119.8(2)
C(14)-C(15)-C(10)	123.3(3)
C(17)-C(16)-Bi(1)	118.9(2)
C(17)-C(16)-C(21)	117.3(3)
C(21)-C(16)-Bi(1)	123.8(2)
C(16)-C(17)-S(1)	118.1(2)
C(16)-C(17)-C(18)	123.2(3)
C(18)-C(17)-S(1)	118.7(2)
C(17)-C(18)-H(18)	120.8
C(19)-C(18)-C(17)	118.4(3)
C(19)-C(18)-H(18)	120.8
C(18)-C(19)-H(19)	120.0
C(18)-C(19)-C(20)	120.0(3)
C(20)-C(19)-H(19)	120.0
C(19)-C(20)-H(20)	119.7
C(19)-C(20)-C(21)	120.6(3)
C(21)-C(20)-H(20)	119.7
C(16)-C(21)-H(21)	119.8
C(20)-C(21)-C(16)	120.5(3)
C(20)-C(21)-H(21)	119.8

X-Ray Crystallographic Analysis of bismuth(III) acetylide A[4]

The single-crystal X-ray diffraction studies were carried out on a Bruker Kappa Apex II CCD diffractometer equipped with Mo Kα radiation (λ = 0.71073). A 0.120 x 0.050 x 0.040 mm colorless crystal was mounted on a Cryoloop with Paratone oil. Data were collected in a nitrogen gas stream at 100(2) K using ϕ and ϖ scans. Crystal-to-detector distance was 40 mm using exposure time 20s with a scan width of 0.70°. Data collection was 100.0% complete to 25.242° in A total of 26572 reflections were collected covering the indices, -10<=h<=10, -12<=k<=12, -25<=l<=25. 3488 reflections were found to be symmetry independent, with a R_{int} of 0.0413. Indexing and unit cell refinement indicated a Primitive, Monoclinic lattice. The space group was found to be $P2_1/c$. The data were integrated using the Bruker SAINT Software program and scaled using the SADABS software program. Solution by direct methods (SHELXT) produced a complete phasing model consistent with the proposed structure. All nonhydrogen atoms were refined anisotropically by full-matrix least-squares (SHELXL-2014). All carbon bonded hydrogen atoms were placed using a riding model. Their positions were constrained relative to their parent atom using the appropriate HFIX command in SHELXL-2014.

Notes: Proposed structure in agreement with model derived from diffraction data. Excellent data and refinement

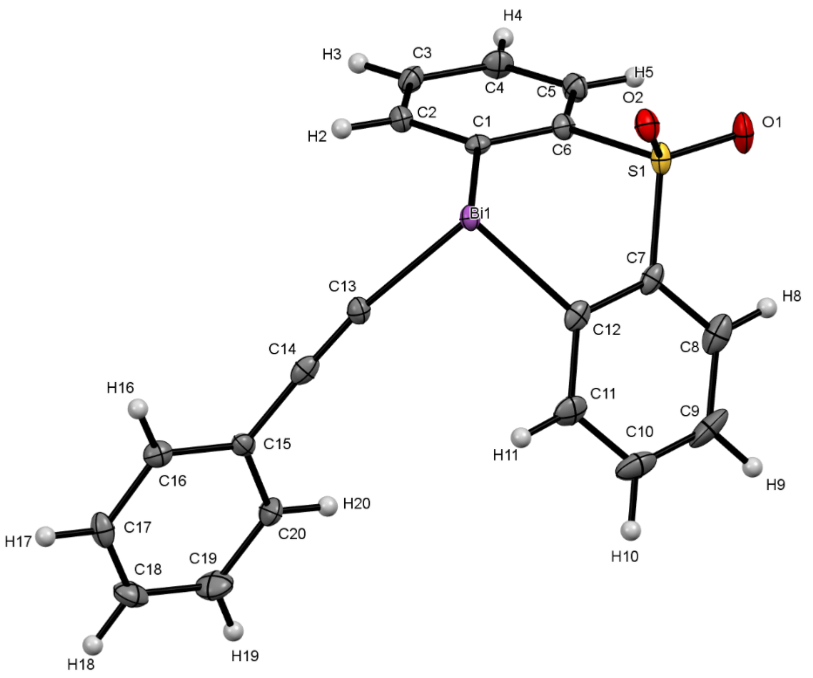

Figure S.3 Asymmetric unit in the crystal structure of compound A[4].

Table S.16 Crystal data and structure refinement for compound A[4].

Empirical formula	C20 H13 Bi O2 S
Molecular formula	C20 H13 Bi O2 S
Formula weight	526.34
Temperature	100.0 K
Wavelength	0.71073 Å
Crystal system	Monoclinic
Space group	P 1 21/c 1

Unit cell dimensions	a = 8.535(4) Å α = 90°.
	b = 9.906(5) Å β = 94.941(17)°.
	c = 20.213(11) Å γ = 90°.
Volume	1702.7(15) Å3
Z	4
Density (calculated)	2.053 Mg/m3
Absorption coefficient	10.486 mm-1
F(000)	992
Crystal size	0.12 x 0.05 x 0.04 mm3
Crystal color, habit	colorless plank
Theta range for data collection	2.023 to 26.359°.
Index ranges	-10<=h<=10, -12<=k<=12, -25<=l<=25
Reflections collected	26572
Independent reflections	3488 [R(int) = 0.0413]
Completeness to theta = 25.242°	100.0 %
Absorption correction	Semi-empirical from equivalents
Max. and min. transmission	0.4910 and 0.3028
Refinement method	Full-matrix least-squares on F2
Data / restraints / parameters	3488 / 0 / 217
Goodness-of-fit on F2	1.029
Final R indices [I>2sigma(I)]	R1 = 0.0172, wR2 = 0.0348
R indices (all data)	R1 = 0.0232, wR2 = 0.0364
Largest diff. peak and hole	0.594 and -0.485 e.Å-3

Table S.17 Bond lengths [Å] and angles [°] for compound A[4].

Bi(1)-C(13)	2.236(3)
Bi(1)-C(1)	2.288(3)
Bi(1)-C(12)	2.290(3)
S(1)-O(2)	1.456(2)
S(1)-O(1)	1.444(2)
S(1)-C(7)	1.766(3)
S(1)-C(6)	1.772(3)
C(13)-C(14)	1.202(4)
C(3)-H(3)	0.9500
C(3)-C(4)	1.395(5)
C(3)-C(2)	1.393(4)
C(1)-C(2)	1.400(4)
C(1)-C(6)	1.396(4)
C(7)-C(12)	1.406(4)
C(7)-C(8)	1.394(4)
C(17)-H(17)	0.9500
C(17)-C(16)	1.402(4)
C(17)-C(18)	1.363(4)
C(14)-C(15)	1.450(4)
C(4)-H(4)	0.9500
C(4)-C(5)	1.381(4)
C(5)-H(5)	0.9500

C(5)-C(6)	1.398(4)
C(2)-H(2)	0.9500
C(20)-H(20)	0.9500
C(20)-C(15)	1.406(4)
C(20)-C(19)	1.379(5)
C(16)-H(16)	0.9500
C(16)-C(15)	1.406(4)
C(12)-C(11)	1.393(5)
C(11)-H(11)	0.9500
C(11)-C(10)	1.395(5)
C(10)-H(10)	0.9500
C(10)-C(9)	1.385(5)
C(8)-H(8)	0.9500
C(8)-C(9)	1.385(5)
C(18)-H(18)	0.9500
C(18)-C(19)	1.380(5)
C(19)-H(19)	0.9500
C(9)-H(9)	0.9500
C(13)-Bi(1)-C(1)	92.23(11)
C(13)-Bi(1)-C(12)	90.07(12)
C(1)-Bi(1)-C(12)	86.04(11)
O(2)-S(1)-C(7)	105.90(14)
O(2)-S(1)-C(6)	106.96(14)
O(1)-S(1)-O(2)	119.21(14)
O(1)-S(1)-C(7)	110.45(15)
O(1)-S(1)-C(6)	109.92(15)
C(7)-S(1)-C(6)	103.15(15)
C(14)-C(13)-Bi(1)	170.0(3)
C(4)-C(3)-H(3)	119.5
C(2)-C(3)-H(3)	119.5
C(2)-C(3)-C(4)	121.0(3)
C(2)-C(1)-Bi(1)	123.6(2)
C(6)-C(1)-Bi(1)	119.4(2)
C(6)-C(1)-C(2)	117.0(3)
C(12)-C(7)-S(1)	117.8(2)
C(8)-C(7)-S(1)	120.1(3)
C(8)-C(7)-C(12)	122.1(3)
C(16)-C(17)-H(17)	119.9
C(18)-C(17)-H(17)	119.9
C(18)-C(17)-C(16)	120.3(3)
C(13)-C(14)-C(15)	177.4(3)
C(3)-C(4)-H(4)	120.0
C(5)-C(4)-C(3)	120.1(3)
C(5)-C(4)-H(4)	120.0
C(4)-C(5)-H(5)	121.0
C(4)-C(5)-C(6)	118.1(3)
C(6)-C(5)-H(5)	121.0

C(3)-C(2)-C(1)	120.4(3)
C(3)-C(2)-H(2)	119.8
C(1)-C(2)-H(2)	119.8
C(15)-C(20)-H(20)	120.2
C(19)-C(20)-H(20)	120.2
C(19)-C(20)-C(15)	119.6(3)
C(17)-C(16)-H(16)	120.1
C(17)-C(16)-C(15)	119.8(3)
C(15)-C(16)-H(16)	120.1
C(7)-C(12)-Bi(1)	118.6(2)
C(11)-C(12)-Bi(1)	123.1(2)
C(11)-C(12)-C(7)	118.2(3)
C(20)-C(15)-C(14)	119.5(3)
C(20)-C(15)-C(16)	118.8(3)
C(16)-C(15)-C(14)	121.6(3)
C(12)-C(11)-H(11)	120.2
C(12)-C(11)-C(10)	119.7(3)
C(10)-C(11)-H(11)	120.2
C(1)-C(6)-S(1)	117.3(2)
C(1)-C(6)-C(5)	123.5(3)
C(5)-C(6)-S(1)	119.2(2)
C(11)-C(10)-H(10)	119.4
C(9)-C(10)-C(11)	121.3(3)
C(9)-C(10)-H(10)	119.4
C(7)-C(8)-H(8)	120.7
C(9)-C(8)-C(7)	118.6(3)
C(9)-C(8)-H(8)	120.7
C(17)-C(18)-H(18)	119.9
C(17)-C(18)-C(19)	120.2(3)
C(19)-C(18)-H(18)	119.9
C(20)-C(19)-C(18)	121.3(3)
C(20)-C(19)-H(19)	119.4
C(18)-C(19)-H(19)	119.4
C(10)-C(9)-H(9)	119.9
C(8)-C(9)-C(10)	120.1(3)
C(8)-C(9)-H(9)	119.9

X-Ray Crystallographic Analysis of bismuth(III) acetylide A[5]

The single-crystal X-ray diffraction studies were carried out on a Bruker APEX II ULTRA CCD diffractometer equipped with Mo Kα radiation (λ= 0.71073 Å). Crystals of the subject compound were used as received. A 0.150 x 0.050 x 0.050 mm colorless prism crystal was mounted on a Cryoloop with Paratone N oil. Data were collected in a nitrogen gas stream at 100(2) K using ϖ scans. Crystal-to-detector distance was 40 mm using an exposure time of 8 seconds with a scan width of 1°. Data collection was 100.0% complete to 25.242° in θ. A total of 23898 reflections were collected. 3274 reflections were found to be symmetry independent, with a Rint of 0.0319. Indexing and unit cell refinement indicated a Primitive Triclinic lattice. The space group was found to be P-1. The data were integrated using the Bruker SAINT Software program and scaled using the SADABS software program. Solution by direct methods (SHELXT) produced a complete phasing model consistent with the proposed structure. All non-hydrogen atoms were refined anisotropically by full-matrix least-squares (SHELXL-2014). All carbon bonded hydrogen atoms were placed using a riding model. Their positions were constrained relative to their parent atom using the appropriate HFIX command in SHELXL-2014.
Notes: Excellent data and refinement. There is a minor chemical disorder at the Br1 atom (93 % Br, 7 %H).

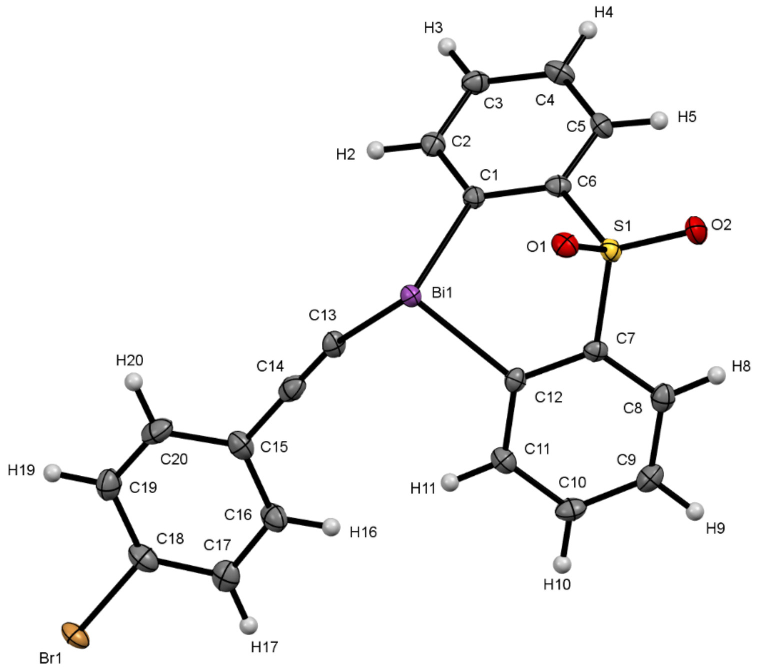

Figure S.4 Asymmetric unit in the crystal structure of compound A[5].

Table S.18 Crystal data and structure refinement for compound A[5].

Empirical formula	C20 H12.07 Bi Br0.92 O2 S
Formula weight	599.17
Temperature	100.0 K
Wavelength	0.71073 Å
Crystal system	Triclinic
Space group	P-1

Unit cell dimensions	a = 8.3788(8) Å	α= 69.917(3)°.
	b = 9.4899(9) Å	β= 87.930(3)°.
	c = 12.4028(11) Å	γ= 74.563(3)°.
Volume	891.15(15) Å3	
Z	2	
Density (calculated)	2.233 Mg/m3	
Absorption coefficient	12.093 mm-1	
F(000)	559	
Crystal size	0.15 x 0.05 x 0.05 mm3	
Theta range for data collection	1.751 to 25.393°.	
Index ranges	-10<=h<=10, -11<=k<=11, -14<=l<=14	
Reflections collected	23898	
Independent reflections	3274 [R(int) = 0.0319]	
Completeness to theta = 25.242°	100.0 %	
Absorption correction	Semi-empirical from equivalents	
Max. and min. transmission	0.4901 and 0.3629	
Refinement method	Full-matrix least-squares on F2	
Data / restraints / parameters	3274 / 0 / 228	
Goodness-of-fit on F2	1.074	
Final R indices [I>2sigma(I)]	R1 = 0.0168, wR2 = 0.0320	
R indices (all data)	R1 = 0.0205, wR2 = 0.0329	
Extinction coefficient	n/a	
Largest diff. peak and hole	0.733 and -0.509 e.Å-3	

Table S.19 Bond lengths [Å] and angles [°] for compound A[5].

Bi(1)-C(8)	2.231(3)
Bi(1)-C(9)	2.271(3)
Bi(1)-C(20)	2.281(3)
Br(1)-C(1)	1.900(3)
S(1)-O(1)	1.437(2)
S(1)-O(2)	1.456(2)
S(1)-C(14)	1.767(3)
S(1)-C(15)	1.763(3)
C(1)-C(2)	1.380(5)
C(1)-C(6)	1.380(5)
C(2)-C(3)	1.381(5)
C(3)-C(4)	1.402(5)
C(4)-C(5)	1.390(5)
C(4)-C(7)	1.458(5)
C(5)-C(6)	1.377(5)
C(7)-C(8)	1.175(5)
C(9)-C(10)	1.390(5)
C(9)-C(14)	1.388(4)
C(10)-C(11)	1.396(5)
C(11)-C(12)	1.389(5)
C(12)-C(13)	1.373(5)
C(13)-C(14)	1.394(5)

C(15)-C(16)	1.391(4)
C(15)-C(20)	1.393(4)
C(16)-C(17)	1.385(5)
C(17)-C(18)	1.384(5)
C(18)-C(19)	1.392(5)
C(19)-C(20)	1.387(5)
C(8)-Bi(1)-C(9)	90.76(12)
C(8)-Bi(1)-C(20)	95.13(12)
C(9)-Bi(1)-C(20)	87.10(11)
O(1)-S(1)-O(2)	119.00(14)
O(1)-S(1)-C(14)	110.21(15)
O(1)-S(1)-C(15)	109.69(15)
O(2)-S(1)-C(14)	105.80(14)
O(2)-S(1)-C(15)	105.77(14)
C(15)-S(1)-C(14)	105.48(15)
C(2)-C(1)-Br(1)	119.9(3)
C(2)-C(1)-C(6)	121.6(3)
C(6)-C(1)-Br(1)	118.5(3)
C(1)-C(2)-C(3)	119.1(3)
C(2)-C(3)-C(4)	120.5(3)
C(3)-C(4)-C(7)	119.9(3)
C(5)-C(4)-C(3)	118.8(3)
C(5)-C(4)-C(7)	121.3(3)
C(6)-C(5)-C(4)	121.0(3)
C(5)-C(6)-C(1)	119.0(3)
C(8)-C(7)-C(4)	178.2(4)
C(7)-C(8)-Bi(1)	159.8(3)
C(10)-C(9)-Bi(1)	123.1(2)
C(14)-C(9)-Bi(1)	119.3(2)
C(14)-C(9)-C(10)	117.7(3)
C(9)-C(10)-C(11)	120.3(3)
C(12)-C(11)-C(10)	120.3(3)
C(13)-C(12)-C(11)	120.4(3)
C(12)-C(13)-C(14)	118.4(3)
C(9)-C(14)-S(1)	117.8(2)
C(9)-C(14)-C(13)	122.8(3)
C(13)-C(14)-S(1)	119.3(2)
C(16)-C(15)-S(1)	118.8(2)
C(16)-C(15)-C(20)	122.6(3)
C(20)-C(15)-S(1)	118.6(2)
C(17)-C(16)-C(15)	118.6(3)
C(18)-C(17)-C(16)	119.8(3)
C(17)-C(18)-C(19)	120.9(3)
C(20)-C(19)-C(18)	120.3(3)
C(15)-C(20)-Bi(1)	118.3(2)
C(19)-C(20)-Bi(1)	123.9(2)
C(19)-C(20)-C(15)	117.8(3)

X-Ray Crystallographic Analysis of bismuth(III) acetylide A[6]

The single-crystal X-ray diffraction studies were carried out on a Bruker APEX II ULTRA CCD diffractometer equipped with Mo Kα radiation (λ = 0.71073 Å). Crystals of the subject compound were used as received. A 0.190 x 0.100 x 0.050 mm colorless prism crystal was mounted on a Cryoloop with Paratone N oil. Data were collected in a nitrogen gas stream at 100(2) K using ϕ and ϖ scans. Crystal-to-detector distance was 45 mm using an exposure time of 2 seconds with a scan width of 0.70°. Data collection was 100.0% complete to 25.242° in θ. A total of 18762 reflections were collected. 3830 reflections were found to be symmetry independent, with a Rint of 0.0335. Indexing and unit cell refinement indicated a Primitive Monoclinic lattice. The space group was found to be P21/c. The data were integrated using the Bruker SAINT Software program and scaled using the SADABS software program. Solution by direct methods (SHELXT) produced a complete phasing model consistent with the proposed structure. All non-hydrogen atoms were refined anisotropically by full-matrix least-squares (SHELXL-2014). All carbon bonded hydrogen atoms were placed using a riding model. Their positions were constrained relative to their parent atom using the appropriate HFIX command in SHELXL-2014.
Notes: Excellent data and refinement. There is a minor chemical disorder with approximately 3% of the iodo-Bi starting material present.

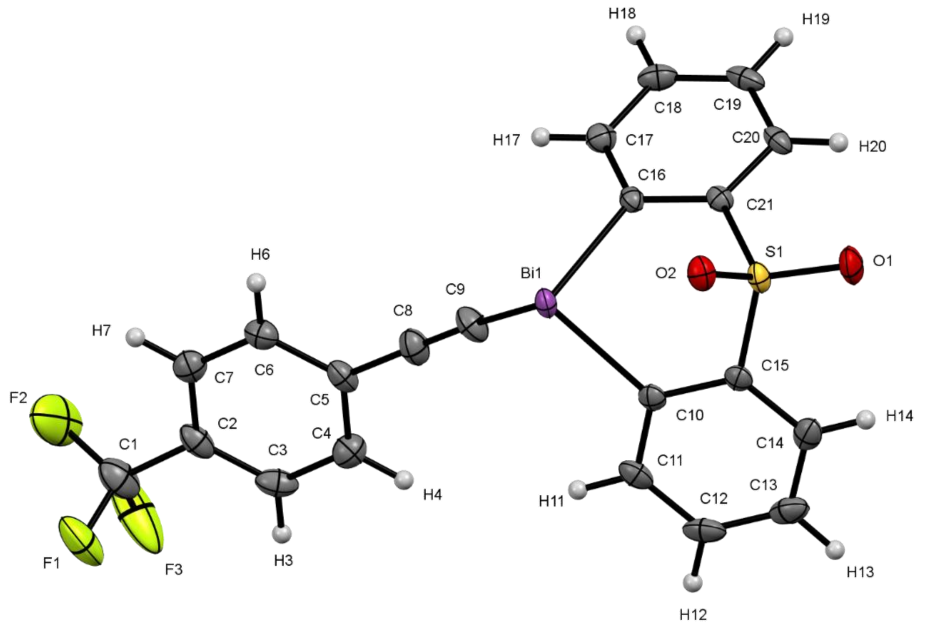

Figure S.55 Asymmetric unit in the crystal structure of compound A[6].

Table S.20 Crystal data and structure refinement for compound A[6].

Empirical formula	C21 H12 Bi F3 I0.03 O2 S
Formula weight	598.15
Temperature	100.0 K
Wavelength	0.71073 Å
Crystal system	Monoclinic

Space group	P 1 21/c 1	
Unit cell dimensions	a = 7.7441(5) Å	α= 90°.
	b = 9.9411(7) Å	β= 98.4280(10)°.
	c = 24.6103(16) Å	γ = 90°.
Volume	1874.2(2) Å3	
Z	4	
Density (calculated)	2.120 Mg/m3	
Absorption coefficient	9.611 mm-1	
F(000)	1126	
Crystal size	0.19 x 0.1 x 0.05 mm3	
Theta range for data collection	1.673 to 26.368°.	
Index ranges	-9<=h<=8, -12<=k<=12, -30<=l<=30	
Reflections collected	18762	
Independent reflections	3830 [R(int) = 0.0335]	
Completeness to theta = 25.242°	100.0 %	
Absorption correction	Semi-empirical from equivalents	
Max. and min. transmission	0.2607 and 0.1551	
Refinement method	Full-matrix least-squares on F2	
Data / restraints / parameters	3830 / 0 / 258	
Goodness-of-fit on F2	1.079	
Final R indices [I>2sigma(I)]	R1 = 0.0189, wR2 = 0.0412	
R indices (all data)	R1 = 0.0219, wR2 = 0.0420	
Extinction coefficient	n/a	
Largest diff. peak and hole	0.985 and -0.521 e.Å-3	

Table S.21 Bond lengths [Å] and angles [°] for compound A[6].

Bi(1)-C(10)	2.263(3)
Bi(1)-C(16)	2.267(3)
Bi(1)-C(9)	2.210(5)
Bi(1)-I(1)	2.931(17)
S(1)-O(2)	1.449(2)
S(1)-O(1)	1.439(2)
S(1)-C(15)	1.765(3)
S(1)-C(21)	1.778(3)
F(1)-C(1)	1.322(4)
C(15)-C(10)	1.393(4)
C(15)-C(14)	1.393(4)
F(3)-C(1)	1.325(5)
F(2)-C(1)	1.333(5)
C(5)-C(4)	1.395(5)
C(5)-C(6)	1.403(5)
C(5)-C(8)	1.434(5)
C(5)-I(1)	2.122(17)
C(10)-C(11)	1.384(4)
C(16)-C(21)	1.382(4)
C(16)-C(17)	1.390(4)
C(21)-C(20)	1.390(4)

C(20)-H(20)	0.9500
C(20)-C(19)	1.385(5)
C(14)-H(14)	0.9500
C(14)-C(13)	1.385(5)
C(12)-H(12)	0.9500
C(12)-C(13)	1.380(5)
C(12)-C(11)	1.394(5)
C(18)-H(18)	0.9500
C(18)-C(19)	1.382(5)
C(18)-C(17)	1.393(5)
C(4)-H(4)	0.9500
C(4)-C(3)	1.383(5)
C(13)-H(13)	0.9500
C(2)-C(7)	1.392(5)
C(2)-C(3)	1.383(5)
C(2)-C(1)	1.477(5)
C(19)-H(19)	0.9500
C(9)-C(8)	1.222(7)
C(7)-H(7)	0.9500
C(7)-C(6)	1.366(5)
C(17)-H(17)	0.9500
C(6)-H(6)	0.9500
C(3)-H(3)	0.9500
C(11)-H(11)	0.9500
C(10)-Bi(1)-C(16)	85.21(10)
C(10)-Bi(1)-I(1)	89.2(3)
C(16)-Bi(1)-I(1)	89.6(3)
C(9)-Bi(1)-C(10)	92.67(13)
C(9)-Bi(1)-C(16)	92.72(12)
O(2)-S(1)-C(15)	106.45(13)
O(2)-S(1)-C(21)	106.44(14)
O(1)-S(1)-O(2)	118.82(14)
O(1)-S(1)-C(15)	110.52(15)
O(1)-S(1)-C(21)	109.77(14)
C(15)-S(1)-C(21)	103.72(14)
C(10)-C(15)-S(1)	117.5(2)
C(14)-C(15)-S(1)	119.8(3)
C(14)-C(15)-C(10)	122.7(3)
C(4)-C(5)-C(6)	118.6(3)
C(4)-C(5)-C(8)	121.1(3)
C(4)-C(5)-I(1)	115.3(5)
C(6)-C(5)-C(8)	120.1(3)
C(6)-C(5)-I(1)	125.9(4)
C(15)-C(10)-Bi(1)	119.0(2)
C(11)-C(10)-Bi(1)	123.2(2)
C(11)-C(10)-C(15)	117.8(3)
C(21)-C(16)-Bi(1)	118.9(2)

C(21)-C(16)-C(17)	117.6(3)
C(17)-C(16)-Bi(1)	123.4(2)
C(16)-C(21)-S(1)	117.7(2)
C(16)-C(21)-C(20)	123.0(3)
C(20)-C(21)-S(1)	119.4(2)
C(21)-C(20)-H(20)	120.9
C(19)-C(20)-C(21)	118.2(3)
C(19)-C(20)-H(20)	120.9
C(15)-C(14)-H(14)	121.0
C(13)-C(14)-C(15)	118.0(3)
C(13)-C(14)-H(14)	121.0
C(13)-C(12)-H(12)	119.6
C(13)-C(12)-C(11)	120.8(3)
C(11)-C(12)-H(12)	119.6
C(19)-C(18)-H(18)	119.9
C(19)-C(18)-C(17)	120.3(3)
C(17)-C(18)-H(18)	119.9
C(5)-C(4)-H(4)	120.0
C(3)-C(4)-C(5)	120.0(3)
C(3)-C(4)-H(4)	120.0
C(14)-C(13)-H(13)	119.8
C(12)-C(13)-C(14)	120.4(3)
C(12)-C(13)-H(13)	119.8
C(7)-C(2)-C(1)	120.1(3)
C(3)-C(2)-C(7)	119.8(3)
C(3)-C(2)-C(1)	120.1(3)
C(20)-C(19)-H(19)	119.8
C(18)-C(19)-C(20)	120.4(3)
C(18)-C(19)-H(19)	119.8
C(8)-C(9)-Bi(1)	160.8(4)
C(2)-C(7)-H(7)	120.1
C(6)-C(7)-C(2)	119.8(3)
C(6)-C(7)-H(7)	120.1
C(16)-C(17)-C(18)	120.6(3)
C(16)-C(17)-H(17)	119.7
C(18)-C(17)-H(17)	119.7
C(5)-C(6)-H(6)	119.4
C(7)-C(6)-C(5)	121.2(3)
C(7)-C(6)-H(6)	119.4
C(4)-C(3)-H(3)	119.7
C(2)-C(3)-C(4)	120.6(3)
C(2)-C(3)-H(3)	119.7
C(9)-C(8)-C(5)	175.0(4)
C(10)-C(11)-C(12)	120.3(3)
C(10)-C(11)-H(11)	119.9
C(12)-C(11)-H(11)	119.9
F(1)-C(1)-F(3)	106.2(3)
F(1)-C(1)-F(2)	104.3(4)

F(1)-C(1)-C(2)	113.7(3)
F(3)-C(1)-F(2)	105.8(4)
F(3)-C(1)-C(2)	112.7(3)
F(2)-C(1)-C(2)	113.3(3)
C(5)-I(1)-Bi(1)	141.4(7)

X-Ray Crystallographic Analysis of bismuth(III) acetylide A[7]

The single-crystal X-ray diffraction studies were carried out on a Bruker Apex II Ultra2 CCD diffractometer equipped with Mo Kα radiation (λ = 0.71073). A 0.090 x 0.070 x 0.040 mm colorless crystal was mounted on a Cryoloop with Paratone oil. Data were collected in a nitrogen gas stream at 100(2) K using ϕ and ϖ scans. Crystal-to-detector distance was 40 mm using exposure time 1.0s with a scan width of 0.80°. Data collection was 100.0% complete to 25.242° in θ. A total of 32975 reflections were collected covering the indices, -16<=h<=16, -9<=k<=9, -25<=l<=20. 4097 reflections were found to be symmetry independent, with a Rint of 0.0705. Indexing and unit cell refinement indicated a Primitive, Monoclinic lattice. The space group was found to be P21/c. The data were integrated using the Bruker SAINT Software program and scaled using the SADABS software program. Solution by direct methods (SHELXT) produced a complete phasing model consistent with the proposed structure. All nonhydrogen atoms were refined anisotropically by full-matrix least-squares (SHELXL-2014). All carbon bonded hydrogen atoms were placed using a riding model. Their positions were constrained relative to their parent atom using the appropriate HFIX command in SHELXL-2014.

Notes: Proposed structure in agreement with model derived from diffraction data. Excellent data and refinement

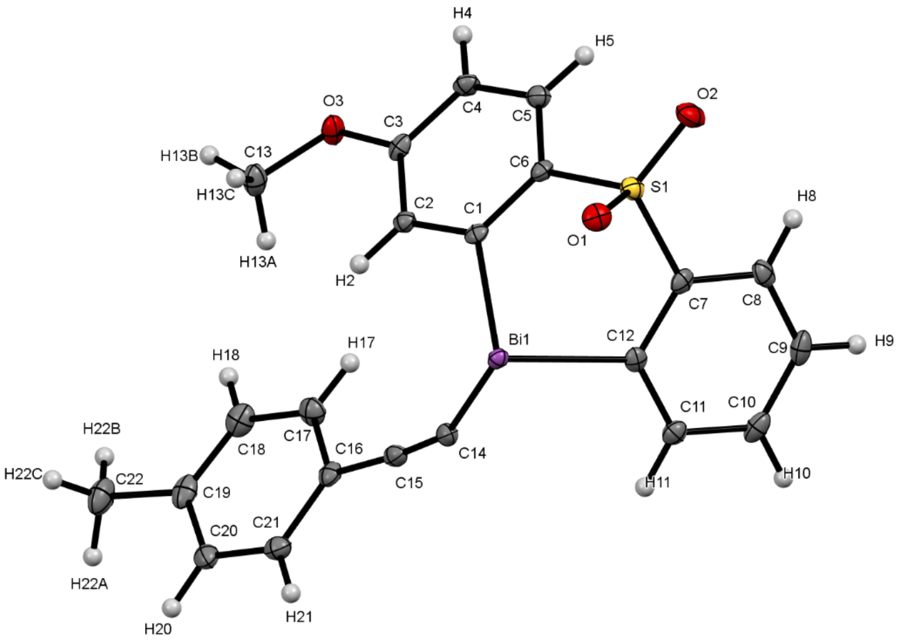

Figure S.16 Asymmetric unit in the crystal structure of compound A[7].

Table S.22 Crystal data and structure refinement for compound A[7].

Empirical formula	C22 H17 Bi O3 S
Molecular formula	C22 H17 Bi O3 S
Formula weight	570.39
Temperature	100.0 K
Wavelength	0.71073 Å
Crystal system	Monoclinic
Space group	P 1 21/c 1
Unit cell dimensions	a = 12.7269(4) Å α = 90°.
	b = 7.4850(2) Å β = 104.6713(8)°.

	c = 20.1551(6) Å γ = 90°.
Volume	1857.39(9) Å3
Z	4
Density (calculated)	2.040 Mg/m3
Absorption coefficient	9.625 mm-1
F(000)	1088
Crystal size	0.09 x 0.07 x 0.04 mm3
Crystal color, habit	colorless block
Theta range for data collection	1.654 to 27.101°.
Index ranges	-16<=h<=16, -9<=k<=9, -25<=l<=20
Reflections collected	32975
Independent reflections	4097 [R(int) = 0.0705]
Completeness to theta = 25.242°	100.0 %
Absorption correction	Semi-empirical from equivalents
Max. and min. transmission	0.2622 and 0.1787
Refinement method	Full-matrix least-squares on F2
Data / restraints / parameters	4097 / 0 / 246
Goodness-of-fit on F2	1.047
Final R indices [I>2sigma(I)]	R1 = 0.0208, wR2 = 0.0521
R indices (all data)	R1 = 0.0220, wR2 = 0.0531
Largest diff. peak and hole	0.591 and -0.923 e.Å-3

Table S.23 Bond lengths [Å] and angles [°] for compound A[7].

Bi(1)-C(1)	2.294(3)
Bi(1)-C(12)	2.270(3)
Bi(1)-C(14)	2.230(3)
S(1)-O(1)	1.453(2)
S(1)-O(2)	1.437(2)
S(1)-C(6)	1.764(3)
S(1)-C(7)	1.768(3)
O(3)-C(3)	1.365(3)
O(3)-C(13)	1.430(4)
C(9)-H(9)	0.9500
C(9)-C(8)	1.390(5)
C(9)-C(10)	1.390(5)
C(2)-H(2)	0.9500
C(2)-C(1)	1.390(4)
C(2)-C(3)	1.401(4)
C(21)-H(21)	0.9500
C(21)-C(20)	1.393(5)
C(21)-C(16)	1.391(4)
C(1)-C(6)	1.393(4)
C(3)-C(4)	1.395(4)
C(13)-H(13A)	0.9800
C(13)-H(13B)	0.9800
C(13)-H(13C)	0.9800
C(8)-H(8)	0.9500

C(8)-C(7)	1.399(4)
C(15)-C(16)	1.440(4)
C(15)-C(14)	1.210(4)
C(20)-H(20)	0.9500
C(20)-C(19)	1.384(5)
C(12)-C(11)	1.386(4)
C(12)-C(7)	1.395(4)
C(6)-C(5)	1.393(4)
C(19)-C(22)	1.503(4)
C(19)-C(18)	1.392(5)
C(16)-C(17)	1.404(4)
C(10)-H(10)	0.9500
C(10)-C(11)	1.400(4)
C(4)-H(4)	0.9500
C(4)-C(5)	1.385(4)
C(11)-H(11)	0.9500
C(5)-H(5)	0.9500
C(17)-H(17)	0.9500
C(17)-C(18)	1.382(4)
C(22)-H(22A)	0.9800
C(22)-H(22B)	0.9800
C(22)-H(22C)	0.9800
C(18)-H(18)	0.9500

C(12)-Bi(1)-C(1)	86.77(9)
C(14)-Bi(1)-C(1)	88.38(11)
C(14)-Bi(1)-C(12)	92.22(10)
O(1)-S(1)-C(6)	106.17(14)
O(1)-S(1)-C(7)	106.10(14)
O(2)-S(1)-O(1)	120.01(14)
O(2)-S(1)-C(6)	110.18(13)
O(2)-S(1)-C(7)	110.24(14)
C(6)-S(1)-C(7)	102.69(13)
C(3)-O(3)-C(13)	117.5(2)
C(8)-C(9)-H(9)	120.0
C(8)-C(9)-C(10)	120.0(3)
C(10)-C(9)-H(9)	120.0
C(1)-C(2)-H(2)	120.4
C(1)-C(2)-C(3)	119.3(3)
C(3)-C(2)-H(2)	120.4
C(20)-C(21)-H(21)	120.0
C(16)-C(21)-H(21)	120.0
C(16)-C(21)-C(20)	120.0(3)
C(2)-C(1)-Bi(1)	122.6(2)
C(2)-C(1)-C(6)	119.0(3)
C(6)-C(1)-Bi(1)	118.1(2)
O(3)-C(3)-C(2)	124.1(3)
O(3)-C(3)-C(4)	115.0(2)

C(4)-C(3)-C(2)	120.9(3)
O(3)-C(13)-H(13A)	109.5
O(3)-C(13)-H(13B)	109.5
O(3)-C(13)-H(13C)	109.5
H(13A)-C(13)-H(13B)	109.5
H(13A)-C(13)-H(13C)	109.5
H(13B)-C(13)-H(13C)	109.5
C(9)-C(8)-H(8)	120.9
C(9)-C(8)-C(7)	118.3(3)
C(7)-C(8)-H(8)	120.9
C(14)-C(15)-C(16)	174.9(3)
C(21)-C(20)-H(20)	119.4
C(19)-C(20)-C(21)	121.2(3)
C(19)-C(20)-H(20)	119.4
C(11)-C(12)-Bi(1)	123.5(2)
C(11)-C(12)-C(7)	117.6(3)
C(7)-C(12)-Bi(1)	118.7(2)
C(1)-C(6)-S(1)	117.6(2)
C(1)-C(6)-C(5)	122.0(3)
C(5)-C(6)-S(1)	120.4(2)
C(20)-C(19)-C(22)	120.4(3)
C(20)-C(19)-C(18)	118.5(3)
C(18)-C(19)-C(22)	121.0(3)
C(21)-C(16)-C(15)	122.0(3)
C(21)-C(16)-C(17)	118.5(3)
C(17)-C(16)-C(15)	119.2(3)
C(9)-C(10)-H(10)	119.7
C(9)-C(10)-C(11)	120.6(3)
C(11)-C(10)-H(10)	119.7
C(3)-C(4)-H(4)	120.0
C(5)-C(4)-C(3)	119.9(3)
C(5)-C(4)-H(4)	120.0
C(12)-C(11)-C(10)	120.7(3)
C(12)-C(11)-H(11)	119.7
C(10)-C(11)-H(11)	119.7
C(6)-C(5)-H(5)	120.6
C(4)-C(5)-C(6)	118.8(3)
C(4)-C(5)-H(5)	120.6
C(8)-C(7)-S(1)	119.9(2)
C(12)-C(7)-S(1)	117.3(2)
C(12)-C(7)-C(8)	122.8(3)
C(16)-C(17)-H(17)	119.7
C(18)-C(17)-C(16)	120.6(3)
C(18)-C(17)-H(17)	119.7
C(19)-C(22)-H(22A)	109.5
C(19)-C(22)-H(22B)	109.5
C(19)-C(22)-H(22C)	109.5
H(22A)-C(22)-H(22B)	109.5

H(22A)-C(22)-H(22C)	109.5
H(22B)-C(22)-H(22C)	109.5
C(15)-C(14)-Bi(1)	156.9(2)
C(19)-C(18)-H(18)	119.6
C(17)-C(18)-C(19)	120.7(3)
C(17)-C(18)-H(18)	119.6

X-Ray Crystallographic Analysis of bismuth(III) acetylide A[8]

The single-crystal X-ray diffraction studies were carried out on a Bruker Apex II Ultra2 CCD diffractometer equipped with Mo Kα radiation (λ = 0.71073). A 0.160 x 0.140 x 0.040 mm colorless crystal was mounted on a Cryoloop with Paratone oil. Data were collected in a nitrogen gas stream at 100(2) K using ϕ and ϖ scans. Crystal-to-detector distance was 40 mm using exposure time 1.0s with a scan width of 0.80°. Data collection was 100.0% complete to 25.242° in θ. A total of 18974 reflections were collected covering the indices, -16<=h<=16, -9<=k<=9, -20<=l<=25. 3886 reflections were found to be symmetry independent, with a R_{int} of 0.0508. Indexing and unit cell refinement indicated a Primitive, Monoclinic lattice. The space group was found to be P21/c. The data were integrated using the Bruker SAINT Software program and scaled using the SADABS software program. Solution by direct methods (SHELXT) produced a complete phasing model consistent with the proposed structure. All nonhydrogen atoms were refined anisotropically by full-matrix least-squares (SHELXL-2014). All carbon bonded hydrogen atoms were placed using a riding model. Their positions were constrained relative to their parent atom using the appropriate HFIX command in SHELXL-2014.

Notes: Proposed structure in agreement with model derived from diffraction data. Excellent data and refinement

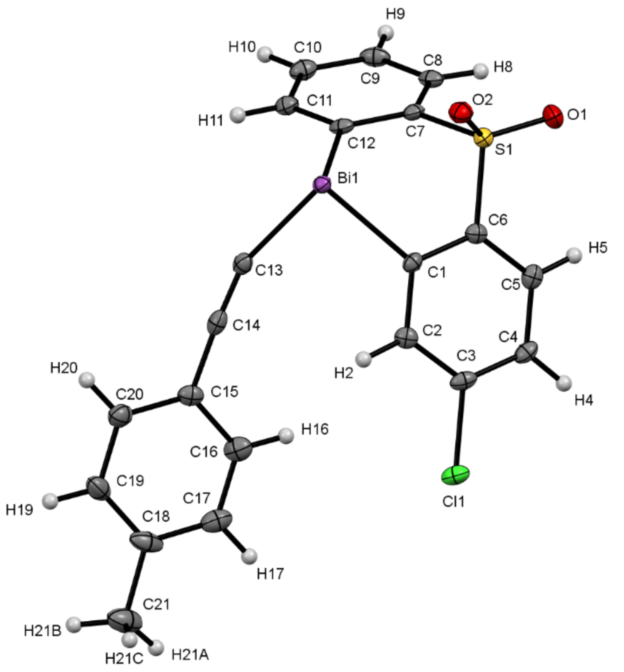

Figure S.67 Asymmetric unit in the crystal structure of compound A[8].

Table S.24 Crystal data and structure refinement for compound A[8].

Empirical formula	C21 H14 Bi Cl O2 S
Molecular formula	C21 H14 Bi Cl O2 S
Formula weight	574.81
Temperature	100.0 K
Wavelength	0.71073 Å
Crystal system	Monoclinic

Space group	P 1 21/c 1
Unit cell dimensions	a = 12.7458(6) Å α = 90°.
	b = 7.3360(3) Å β = 105.2780(10)°.
	c = 20.2483(8) Å γ = 90°.
Volume	1826.37(14) Å3
Z	4
Density (calculated)	2.090 Mg/m3
Absorption coefficient	9.927 mm-1
F(000)	1088
Crystal size	0.16 x 0.14 x 0.04 mm3
Crystal color, habit	colorless irregular
Theta range for data collection	1.656 to 26.730°.
Index ranges	-16<=h<=16, -9<=k<=9, -20<=l<=25
Reflections collected	18974
Independent reflections	3886 [R(int) = 0.0508]
Completeness to theta = 25.242°	100.0 %
Absorption correction	Semi-empirical from equivalents
Max. and min. transmission	0.2612 and 0.1459
Refinement method	Full-matrix least-squares on F2
Data / restraints / parameters	3886 / 0 / 236
Goodness-of-fit on F2	1.050
Final R indices [I>2sigma(I)]	R1 = 0.0194, wR2 = 0.0487
R indices (all data)	R1 = 0.0214, wR2 = 0.0499
Largest diff. peak and hole	1.068 and -0.449 e.Å-3

Table S.25 Bond lengths [Å] and angles [°] for compound A[8].

Bi(1)-C(1)	2.284(3)
Bi(1)-C(12)	2.270(3)
Bi(1)-C(13)	2.230(3)
Cl(1)-C(3)	1.749(3)
S(1)-O(2)	1.452(2)
S(1)-O(1)	1.436(2)
S(1)-C(6)	1.770(3)
S(1)-C(7)	1.767(3)
C(20)-H(20)	0.9500
C(20)-C(19)	1.395(5)
C(20)-C(15)	1.391(5)
C(6)-C(1)	1.394(4)
C(6)-C(5)	1.389(4)
C(17)-H(17)	0.9500
C(17)-C(18)	1.395(5)
C(17)-C(16)	1.382(4)
C(21)-H(21A)	0.9800
C(21)-H(21B)	0.9800
C(21)-H(21C)	0.9800
C(21)-C(18)	1.500(4)
C(1)-C(2)	1.393(4)

C(11)-H(11)	0.9500
C(11)-C(12)	1.384(4)
C(11)-C(10)	1.396(4)
C(12)-C(7)	1.397(4)
C(5)-H(5)	0.9500
C(5)-C(4)	1.395(4)
C(8)-H(8)	0.9500
C(8)-C(9)	1.386(4)
C(8)-C(7)	1.394(4)
C(9)-H(9)	0.9500
C(9)-C(10)	1.392(5)
C(3)-C(2)	1.386(4)
C(3)-C(4)	1.383(4)
C(14)-C(13)	1.196(4)
C(14)-C(15)	1.444(4)
C(19)-H(19)	0.9500
C(19)-C(18)	1.391(5)
C(2)-H(2)	0.9500
C(10)-H(10)	0.9500
C(4)-H(4)	0.9500
C(15)-C(16)	1.401(4)
C(16)-H(16)	0.9500
C(12)-Bi(1)-C(1)	86.63(9)
C(13)-Bi(1)-C(1)	87.84(11)
C(13)-Bi(1)-C(12)	94.46(10)
O(2)-S(1)-C(6)	105.40(13)
O(2)-S(1)-C(7)	106.16(14)
O(1)-S(1)-O(2)	119.79(14)
O(1)-S(1)-C(6)	110.36(13)
O(1)-S(1)-C(7)	110.34(14)
C(7)-S(1)-C(6)	103.46(13)
C(19)-C(20)-H(20)	119.8
C(15)-C(20)-H(20)	119.8
C(15)-C(20)-C(19)	120.4(3)
C(1)-C(6)-S(1)	117.0(2)
C(5)-C(6)-S(1)	120.2(2)
C(5)-C(6)-C(1)	122.8(3)
C(18)-C(17)-H(17)	119.6
C(16)-C(17)-H(17)	119.6
C(16)-C(17)-C(18)	120.8(3)
H(21A)-C(21)-H(21B)	109.5
H(21A)-C(21)-H(21C)	109.5
H(21B)-C(21)-H(21C)	109.5
C(18)-C(21)-H(21A)	109.5
C(18)-C(21)-H(21B)	109.5
C(18)-C(21)-H(21C)	109.5
C(6)-C(1)-Bi(1)	118.7(2)
C(2)-C(1)-Bi(1)	123.0(2)

C(2)-C(1)-C(6)	118.2(3)
C(12)-C(11)-H(11)	119.8
C(12)-C(11)-C(10)	120.5(3)
C(10)-C(11)-H(11)	119.8
C(11)-C(12)-Bi(1)	123.6(2)
C(11)-C(12)-C(7)	117.9(3)
C(7)-C(12)-Bi(1)	118.4(2)
C(6)-C(5)-H(5)	120.7
C(6)-C(5)-C(4)	118.6(3)
C(4)-C(5)-H(5)	120.7
C(9)-C(8)-H(8)	120.8
C(9)-C(8)-C(7)	118.3(3)
C(7)-C(8)-H(8)	120.8
C(8)-C(9)-H(9)	119.9
C(8)-C(9)-C(10)	120.1(3)
C(10)-C(9)-H(9)	119.9
C(2)-C(3)-Cl(1)	118.1(2)
C(4)-C(3)-Cl(1)	118.9(2)
C(4)-C(3)-C(2)	123.0(3)
C(13)-C(14)-C(15)	176.4(3)
C(14)-C(13)-Bi(1)	155.6(3)
C(20)-C(19)-H(19)	119.5
C(18)-C(19)-C(20)	121.0(3)
C(18)-C(19)-H(19)	119.5
C(1)-C(2)-H(2)	120.6
C(3)-C(2)-C(1)	118.8(3)
C(3)-C(2)-H(2)	120.6
C(17)-C(18)-C(21)	120.9(3)
C(19)-C(18)-C(17)	118.3(3)
C(19)-C(18)-C(21)	120.7(3)
C(11)-C(10)-H(10)	119.8
C(9)-C(10)-C(11)	120.5(3)
C(9)-C(10)-H(10)	119.8
C(12)-C(7)-S(1)	117.7(2)
C(8)-C(7)-S(1)	119.7(2)
C(8)-C(7)-C(12)	122.6(3)
C(5)-C(4)-H(4)	120.7
C(3)-C(4)-C(5)	118.6(3)
C(3)-C(4)-H(4)	120.7
C(20)-C(15)-C(14)	121.9(3)
C(20)-C(15)-C(16)	118.4(3)
C(16)-C(15)-C(14)	119.5(3)
C(17)-C(16)-C(15)	120.9(3)
C(17)-C(16)-H(16)	119.6
C(15)-C(16)-H(16)	119.6

X-Ray Crystallographic Analysis of 2,8-dimethoxy-10-(p-tolylethynyl)-10H-dibenzo[b,e][1,4]thiabismine 5,5-dioxide A[9]

The single-crystal X-ray diffraction studies were carried out on a Bruker Apex II Ultra CCD diffractometer equipped with Mo Kα radiation (λ= 0.71073). Crystals of the subject compound were used as received. A 0.140 x 0.140 x 0.050 mm colorless crystal was mounted on a Cryoloop with Paratone oil. Data were collected in a nitrogen gas stream at 100(2) K using ϕ and ϖ scans. Crystal-to-detector distance was 40 mm using exposure time 5s with a scan width of 0.70°. Data collection was 100.0% complete to 25.242° in θ. A total of 25165 reflections were collected covering the indices, -9<=h<=9, -23<=k<=23, -16<=l<=16. 3952 reflections were found to be symmetry independent, with a Rint of 0.0281. Indexing and unit cell refinement indicated a Primitive, Monoclinic lattice. The space group was found to be P21/c. The data were integrated using the Bruker SAINT Software program and scaled using the SADABS software program. Solution by direct methods (SHELXT) produced a complete phasing model consistent with the proposed structure. All nonhydrogen atoms were refined anisotropically by full-matrix least-squares (SHELXL-2014). All carbon bonded hydrogen atoms were placed using a riding model. Their positions were constrained relative to their parent atom using the appropriate HFIX command in SHELXL-2014.
Notes: Proposed structure in agreement with model derived from diffraction data. Minor disorder or chemical impurity/co-crystalized has not been modeled

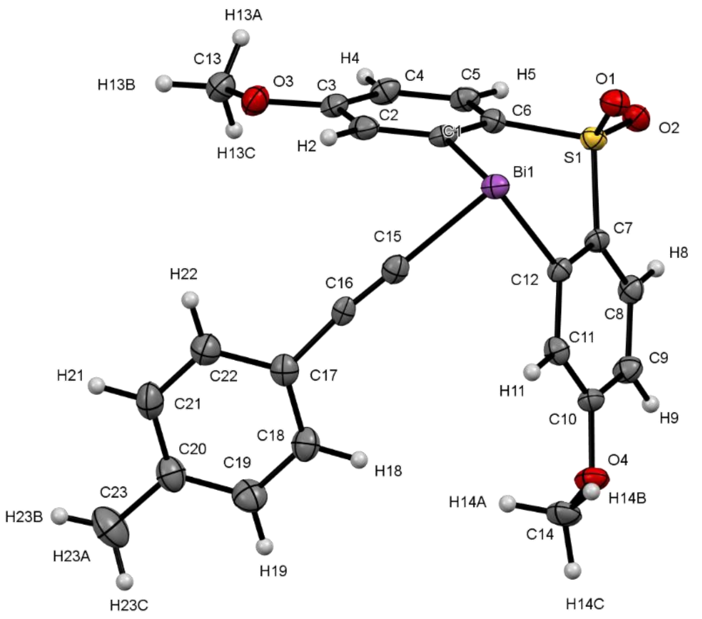

Figure S.18 Crystal structure of compound A[9].

Table S.26 Crystal data and structure refinement for compound A[9].

Empirical formula	C23 H19 Bi O4 S
Molecular formula	C23 H19 Bi O4 S
Formula weight	600.42
Temperature	100.0 K
Wavelength	0.71073 Å

Crystal system	Monoclinic
Space group	P 1 21/c 1
Unit cell dimensions	a = 7.6537(3) Å □= 90°.
	b = 19.4904(8) Å □= 90.9170(10)°.
	c = 13.9017(6) Å □□= 90°.
Volume	2073.50(15) Å3
Z	4
Density (calculated)	1.923 Mg/m3
Absorption coefficient	8.630 mm-1
F(000)	1152
Crystal size	0.14 x 0.14 x 0.05 mm3
Crystal color, habit	colorless block
Theta range for data collection	1.799 to 25.680°.
Index ranges	-9<=h<=9, -23<=k<=23, -16<=l<=16
Reflections collected	25165
Independent reflections	3952 [R(int) = 0.0281]
Completeness to theta = 25.242°	100.0 %
Absorption correction	Semi-empirical from equivalents
Max. and min. transmission	0.0926 and 0.0606
Refinement method	Full-matrix least-squares on F2
Data / restraints / parameters	3952 / 15 / 265
Goodness-of-fit on F2	1.088
Final R indices [I>2sigma(I)]	R1 = 0.0261, wR2 = 0.0606
R indices (all data)	R1 = 0.0305, wR2 = 0.0624
Largest diff. peak and hole	3.365 and -1.161 e.Å-3

Table S.27 Bond lengths [Å] and angles [°] for compound A[9].

Bi(1)-C(1)	2.287(4)
Bi(1)-C(12)	2.266(5)
Bi(1)-C(15)	2.221(5)
S(1)-O(1)	1.450(3)
S(1)-O(2)	1.443(3)
S(1)-C(6)	1.761(5)
S(1)-C(7)	1.764(5)
O(3)-C(3)	1.360(6)
O(3)-C(13)	1.430(6)
O(4)-C(10)	1.362(5)
O(4)-C(14)	1.431(6)
C(1)-C(2)	1.382(7)
C(1)-C(6)	1.399(6)
C(2)-H(2)	0.9500
C(2)-C(3)	1.401(7)
C(3)-C(4)	1.395(7)
C(4)-H(4)	0.9500
C(4)-C(5)	1.386(7)
C(5)-H(5)	0.9500
C(5)-C(6)	1.389(7)

C(7)-C(8)	1.385(6)
C(7)-C(12)	1.397(6)
C(8)-H(8)	0.9500
C(8)-C(9)	1.384(7)
C(9)-H(9)	0.9500
C(9)-C(10)	1.390(7)
C(10)-C(11)	1.398(6)
C(11)-H(11)	0.9500
C(11)-C(12)	1.400(6)
C(13)-H(13A)	0.9800
C(13)-H(13B)	0.9800
C(13)-H(13C)	0.9800
C(14)-H(14A)	0.9800
C(14)-H(14B)	0.9800
C(14)-H(14C)	0.9800
C(15)-C(16)	1.153(6)
C(16)-C(17)	1.478(7)
C(17)-C(18)	1.398(7)
C(17)-C(22)	1.393(7)
C(18)-H(18)	0.9500
C(18)-C(19)	1.385(7)
C(19)-H(19)	0.9500
C(19)-C(20)	1.390(8)
C(20)-C(21)	1.399(8)
C(20)-C(23)	1.507(7)
C(21)-H(21)	0.9500
C(21)-C(22)	1.385(7)
C(22)-H(22)	0.9500
C(23)-H(23A)	0.9800
C(23)-H(23B)	0.9800
C(23)-H(23C)	0.9800
C(12)-Bi(1)-C(1)	85.38(16)
C(15)-Bi(1)-C(1)	87.64(16)
C(15)-Bi(1)-C(12)	89.16(17)
O(1)-S(1)-C(6)	107.4(2)
O(1)-S(1)-C(7)	106.9(2)
O(2)-S(1)-O(1)	119.0(2)
O(2)-S(1)-C(6)	109.6(2)
O(2)-S(1)-C(7)	109.7(2)
C(6)-S(1)-C(7)	103.0(2)
C(3)-O(3)-C(13)	118.3(4)
C(10)-O(4)-C(14)	118.4(4)
C(2)-C(1)-Bi(1)	122.9(3)
C(2)-C(1)-C(6)	117.9(4)
C(6)-C(1)-Bi(1)	118.7(3)
C(1)-C(2)-H(2)	119.7
C(1)-C(2)-C(3)	120.7(4)

C(3)-C(2)-H(2)	119.7
O(3)-C(3)-C(2)	115.2(4)
O(3)-C(3)-C(4)	124.2(4)
C(4)-C(3)-C(2)	120.5(5)
C(3)-C(4)-H(4)	120.4
C(5)-C(4)-C(3)	119.3(4)
C(5)-C(4)-H(4)	120.4
C(4)-C(5)-H(5)	120.3
C(4)-C(5)-C(6)	119.5(4)
C(6)-C(5)-H(5)	120.3
C(1)-C(6)-S(1)	116.9(4)
C(5)-C(6)-S(1)	120.8(4)
C(5)-C(6)-C(1)	122.1(4)
C(8)-C(7)-S(1)	121.3(3)
C(8)-C(7)-C(12)	121.9(4)
C(12)-C(7)-S(1)	116.6(3)
C(7)-C(8)-H(8)	120.5
C(9)-C(8)-C(7)	119.0(4)
C(9)-C(8)-H(8)	120.5
C(8)-C(9)-H(9)	120.0
C(8)-C(9)-C(10)	120.1(4)
C(10)-C(9)-H(9)	120.0
O(4)-C(10)-C(9)	115.6(4)
O(4)-C(10)-C(11)	123.4(4)
C(9)-C(10)-C(11)	121.0(4)
C(10)-C(11)-H(11)	120.4
C(10)-C(11)-C(12)	119.2(4)
C(12)-C(11)-H(11)	120.4
C(7)-C(12)-Bi(1)	119.4(3)
C(7)-C(12)-C(11)	118.8(4)
C(11)-C(12)-Bi(1)	121.5(3)
O(3)-C(13)-H(13A)	109.5
O(3)-C(13)-H(13B)	109.5
O(3)-C(13)-H(13C)	109.5
H(13A)-C(13)-H(13B)	109.5
H(13A)-C(13)-H(13C)	109.5
H(13B)-C(13)-H(13C)	109.5
O(4)-C(14)-H(14A)	109.5
O(4)-C(14)-H(14B)	109.5
O(4)-C(14)-H(14C)	109.5
H(14A)-C(14)-H(14B)	109.5
H(14A)-C(14)-H(14C)	109.5
H(14B)-C(14)-H(14C)	109.5
C(16)-C(15)-Bi(1)	168.9(4)
C(15)-C(16)-C(17)	172.4(5)
C(18)-C(17)-C(16)	121.7(4)
C(22)-C(17)-C(16)	119.2(4)
C(22)-C(17)-C(18)	119.0(5)

C(17)-C(18)-H(18)	119.8
C(19)-C(18)-C(17)	120.4(5)
C(19)-C(18)-H(18)	119.8
C(18)-C(19)-H(19)	119.5
C(18)-C(19)-C(20)	120.9(5)
C(20)-C(19)-H(19)	119.5
C(19)-C(20)-C(21)	118.3(5)
C(19)-C(20)-C(23)	121.3(5)
C(21)-C(20)-C(23)	120.4(5)
C(20)-C(21)-H(21)	119.4
C(22)-C(21)-C(20)	121.2(5)
C(22)-C(21)-H(21)	119.4
C(17)-C(22)-H(22)	119.9
C(21)-C(22)-C(17)	120.1(5)
C(21)-C(22)-H(22)	119.9
C(20)-C(23)-H(23A)	109.5
C(20)-C(23)-H(23B)	109.5
C(20)-C(23)-H(23C)	109.5
H(23A)-C(23)-H(23B)	109.5
H(23A)-C(23)-H(23C)	109.5
H(23B)-C(23)-H(23C)	109.5

X-Ray Crystallographic Analysis of bismuth(III) acetylide A[10]

The single-crystal X-ray diffraction studies were carried out on a Bruker Apex II Ultra CCD diffractometer equipped with Mo Kα radiation (λ = 0.71073). A 0.220 x 0.15 x 0.125 mm colorless crystal was mounted on a Cryoloop with Paratone oil. Data were collected in a nitrogen gas stream at 100(2) K using ϕ and ϖ scans. Crystal-to-detector distance was 40 mm using exposure time 1s with a scan width of 0.80°. Data collection was 100.0% complete to 25.242° in θ. A total of 31005 reflections were collected covering the indices, -17<=h<=17, -12<=k<=12, -18<=l<=18. 3995 reflections were found to be symmetry independent, with a Rint of 0.0705. Indexing and unit cell refinement indicated a Primitive, Monoclinic lattice. The space group was found to be $P2_1/c$. The data were integrated using the Bruker SAINT Software program and scaled using the SADABS software program. Solution by direct methods (SHELXT) produced a complete phasing model consistent with the proposed structure. All nonhydrogen atoms were refined anisotropically by full-matrix least-squares (SHELXL-2014). All carbon bonded hydrogen atoms were placed using a riding model. Their positions were constrained relative to their parent atom using the appropriate HFIX command in SHELXL-2014.

Notes: Proposed structure in agreement with model derived from diffraction data. Slightly higher residual density 3.38 e.Å-3 possibly due to minor disorder

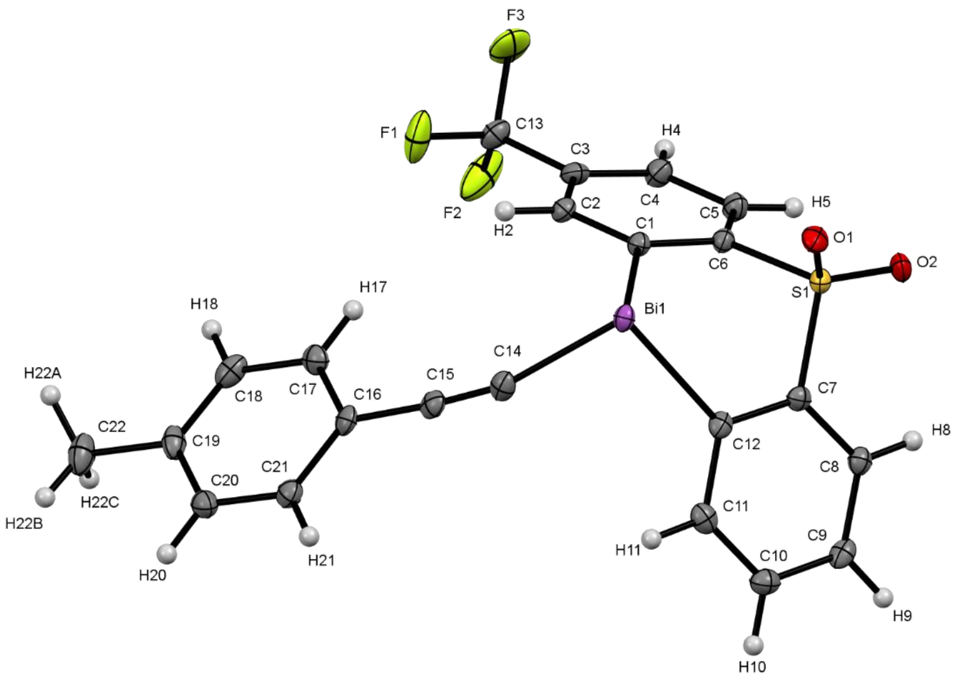

Figure S.197 Asymmetric unit in the crystal structure of compound A[10].

Table S.28 Crystal data and structure refinement for compound A[10].

Empirical formula	C22 H14 Bi F3 O2 S
Molecular formula	C22 H14 Bi F3 O2 S
Formula weight	608.37
Temperature	100.0 K
Wavelength	0.71073 Å

Crystal system	Monoclinic
Space group	P 1 21/c 1
Unit cell dimensions	a = 14.1582(7) Å α = 90°.
	b = 9.6556(5) Å β = 100.4560(10)°.
	c = 14.5266(7) Å γ = 90°.
Volume	1952.90(17) Å3
Z	4
Density (calculated)	2.069 Mg/m3
Absorption coefficient	9.179 mm-1
F(000)	1152
Crystal size	0.22 x 0.15 x 0.125 mm3
Crystal color, habit	colorless irregular
Theta range for data collection	1.463 to 26.370°.
Index ranges	-17<=h<=17, -12<=k<=12, -18<=l<=17
Reflections collected	31005
Independent reflections	3995 [R(int) = 0.0705]
Completeness to theta = 25.242°	100.0 %
Absorption correction	Semi-empirical from equivalents
Max. and min. transmission	0.2607 and 0.1761
Refinement method	Full-matrix least-squares on F2
Data / restraints / parameters	3995 / 0 / 263
Goodness-of-fit on F2	1.031
Final R indices [I>2sigma(I)]	R1 = 0.0242, wR2 = 0.0628
R indices (all data)	R1 = 0.0264, wR2 = 0.0639
Largest diff. peak and hole	3.380 and -0.951 e.Å-3

Table S.29 Bond lengths [Å] and angles [°] for compound A[10].

Bi(1)-C(1)	2.277(4)
Bi(1)-C(12)	2.271(4)
Bi(1)-C(14)	2.209(4)
S(1)-O(1)	1.446(3)
S(1)-O(2)	1.439(3)
S(1)-C(6)	1.768(4)
S(1)-C(7)	1.763(4)
F(1)-C(13)	1.322(5)
F(2)-C(13)	1.331(5)
F(3)-C(13)	1.349(5)
C(1)-C(2)	1.394(5)
C(1)-C(6)	1.398(5)
C(2)-C(3)	1.390(5)
C(3)-C(4)	1.384(6)
C(3)-C(13)	1.497(6)
C(4)-C(5)	1.397(6)
C(5)-C(6)	1.388(5)
C(7)-C(8)	1.390(5)
C(7)-C(12)	1.398(5)
C(8)-C(9)	1.388(6)

C(9)-C(10)	1.388(6)
C(10)-C(11)	1.397(6)
C(11)-C(12)	1.396(5)
C(14)-C(15)	1.198(6)
C(15)-C(16)	1.447(6)
C(16)-C(17)	1.404(6)
C(16)-C(21)	1.401(6)
C(17)-C(18)	1.380(6)
C(18)-C(19)	1.384(6)
C(19)-C(20)	1.396(6)
C(19)-C(22)	1.509(6)
C(20)-C(21)	1.387(5)
C(12)-Bi(1)-C(1)	86.56(13)
C(14)-Bi(1)-C(1)	86.97(14)
C(14)-Bi(1)-C(12)	92.50(14)
O(1)-S(1)-C(6)	105.38(17)
O(1)-S(1)-C(7)	107.05(18)
O(2)-S(1)-O(1)	119.73(17)
O(2)-S(1)-C(6)	109.47(18)
O(2)-S(1)-C(7)	110.08(18)
C(7)-S(1)-C(6)	103.93(18)
C(2)-C(1)-Bi(1)	122.3(3)
C(2)-C(1)-C(6)	117.2(3)
C(6)-C(1)-Bi(1)	120.2(3)
C(3)-C(2)-C(1)	120.1(4)
C(2)-C(3)-C(13)	120.8(4)
C(4)-C(3)-C(2)	121.9(4)
C(4)-C(3)-C(13)	117.3(4)
C(3)-C(4)-C(5)	119.0(4)
C(6)-C(5)-C(4)	118.5(4)
C(1)-C(6)-S(1)	118.1(3)
C(5)-C(6)-S(1)	118.6(3)
C(5)-C(6)-C(1)	123.3(3)
C(8)-C(7)-S(1)	118.8(3)
C(8)-C(7)-C(12)	122.9(4)
C(12)-C(7)-S(1)	118.3(3)
C(9)-C(8)-C(7)	118.6(4)
C(10)-C(9)-C(8)	119.7(4)
C(9)-C(10)-C(11)	121.2(4)
C(12)-C(11)-C(10)	120.1(4)
C(7)-C(12)-Bi(1)	120.5(3)
C(11)-C(12)-Bi(1)	122.0(3)
C(11)-C(12)-C(7)	117.5(4)
F(1)-C(13)-F(2)	108.1(4)
F(1)-C(13)-F(3)	105.9(3)
F(1)-C(13)-C(3)	113.5(3)
F(2)-C(13)-F(3)	105.0(3)
F(2)-C(13)-C(3)	112.6(3)

F(3)-C(13)-C(3)	111.2(3)
C(15)-C(14)-Bi(1)	166.1(4)
C(14)-C(15)-C(16)	176.1(4)
C(17)-C(16)-C(15)	119.9(4)
C(21)-C(16)-C(15)	121.9(4)
C(21)-C(16)-C(17)	118.1(4)
C(18)-C(17)-C(16)	120.8(4)
C(17)-C(18)-C(19)	121.2(4)
C(18)-C(19)-C(20)	118.4(4)
C(18)-C(19)-C(22)	121.0(4)
C(20)-C(19)-C(22)	120.6(4)
C(21)-C(20)-C(19)	121.1(4)
C(20)-C(21)-C(16)	120.4(4)

X-Ray Crystallographic Analysis of 10-phenyl-10H-dibenzo[b,e][1,4]thiabismine 5,5-dioxide 26

The single-crystal X-ray diffraction studies were carried out on a Bruker Apex II Ultra CCD diffractometer equipped with Mo Kα radiation (λ = 0.71073). Crystals of the subject compound were used as received. A 0.200 x 0.030 x 0.030 mm colorless crystal was mounted on a Cryoloop with Paratone oil. Data were collected in a nitrogen gas stream at 100(2) K using ϕ and ϖ scans. Crystal-to-detector distance was 50 mm using exposure time 4s with a scan width of 0.70°. Data collection was 99.9% complete to 25.242° in θ. A total of 23456 reflections were collected covering the indices, -30<=h<=29, -17<=k<=17, -6<=l<=6. 3614 reflections were found to be symmetry independent, with a R_{int} of 0.0393. Indexing and unit cell refinement indicated a Primitive, Orthorhombic lattice. The space group was found to be $Pna2_1$. The data were integrated using the Bruker SAINT Software program and scaled using the SADABS software program. Solution by direct methods (SHELXT) produced a complete phasing model consistent with the proposed structure. All nonhydrogen atoms were refined anisotropically by full-matrix least-squares (SHELXL-2014). All carbon bonded hydrogen atoms were placed using a riding model. Their positions were constrained relative to their parent atom using the appropriate HFIX command in SHELXL-2014.
Notes: Proposed structure in agreement with model derived from diffraction data. Minor twin component tried hkl5 but return to single domain integration

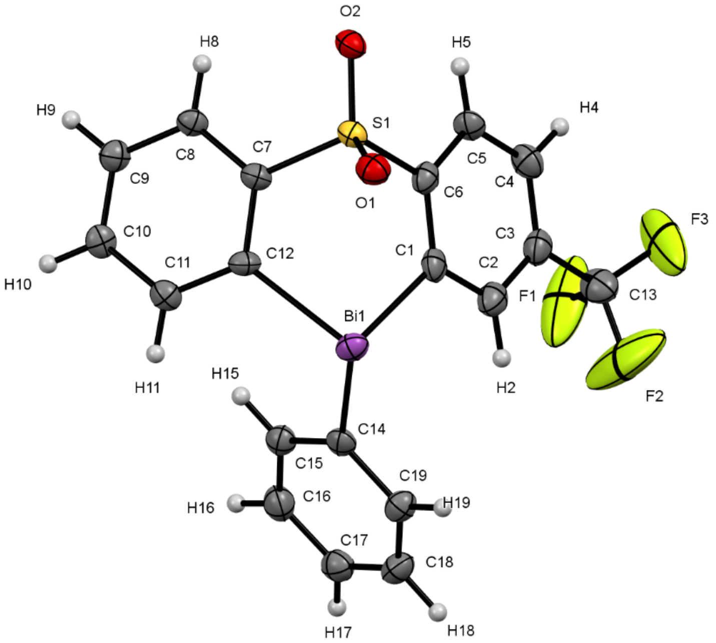

Figure S.8 Crystal structure of compound 26.

Table S.30 Crystal data and structure refinement for compound 26.

Empirical formula	C19 H12 Bi F3 O2 S
Molecular formula	C19 H12 Bi F3 O2 S
Formula weight	570.33
Temperature	100.0 K
Wavelength	0.71073 Å
Crystal system	Orthorhombic
Space group	Pna21

Unit cell dimensions	a = 24.2413(8) Å α = 90°.
	b = 14.1185(5) Å β = 90°.
	c = 5.1234(2) Å γ = 90°.
Volume	1753.49(11) Å3
Z	4
Density (calculated)	2.160 Mg/m3
Absorption coefficient	10.214 mm-1
F(000)	1072
Crystal size	0.2 x 0.03 x 0.03 mm3
Crystal color, habit	colorless plank
Theta range for data collection	1.669 to 26.437°.
Index ranges	-30<=h<=29, -17<=k<=17, -6<=l<=6
Reflections collected	23456
Independent reflections	3614 [R(int) = 0.0393]
Completeness to theta = 25.242°	99.9 %
Absorption correction	Semi-empirical from equivalents
Max. and min. transmission	0.0452 and 0.0204
Refinement method	Full-matrix least-squares on F2
Data / restraints / parameters	3614 / 37 / 235
Goodness-of-fit on F2	1.092
Final R indices [I>2sigma(I)]	R1 = 0.0259, wR2 = 0.0546
R indices (all data)	R1 = 0.0290, wR2 = 0.0556
Absolute structure parameter	-0.007(6)
Largest diff. peak and hole	1.276 and -2.343 e.Å-3

Table S.31 Bond lengths [Å] and angles [°] for compound 26.

Bi(1)-C(1)	2.292(9)
Bi(1)-C(12)	2.260(8)
Bi(1)-C(14)	2.273(9)
S(1)-O(1)	1.436(10)
S(1)-O(2)	1.439(6)
S(1)-C(6)	1.778(9)
S(1)-C(7)	1.752(8)
F(1)-C(13)	1.285(13)
F(2)-C(13)	1.308(13)
F(3)-C(13)	1.261(12)
C(1)-C(2)	1.390(12)
C(1)-C(6)	1.396(12)
C(2)-C(3)	1.398(15)
C(3)-C(4)	1.383(13)
C(3)-C(13)	1.501(13)
C(4)-C(5)	1.389(12)
C(5)-C(6)	1.395(12)
C(7)-C(8)	1.401(11)
C(7)-C(12)	1.398(11)
C(8)-C(9)	1.395(12)
C(9)-C(10)	1.383(12)

C(10)-C(11)	1.423(11)
C(11)-C(12)	1.395(12)
C(14)-C(15)	1.383(11)
C(14)-C(19)	1.398(11)
C(15)-C(16)	1.383(13)
C(16)-C(17)	1.367(13)
C(17)-C(18)	1.377(12)
C(18)-C(19)	1.390(12)
C(12)-Bi(1)-C(1)	86.4(3)
C(12)-Bi(1)-C(14)	97.7(3)
C(14)-Bi(1)-C(1)	89.3(3)
O(1)-S(1)-O(2)	118.8(3)
O(1)-S(1)-C(6)	105.7(3)
O(1)-S(1)-C(7)	107.7(4)
O(2)-S(1)-C(6)	109.9(4)
O(2)-S(1)-C(7)	109.9(4)
C(7)-S(1)-C(6)	103.7(4)
C(2)-C(1)-Bi(1)	121.9(7)
C(2)-C(1)-C(6)	117.5(8)
C(6)-C(1)-Bi(1)	120.6(6)
C(1)-C(2)-C(3)	120.1(8)
C(2)-C(3)-C(13)	119.2(8)
C(4)-C(3)-C(2)	121.2(8)
C(4)-C(3)-C(13)	119.6(10)
C(3)-C(4)-C(5)	119.9(9)
C(4)-C(5)-C(6)	118.1(8)
C(1)-C(6)-S(1)	117.4(7)
C(5)-C(6)-S(1)	119.5(7)
C(5)-C(6)-C(1)	123.1(8)
C(8)-C(7)-S(1)	118.9(6)
C(12)-C(7)-S(1)	118.9(6)
C(12)-C(7)-C(8)	122.2(8)
C(9)-C(8)-C(7)	119.1(8)
C(10)-C(9)-C(8)	120.2(8)
C(9)-C(10)-C(11)	120.0(8)
C(12)-C(11)-C(10)	120.6(8)
C(7)-C(12)-Bi(1)	120.1(6)
C(11)-C(12)-Bi(1)	122.1(6)
C(11)-C(12)-C(7)	117.9(8)
F(1)-C(13)-F(2)	103.0(11)
F(1)-C(13)-C(3)	111.6(10)
F(2)-C(13)-C(3)	113.2(9)
F(3)-C(13)-F(1)	107.4(10)
F(3)-C(13)-F(2)	106.1(11)
F(3)-C(13)-C(3)	114.7(9)
C(15)-C(14)-Bi(1)	125.0(6)
C(15)-C(14)-C(19)	119.5(8)

C(19)-C(14)-Bi(1)	115.4(6)
C(16)-C(15)-C(14)	119.3(9)
C(17)-C(16)-C(15)	121.7(11)
C(16)-C(17)-C(18)	119.2(10)
C(17)-C(18)-C(19)	120.5(8)
C(18)-C(19)-C(14)	119.6(8)

X-Ray Crystallographic Analysis of 10-phenyl-10H-dibenzo[b,e][1,4]thiabismine 5,5-dioxide 28

The single-crystal X-ray diffraction studies were carried out on a Bruker Kappa Apex II CCD diffractometer equipped with Mo Kα radiation (λ = 0.71073). A 0.180 x 0.150 x 0.130 mm colorless crystal was mounted on a Cryoloop with Paratone oil. Data were collected in a nitrogen gas stream at 100(2) K using ϕ and ϖ scans. Crystal-to-detector distance was 40 mm using exposure time 2s with a scan width of 0.70°. Data collection was 99.9% complete to 25.242° in θ. A total of 13836 reflections were collected covering the indices, -12<=h<=11, -12<=k<=9, -13<=l<=10. 3650 reflections were found to be symmetry independent, with a Rint of 0.0323. Indexing and unit cell refinement indicated a Primitive, Triclinic lattice. The space group was found to be *P*-1. The data were integrated using the Bruker SAINT Software program and scaled using the SADABS software program. Solution by direct methods (SHELXT) produced a complete phasing model consistent with the proposed structure. All nonhydrogen atoms were refined anisotropically by full-matrix least-squares (SHELXL-2014). All carbon bonded hydrogen atoms were placed using a riding model. Their positions were constrained relative to their parent atom using the appropriate HFIX command in SHELXL-2014.

Notes: Proposed structure in agreement with model derived from diffraction data. Excellent data and refinement

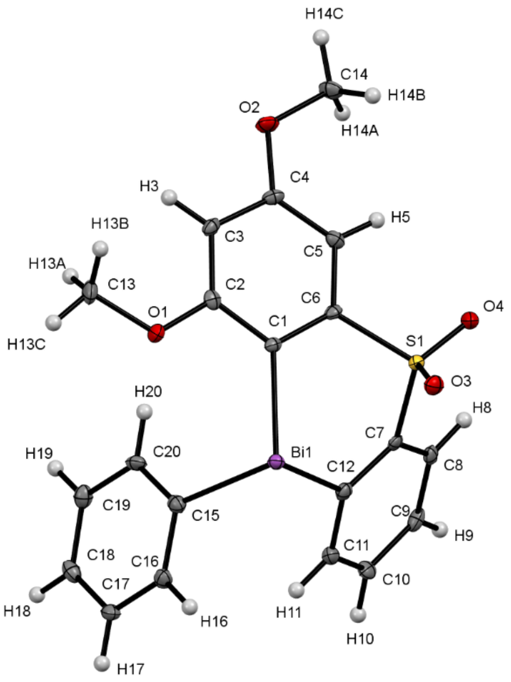

Figure S.219 Crystal structure of compound 28.

Table S.32 Crystal data and structure refinement for compound 28.

Empirical formula	C20 H17 Bi O4 S
Molecular formula	C20 H17 Bi O4 S
Formula weight	562.38
Temperature	100.0 K
Wavelength	0.71073 Å

Crystal system	Triclinic
Space group	P-1
Unit cell dimensions	a = 9.9992(7) Å α = 69.723(2)°.
	b = 10.2711(7) Å β = 86.066(2)°.
	c = 10.5288(7) Å γ = 62.662(2)°.
Volume	895.70(11) Å3
Z	2
Density (calculated)	2.085 Mg/m3
Absorption coefficient	9.982 mm-1
F(000)	536
Crystal size	0.175 x 0.035 x 0.02 mm3
Crystal color, habit	colorless plank
Theta range for data collection	2.074 to 26.372°.
Index ranges	-12<=h<=11, -12<=k<=9, -13<=l<=10
Reflections collected	13836
Independent reflections	3650 [R(int) = 0.0323]
Completeness to theta = 25.242°	99.9 %
Absorption correction	Semi-empirical from equivalents
Max. and min. transmission	0.2607 and 0.1758
Refinement method	Full-matrix least-squares on F2
Data / restraints / parameters	3650 / 0 / 237
Goodness-of-fit on F2	1.050
Final R indices [I>2sigma(I)]	R1 = 0.0164, wR2 = 0.0359
R indices (all data)	R1 = 0.0183, wR2 = 0.0364
Largest diff. peak and hole	0.638 and -0.661 e.Å-3

Table S.33 Bond lengths [Å] and angles [°] for compound 28.

Bi(1)-C(1)	2.263(3)
Bi(1)-C(12)	2.288(3)
Bi(1)-C(15)	2.265(3)
S(1)-O(3)	1.450(2)
S(1)-O(4)	1.441(2)
S(1)-C(6)	1.778(3)
S(1)-C(7)	1.767(3)
O(1)-C(2)	1.370(3)
O(1)-C(13)	1.439(3)
O(2)-C(4)	1.366(3)
O(2)-C(14)	1.439(3)
C(1)-C(2)	1.399(4)
C(1)-C(6)	1.392(4)
C(2)-C(3)	1.387(4)
C(3)-H(3)	0.9500
C(3)-C(4)	1.401(4)
C(4)-C(5)	1.392(4)
C(5)-H(5)	0.9500
C(5)-C(6)	1.387(4)
C(7)-C(8)	1.395(4)

C(7)-C(12)	1.395(4)
C(8)-H(8)	0.9500
C(8)-C(9)	1.384(4)
C(9)-H(9)	0.9500
C(9)-C(10)	1.389(4)
C(10)-H(10)	0.9500
C(10)-C(11)	1.382(4)
C(11)-H(11)	0.9500
C(11)-C(12)	1.390(4)
C(13)-H(13A)	0.9800
C(13)-H(13B)	0.9800
C(13)-H(13C)	0.9800
C(14)-H(14A)	0.9800
C(14)-H(14B)	0.9800
C(14)-H(14C)	0.9800
C(15)-C(16)	1.390(4)
C(15)-C(20)	1.390(4)
C(16)-H(16)	0.9500
C(16)-C(17)	1.394(4)
C(17)-H(17)	0.9500
C(17)-C(18)	1.384(4)
C(18)-H(18)	0.9500
C(18)-C(19)	1.385(4)
C(19)-H(19)	0.9500
C(19)-C(20)	1.400(4)
C(20)-H(20)	0.9500
C(1)-Bi(1)-C(12)	87.99(10)
C(1)-Bi(1)-C(15)	97.72(10)
C(15)-Bi(1)-C(12)	87.27(10)
O(3)-S(1)-C(6)	106.45(13)
O(3)-S(1)-C(7)	107.06(13)
O(4)-S(1)-O(3)	118.80(12)
O(4)-S(1)-C(6)	109.07(13)
O(4)-S(1)-C(7)	109.00(13)
C(7)-S(1)-C(6)	105.69(13)
C(2)-O(1)-C(13)	117.8(2)
C(4)-O(2)-C(14)	117.0(2)
C(2)-C(1)-Bi(1)	121.2(2)
C(6)-C(1)-Bi(1)	122.2(2)
C(6)-C(1)-C(2)	116.3(2)
O(1)-C(2)-C(1)	114.3(2)
O(1)-C(2)-C(3)	123.8(3)
C(3)-C(2)-C(1)	121.8(3)
C(2)-C(3)-H(3)	120.4
C(2)-C(3)-C(4)	119.2(3)
C(4)-C(3)-H(3)	120.4
O(2)-C(4)-C(3)	114.6(2)

O(2)-C(4)-C(5)	124.4(3)
C(5)-C(4)-C(3)	121.0(3)
C(4)-C(5)-H(5)	121.4
C(6)-C(5)-C(4)	117.3(3)
C(6)-C(5)-H(5)	121.4
C(1)-C(6)-S(1)	118.3(2)
C(5)-C(6)-S(1)	117.1(2)
C(5)-C(6)-C(1)	124.2(3)
C(8)-C(7)-S(1)	118.9(2)
C(8)-C(7)-C(12)	122.6(3)
C(12)-C(7)-S(1)	118.5(2)
C(7)-C(8)-H(8)	120.8
C(9)-C(8)-C(7)	118.4(3)
C(9)-C(8)-H(8)	120.8
C(8)-C(9)-H(9)	120.1
C(8)-C(9)-C(10)	119.8(3)
C(10)-C(9)-H(9)	120.1
C(9)-C(10)-H(10)	119.6
C(11)-C(10)-C(9)	120.9(3)
C(11)-C(10)-H(10)	119.6
C(10)-C(11)-H(11)	119.6
C(10)-C(11)-C(12)	120.8(3)
C(12)-C(11)-H(11)	119.6
C(7)-C(12)-Bi(1)	121.4(2)
C(11)-C(12)-Bi(1)	121.1(2)
C(11)-C(12)-C(7)	117.3(3)
O(1)-C(13)-H(13A)	109.5
O(1)-C(13)-H(13B)	109.5
O(1)-C(13)-H(13C)	109.5
H(13A)-C(13)-H(13B)	109.5
H(13A)-C(13)-H(13C)	109.5
H(13B)-C(13)-H(13C)	109.5
O(2)-C(14)-H(14A)	109.5
O(2)-C(14)-H(14B)	109.5
O(2)-C(14)-H(14C)	109.5
H(14A)-C(14)-H(14B)	109.5
H(14A)-C(14)-H(14C)	109.5
H(14B)-C(14)-H(14C)	109.5
C(16)-C(15)-Bi(1)	115.8(2)
C(20)-C(15)-Bi(1)	124.6(2)
C(20)-C(15)-C(16)	118.9(3)
C(15)-C(16)-H(16)	119.8
C(15)-C(16)-C(17)	120.5(3)
C(17)-C(16)-H(16)	119.8
C(16)-C(17)-H(17)	119.9
C(18)-C(17)-C(16)	120.2(3)
C(18)-C(17)-H(17)	119.9
C(17)-C(18)-H(18)	120.0

C(17)-C(18)-C(19)	120.0(3)
C(19)-C(18)-H(18)	120.0
C(18)-C(19)-H(19)	120.2
C(18)-C(19)-C(20)	119.6(3)
C(20)-C(19)-H(19)	120.2
C(15)-C(20)-C(19)	120.7(3)
C(15)-C(20)-H(20)	119.7
C(19)-C(20)-H(20)	119.7

X-Ray Crystallographic Analysis of 10-iodo-10H-dibenzo[b,e][1,4]thiabismine 5,5-dioxide 33

The single-crystal X-ray diffraction studies were carried out on a Bruker Apex II Ultra CCD diffractometer equipped with Mo Kα radiation (λ = 0.71073). A 0.140 x 0.025 x 0.025 mm colorless crystal was mounted on a Cryoloop with Paratone oil. Data were collected in a nitrogen gas stream at 100(2) K using ϕ and ϖ scans. Crystal-to-detector distance was 60 mm using exposure time 3s with a scan width of 0.60°. Data collection was 99.9% complete to 25.242° in θ. A total of 14363 reflections were collected covering the indices, -11<=h<=11, -6<=k<=6, -44<=l<=29. 3213 reflections were found to be symmetry independent, with a Rint of 0.0204. Indexing and unit cell refinement indicated a Primitive, Monoclinic lattice. The space group was found to be P21/n. The data were integrated using the Bruker SAINT Software program and scaled using the SADABS software program. Solution by direct methods (SHELXT) produced a complete phasing model consistent with the proposed structure. All nonhydrogen atoms were refined anisotropically by full-matrix least-squares (SHELXL-2014). All carbon bonded hydrogen atoms were placed using a riding model. Their positions were constrained relative to their parent atom using the appropriate HFIX command in SHELXL-2014.
Notes: Proposed structure in agreement with model derived from diffraction data. Excellent data and refinement

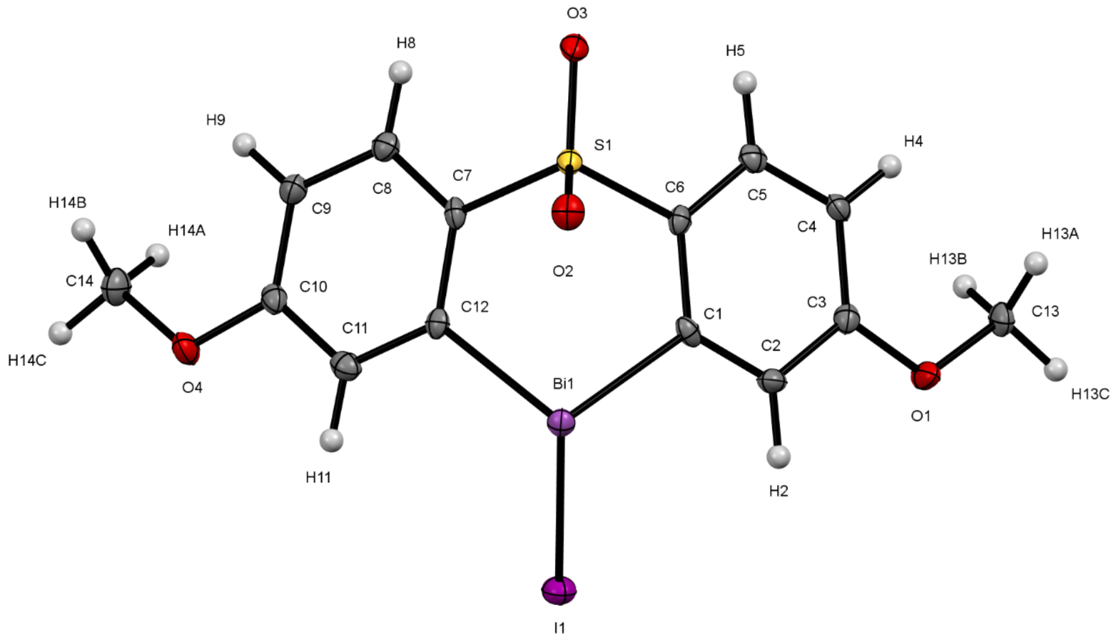

Figure S.22 Crystal structure of compound 33.

Table S.34 Crystal data and structure refinement for compound 33.

Empirical formula	C14 H12 Bi I O4 S
Molecular formula	C14 H12 Bi I O4 S
Formula weight	612.18
Temperature	100.0 K
Wavelength	0.71073 Å
Crystal system	Monoclinic
Space group	P 1 21/n 1

Unit cell dimensions	a = 9.1914(6) Å α = 90°.
	b = 4.8207(3) Å β = 91.7600(10)°.
	c = 35.739(2) Å γ = 90°.
Volume	1582.82(18) Å3
Z	4
Density (calculated)	2.569 Mg/m3
Absorption coefficient	13.232 mm-1
F(000)	1120
Crystal size	0.14 x 0.025 x 0.025 mm3
Crystal color, habit	colorless plank
Theta range for data collection	2.272 to 26.372°.
Index ranges	-11<=h<=11, -6<=k<=6, -44<=l<=29
Reflections collected	14363
Independent reflections	3213 [R(int) = 0.0204]
Completeness to theta = 25.242°	99.9 %
Absorption correction	Semi-empirical from equivalents
Max. and min. transmission	0.2607 and 0.1503
Refinement method	Full-matrix least-squares on F2
Data / restraints / parameters	3213 / 0 / 192
Goodness-of-fit on F2	1.251
Final R indices [I>2sigma(I)]	R1 = 0.0186, wR2 = 0.0395
R indices (all data)	R1 = 0.0190, wR2 = 0.0396
Largest diff. peak and hole	0.855 and -1.101 e.Å-3

Table S.35 Bond lengths [Å] and angles [°] for compound 33.

Bi(1)-I(1)	2.8715(3)
Bi(1)-C(1)	2.275(4)
Bi(1)-C(12)	2.270(4)
S(1)-O(2)	1.459(3)
S(1)-O(3)	1.433(3)
S(1)-C(6)	1.763(4)
S(1)-C(7)	1.761(4)
O(1)-C(3)	1.363(4)
O(1)-C(13)	1.433(4)
O(4)-C(10)	1.364(4)
O(4)-C(14)	1.434(5)
C(1)-C(2)	1.379(5)
C(1)-C(6)	1.398(5)
C(2)-H(2)	0.9500
C(2)-C(3)	1.407(5)
C(3)-C(4)	1.392(5)
C(4)-H(4)	0.9500
C(4)-C(5)	1.394(5)
C(5)-H(5)	0.9500
C(5)-C(6)	1.384(5)
C(7)-C(8)	1.383(5)
C(7)-C(12)	1.400(5)

C(8)-H(8)	0.9500
C(8)-C(9)	1.395(5)
C(9)-H(9)	0.9500
C(9)-C(10)	1.391(5)
C(10)-C(11)	1.402(5)
C(11)-H(11)	0.9500
C(11)-C(12)	1.384(5)
C(13)-H(13A)	0.9800
C(13)-H(13B)	0.9800
C(13)-H(13C)	0.9800
C(14)-H(14A)	0.9800
C(14)-H(14B)	0.9800
C(14)-H(14C)	0.9800

C(1)-Bi(1)-I(1)	91.33(9)
C(12)-Bi(1)-I(1)	93.48(9)
C(12)-Bi(1)-C(1)	87.54(13)
O(2)-S(1)-C(6)	104.41(17)
O(2)-S(1)-C(7)	104.26(17)
O(3)-S(1)-O(2)	119.25(16)
O(3)-S(1)-C(6)	110.53(17)
O(3)-S(1)-C(7)	111.58(17)
C(7)-S(1)-C(6)	105.74(17)
C(3)-O(1)-C(13)	118.3(3)
C(10)-O(4)-C(14)	117.4(3)
C(2)-C(1)-Bi(1)	123.4(3)
C(2)-C(1)-C(6)	118.7(3)
C(6)-C(1)-Bi(1)	117.9(3)
C(1)-C(2)-H(2)	120.2
C(1)-C(2)-C(3)	119.6(3)
C(3)-C(2)-H(2)	120.2
O(1)-C(3)-C(2)	114.5(3)
O(1)-C(3)-C(4)	124.2(3)
C(4)-C(3)-C(2)	121.3(3)
C(3)-C(4)-H(4)	120.6
C(3)-C(4)-C(5)	118.9(3)
C(5)-C(4)-H(4)	120.6
C(4)-C(5)-H(5)	120.3
C(6)-C(5)-C(4)	119.4(3)
C(6)-C(5)-H(5)	120.3
C(1)-C(6)-S(1)	116.9(3)
C(5)-C(6)-S(1)	121.0(3)
C(5)-C(6)-C(1)	122.1(3)
C(8)-C(7)-S(1)	121.0(3)
C(8)-C(7)-C(12)	122.2(3)
C(12)-C(7)-S(1)	116.8(3)
C(7)-C(8)-H(8)	120.4
C(7)-C(8)-C(9)	119.2(3)

C(9)-C(8)-H(8)	120.4
C(8)-C(9)-H(9)	120.4
C(10)-C(9)-C(8)	119.2(3)
C(10)-C(9)-H(9)	120.4
O(4)-C(10)-C(9)	123.9(3)
O(4)-C(10)-C(11)	115.0(3)
C(9)-C(10)-C(11)	121.1(3)
C(10)-C(11)-H(11)	120.1
C(12)-C(11)-C(10)	119.8(3)
C(12)-C(11)-H(11)	120.1
C(7)-C(12)-Bi(1)	117.9(3)
C(11)-C(12)-Bi(1)	123.6(3)
C(11)-C(12)-C(7)	118.5(3)
O(1)-C(13)-H(13A)	109.5
O(1)-C(13)-H(13B)	109.5
O(1)-C(13)-H(13C)	109.5
H(13A)-C(13)-H(13B)	109.5
H(13A)-C(13)-H(13C)	109.5
H(13B)-C(13)-H(13C)	109.5
O(4)-C(14)-H(14A)	109.5
O(4)-C(14)-H(14B)	109.5
O(4)-C(14)-H(14C)	109.5
H(14A)-C(14)-H(14B)	109.5
H(14A)-C(14)-H(14C)	109.5
H(14B)-C(14)-H(14C)	109.5

5. ^{1}H, ^{13}C and ^{19}F NMR Spectra

Parameter	Value
Title	PROTON_01
Comment	AN-5
Origin	Varian
Owner	nazarova
Instrument	vnmrs
Solvent	cdcl3
Temperature	25.0
Pulse Sequence	s2pul
Experiment	1D
Number of Scans	8
Receiver Gain	48
Relaxation Delay	1.0000
Pulse Width	3.7000
Spectrometer Frequency	499.76
Spectral Width	6009.6
Lowest Frequency	-512.0
Nucleus	1H
Acquired Size	16384
Spectral Size	65536

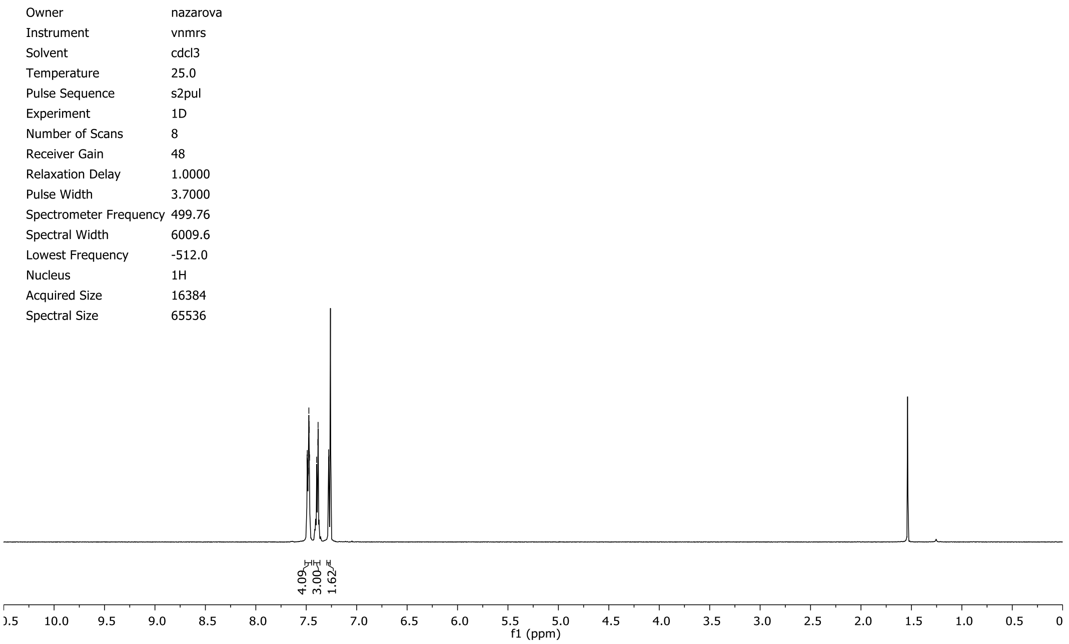

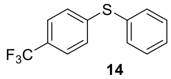

14

Parameter	Value
Title	CARBON_01
Comment	AN-5
Origin	Varian
Owner	nazarova
Instrument	vnmrs
Solvent	cdcl3
Temperature	25.0
Pulse Sequence	s2pul
Experiment	1D
Number of Scans	6336
Receiver Gain	30
Relaxation Delay	4.0000
Pulse Width	4.9500
Spectrometer Frequency	125.68
Spectral Width	31250.0
Lowest Frequency	-1781.2
Nucleus	13C
Acquired Size	32768
Spectral Size	65536

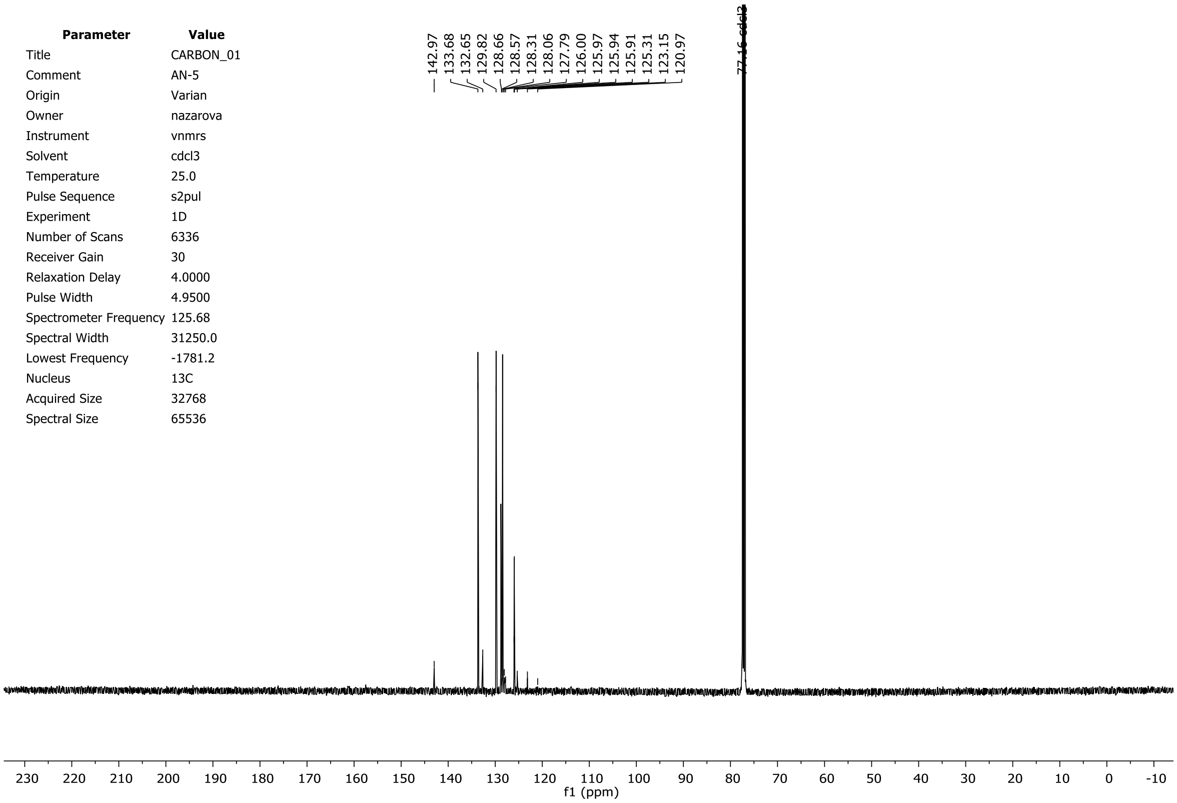

Parameter	Value
Title	FLUORINE_01
Comment	AN-5
Origin	Varian
Owner	nazarova
Instrument	vnmrs
Solvent	cdcl3
Temperature	25.0
Pulse Sequence	s2pul
Experiment	1D
Number of Scans	16
Receiver Gain	60
Relaxation Delay	1.0000
Pulse Width	3.3667
Spectrometer Frequency	470.21
Spectral Width	108695.7
Lowest Frequency	-94318.8
Nucleus	19F
Acquired Size	65536
Spectral Size	131072

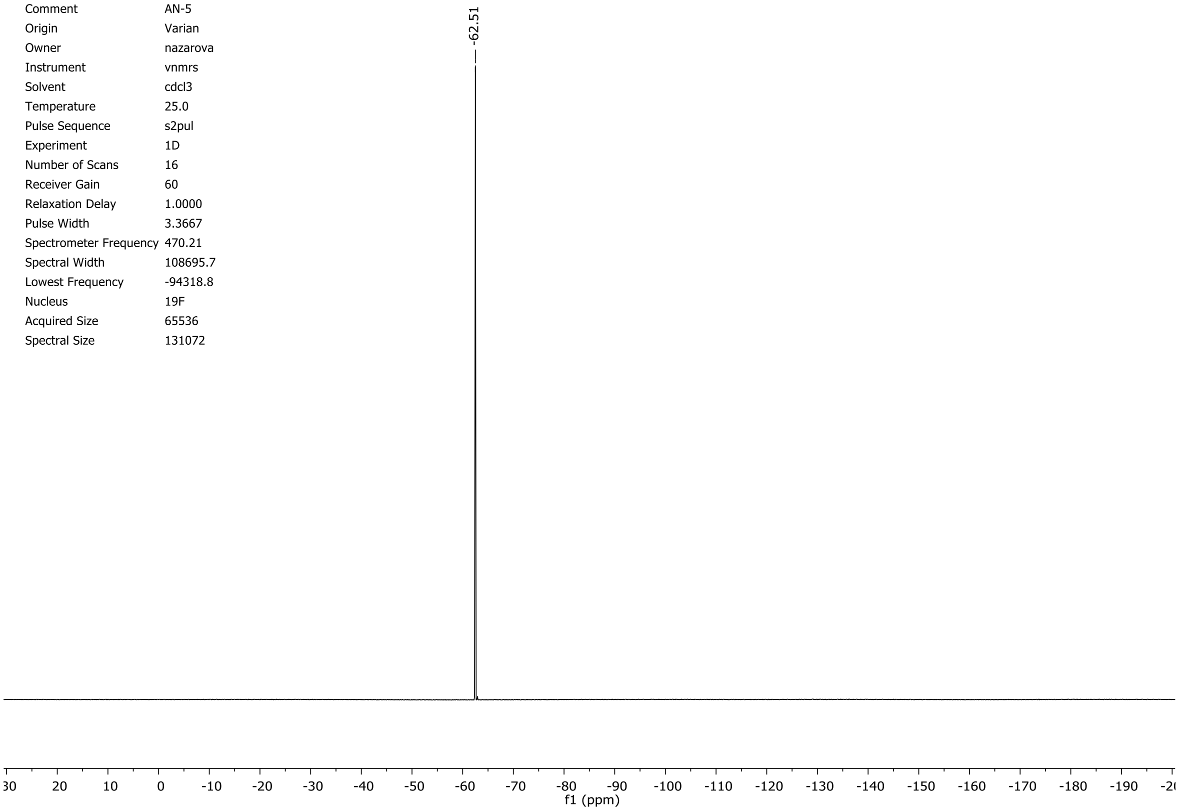

Parameter	Value
Title	PROTON_03
Comment	AN-12
Origin	Varian
Owner	nazarova
Instrument	vnmrs
Solvent	cdcl3
Temperature	25.0
Pulse Sequence	s2pul
Experiment	1D
Number of Scans	8
Receiver Gain	44
Relaxation Delay	2.0000
Pulse Width	2.4667
Spectrometer Frequency	499.76
Spectral Width	6009.6
Lowest Frequency	-511.5
Nucleus	1H
Acquired Size	16384
Spectral Size	65536

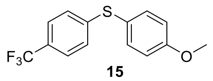

15

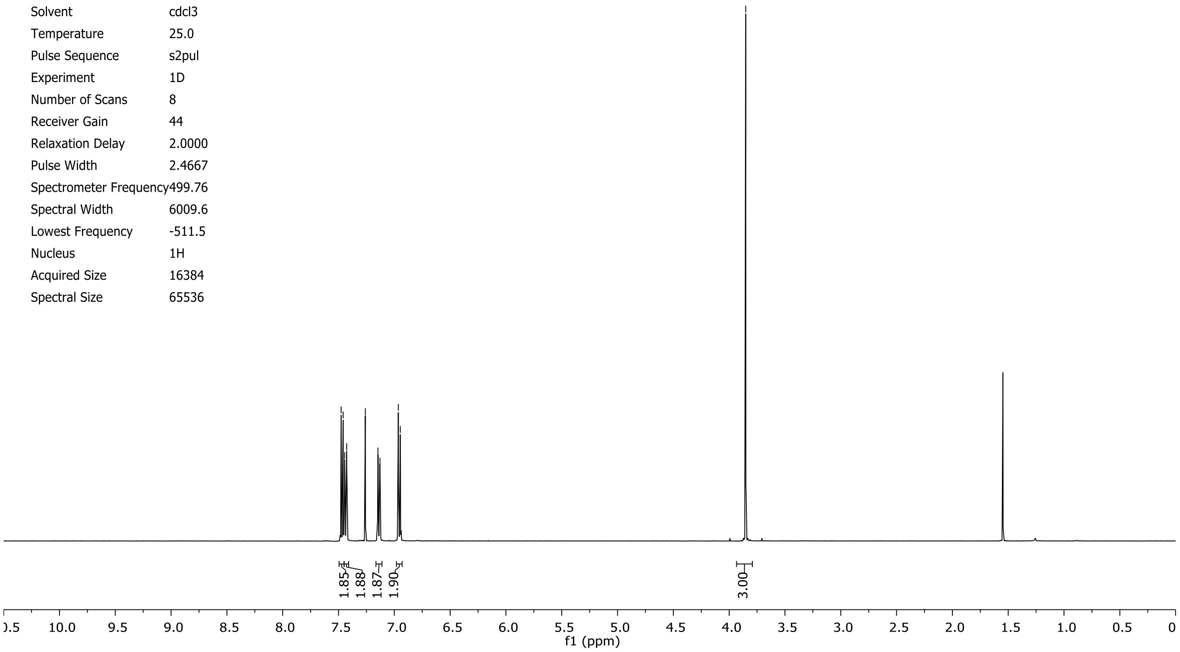

Parameter	Value
Title	CARBON_01
Comment	AN-12
Origin	Varian
Owner	nazarova
Instrument	vnmrs
Solvent	cdcl3
Temperature	25.0
Pulse Sequence	s2pul
Experiment	1D
Number of Scans	10752
Receiver Gain	30
Relaxation Delay	2.0000
Pulse Width	4.9500
Spectrometer Frequency	125.68
Spectral Width	31250.0
Lowest Frequency	-1781.7
Nucleus	13C
Acquired Size	32768
Spectral Size	65536

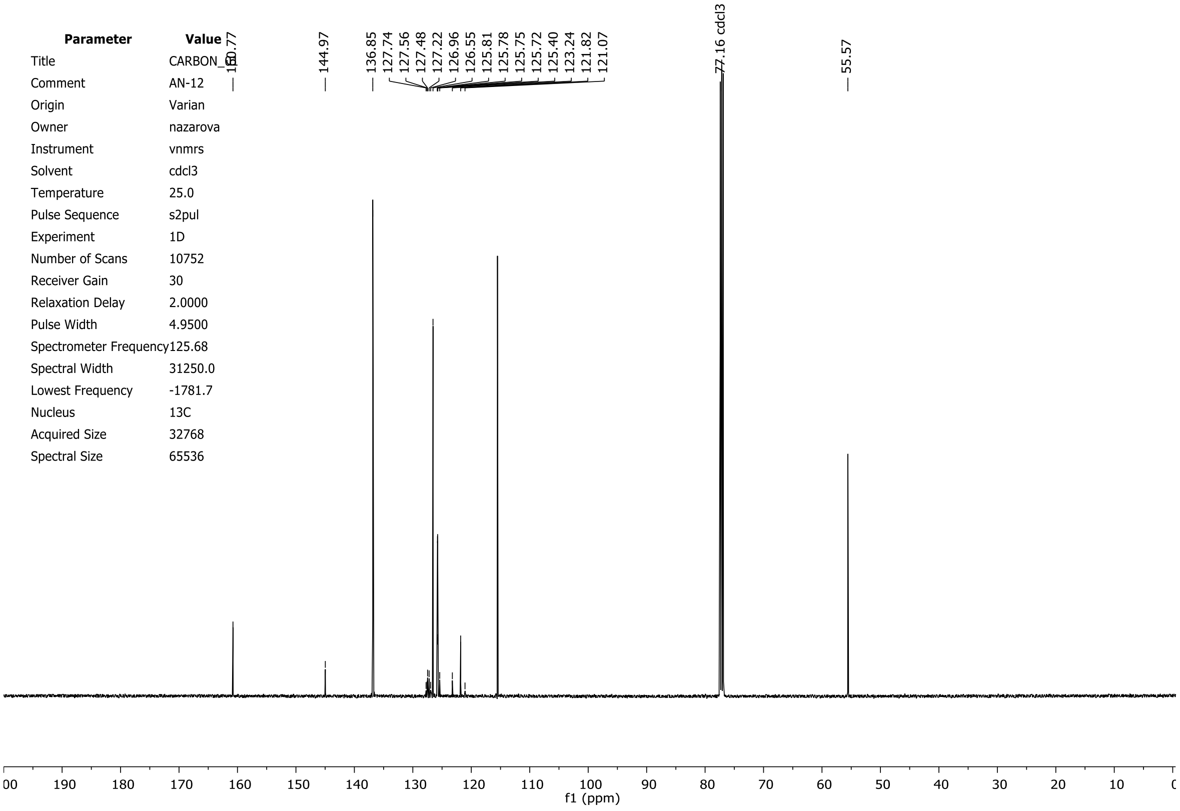

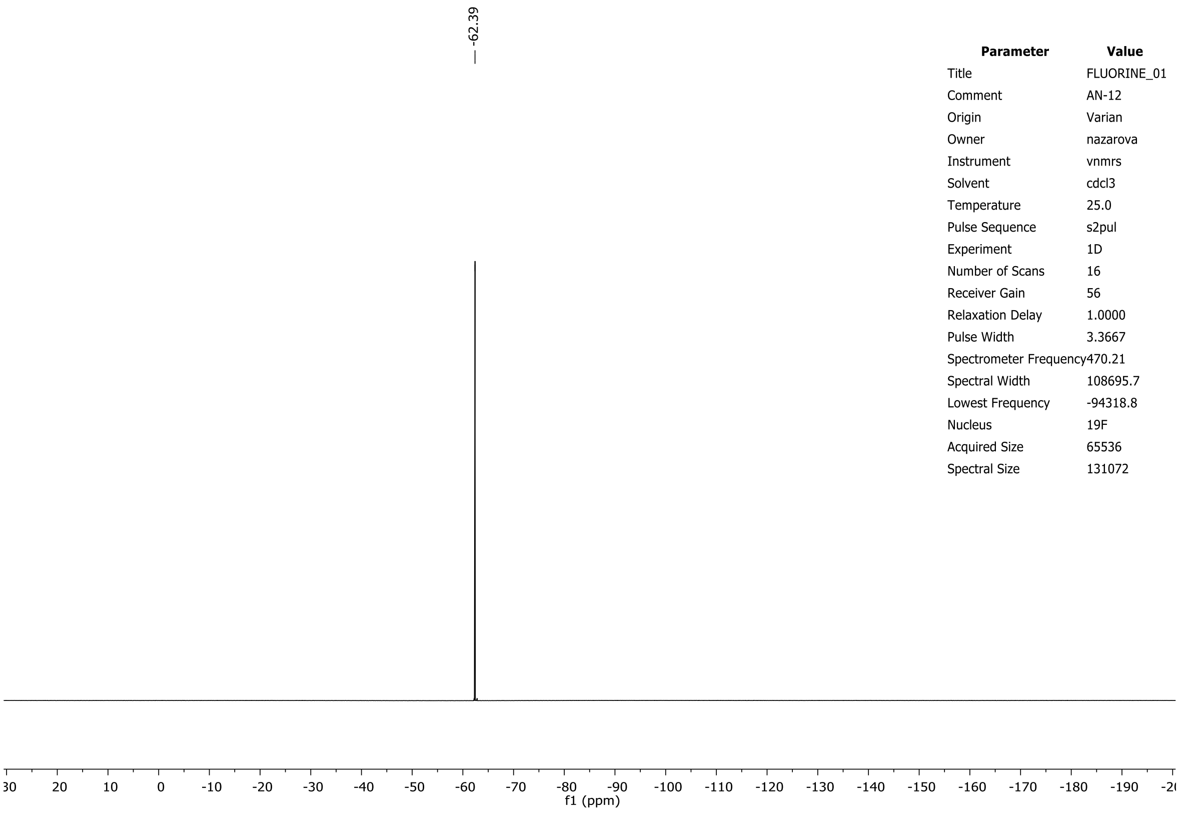

Parameter	Value
Title	FLUORINE_01
Comment	AN-12
Origin	Varian
Owner	nazarova
Instrument	vnmrs
Solvent	cdcl3
Temperature	25.0
Pulse Sequence	s2pul
Experiment	1D
Number of Scans	16
Receiver Gain	56
Relaxation Delay	1.0000
Pulse Width	3.3667
Spectrometer Frequency	470.21
Spectral Width	108695.7
Lowest Frequency	-94318.8
Nucleus	19F
Acquired Size	65536
Spectral Size	131072

Parameter	Value
Title	PROTON_02
Comment	AN-6
Origin	Varian
Owner	nazarova
Instrument	vnmrs
Solvent	cdcl3
Temperature	25.0
Pulse Sequence	s2pul
Experiment	1D
Number of Scans	8
Receiver Gain	18
Relaxation Delay	1.0000
Pulse Width	3.7000
Spectrometer Frequency	499.76
Spectral Width	6009.6
Lowest Frequency	-511.0
Nucleus	1H
Acquired Size	16384
Spectral Size	65536

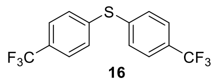

16

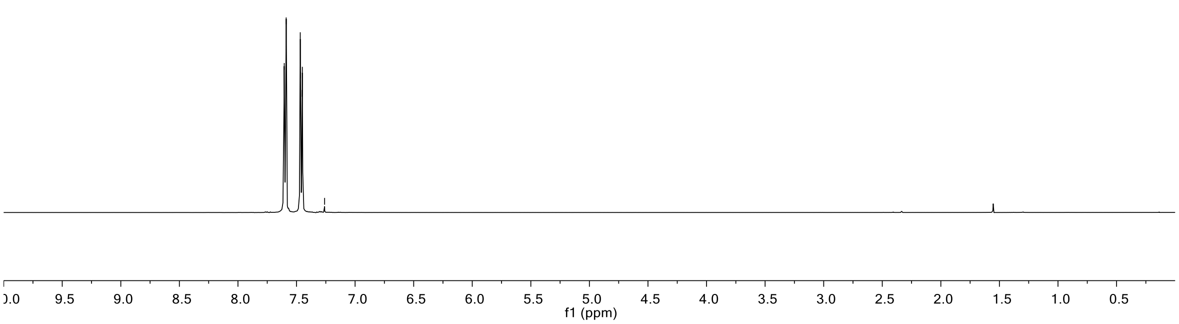

Parameter	Value
Title	CARBON_01
Comment	AN-6
Origin	Varian
Owner	nazarova
Instrument	vnmrs
Solvent	cdcl3
Temperature	25.0
Pulse Sequence	s2pul
Experiment	1D
Number of Scans	704
Receiver Gain	30
Relaxation Delay	2.0000
Pulse Width	4.9500
Spectrometer Frequency	125.68
Spectral Width	31250.0
Lowest Frequency	-1779.8
Nucleus	13C
Acquired Size	32768
Spectral Size	65536

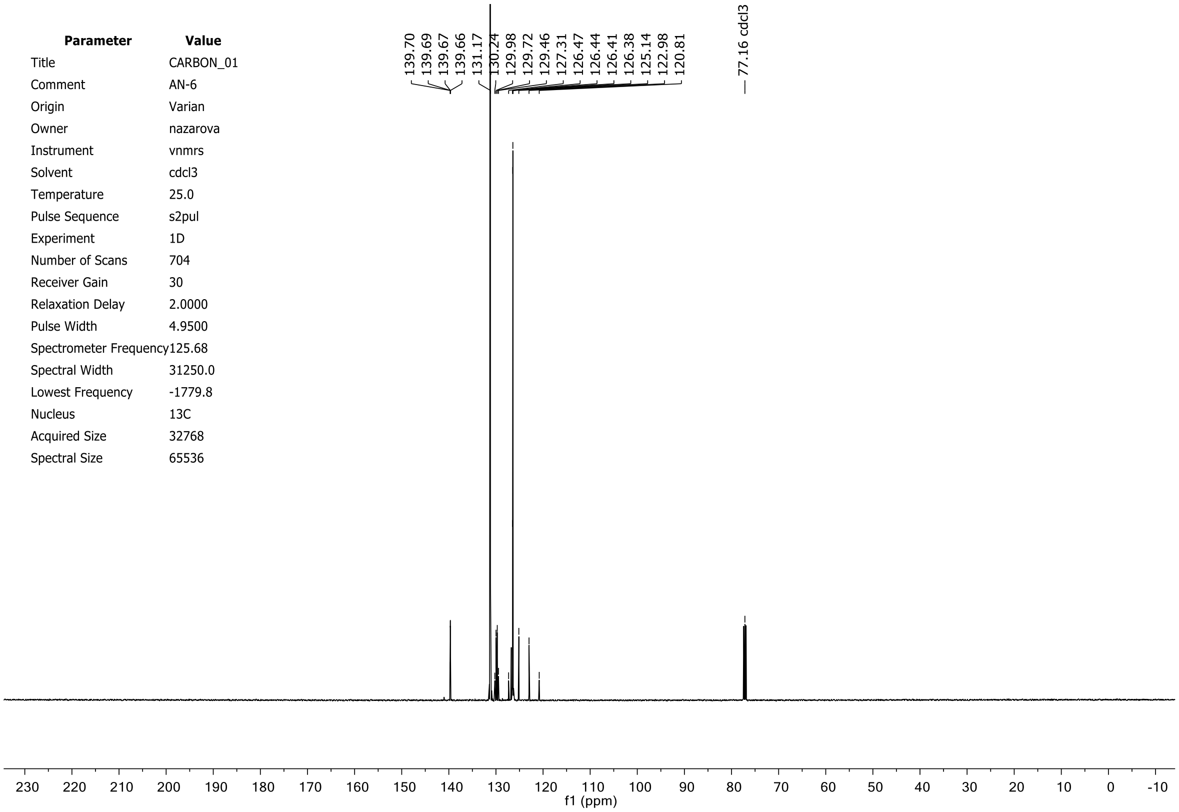

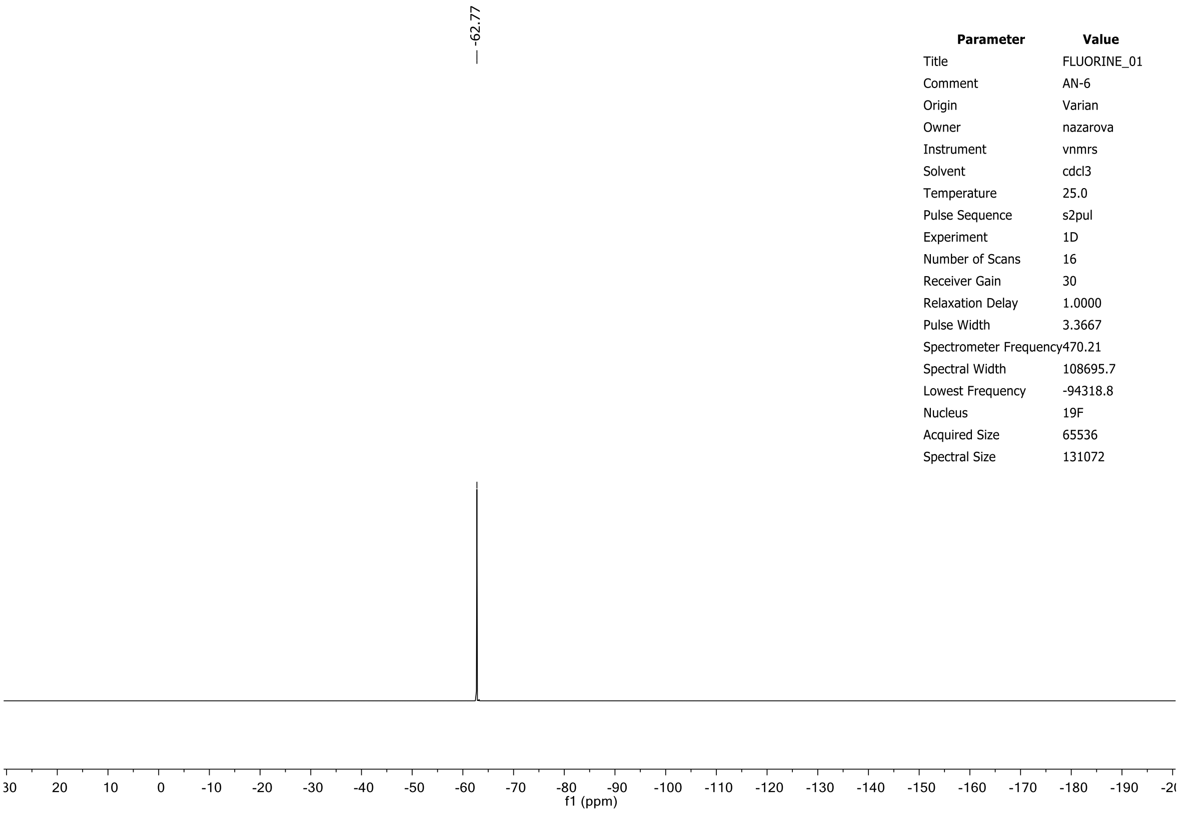

Parameter	Value
Title	FLUORINE_01
Comment	AN-6
Origin	Varian
Owner	nazarova
Instrument	vnmrs
Solvent	cdcl3
Temperature	25.0
Pulse Sequence	s2pul
Experiment	1D
Number of Scans	16
Receiver Gain	30
Relaxation Delay	1.0000
Pulse Width	3.3667
Spectrometer Frequency	470.21
Spectral Width	108695.7
Lowest Frequency	-94318.8
Nucleus	19F
Acquired Size	65536
Spectral Size	131072

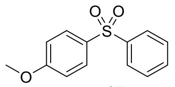

17

Parameter	Value
Title	PROTON_02
Comment	AN-7
Origin	Varian
Owner	nazarova
Instrument	vnmrs
Solvent	cdcl3
Temperature	25.0
Pulse Sequence	s2pul
Experiment	1D
Number of Scans	8
Receiver Gain	48
Relaxation Delay	5.0000
Pulse Width	3.7000
Spectrometer Frequency	499.76
Spectral Width	5000.0
Lowest Frequency	-6.2
Nucleus	1H
Acquired Size	16384
Spectral Size	65536

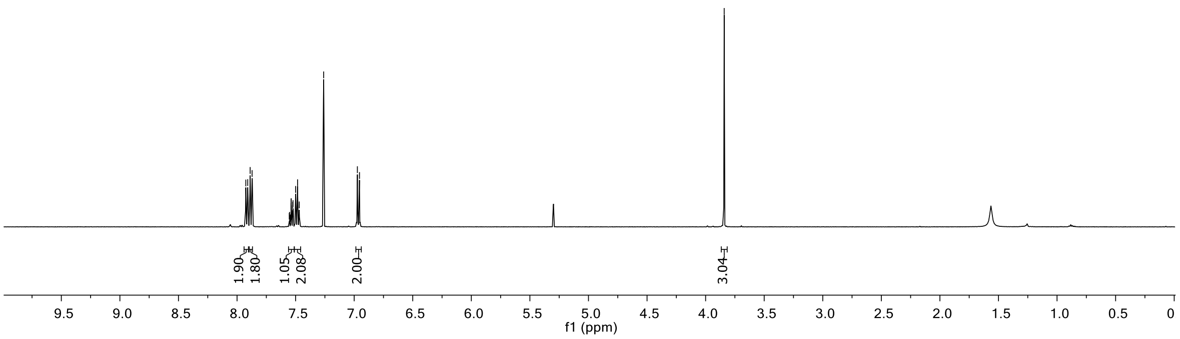

Parameter	Value
Title	CARBON_01
Comment	AN-7
Origin	Varian
Owner	nazarova
Instrument	vnmrs
Solvent	cdcl3
Temperature	25.0
Pulse Sequence	s2pul
Experiment	1D
Number of Scans	9984
Receiver Gain	30
Relaxation Delay	3.0000
Pulse Width	4.9500
Spectrometer Frequency	125.68
Spectral Width	31250.0
Lowest Frequency	-1781.8
Nucleus	13C
Acquired Size	32768
Spectral Size	65536

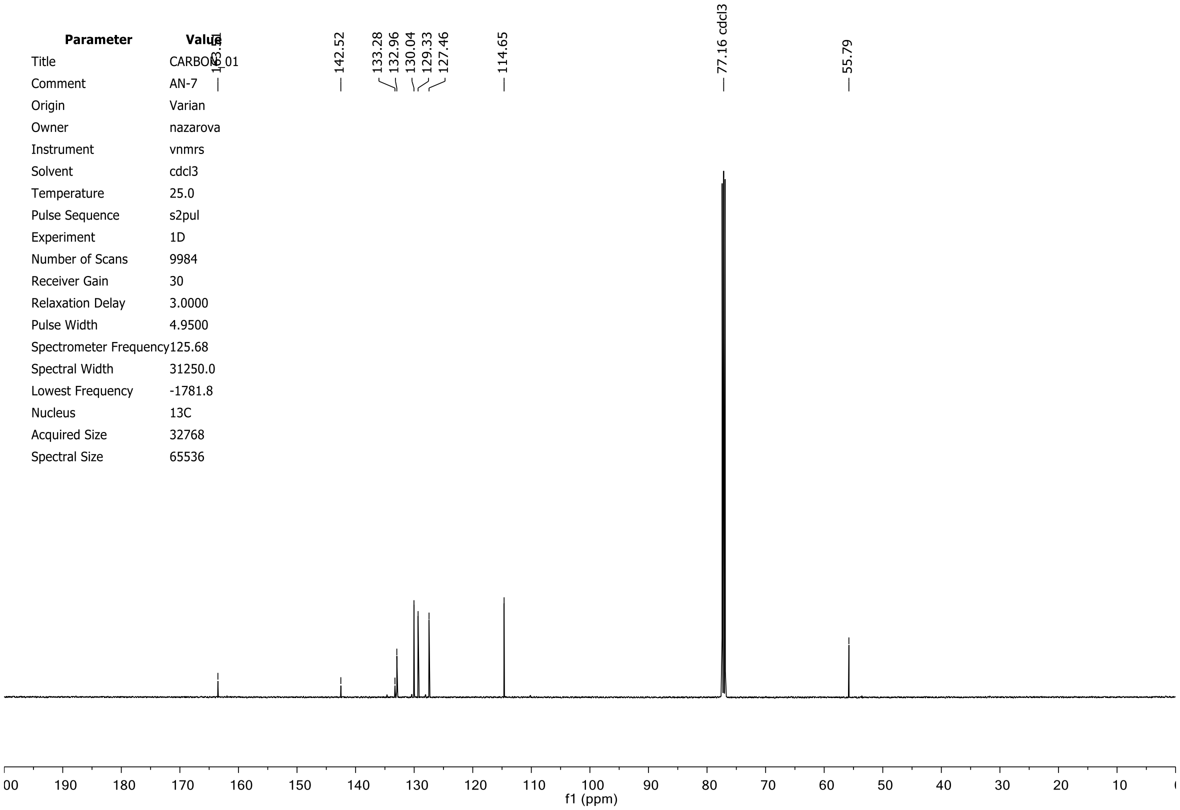

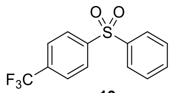

19

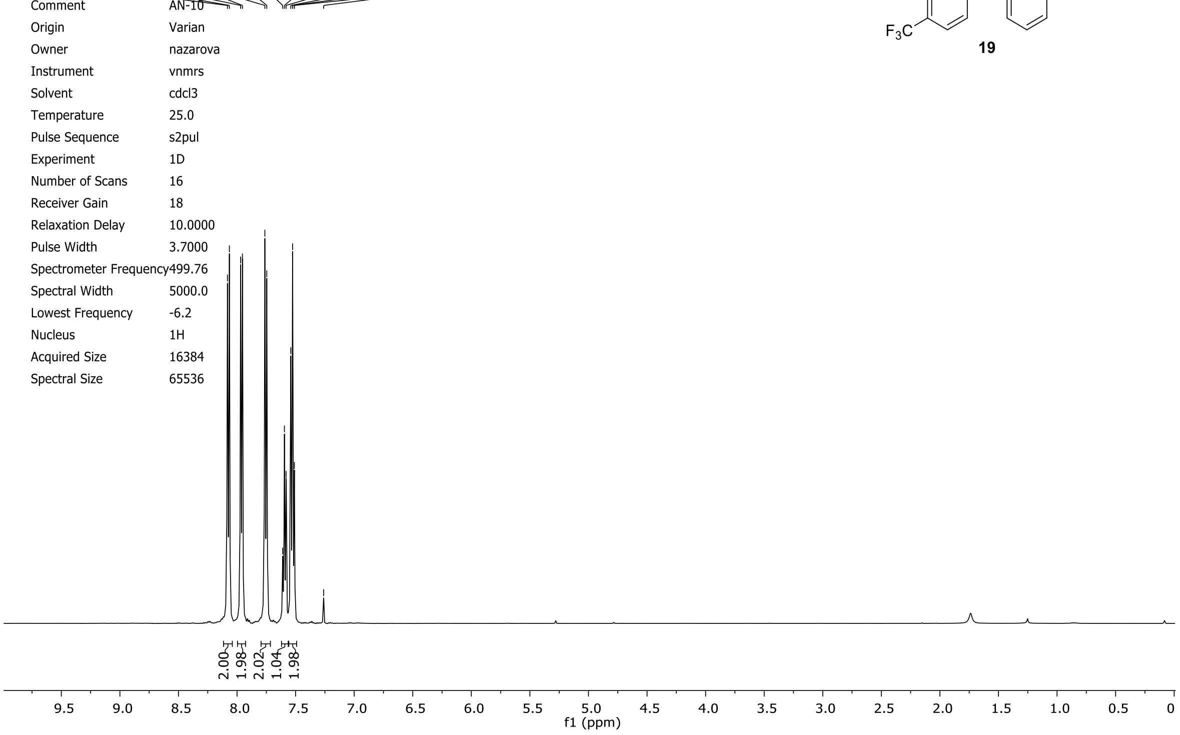

Parameter	Value
Title	PROTON_01
Comment	AN-10
Origin	Varian
Owner	nazarova
Instrument	vnmrs
Solvent	cdcl3
Temperature	25.0
Pulse Sequence	s2pul
Experiment	1D
Number of Scans	16
Receiver Gain	18
Relaxation Delay	10.0000
Pulse Width	3.7000
Spectrometer Frequency	499.76
Spectral Width	5000.0
Lowest Frequency	-6.2
Nucleus	1H
Acquired Size	16384
Spectral Size	65536

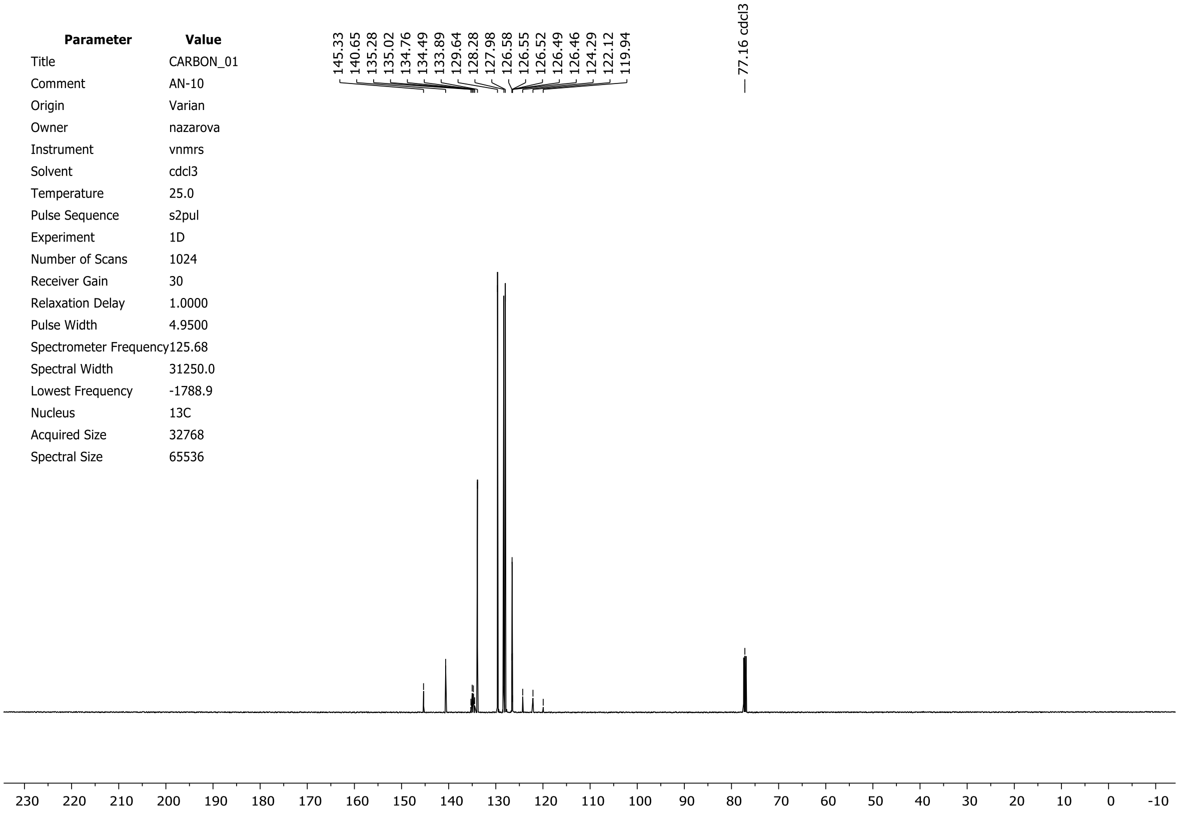

Parameter	Value
Title	CARBON_01
Comment	AN-10
Origin	Varian
Owner	nazarova
Instrument	vnmrs
Solvent	cdcl3
Temperature	25.0
Pulse Sequence	s2pul
Experiment	1D
Number of Scans	1024
Receiver Gain	30
Relaxation Delay	1.0000
Pulse Width	4.9500
Spectrometer Frequency	125.68
Spectral Width	31250.0
Lowest Frequency	-1788.9
Nucleus	13C
Acquired Size	32768
Spectral Size	65536

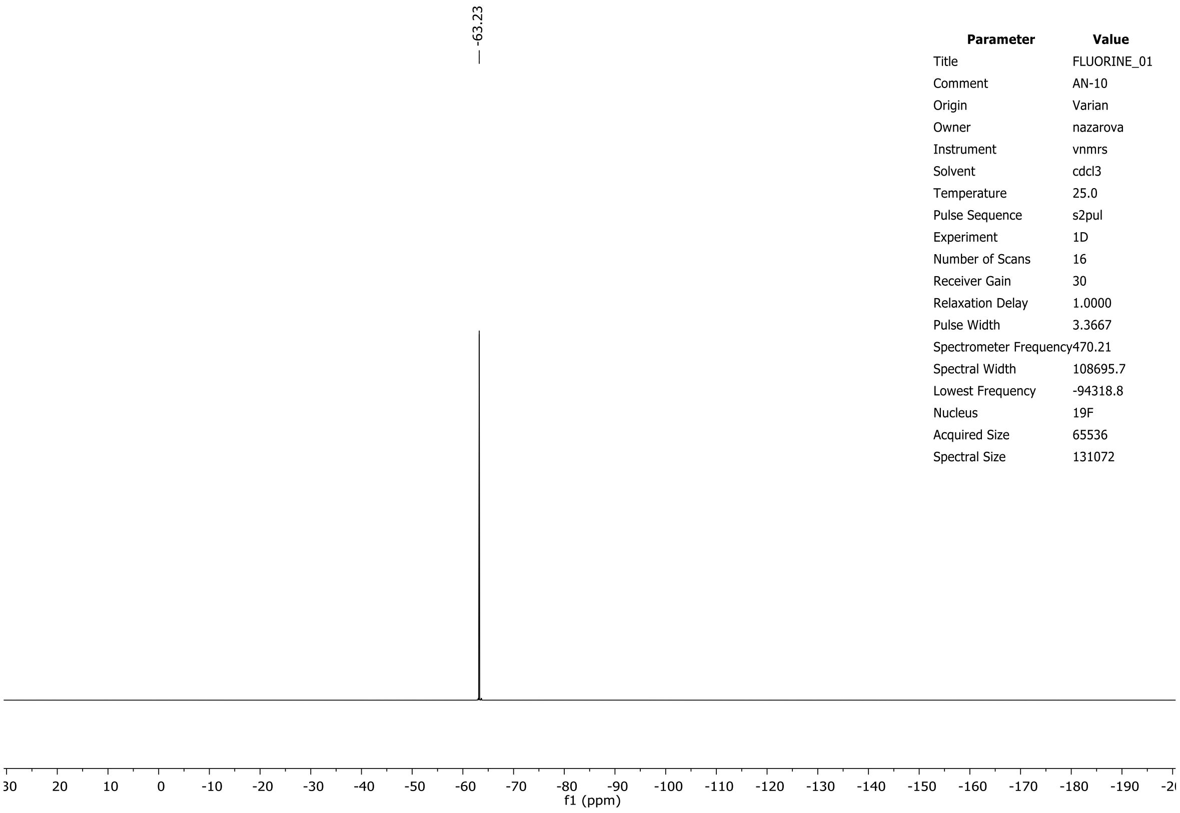

Parameter	Value
Title	FLUORINE_01
Comment	AN-10
Origin	Varian
Owner	nazarova
Instrument	vnmrs
Solvent	cdcl3
Temperature	25.0
Pulse Sequence	s2pul
Experiment	1D
Number of Scans	16
Receiver Gain	30
Relaxation Delay	1.0000
Pulse Width	3.3667
Spectrometer Frequency	470.21
Spectral Width	108695.7
Lowest Frequency	-94318.8
Nucleus	19F
Acquired Size	65536
Spectral Size	131072

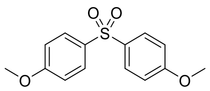

20

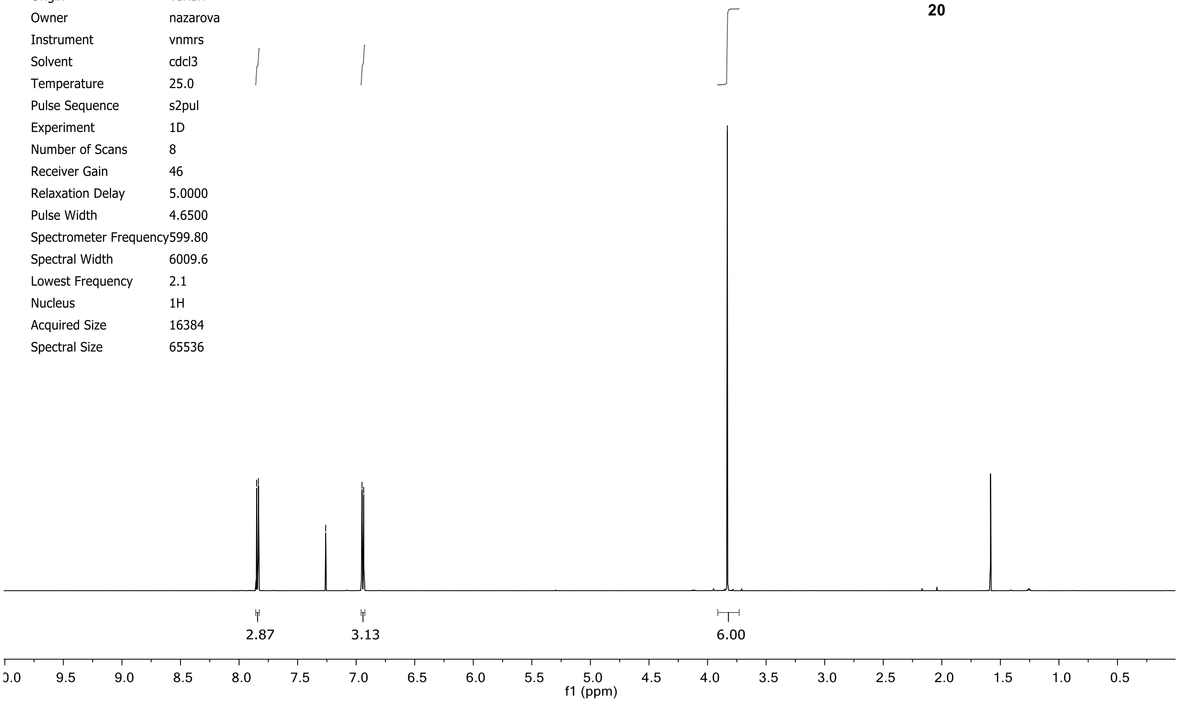

Parameter	Value
Title	PROTON_02
Comment	AN-11
Origin	Varian
Owner	nazarova
Instrument	vnmrs
Solvent	cdcl3
Temperature	25.0
Pulse Sequence	s2pul
Experiment	1D
Number of Scans	8
Receiver Gain	46
Relaxation Delay	5.0000
Pulse Width	4.6500
Spectrometer Frequency	599.80
Spectral Width	6009.6
Lowest Frequency	2.1
Nucleus	1H
Acquired Size	16384
Spectral Size	65536

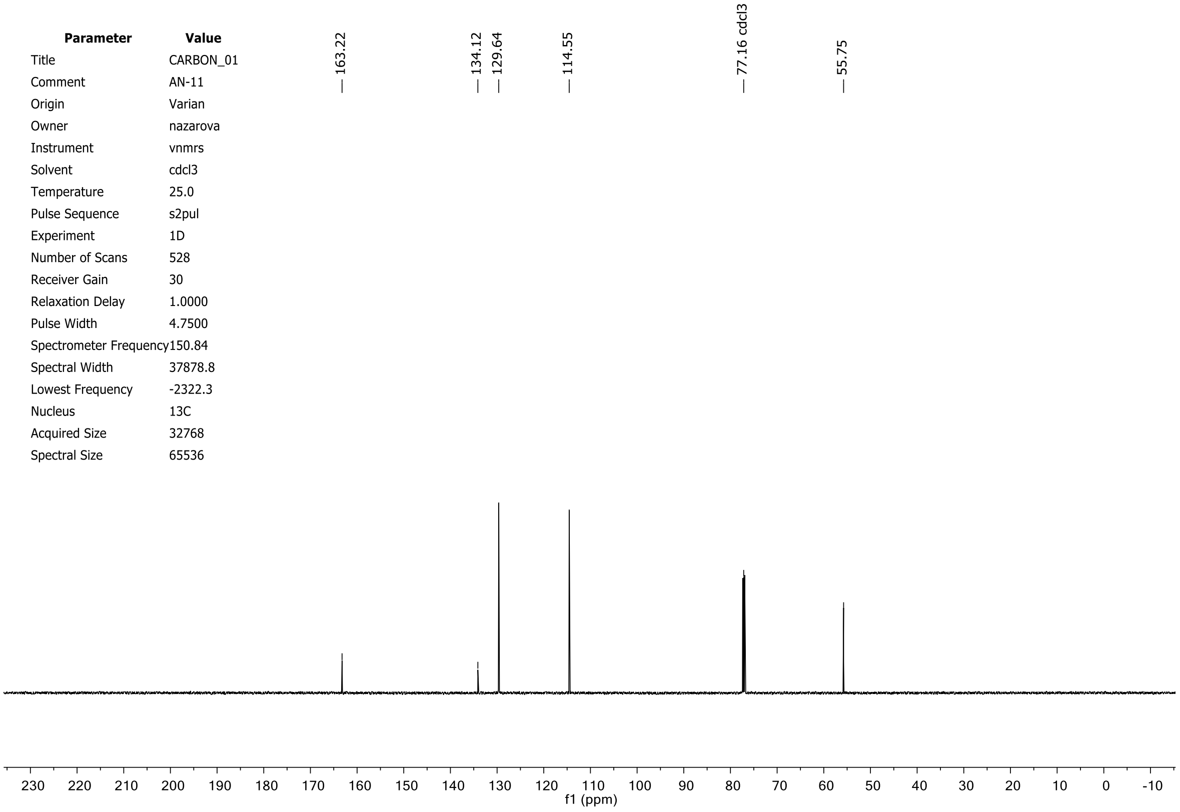

Parameter	Value
Title	CARBON_01
Comment	AN-11
Origin	Varian
Owner	nazarova
Instrument	vnmrs
Solvent	cdcl3
Temperature	25.0
Pulse Sequence	s2pul
Experiment	1D
Number of Scans	528
Receiver Gain	30
Relaxation Delay	1.0000
Pulse Width	4.7500
Spectrometer Frequency	150.84
Spectral Width	37878.8
Lowest Frequency	-2322.3
Nucleus	13C
Acquired Size	32768
Spectral Size	65536

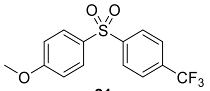

21

Parameter	Value
Title	PROTON_02
Comment	AN-12
Origin	Varian
Owner	nazarova
Instrument	vnmrs
Solvent	cdcl3
Temperature	25.0
Pulse Sequence	s2pul
Experiment	1D
Number of Scans	8
Receiver Gain	30
Relaxation Delay	10.0000
Pulse Width	3.7000
Spectrometer Frequency	499.76
Spectral Width	5000.0
Lowest Frequency	-6.2
Nucleus	1H
Acquired Size	16384
Spectral Size	65536

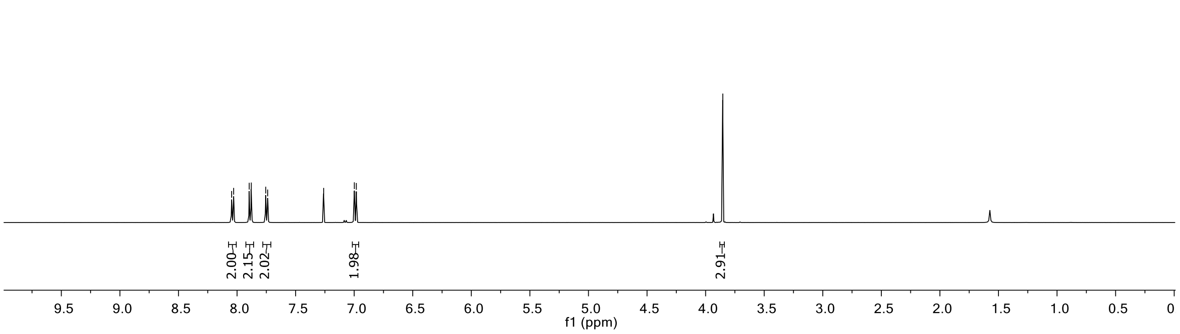

Parameter	Value
Title	CARBON_01
Comment	AN-12
Origin	Varian
Owner	nazarova
Instrument	vnmrs
Solvent	cdcl3
Temperature	25.0
Pulse Sequence	s2pul
Experiment	1D
Number of Scans	640
Receiver Gain	30
Relaxation Delay	2.0000
Pulse Width	4.9500
Spectrometer Frequency	125.68
Spectral Width	31250.0
Lowest Frequency	-1782.0
Nucleus	13C
Acquired Size	32768
Spectral Size	65536

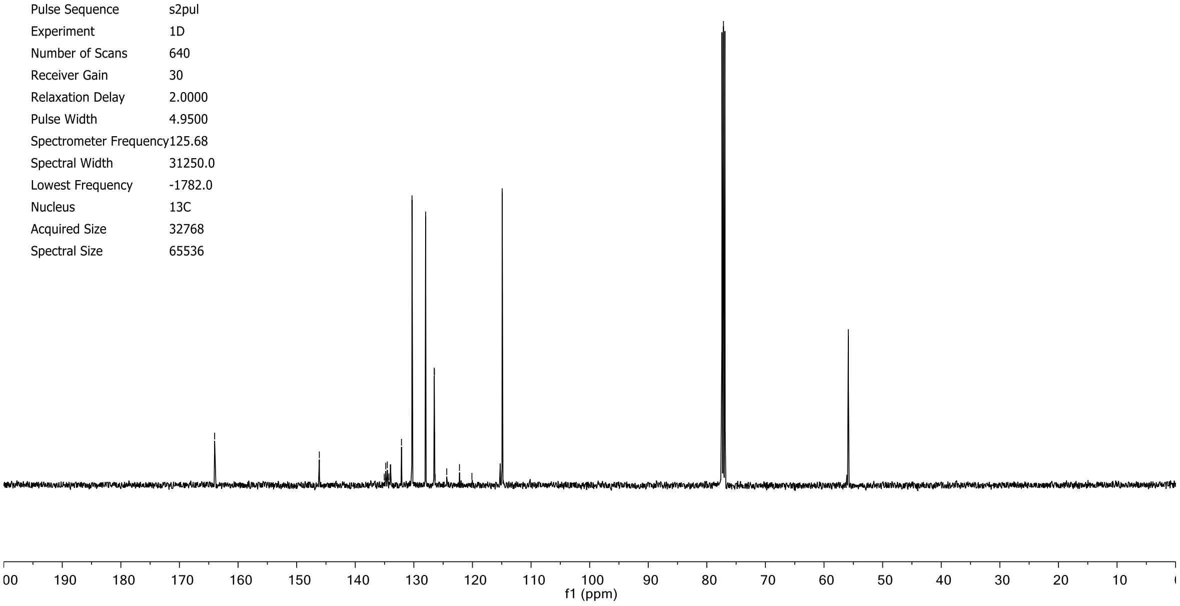

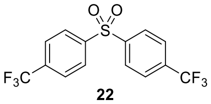

22

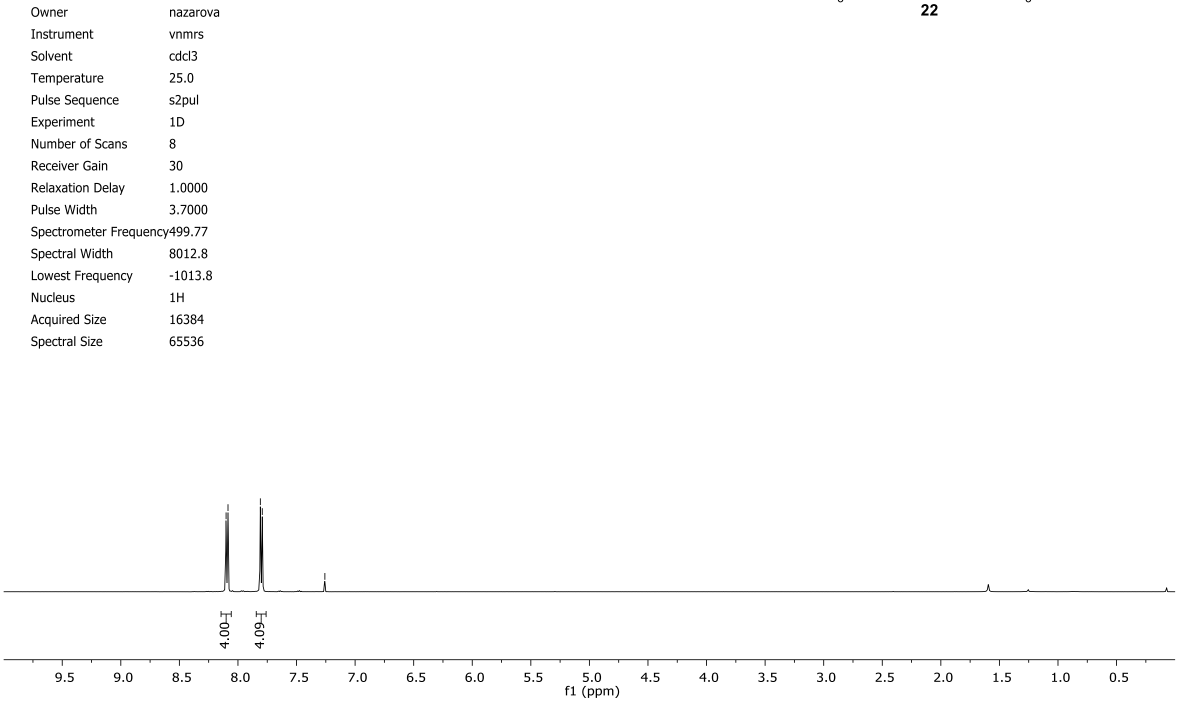

Parameter	Value
Title	PROTON_01
Comment	AN-13
Origin	Varian
Owner	nazarova
Instrument	vnmrs
Solvent	cdcl3
Temperature	25.0
Pulse Sequence	s2pul
Experiment	1D
Number of Scans	8
Receiver Gain	30
Relaxation Delay	1.0000
Pulse Width	3.7000
Spectrometer Frequency	499.77
Spectral Width	8012.8
Lowest Frequency	-1013.8
Nucleus	1H
Acquired Size	16384
Spectral Size	65536

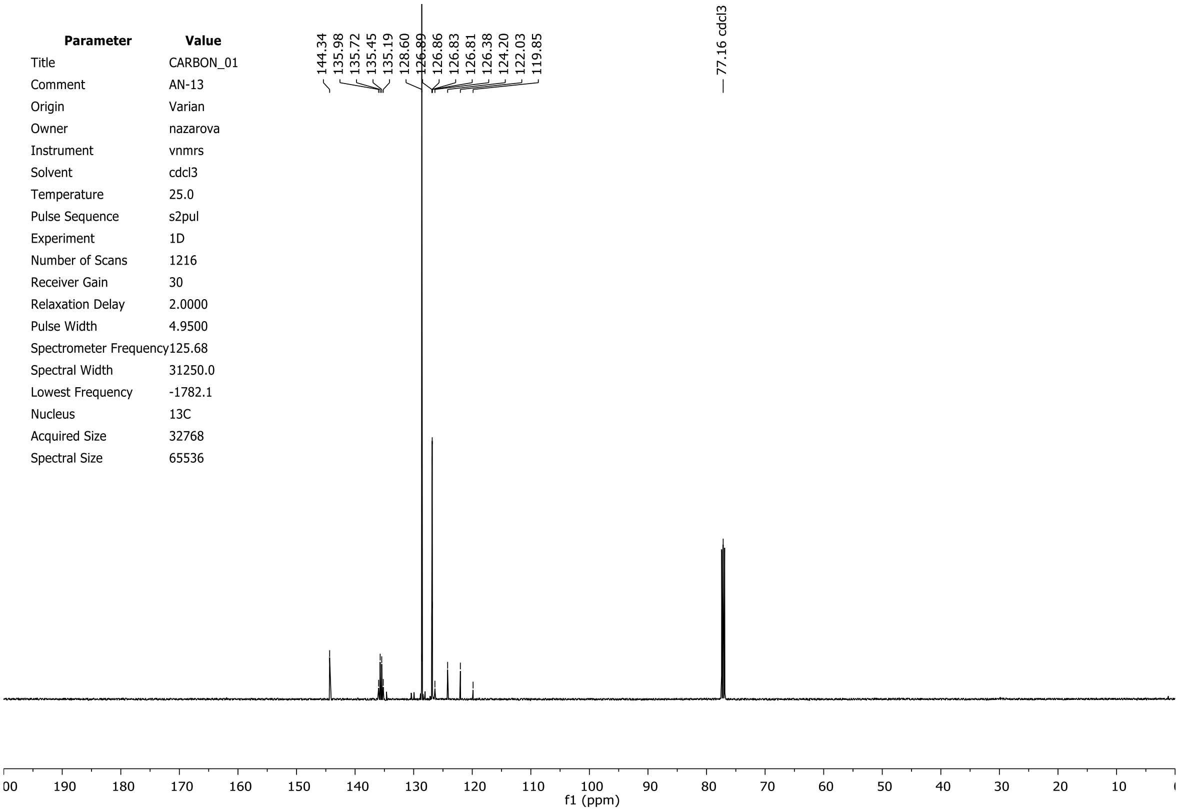

Parameter	Value
Title	CARBON_01
Comment	AN-13
Origin	Varian
Owner	nazarova
Instrument	vnmrs
Solvent	cdcl3
Temperature	25.0
Pulse Sequence	s2pul
Experiment	1D
Number of Scans	1216
Receiver Gain	30
Relaxation Delay	2.0000
Pulse Width	4.9500
Spectrometer Frequency	125.68
Spectral Width	31250.0
Lowest Frequency	-1782.1
Nucleus	13C
Acquired Size	32768
Spectral Size	65536

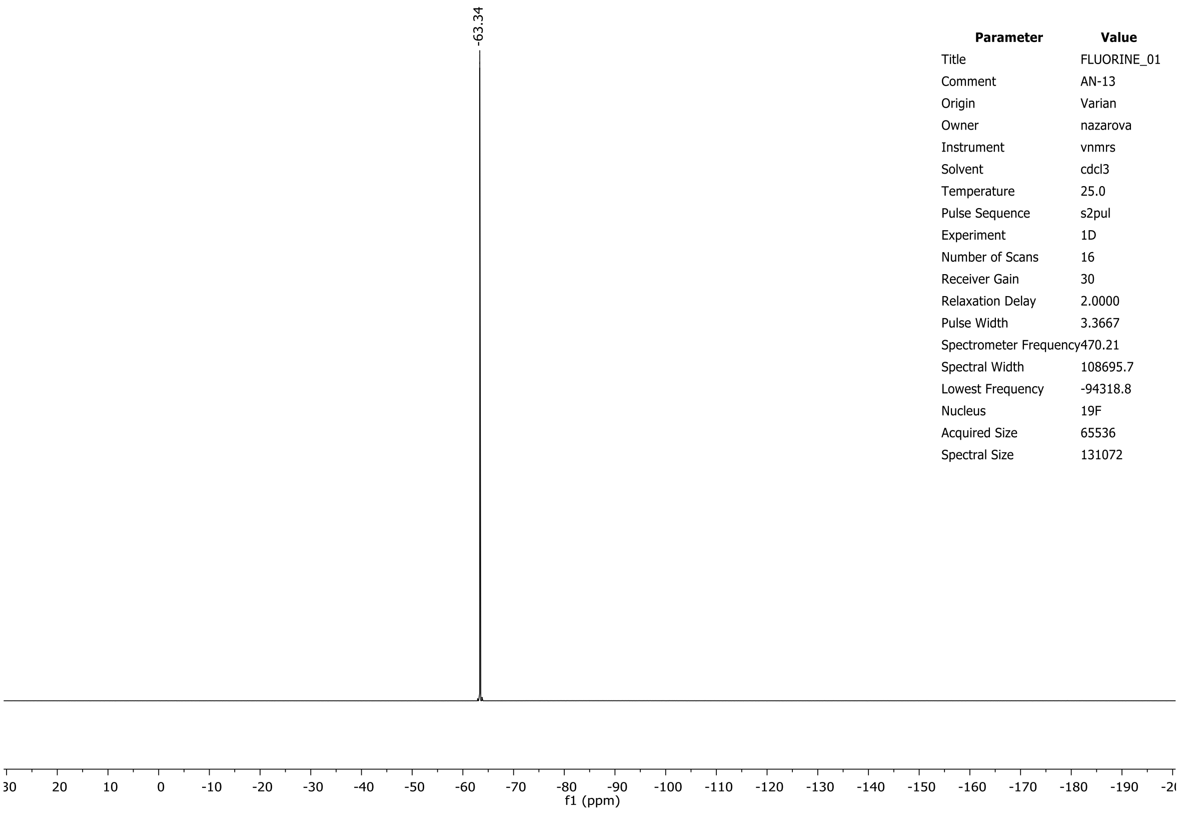

Parameter	Value
Title	FLUORINE_01
Comment	AN-13
Origin	Varian
Owner	nazarova
Instrument	vnmrs
Solvent	cdcl3
Temperature	25.0
Pulse Sequence	s2pul
Experiment	1D
Number of Scans	16
Receiver Gain	30
Relaxation Delay	2.0000
Pulse Width	3.3667
Spectrometer Frequency	470.21
Spectral Width	108695.7
Lowest Frequency	-94318.8
Nucleus	19F
Acquired Size	65536
Spectral Size	131072

Parameter	Value
Title	PROTON_01
Comment	AN-17
Origin	Varian
Owner	nazarova
Instrument	vnmrs
Solvent	cdcl3
Temperature	25.0
Pulse Sequence	s2pul
Experiment	1D
Number of Scans	8
Receiver Gain	48
Relaxation Delay	1.0000
Pulse Width	3.7000
Spectrometer Frequency	499.76
Spectral Width	5000.0
Lowest Frequency	-6.7
Nucleus	1H
Acquired Size	16384
Spectral Size	65536

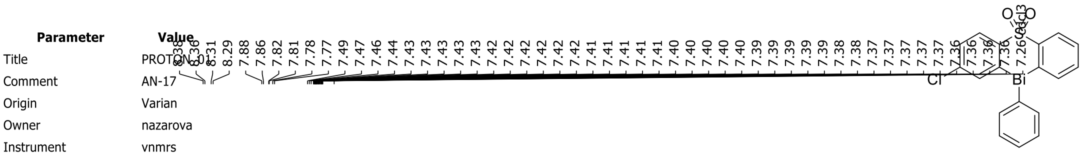

25

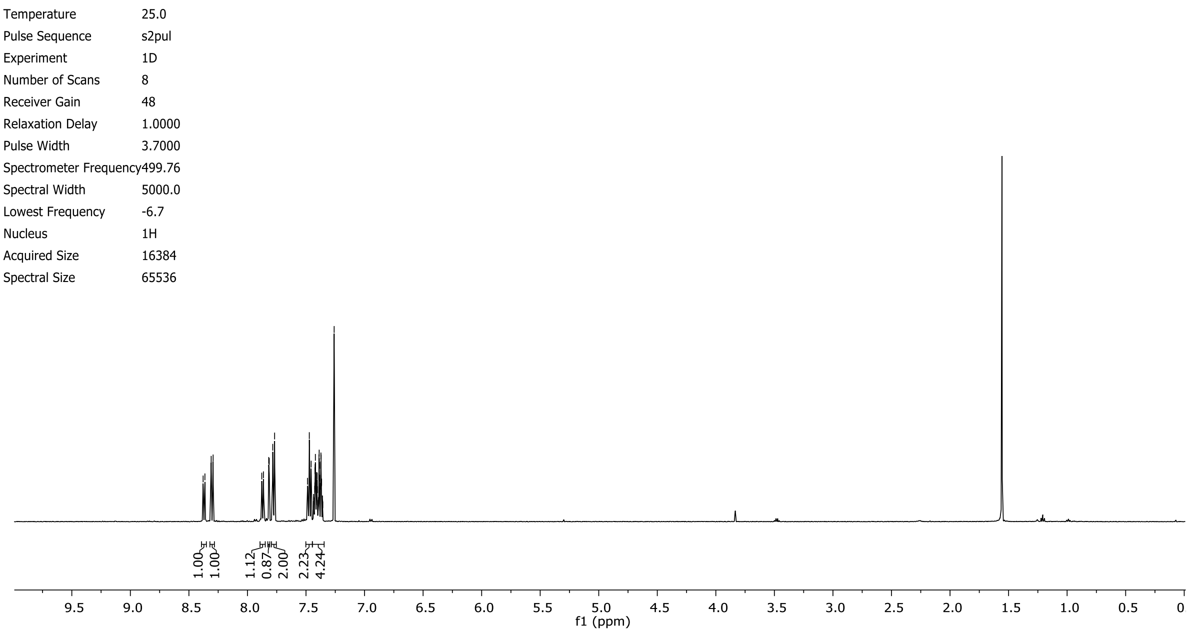

Parameter	Value
Title	CARBON_01
Comment	AN-17
Origin	Varian
Owner	nazarova
Instrument	vnmrs
Solvent	cdcl3
Temperature	25.0
Pulse Sequence	s2pul
Experiment	1D
Number of Scans	2880
Receiver Gain	30
Relaxation Delay	3.0000
Pulse Width	4.9500
Spectrometer Frequency	125.68
Spectral Width	31250.0
Lowest Frequency	-1781.8
Nucleus	13C
Acquired Size	32768
Spectral Size	65536

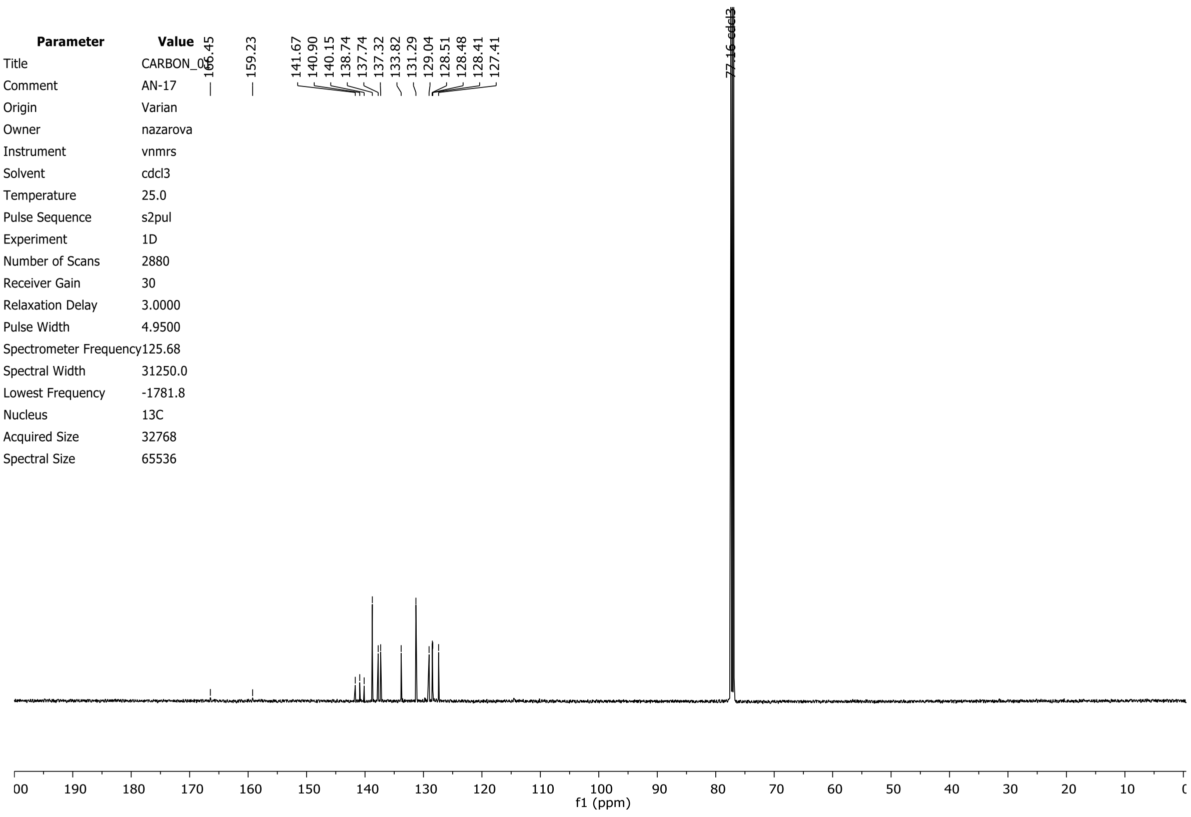

Parameter	Value
Title	PROTON_01
Comment	AN-14
Origin	Varian
Owner	nazarova
Instrument	vnmrs
Solvent	cdcl3
Temperature	25.0
Pulse Sequence	s2pul
Experiment	1D
Number of Scans	8
Receiver Gain	30
Relaxation Delay	5.0000
Pulse Width	3.7000
Spectrometer Frequency	499.76
Spectral Width	5000.0
Lowest Frequency	-6.2
Nucleus	1H
Acquired Size	16384
Spectral Size	65536

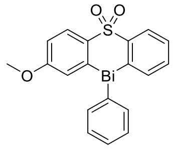

23

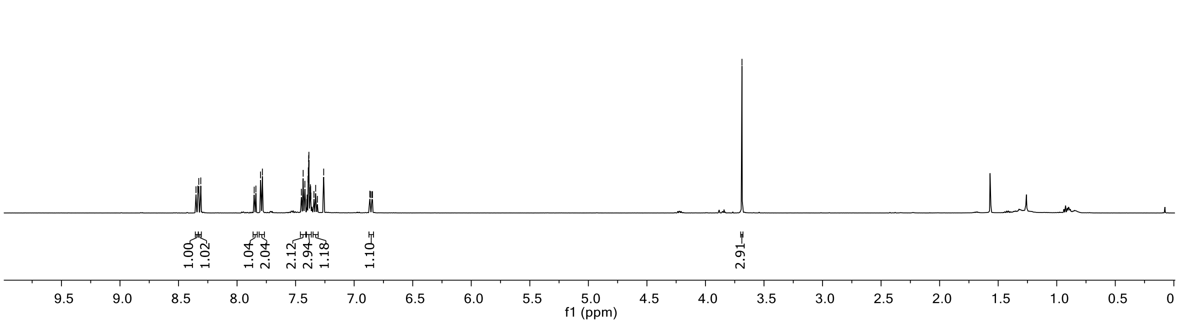

Parameter	Value
Title	CARBON_01
Comment	AN-14
Origin	Varian
Owner	nazarova
Instrument	vnmrs
Solvent	cdcl3
Temperature	25.0
Pulse Sequence	s2pul
Experiment	1D
Number of Scans	384
Receiver Gain	30
Relaxation Delay	2.0000
Pulse Width	4.9500
Spectrometer Frequency	125.68
Spectral Width	25510.2
Lowest Frequency	-251.6
Nucleus	13C
Acquired Size	32768
Spectral Size	65536

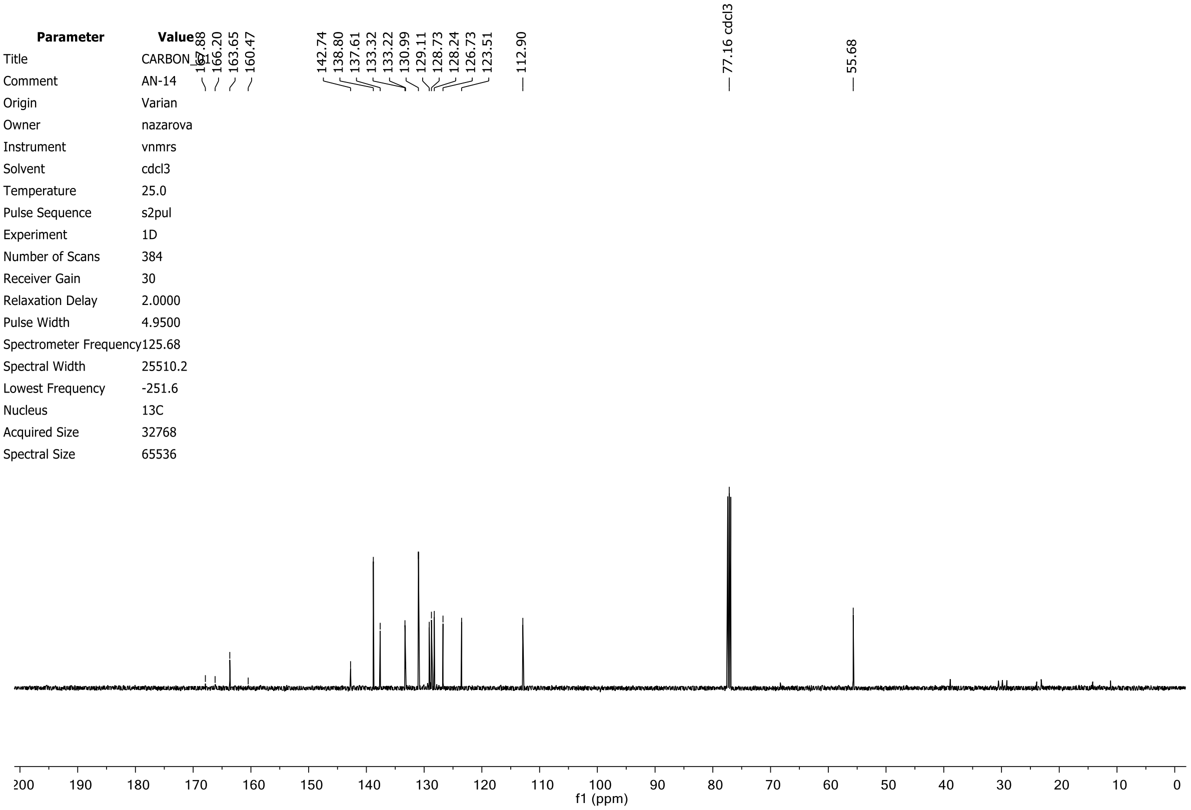

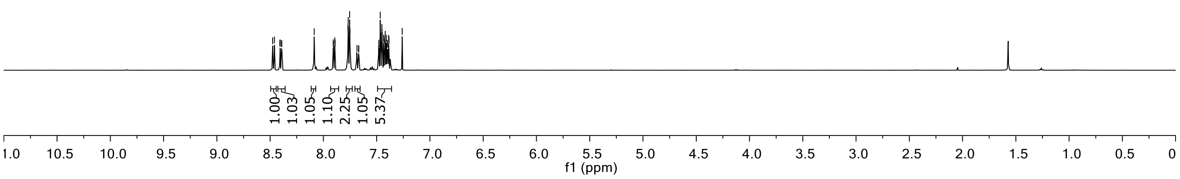

Parameter	Value
Title	PROTON_01
Comment	AN-18
Origin	Varian
Owner	nazarova
Instrument	vnmrs
Solvent	cdcl3
Temperature	25.0
Pulse Sequence	s2pul
Experiment	1D
Number of Scans	8
Receiver Gain	30
Relaxation Delay	1.0000
Pulse Width	3.7000
Spectrometer Frequency	499.76
Spectral Width	6009.6
Lowest Frequency	-511.5
Nucleus	1H
Acquired Size	16384
Spectral Size	65536

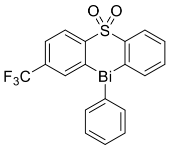

26

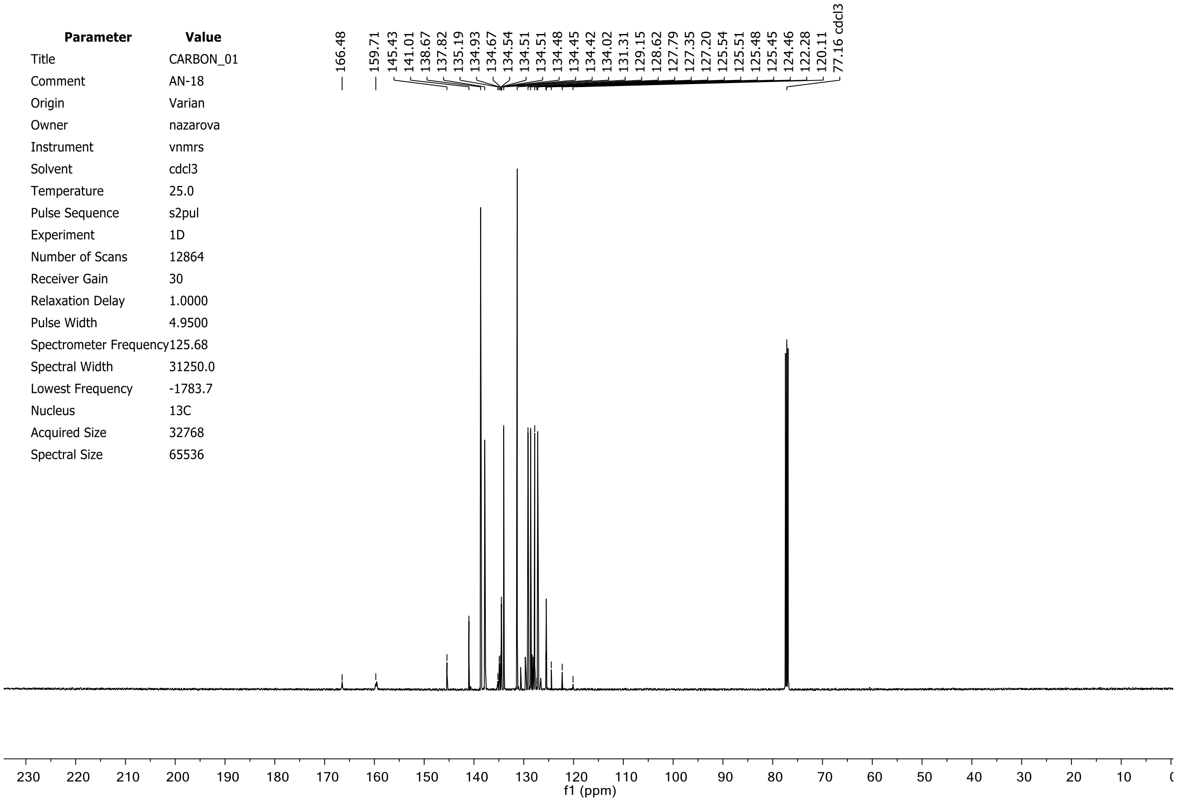

Parameter	Value
Title	CARBON_01
Comment	AN-18
Origin	Varian
Owner	nazarova
Instrument	vnmrs
Solvent	cdcl3
Temperature	25.0
Pulse Sequence	s2pul
Experiment	1D
Number of Scans	12864
Receiver Gain	30
Relaxation Delay	1.0000
Pulse Width	4.9500
Spectrometer Frequency	125.68
Spectral Width	31250.0
Lowest Frequency	-1783.7
Nucleus	13C
Acquired Size	32768
Spectral Size	65536

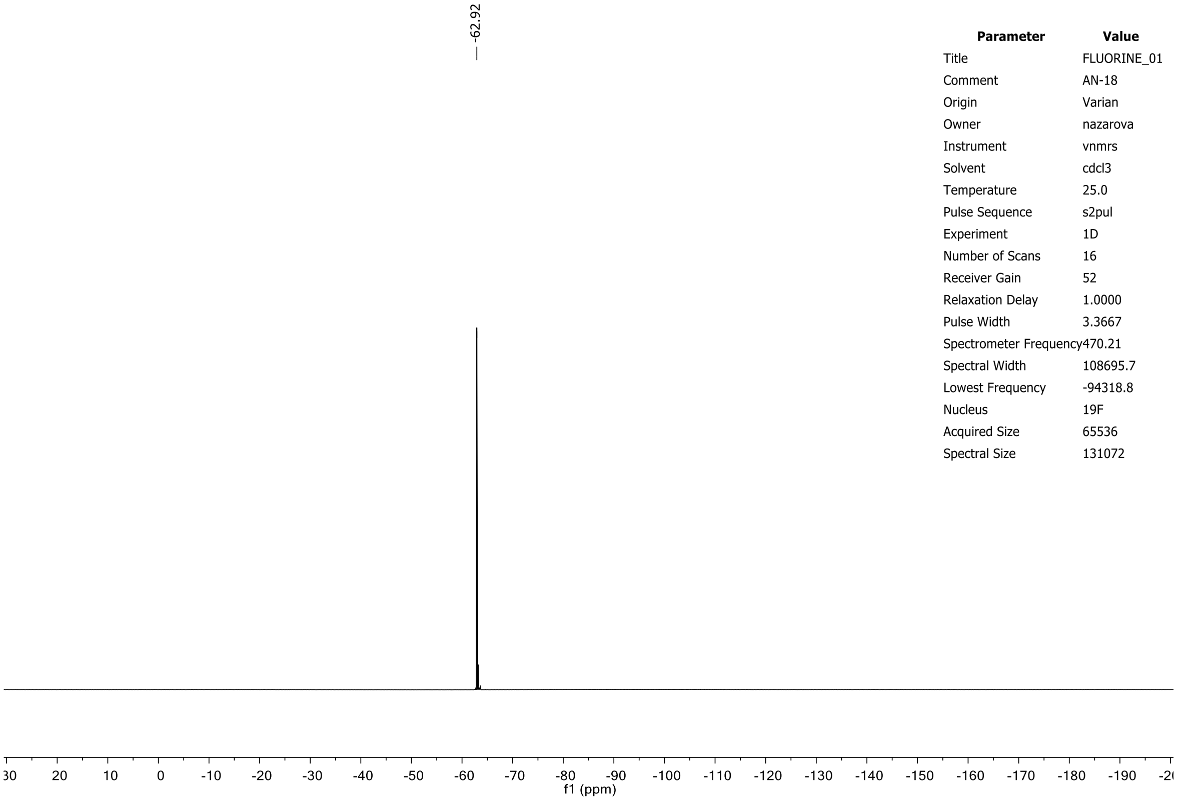
-62.92
Parameter Value
Title FLUORINE_01
Comment AN-18
Origin Varian
Owner nazarova
Instrument vnmrs
Solvent cdcl3
Temperature 25.0
Pulse Sequence s2pul
Experiment 1D
Number of Scans 16
Receiver Gain 52
Relaxation Delay 1.0000
Pulse Width 3.3667
Spectrometer Frequency 470.21
Spectral Width 108695.7
Lowest Frequency -94318.8
Nucleus 19F
Acquired Size 65536
Spectral Size 131072
30 20 10 0 -10 -20 -30 -40 -50 -60 -70 -80 -90 -100 -110 -120 -130 -140 -150 -160 -170 -180 -190
f1 (ppm)

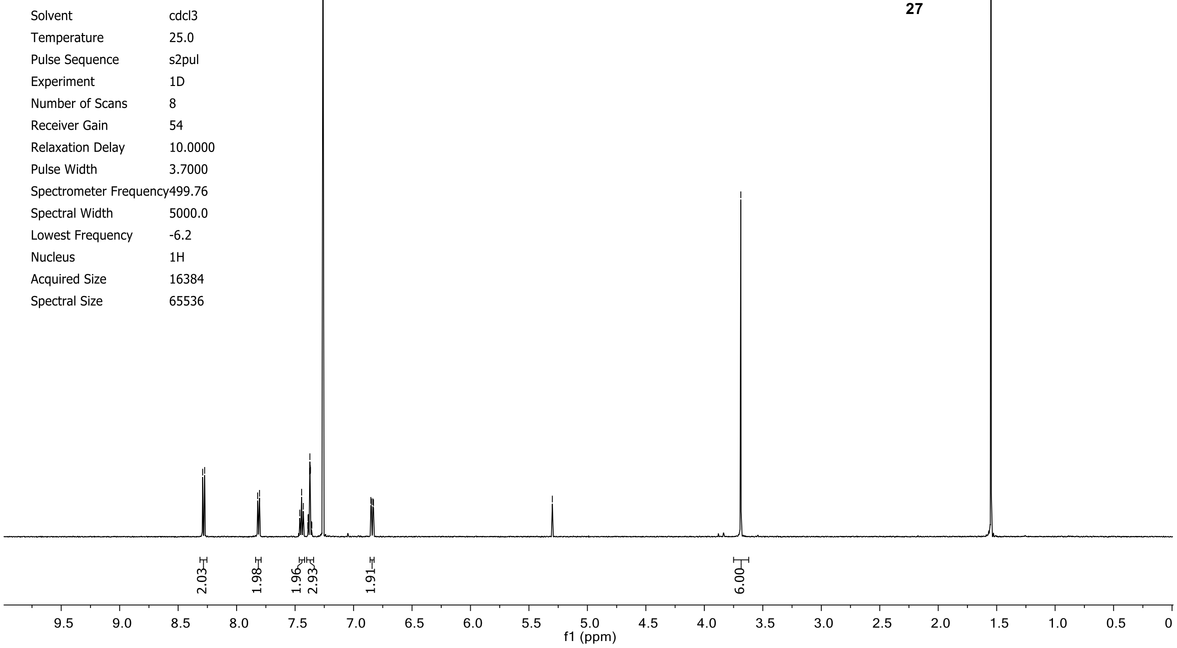

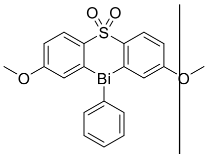

27

Parameter	Value
Title	PROTON_01
Comment	AN-19
Origin	Varian
Owner	nazarova
Instrument	vnmrs
Solvent	cdcl3
Temperature	25.0
Pulse Sequence	s2pul
Experiment	1D
Number of Scans	8
Receiver Gain	54
Relaxation Delay	10.0000
Pulse Width	3.7000
Spectrometer Frequency	499.76
Spectral Width	5000.0
Lowest Frequency	-6.2
Nucleus	1H
Acquired Size	16384
Spectral Size	65536

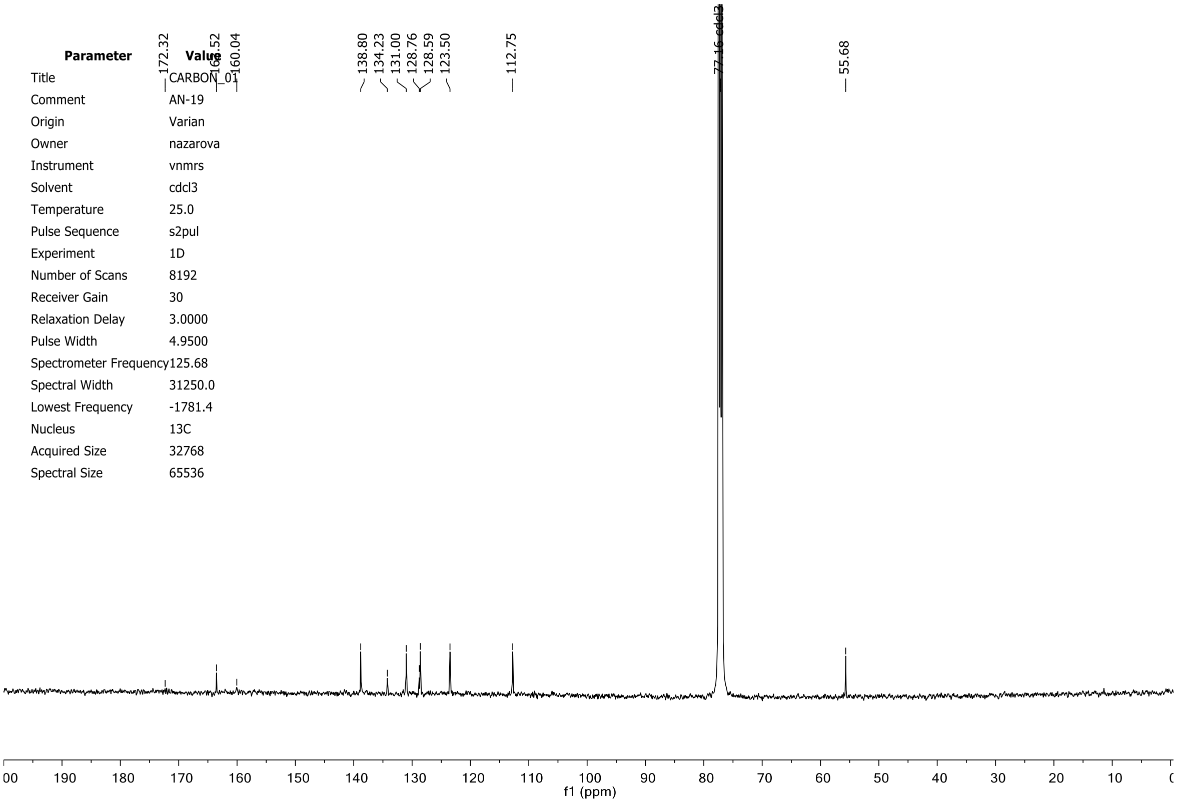

Parameter	Value
Title	CARBON_01
Comment	AN-19
Origin	Varian
Owner	nazarova
Instrument	vnmrs
Solvent	cdcl3
Temperature	25.0
Pulse Sequence	s2pul
Experiment	1D
Number of Scans	8192
Receiver Gain	30
Relaxation Delay	3.0000
Pulse Width	4.9500
Spectrometer Frequency	125.68
Spectral Width	31250.0
Lowest Frequency	-1781.4
Nucleus	13C
Acquired Size	32768
Spectral Size	65536

Parameter	Value
Title	PROTON_01
Comment	AN-21
Origin	Varian
Owner	nazarova
Instrument	mercury
Solvent	cdcl3
Temperature	25.0
Pulse Sequence	s2pul
Experiment	1D
Number of Scans	8
Receiver Gain	6
Relaxation Delay	5.0000
Pulse Width	3.7500
Spectrometer Frequency	400.12
Spectral Width	4001.6
Lowest Frequency	-15.4
Nucleus	1H
Acquired Size	16384
Spectral Size	65536

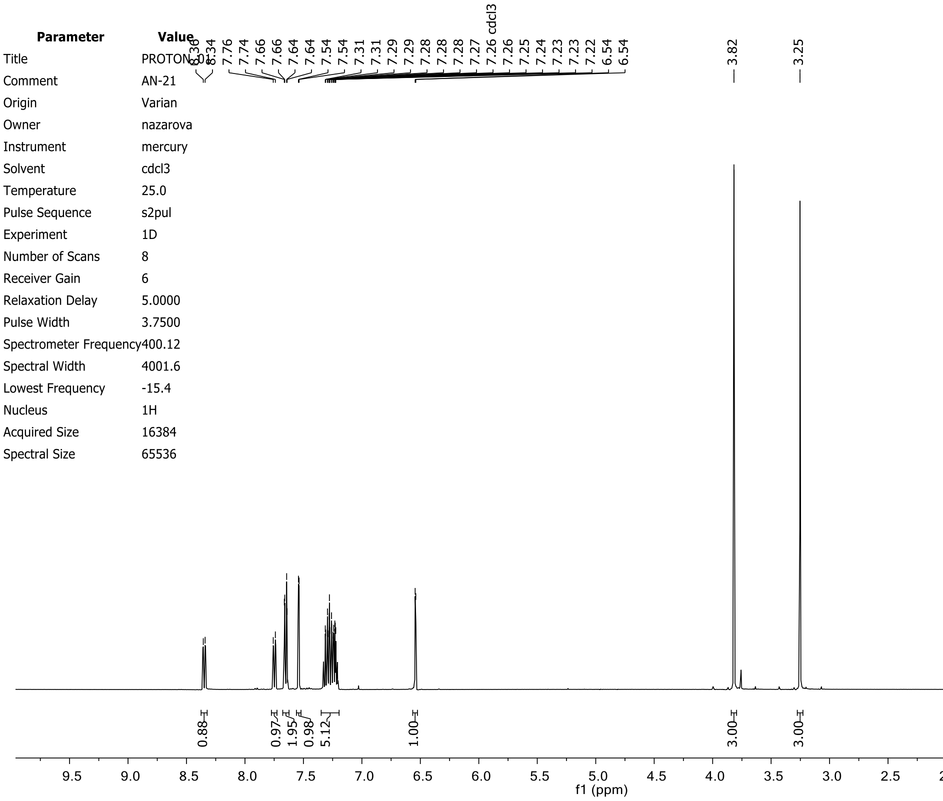

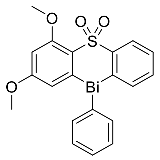

28

Parameter	Value
Title	CARBON_01
Comment	AN-21
Origin	Varian
Owner	nazarova
Instrument	mercury
Solvent	cdcl3
Temperature	25.0
Pulse Sequence	s2pul
Experiment	1D
Number of Scans	512
Receiver Gain	36
Relaxation Delay	1.0000
Pulse Width	16.2500
Spectrometer Frequency	100.62
Spectral Width	25125.6
Lowest Frequency	-1491.5
Nucleus	13C
Acquired Size	32768
Spectral Size	65536

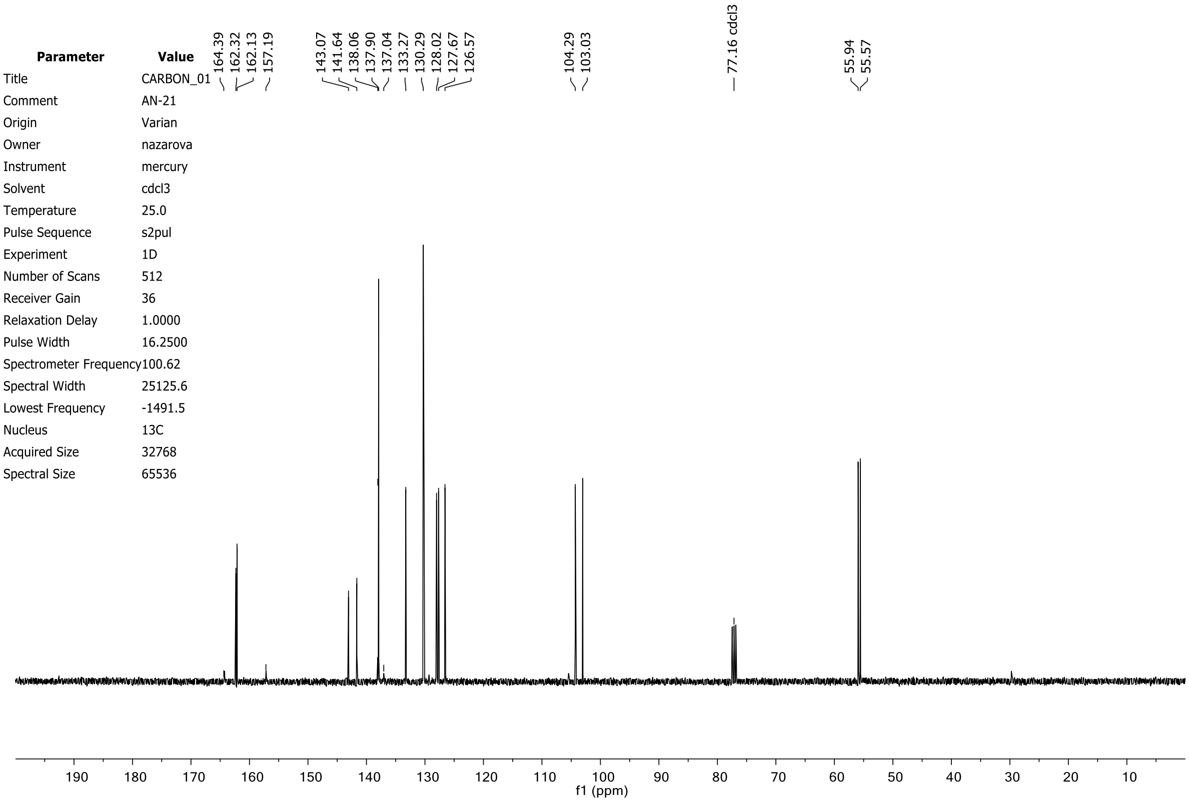

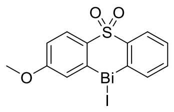

29

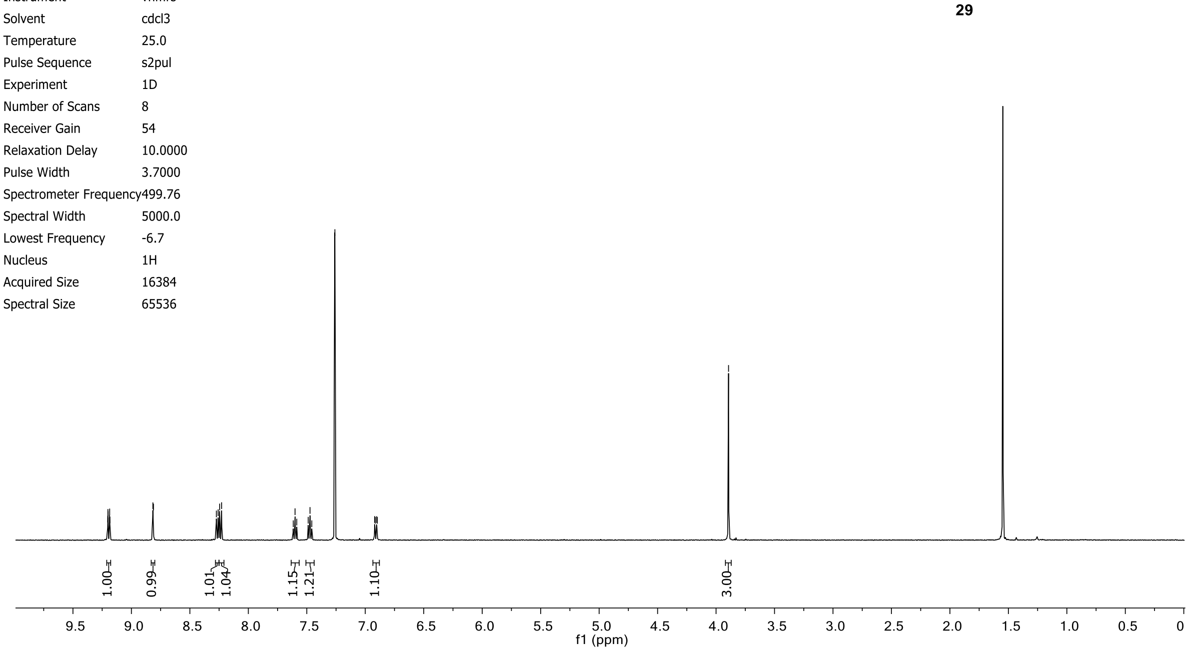
Parameter Value
Title PROTON_02
Comment AN-22
Origin Varian
Owner nazarova
Instrument vnmrs
Solvent cdcl3
Temperature 25.0
Pulse Sequence s2pul
Experiment 1D
Number of Scans 8
Receiver Gain 54
Relaxation Delay 10.0000
Pulse Width 3.7000
Spectrometer Frequency 499.76
Spectral Width 5000.0
Lowest Frequency -6.7
Nucleus 1H
Acquired Size 16384
Spectral Size 65536
9.20
9.20
9.20
9.19
9.19
9.18
8.82
8.81
8.27
8.26
8.25
8.23
7.62
7.60
7.59
7.49
7.47
7.46
7.26 cdcl3
6.92
6.91
6.90
6.90
3.89
1.00
0.99
1.01
1.04
1.15
1.21
1.10
3.00
9.5 9.0 8.5 8.0 7.5 7.0 6.5 6.0 5.5 5.0 4.5 4.0 3.5 3.0 2.5 2.0 1.5 1.0 0.5 0
f1 (ppm)

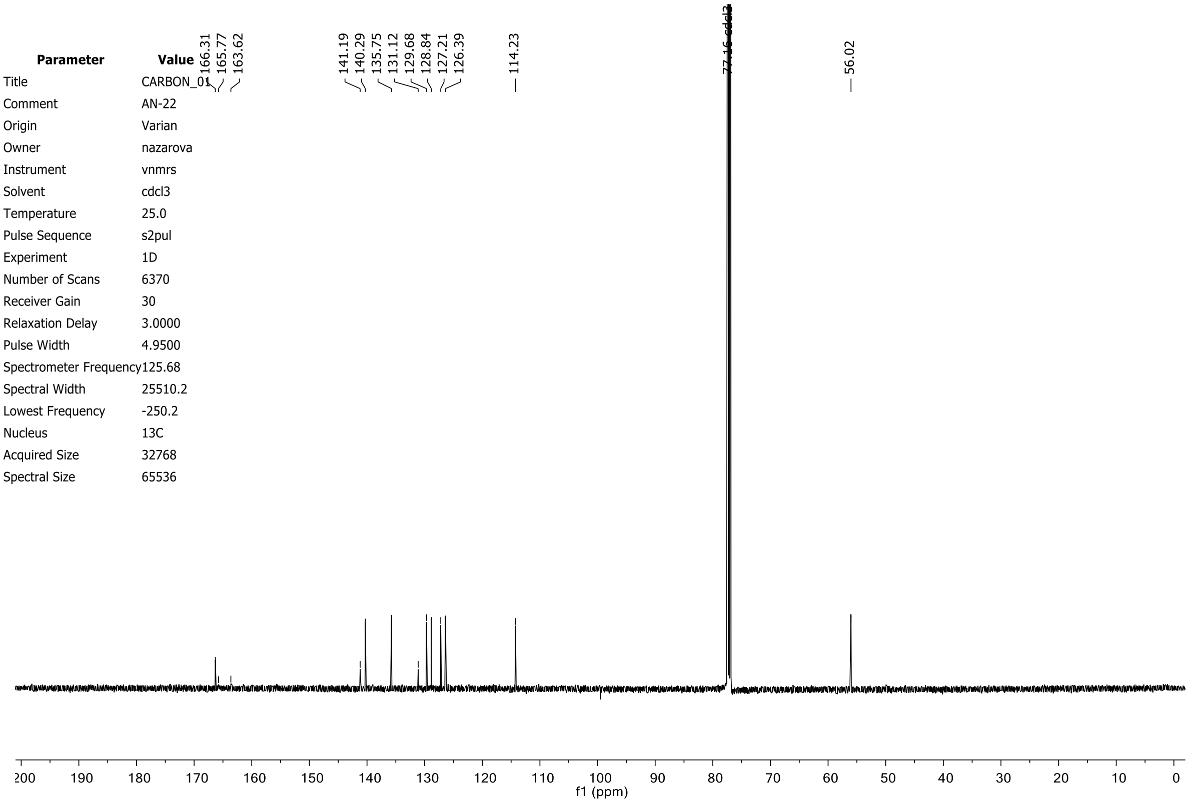
Parameter Value
Title CARBON_01
Comment AN-22
Origin Varian
Owner nazarova
Instrument vnmrs
Solvent cdcl3
Temperature 25.0
Pulse Sequence s2pul
Experiment 1D
Number of Scans 6370
Receiver Gain 30
Relaxation Delay 3.0000
Pulse Width 4.9500
Spectrometer Frequency 125.68
Spectral Width 25510.2
Lowest Frequency -250.2
Nucleus 13C
Acquired Size 32768
Spectral Size 65536
166.31
165.77
163.62
141.19
140.29
135.75
131.12
129.68
128.84
127.21
126.39
114.23
77.16 cdcl3
56.02
200 190 180 170 160 150 140 130 120 110 100 90 80 70 60 50 40 30 20 10 0
f1 (ppm)

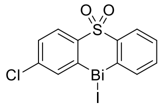

31

Parameter	Value
Title	PROTON_02
Comment	AN-25
Origin	Varian
Owner	nazarova
Instrument	vnmrs
Solvent	cdcl3
Temperature	25.0
Pulse Sequence	s2pul
Experiment	1D
Number of Scans	8
Receiver Gain	52
Relaxation Delay	5.0000
Pulse Width	3.7000
Spectrometer Frequency	499.76
Spectral Width	5000.0
Lowest Frequency	-6.2
Nucleus	1H
Acquired Size	16384
Spectral Size	65536

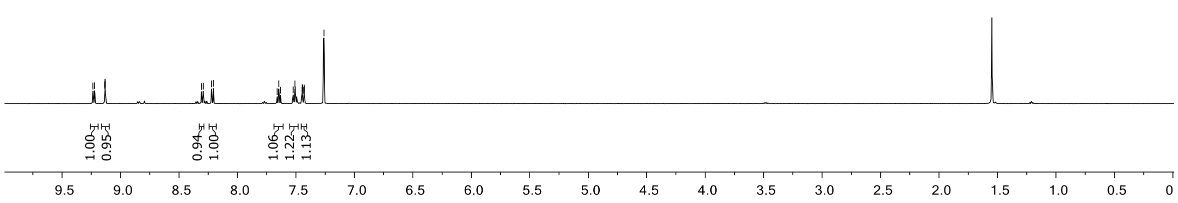

Parameter	Value
Title	CARBON_01
Comment	AN-25
Origin	Varian
Owner	nazarova
Instrument	vnmrs
Solvent	cdcl3
Temperature	25.0
Pulse Sequence	s2pul
Experiment	1D
Number of Scans	8896
Receiver Gain	30
Relaxation Delay	3.0000
Pulse Width	4.9500
Spectrometer Frequency	125.68
Spectral Width	26595.7
Lowest Frequency	-1339.6
Nucleus	13C
Acquired Size	32768
Spectral Size	65536

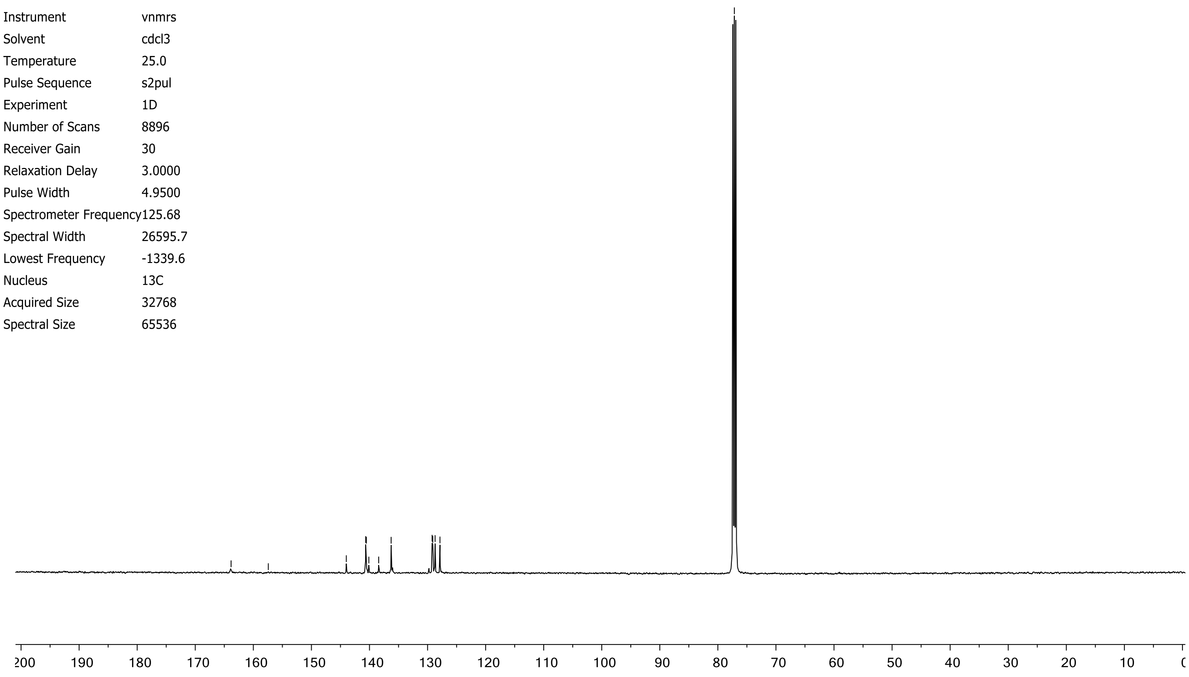

Parameter	Value
Title	PROTON_02
Comment	AN-26
Origin	Varian
Owner	nazarova
Instrument	vnmrs
Solvent	cdcl3
Temperature	25.0
Pulse Sequence	s2pul
Experiment	1D
Number of Scans	8
Receiver Gain	46
Relaxation Delay	2.0000
Pulse Width	3.7000
Spectrometer Frequency	499.76
Spectral Width	6009.6
Lowest Frequency	-511.0
Nucleus	1H
Acquired Size	16384
Spectral Size	65536

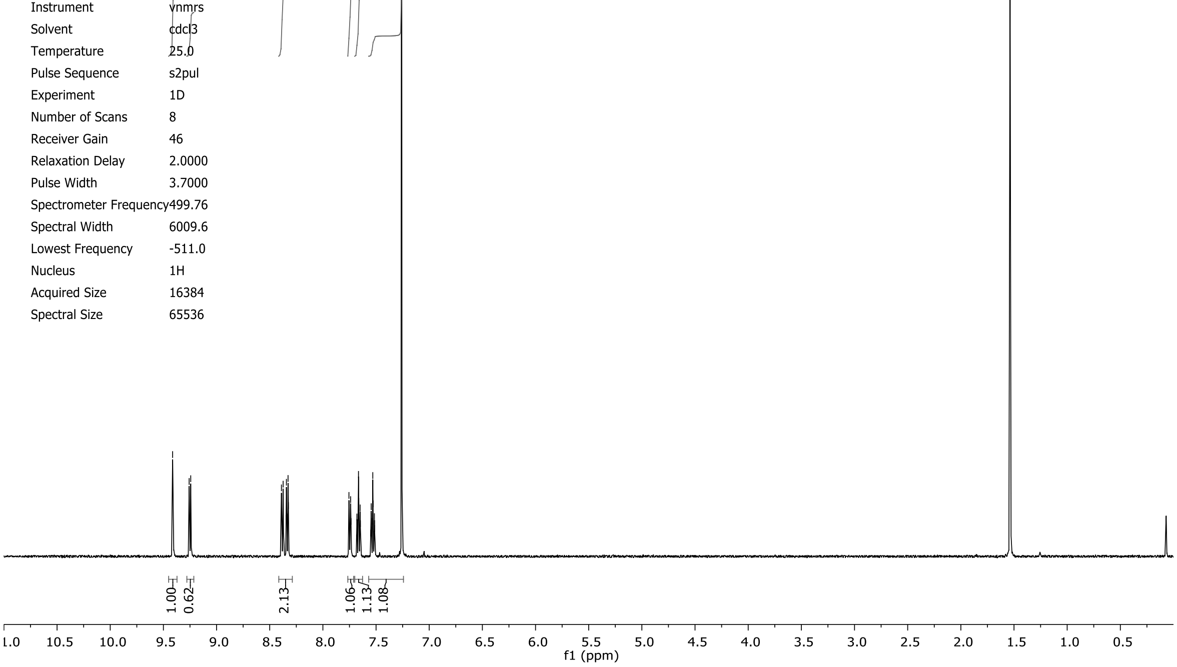

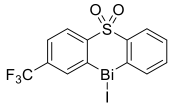

32

Parameter	Value
Title	CARBON_01
Comment	AN-26
Origin	Varian
Owner	nazarova
Instrument	vnmrs
Solvent	cdcl3
Temperature	25.0
Pulse Sequence	s2pul
Experiment	1D
Number of Scans	8064
Receiver Gain	30
Relaxation Delay	4.0000
Pulse Width	4.9500
Spectrometer Frequency	125.68
Spectral Width	31250.0
Lowest Frequency	-1801.8
Nucleus	13C
Acquired Size	32768
Spectral Size	65536

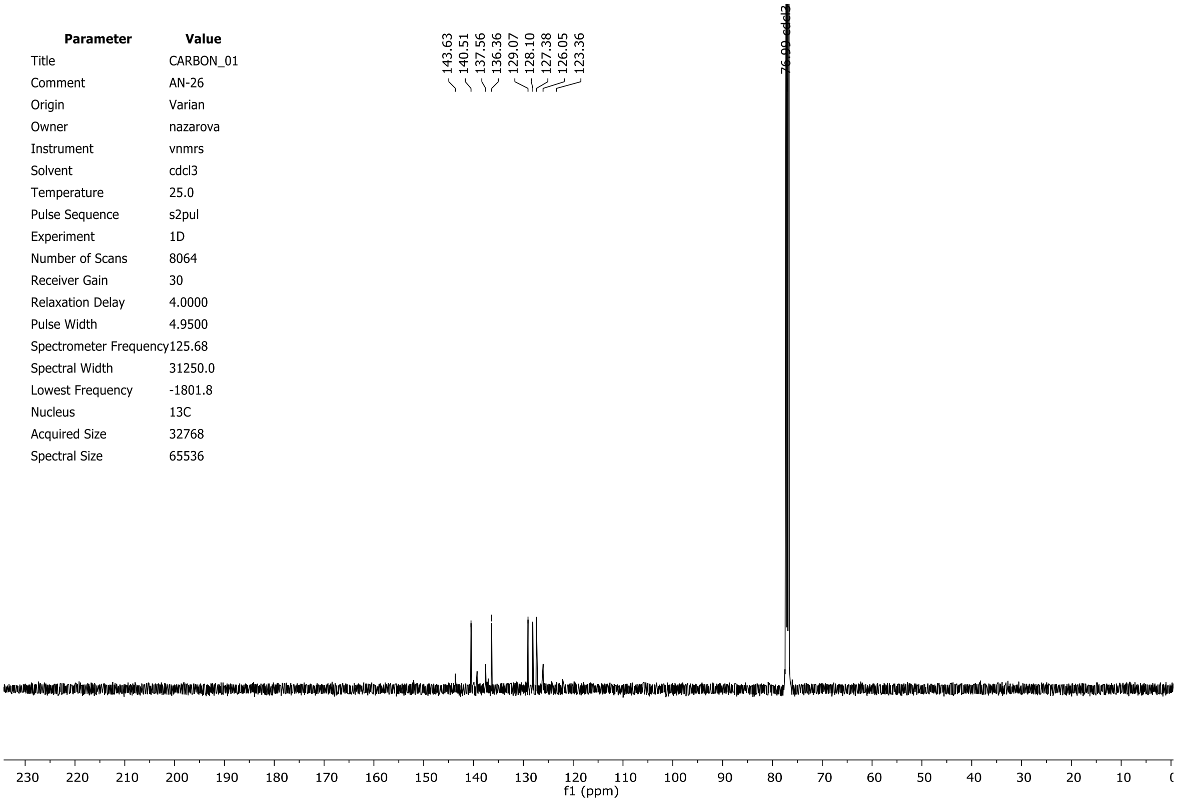

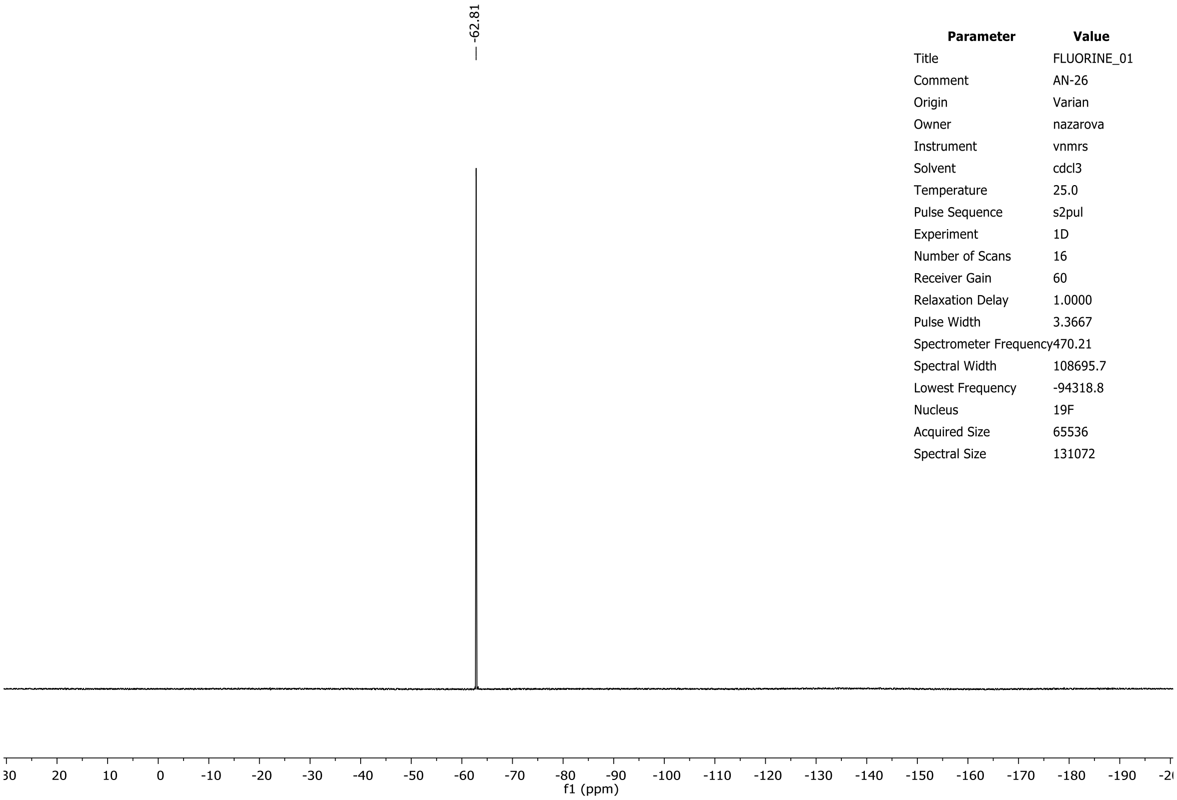

Parameter	Value
Title	FLUORINE_01
Comment	AN-26
Origin	Varian
Owner	nazarova
Instrument	vnmrs
Solvent	cdcl3
Temperature	25.0
Pulse Sequence	s2pul
Experiment	1D
Number of Scans	16
Receiver Gain	60
Relaxation Delay	1.0000
Pulse Width	3.3667
Spectrometer Frequency	470.21
Spectral Width	108695.7
Lowest Frequency	-94318.8
Nucleus	19F
Acquired Size	65536
Spectral Size	131072

Parameter	Value
Title	PROTON_02
Comment	AN-27
Origin	Varian
Owner	nazarova
Instrument	vnmrs
Solvent	cdcl3
Temperature	25.0
Pulse Sequence	s2pul
Experiment	1D
Number of Scans	8
Receiver Gain	52
Relaxation Delay	1.0000
Pulse Width	3.7000
Spectrometer Frequency	499.76
Spectral Width	6009.6
Lowest Frequency	-511.7
Nucleus	1H
Acquired Size	16384
Spectral Size	65536

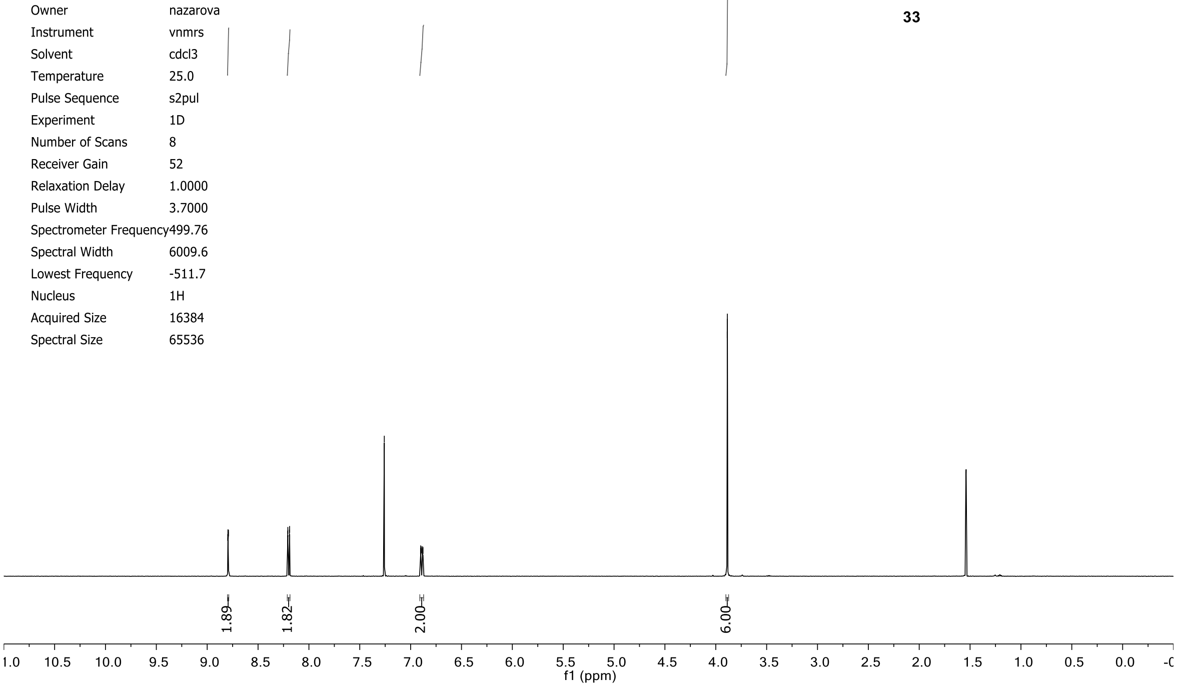

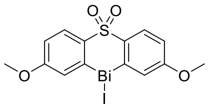

33

Parameter	Value
Title	CARBON_01
Comment	AN-27
Origin	Varian
Owner	nazarova
Instrument	vnmrs
Solvent	cdcl3
Temperature	25.0
Pulse Sequence	s2pul
Experiment	1D
Number of Scans	9168
Receiver Gain	30
Relaxation Delay	2.0000
Pulse Width	4.9500
Spectrometer Frequency	125.68
Spectral Width	31250.0
Lowest Frequency	-1781.7
Nucleus	13C
Acquired Size	32768
Spectral Size	65536

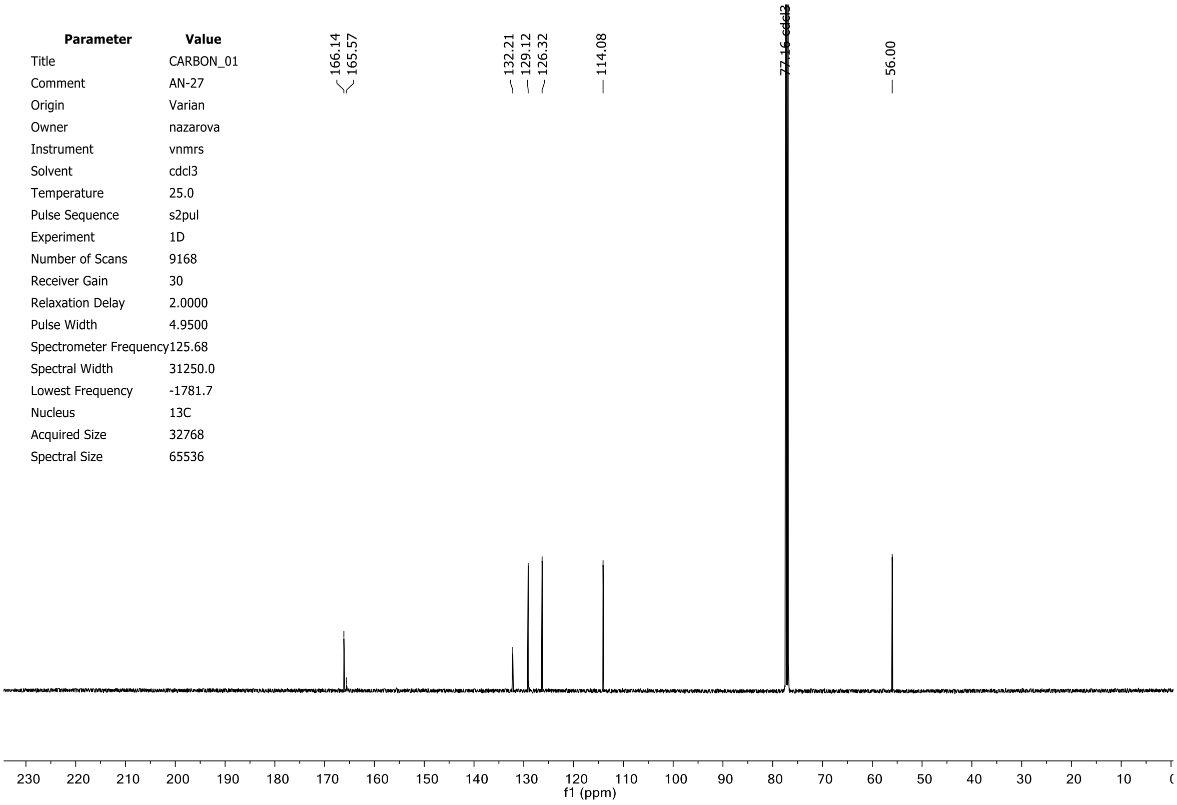

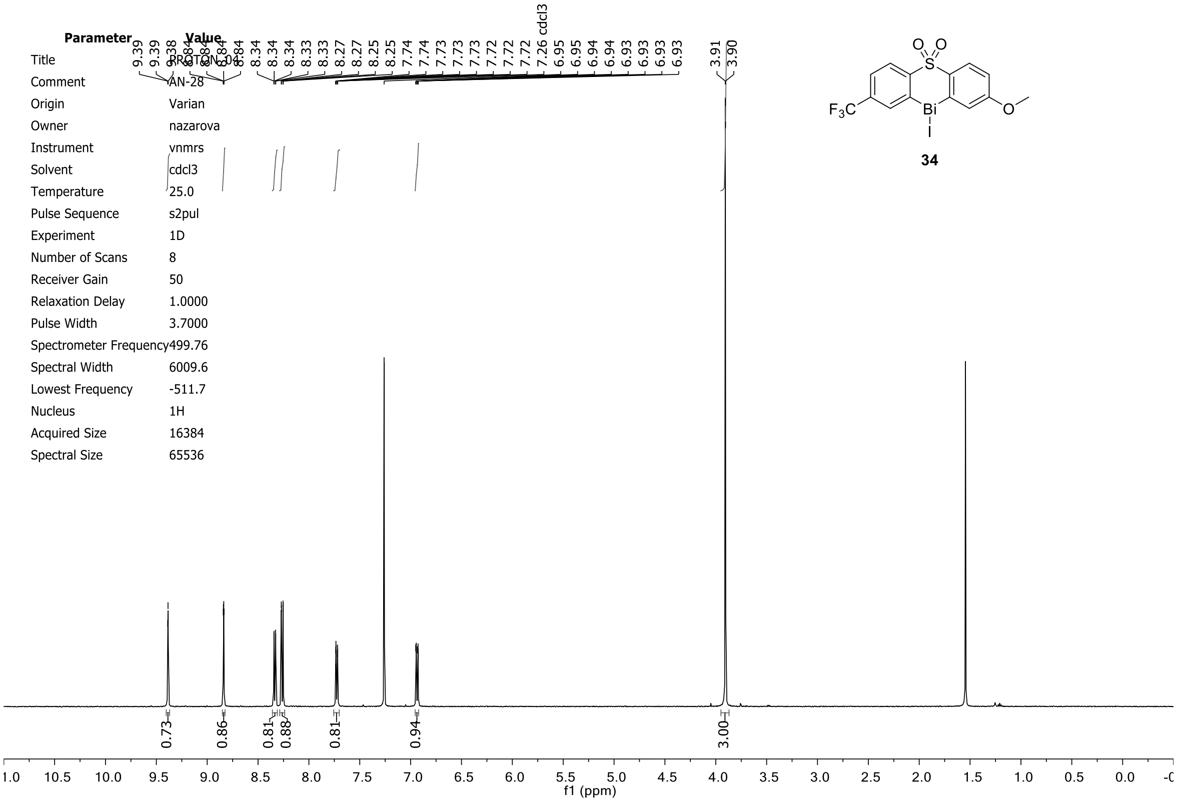

34

Parameter	Value
Title	PROTON_04
Comment	AN-28
Origin	Varian
Owner	nazarova
Instrument	vnmrs
Solvent	cdcl3
Temperature	25.0
Pulse Sequence	s2pul
Experiment	1D
Number of Scans	8
Receiver Gain	50
Relaxation Delay	1.0000
Pulse Width	3.7000
Spectrometer Frequency	499.76
Spectral Width	6009.6
Lowest Frequency	-511.7
Nucleus	1H
Acquired Size	16384
Spectral Size	65536

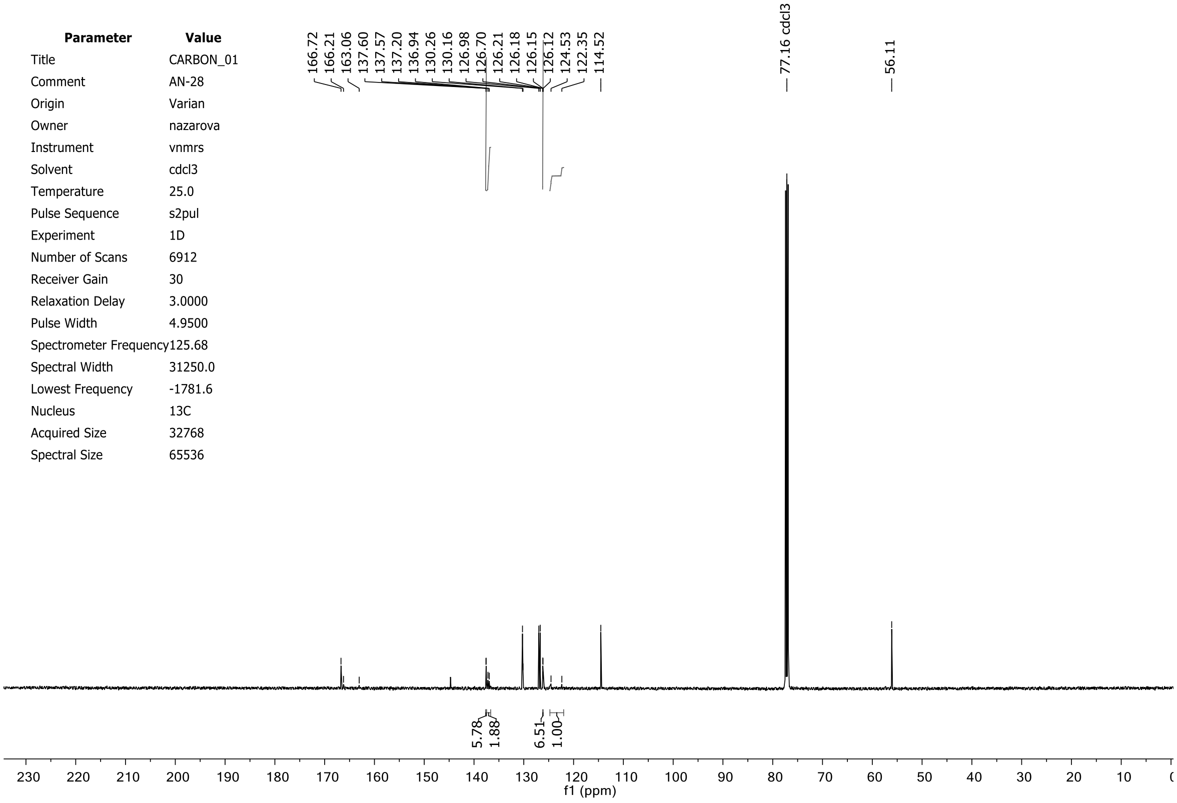

Parameter	Value
Title	CARBON_01
Comment	AN-28
Origin	Varian
Owner	nazarova
Instrument	vnmrs
Solvent	cdcl3
Temperature	25.0
Pulse Sequence	s2pul
Experiment	1D
Number of Scans	6912
Receiver Gain	30
Relaxation Delay	3.0000
Pulse Width	4.9500
Spectrometer Frequency	125.68
Spectral Width	31250.0
Lowest Frequency	-1781.6
Nucleus	13C
Acquired Size	32768
Spectral Size	65536

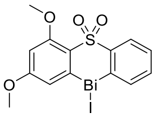

35

Parameter	Value
Title	PROTON_03
Comment	AN-30
Origin	Varian
Owner	nazarova
Instrument	vnmrs
Solvent	cdcl3
Temperature	25.0
Pulse Sequence	s2pul
Experiment	1D
Number of Scans	8
Receiver Gain	52
Relaxation Delay	5.0000
Pulse Width	3.7000
Spectrometer Frequency	499.76
Spectral Width	6009.6
Lowest Frequency	-511.0
Nucleus	1H
Acquired Size	16384
Spectral Size	65536

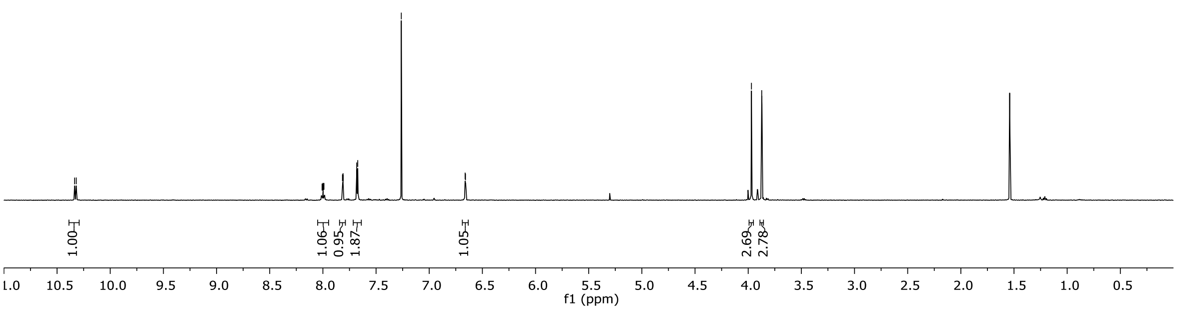

Parameter	Value
Title	CARBON_01
Comment	AN-30
Origin	Varian
Owner	nazarova
Instrument	vnmrs
Solvent	cdcl3
Temperature	25.0
Pulse Sequence	s2pul
Experiment	1D
Number of Scans	3000
Receiver Gain	30
Relaxation Delay	5.0000
Pulse Width	4.9500
Spectrometer Frequency	125.68
Spectral Width	31250.0
Lowest Frequency	-1781.1
Nucleus	13C
Acquired Size	32768
Spectral Size	65536

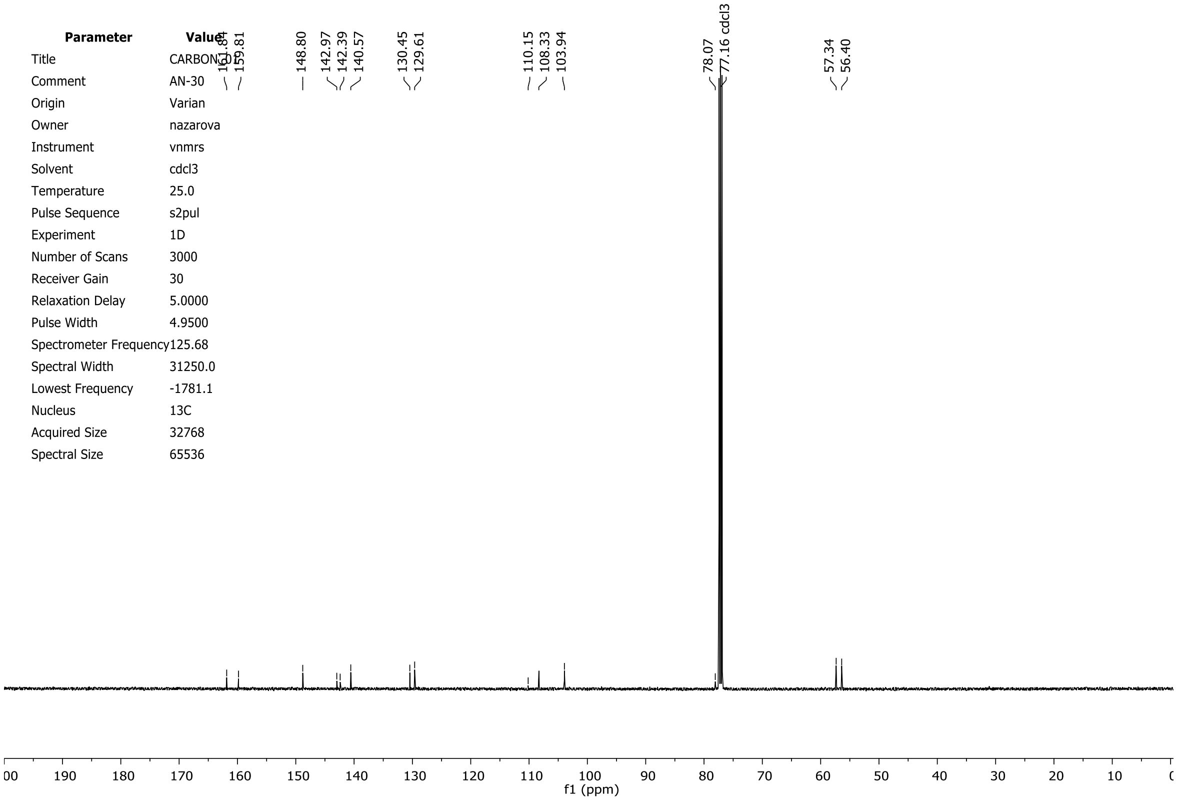

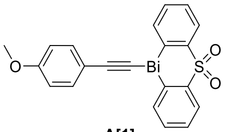

A[1]

Parameter	Value
Title	PROTON_01
Comment	AN-31
Origin	Varian
Owner	nazarova
Instrument	vnmrs
Solvent	cdcl3
Temperature	25.0
Pulse Sequence	s2pul
Experiment	1D
Number of Scans	8
Receiver Gain	50
Relaxation Delay	5.0000
Pulse Width	3.7000
Spectrometer Frequency	499.76
Spectral Width	5000.0
Lowest Frequency	-6.2
Nucleus	1H
Acquired Size	16384
Spectral Size	65536

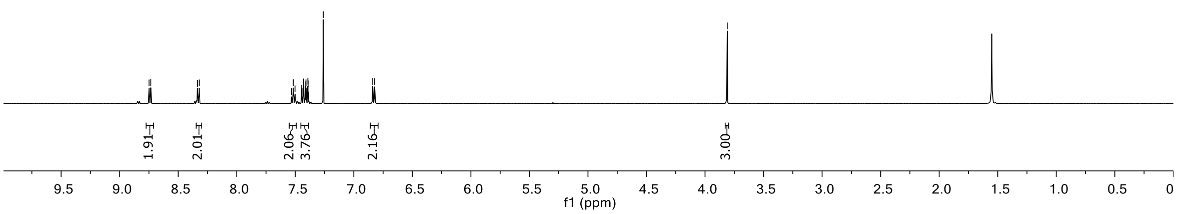

Parameter	Value
Title	CARBON_01
Comment	AN-31
Origin	Varian
Owner	nazarova
Instrument	vnmrs
Solvent	cdcl3
Temperature	25.0
Pulse Sequence	s2pul
Experiment	1D
Number of Scans	7872
Receiver Gain	30
Relaxation Delay	3.0000
Pulse Width	4.9500
Spectrometer Frequency	125.68
Spectral Width	31250.0
Lowest Frequency	-1781.8
Nucleus	13C
Acquired Size	32768
Spectral Size	65536

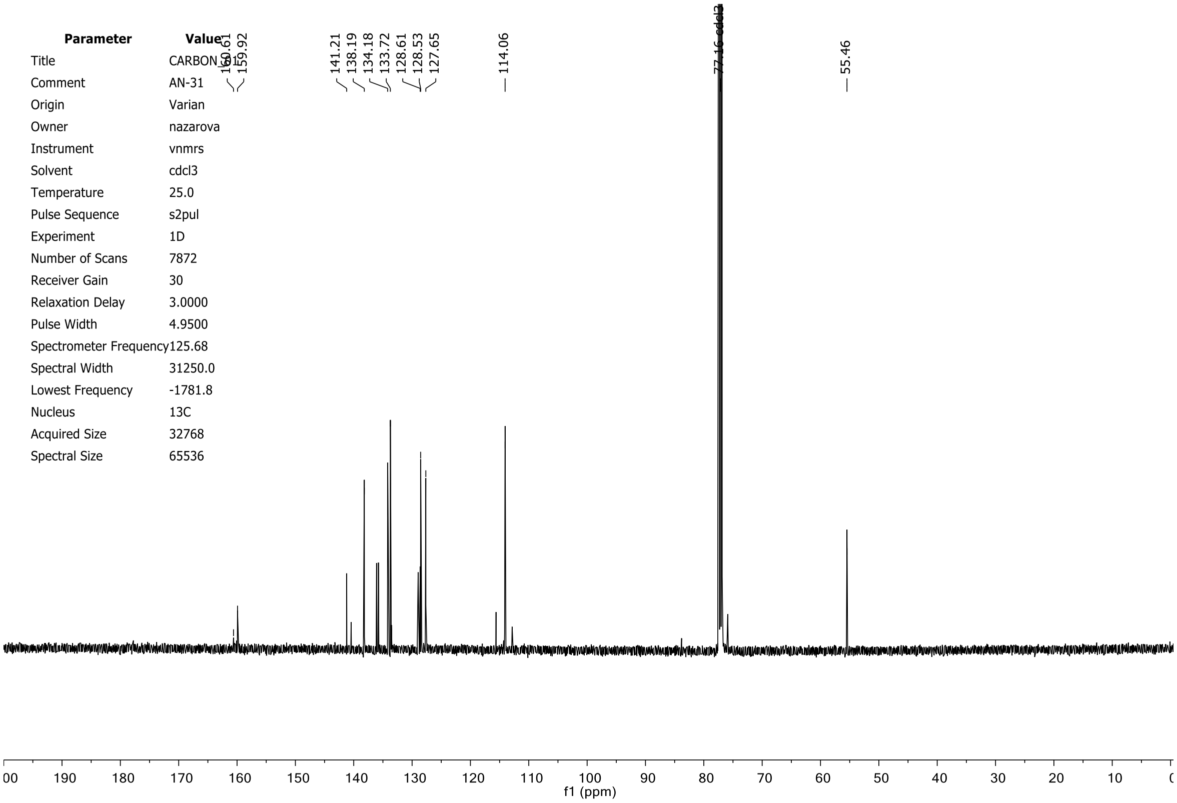

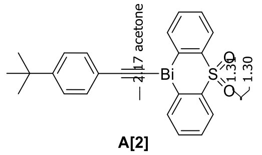

A[2]

Parameter	Value
Title	PROTON_01
Comment	AN-32
Origin	Varian
Owner	nazarova
Instrument	vnmrs
Solvent	cdcl3
Temperature	25.0
Pulse Sequence	s2pul
Experiment	1D
Number of Scans	8
Receiver Gain	48
Relaxation Delay	5.0000
Pulse Width	3.7000
Spectrometer Frequency	499.76
Spectral Width	5000.0
Lowest Frequency	-7.7
Nucleus	1H
Acquired Size	16384
Spectral Size	65536

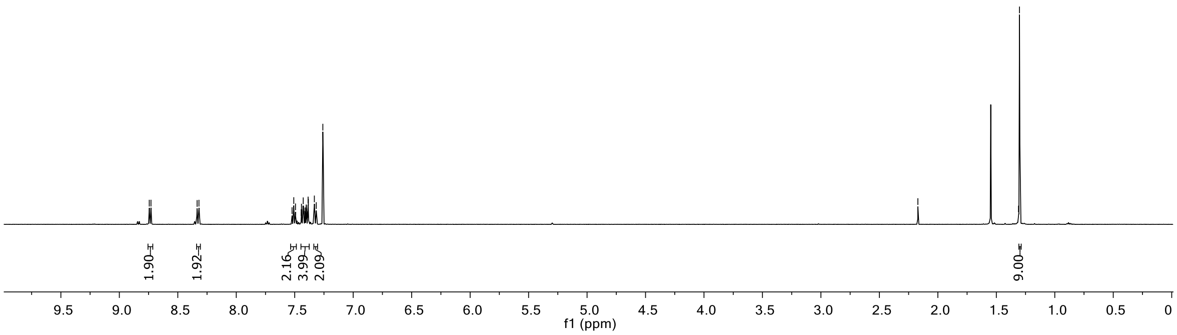

Parameter	Value
Title	CARBON_01
Comment	AN-32
Origin	Varian
Owner	nazarova
Instrument	vnmrs
Solvent	cdcl3
Temperature	25.0
Pulse Sequence	s2pul
Experiment	1D
Number of Scans	9728
Receiver Gain	30
Relaxation Delay	3.0000
Pulse Width	4.9500
Spectrometer Frequency	125.68
Spectral Width	31250.0
Lowest Frequency	-1782.0
Nucleus	13C
Acquired Size	32768
Spectral Size	65536

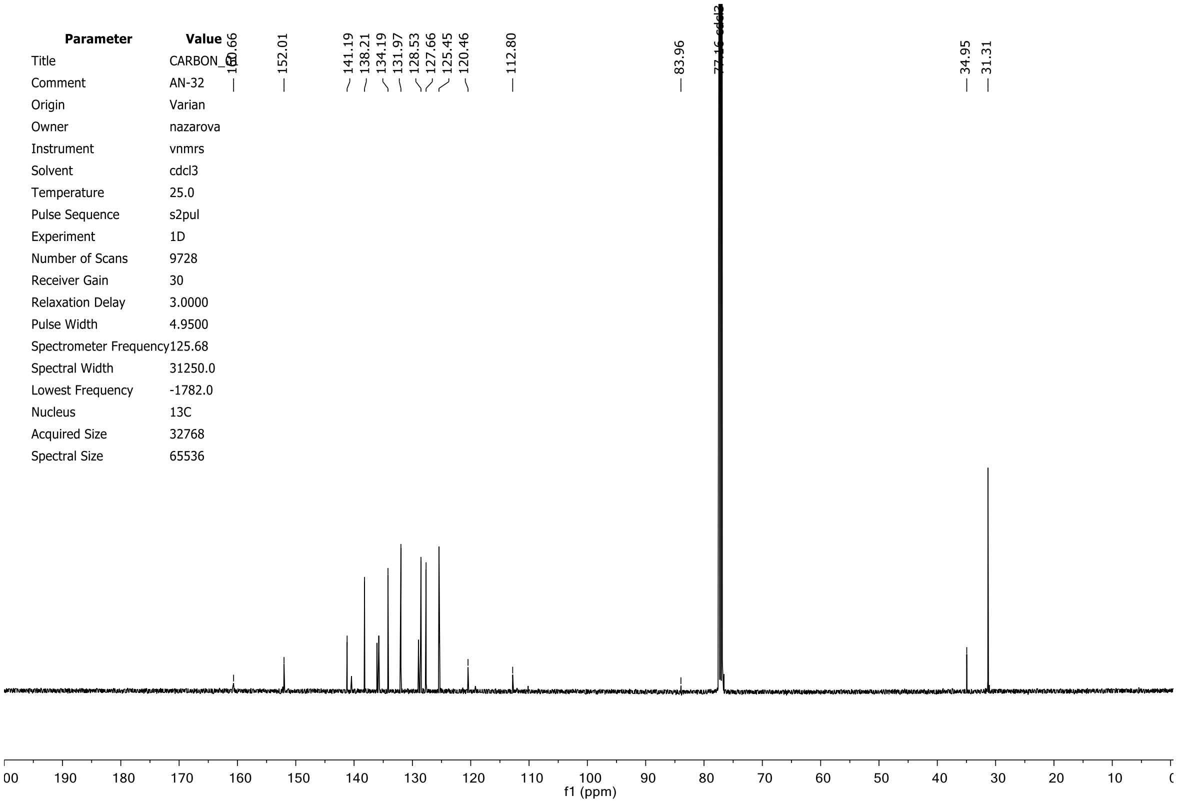

Parameter	Value
Title	PROTON_02
Comment	AN-33
Origin	Varian
Owner	nazarova
Instrument	vnmrs
Solvent	cdcl3
Temperature	25.0
Pulse Sequence	s2pul
Experiment	1D
Number of Scans	16
Receiver Gain	52
Relaxation Delay	10.0000
Pulse Width	3.7000
Spectrometer Frequency	499.76
Spectral Width	5000.0
Lowest Frequency	-6.2
Nucleus	1H
Acquired Size	16384
Spectral Size	65536

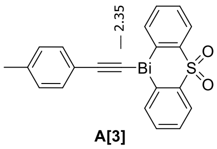

A[3]

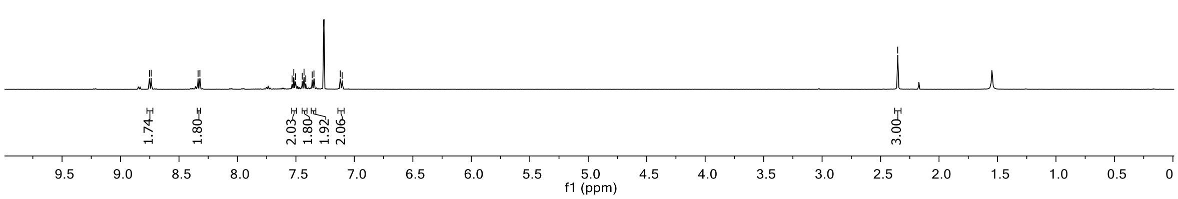

Parameter	Value
Title	CARBON_01
Comment	AN-33
Origin	Varian
Owner	nazarova
Instrument	vnmrs
Solvent	cdcl3
Temperature	25.0
Pulse Sequence	s2pul
Experiment	1D
Number of Scans	12864
Receiver Gain	30
Relaxation Delay	3.0000
Pulse Width	4.9500
Spectrometer Frequency	125.68
Spectral Width	31250.0
Lowest Frequency	-1781.8
Nucleus	13C
Acquired Size	32768
Spectral Size	65536

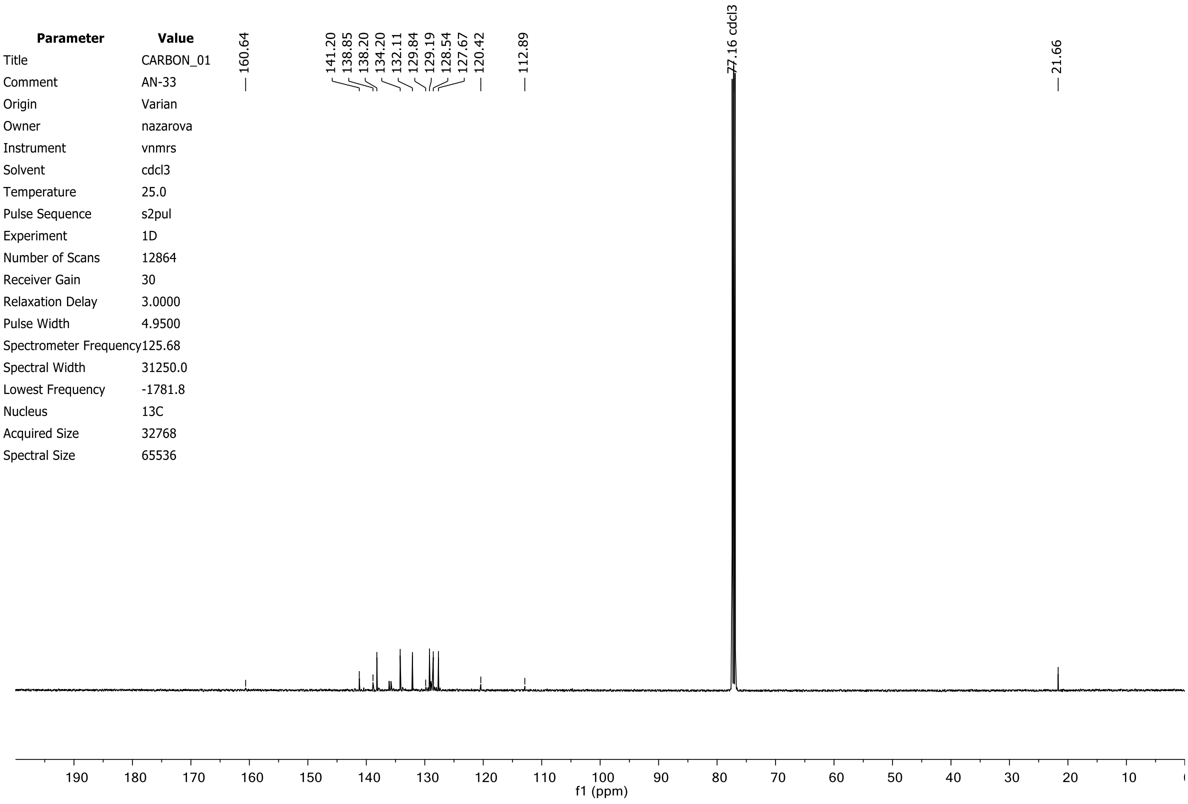

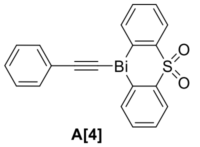

A[4]

Parameter	Value
Title	PROTON_01
Comment	AN-34
Origin	Varian
Owner	nazarova
Instrument	vnmrs
Solvent	cdcl3
Temperature	25.0
Pulse Sequence	s2pul
Experiment	1D
Number of Scans	8
Receiver Gain	50
Relaxation Delay	10.0000
Pulse Width	3.7000
Spectrometer Frequency	499.76
Spectral Width	5000.0
Lowest Frequency	-7.2
Nucleus	1H
Acquired Size	16384
Spectral Size	65536

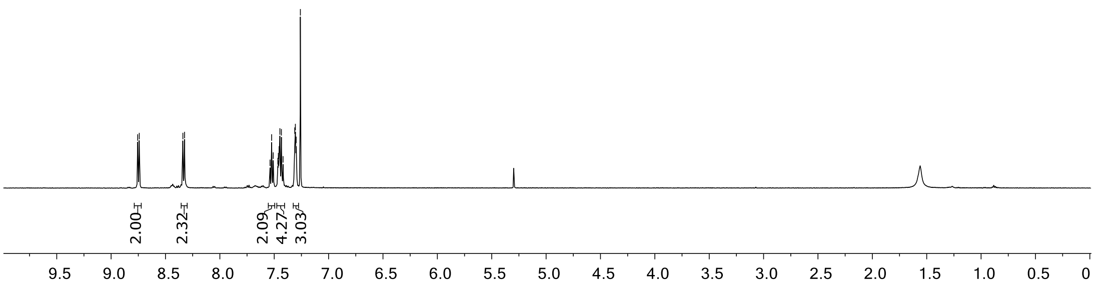

Parameter	Value
Title	CARBON_01
Comment	AN-34
Origin	Varian
Owner	nazarova
Instrument	vnmrs
Solvent	cdcl3
Temperature	25.0
Pulse Sequence	s2pul
Experiment	1D
Number of Scans	1344
Receiver Gain	30
Relaxation Delay	3.0000
Pulse Width	4.9500
Spectrometer Frequency	125.68
Spectral Width	25510.2
Lowest Frequency	-250.5
Nucleus	13C
Acquired Size	32768
Spectral Size	65536

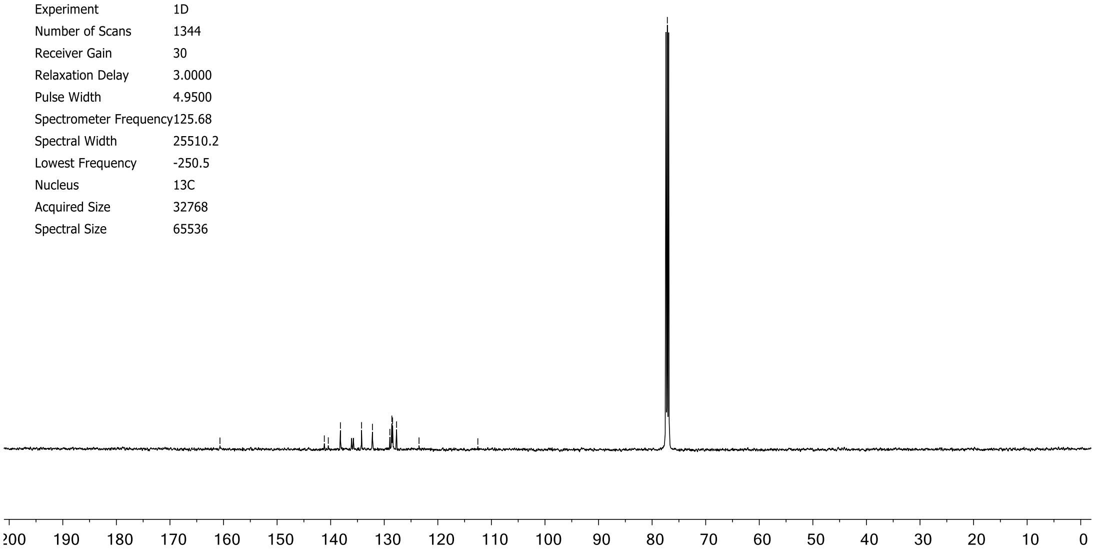

Parameter	Value
Title	PROTON_02
Comment	AN-35
Origin	Varian
Owner	nazarova
Instrument	vnmrs
Solvent	cdcl3
Temperature	25.0
Pulse Sequence	s2pul
Experiment	1D
Number of Scans	8
Receiver Gain	54
Relaxation Delay	10.0000
Pulse Width	3.7000
Spectrometer Frequency	499.77
Spectral Width	6983.2
Lowest Frequency	1.8
Nucleus	1H
Acquired Size	16384
Spectral Size	65536

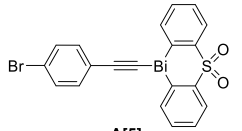

A[5]

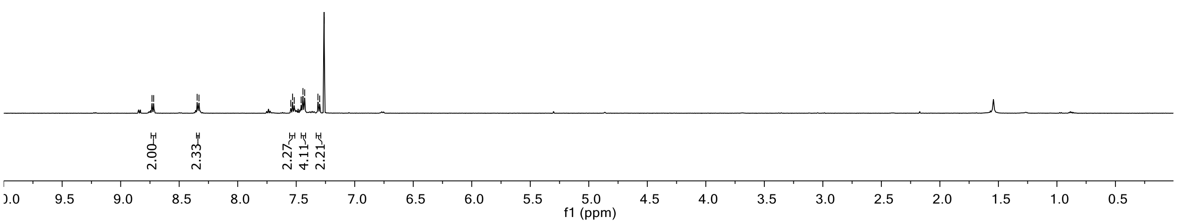

Parameter	Value
Title	CARBON_01
Comment	AN-35
Origin	Varian
Owner	nazarova
Instrument	vnmrs
Solvent	cdcl3
Temperature	25.0
Pulse Sequence	s2pul
Experiment	1D
Number of Scans	6656
Receiver Gain	30
Relaxation Delay	3.0000
Pulse Width	4.9500
Spectrometer Frequency	125.68
Spectral Width	31250.0
Lowest Frequency	-1781.6
Nucleus	13C
Acquired Size	32768
Spectral Size	65536

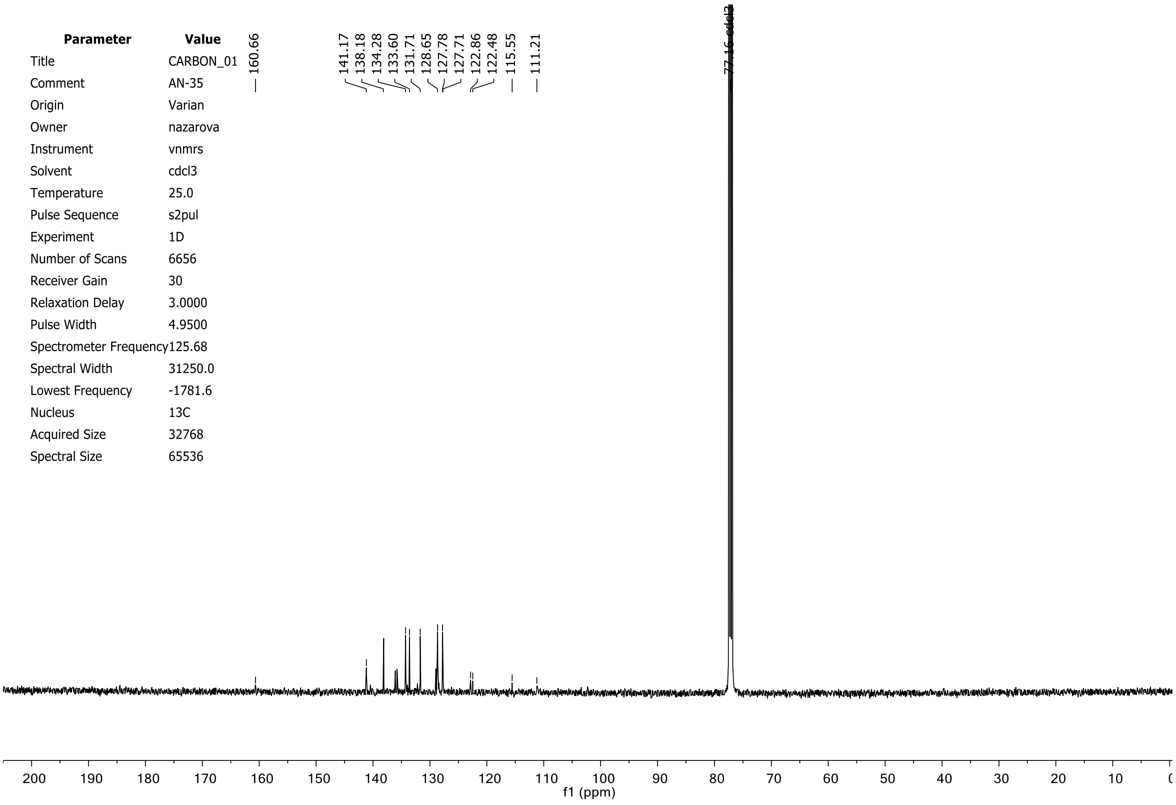

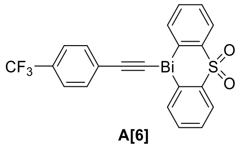

A[6]

Parameter	Value
Title	PROTON_02
Comment	AN-36
Origin	Varian
Owner	nazarova
Instrument	vnmrs
Solvent	cdcl3
Temperature	25.0
Pulse Sequence	s2pul
Experiment	1D
Number of Scans	8
Receiver Gain	30
Relaxation Delay	5.0000
Pulse Width	3.7000
Spectrometer Frequency	499.76
Spectral Width	5000.0
Lowest Frequency	-6.7
Nucleus	1H
Acquired Size	16384
Spectral Size	65536

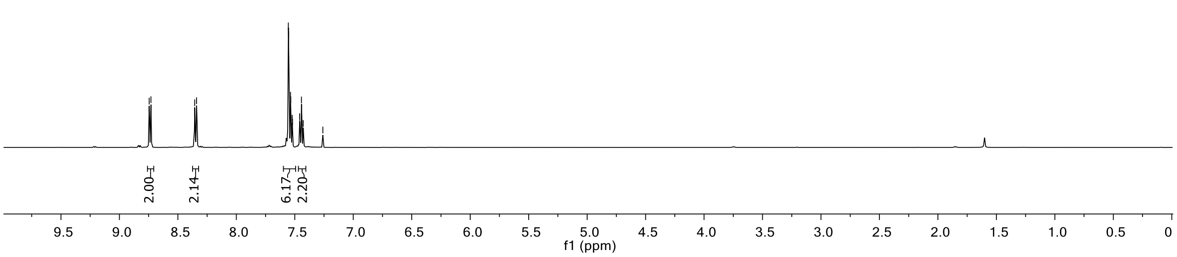

Parameter	Value
Title	CARBON_01
Comment	AN-36
Origin	Varian
Owner	nazarova
Instrument	vnmrs
Solvent	cdcl3
Temperature	25.0
Pulse Sequence	s2pul
Experiment	1D
Number of Scans	2816
Receiver Gain	30
Relaxation Delay	5.0000
Pulse Width	4.9500
Spectrometer Frequency	125.68
Spectral Width	26595.7
Lowest Frequency	-1344.5
Nucleus	13C
Acquired Size	32768
Spectral Size	65536

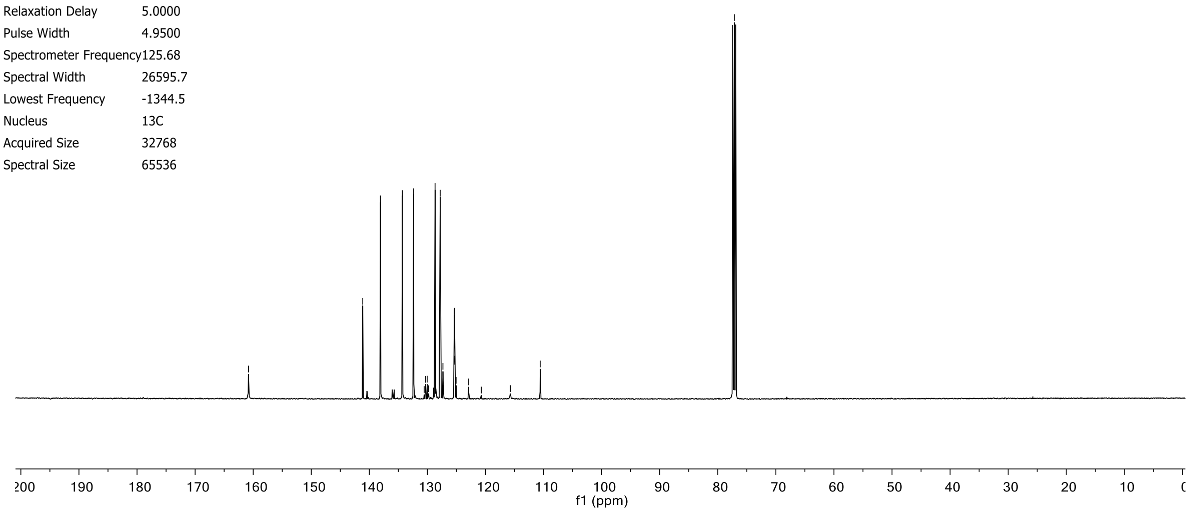

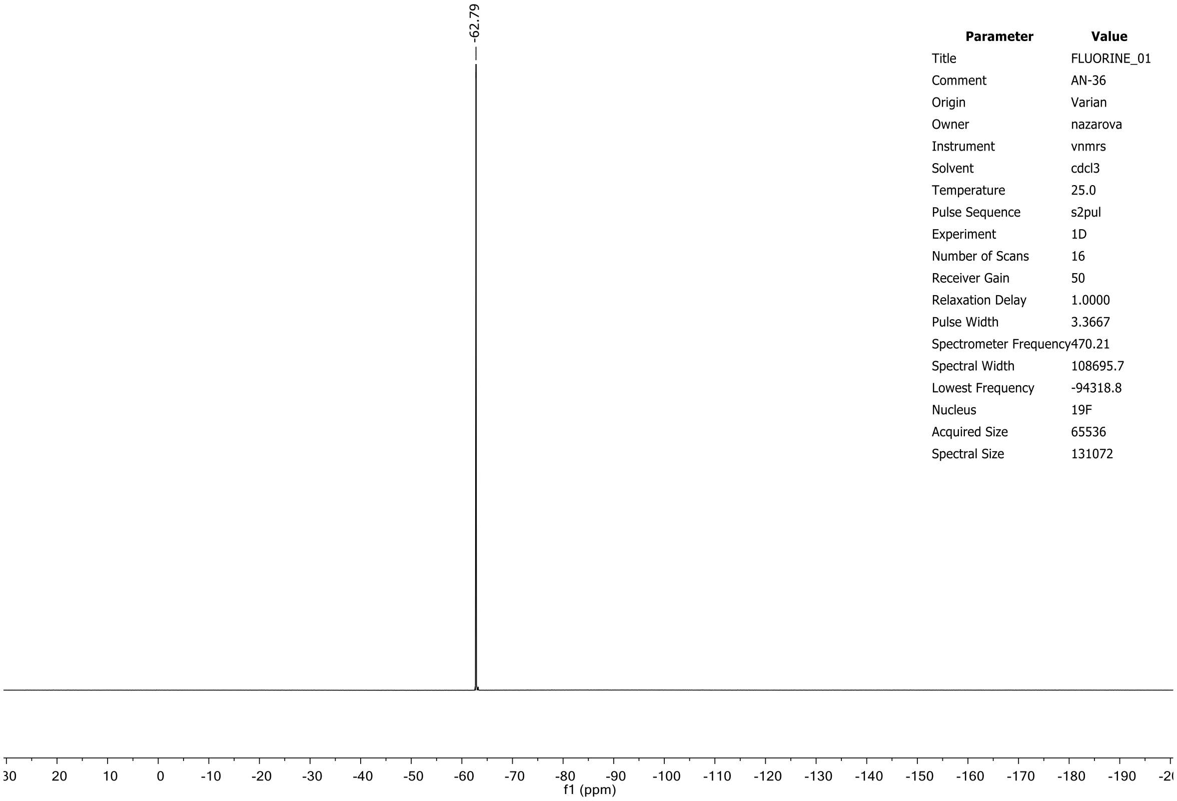
-62.79
Parameter Value
Title FLUORINE_01
Comment AN-36
Origin Varian
Owner nazarova
Instrument vnmrs
Solvent cdcl3
Temperature 25.0
Pulse Sequence s2pul
Experiment 1D
Number of Scans 16
Receiver Gain 50
Relaxation Delay 1.0000
Pulse Width 3.3667
Spectrometer Frequency 470.21
Spectral Width 108695.7
Lowest Frequency -94318.8
Nucleus 19F
Acquired Size 65536
Spectral Size 131072
f1 (ppm)

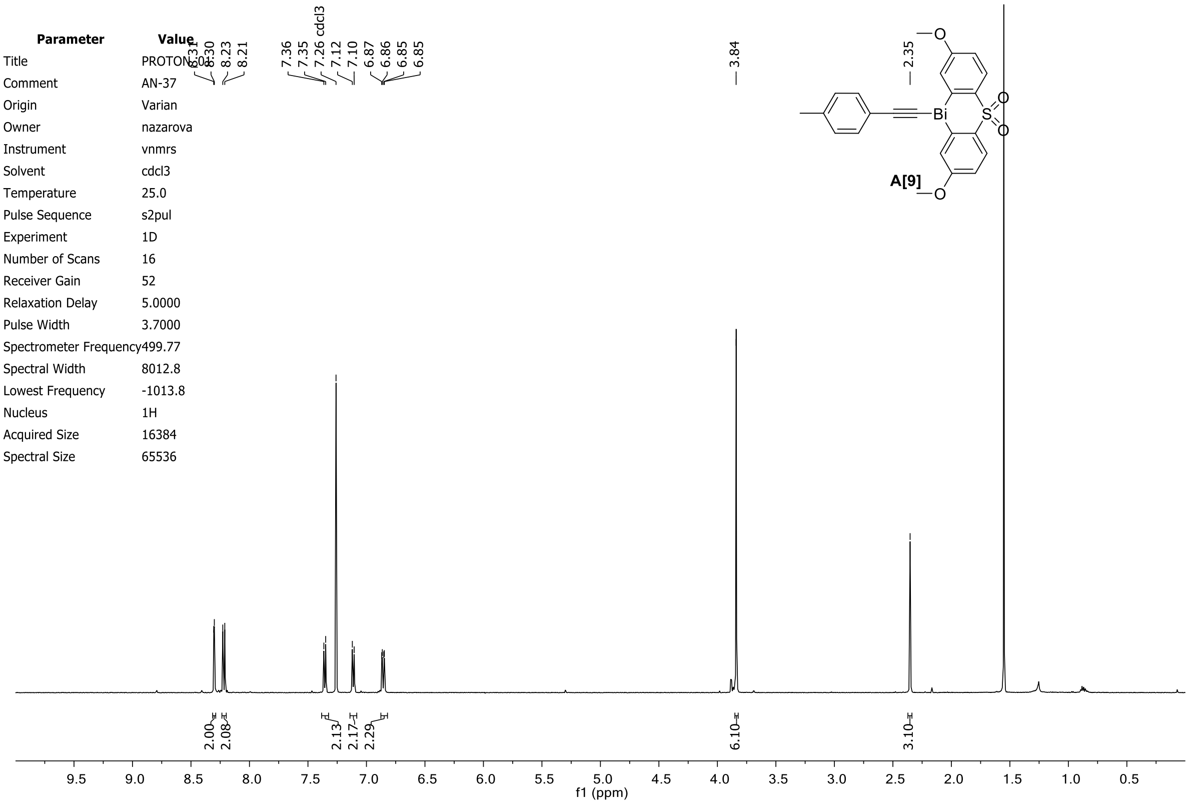
Parameter Value
Title PROTON_01
Comment AN-37
Origin Varian
Owner nazarova
Instrument vnmrs
Solvent cdcl3
Temperature 25.0
Pulse Sequence s2pul
Experiment 1D
Number of Scans 16
Receiver Gain 52
Relaxation Delay 5.0000
Pulse Width 3.7000
Spectrometer Frequency 499.77
Spectral Width 8012.8
Lowest Frequency -1013.8
Nucleus 1H
Acquired Size 16384
Spectral Size 65536
8.31 8.30 8.23 8.21
7.36 7.35 7.26 cdcl3 7.12 7.10 6.87 6.86 6.85 6.85
3.84
2.35
2.00 2.08 2.13 2.17 2.29 6.10 3.10
A[9]
Bi
S
O
f1 (ppm)

Parameter	Value
Title	CARBON_01
Comment	AN-37
Origin	Varian
Owner	nazarova
Instrument	vnmrs
Solvent	cdcl3
Temperature	25.0
Pulse Sequence	s2pul
Experiment	1D
Number of Scans	10300
Receiver Gain	30
Relaxation Delay	1.0000
Pulse Width	4.9500
Spectrometer Frequency	125.68
Spectral Width	31250.0
Lowest Frequency	-1781.6
Nucleus	13C
Acquired Size	32768
Spectral Size	65536

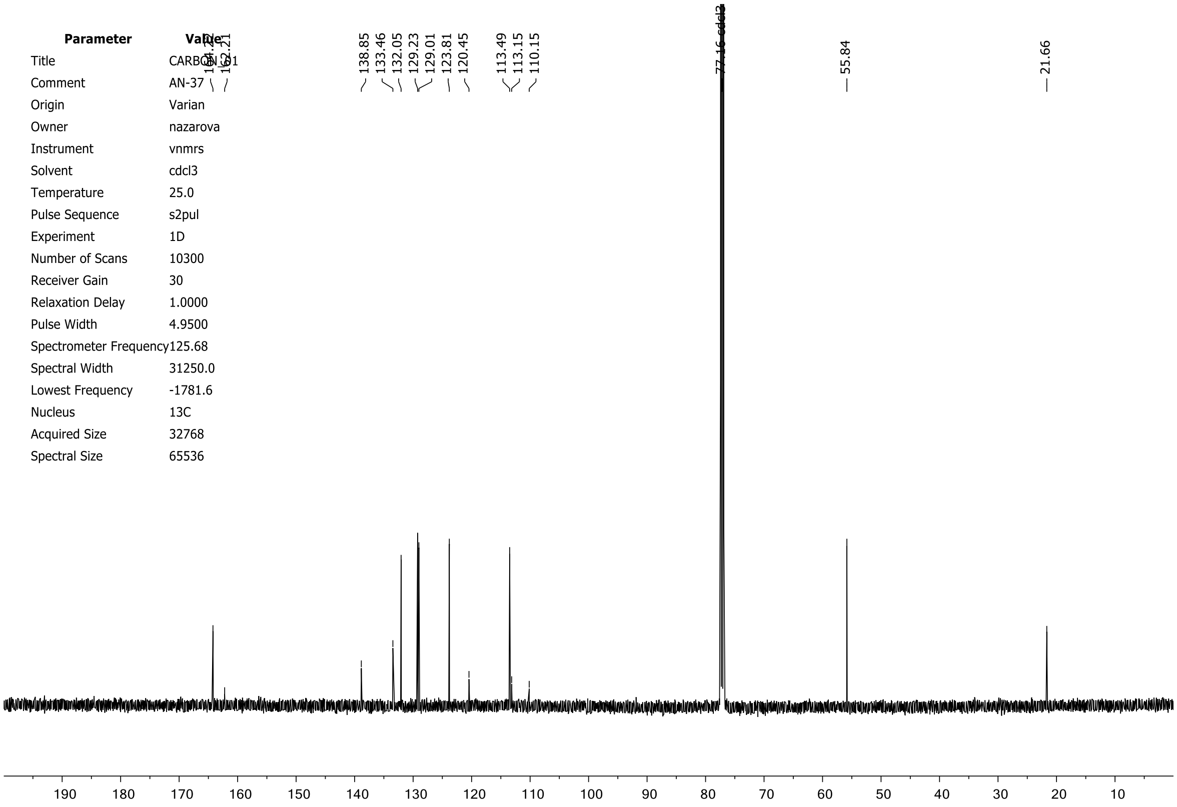

Parameter	Value
Title	[illegible]
Comment	AN-[illegible]
Origin	Varian
Owner	nazarova
Instrument	vnmrs
Solvent	cdcl3
Temperature	25.0
Pulse Sequence	s2pul
Experiment	1D
Number of Scans	8
Receiver Gain	44
Relaxation Delay	10.0000
Pulse Width	3.7000
Spectrometer Frequency	499.76
Spectral Width	5000.0
Lowest Frequency	-6.7
Nucleus	1H
Acquired Size	16384
Spectral Size	65536

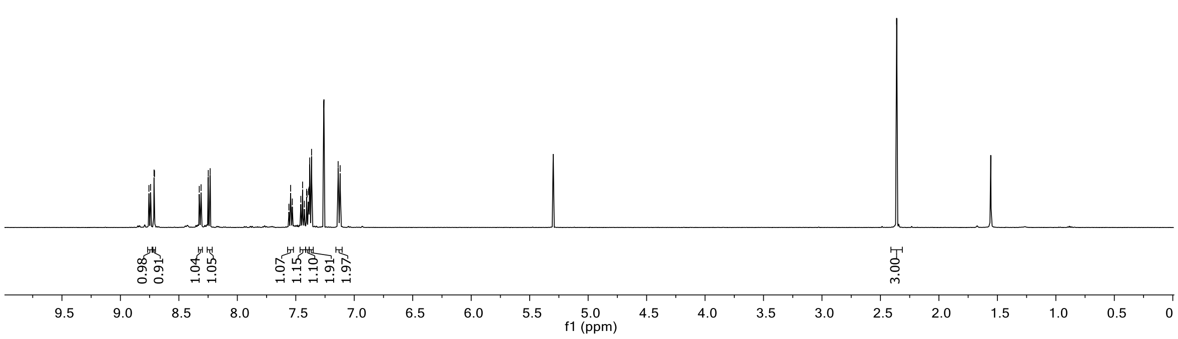

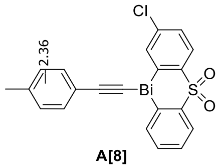

A[8]

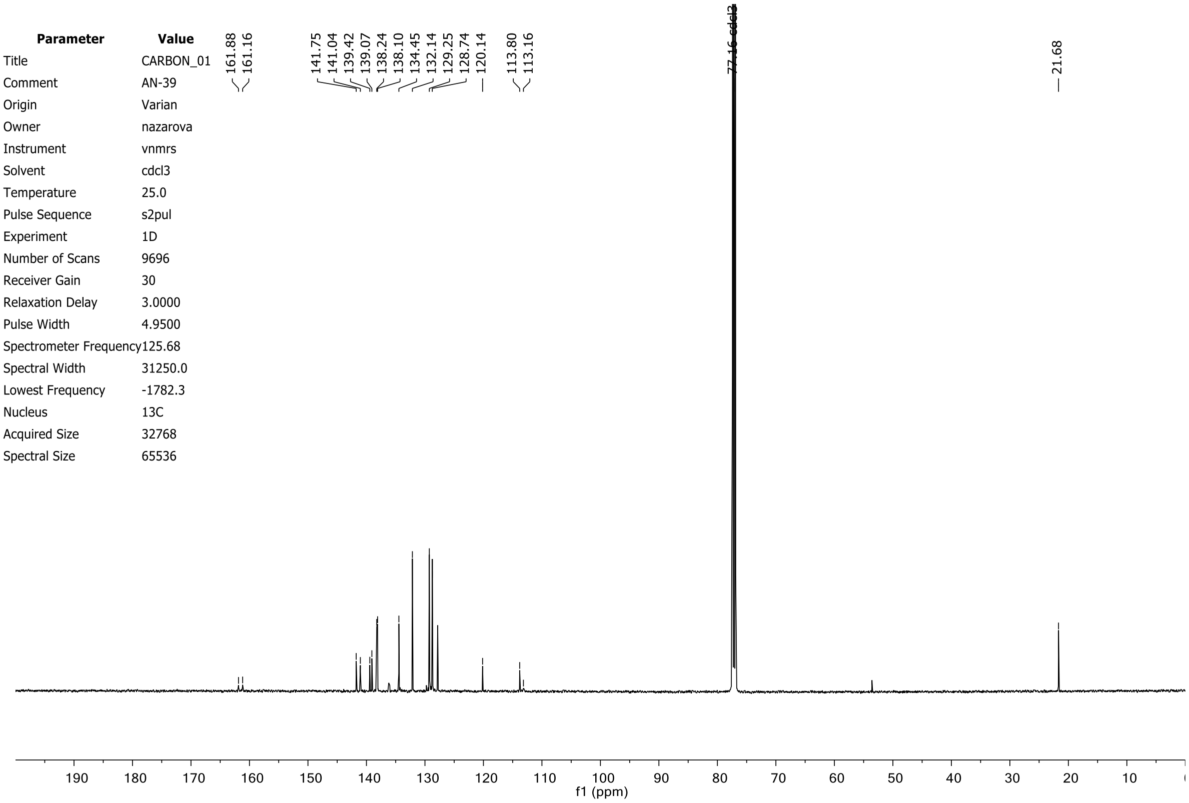

Parameter	Value
Title	CARBON_01
Comment	AN-39
Origin	Varian
Owner	nazarova
Instrument	vnmrs
Solvent	cdcl3
Temperature	25.0
Pulse Sequence	s2pul
Experiment	1D
Number of Scans	9696
Receiver Gain	30
Relaxation Delay	3.0000
Pulse Width	4.9500
Spectrometer Frequency	125.68
Spectral Width	31250.0
Lowest Frequency	-1782.3
Nucleus	13C
Acquired Size	32768
Spectral Size	65536

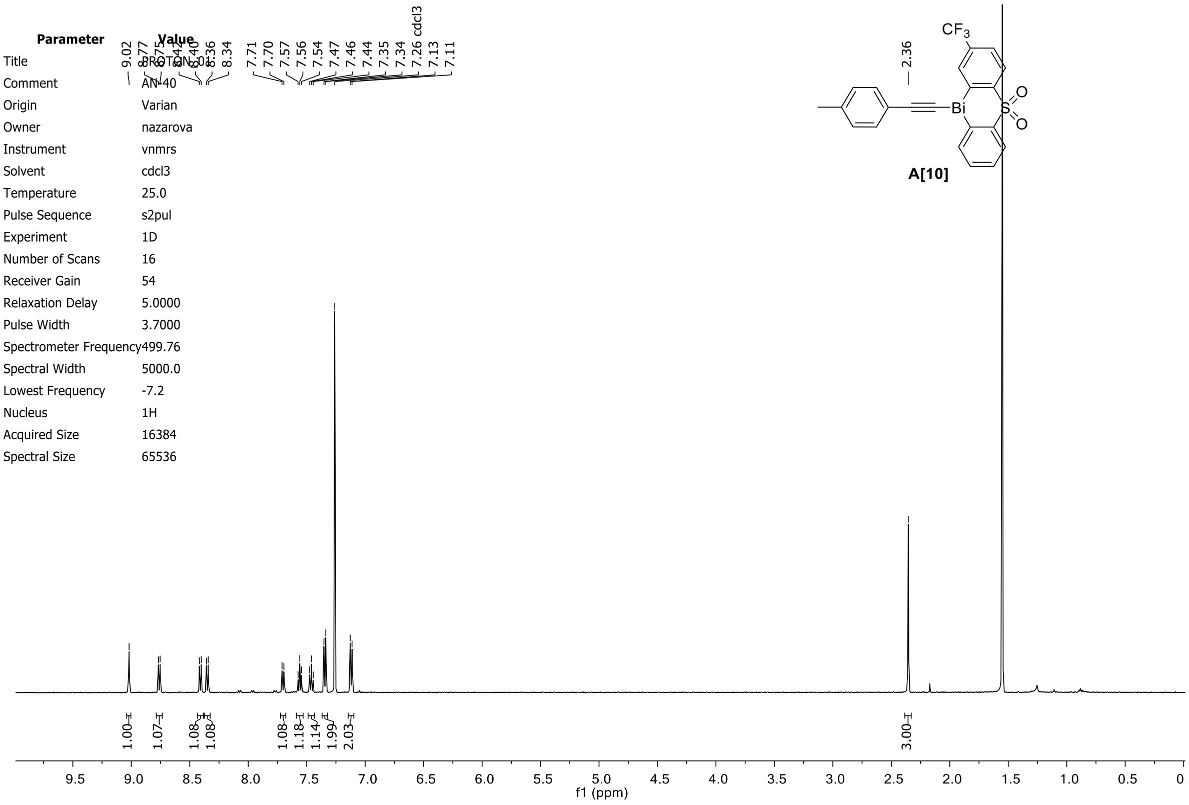

Parameter	Value
Title	PROTON_01
Comment	AN-40
Origin	Varian
Owner	nazarova
Instrument	vnmrs
Solvent	cdcl3
Temperature	25.0
Pulse Sequence	s2pul
Experiment	1D
Number of Scans	16
Receiver Gain	54
Relaxation Delay	5.0000
Pulse Width	3.7000
Spectrometer Frequency	499.76
Spectral Width	5000.0
Lowest Frequency	-7.2
Nucleus	1H
Acquired Size	16384
Spectral Size	65536

Parameter	Value
Title	CARBON_01
Comment	AN-40
Origin	Varian
Owner	nazarova
Instrument	vnmrs
Solvent	cdcl3
Temperature	25.0
Pulse Sequence	s2pul
Experiment	1D
Number of Scans	9216
Receiver Gain	30
Relaxation Delay	3.0000
Pulse Width	4.9500
Spectrometer Frequency	125.68
Spectral Width	31250.0
Lowest Frequency	-1801.8
Nucleus	13C
Acquired Size	32768
Spectral Size	65536

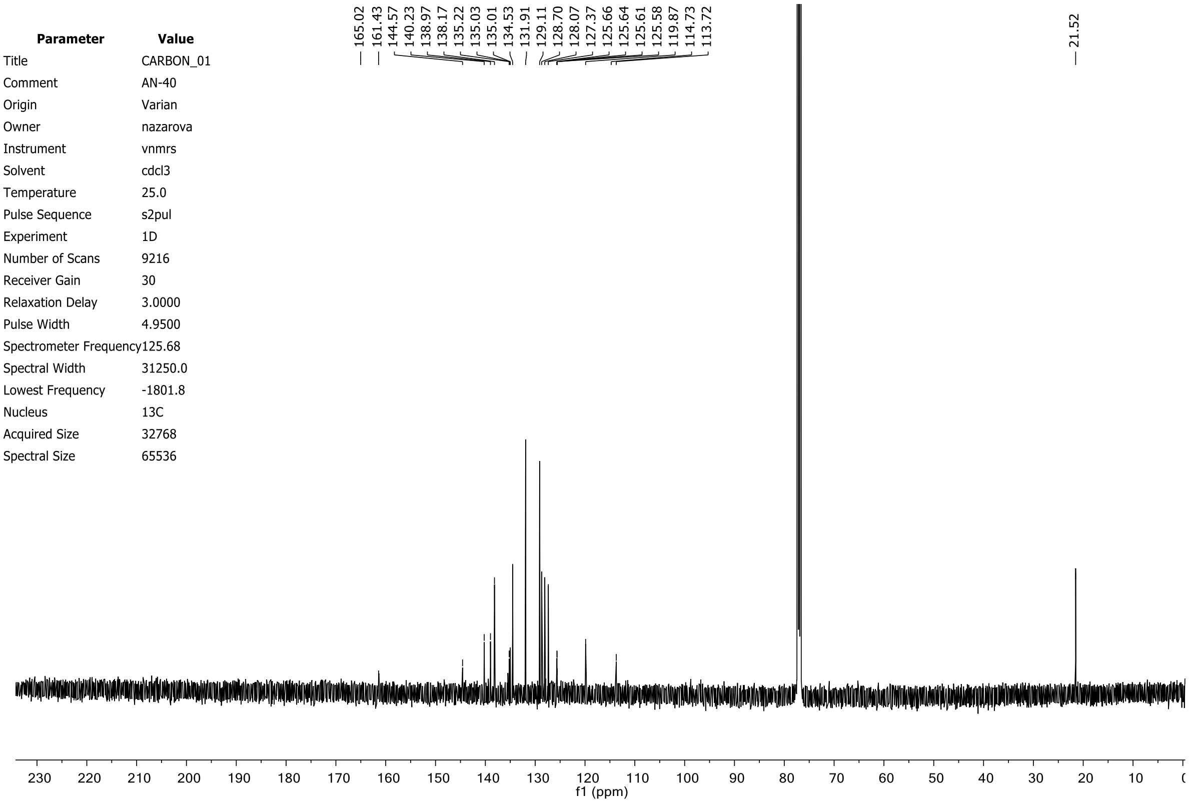

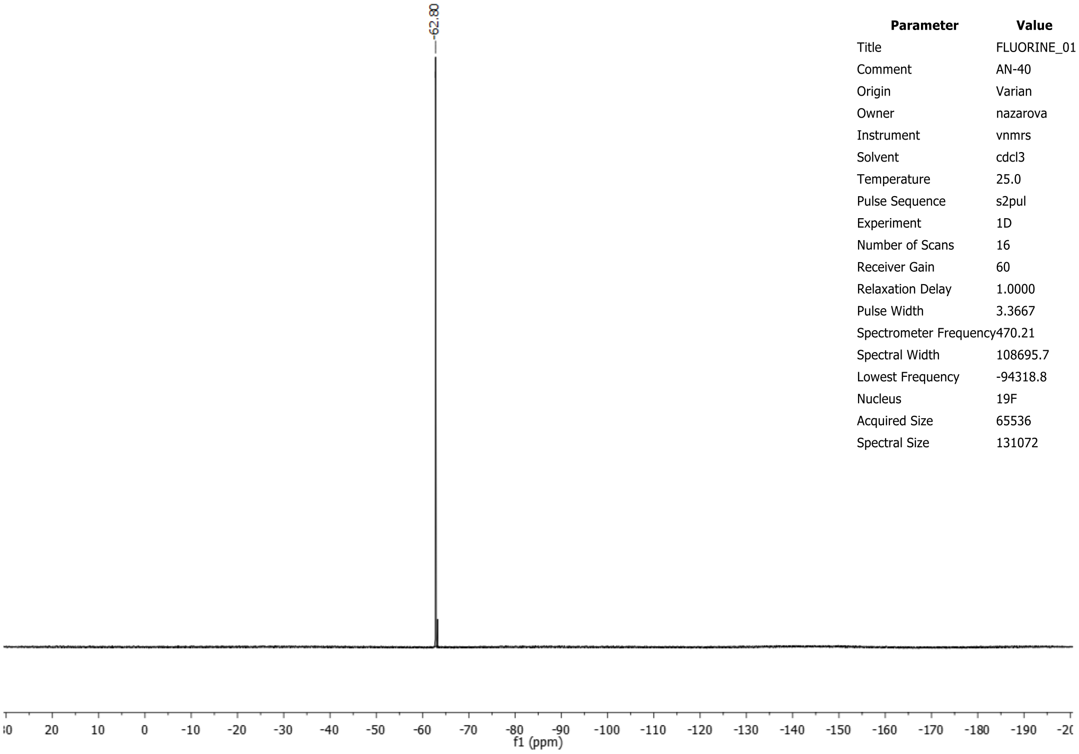

Parameter	Value
Title	FLUORINE_01
Comment	AN-40
Origin	Varian
Owner	nazarova
Instrument	vnmrs
Solvent	cdcl3
Temperature	25.0
Pulse Sequence	s2pul
Experiment	1D
Number of Scans	16
Receiver Gain	60
Relaxation Delay	1.0000
Pulse Width	3.3667
Spectrometer Frequency	470.21
Spectral Width	108695.7
Lowest Frequency	-94318.8
Nucleus	19F
Acquired Size	65536
Spectral Size	131072

Parameter	Value
Title	PROTON_01
Comment	AN-42
Origin	Varian
Owner	nazarova
Instrument	vnmrs
Solvent	cdcl3
Temperature	25.0
Pulse Sequence	s2pul
Experiment	1D
Number of Scans	8
Receiver Gain	46
Relaxation Delay	10.0000
Pulse Width	3.7000
Spectrometer Frequency	499.76
Spectral Width	5000.0
Lowest Frequency	-7.1
Nucleus	1H
Acquired Size	16384
Spectral Size	65536

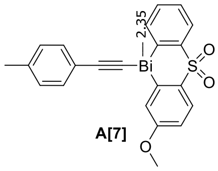

A[7]

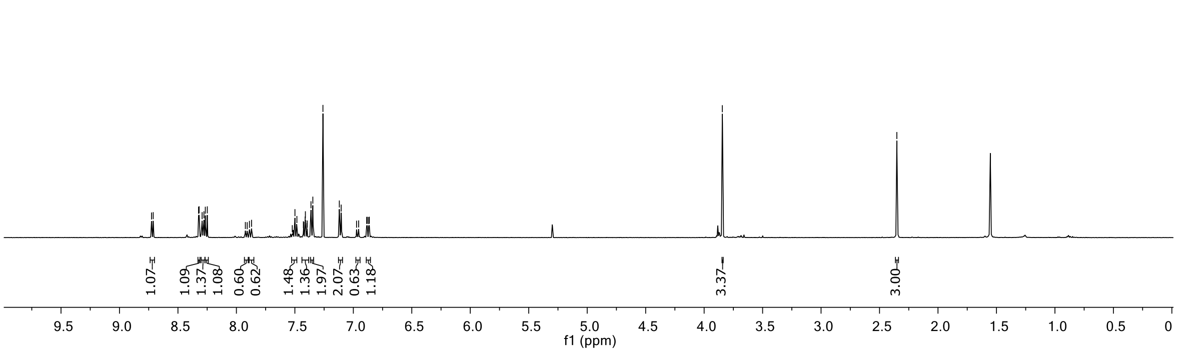

Parameter	Value
Title	CARBON_01
Comment	AN-42
Origin	Varian
Owner	nazarova
Instrument	vnmrs
Solvent	cdcl3
Temperature	25.0
Pulse Sequence	s2pul
Experiment	1D
Number of Scans	14720
Receiver Gain	30
Relaxation Delay	3.0000
Pulse Width	4.9500
Spectrometer Frequency	125.68
Spectral Width	31250.0
Lowest Frequency	-1782.2
Nucleus	13C
Acquired Size	32768
Spectral Size	65536

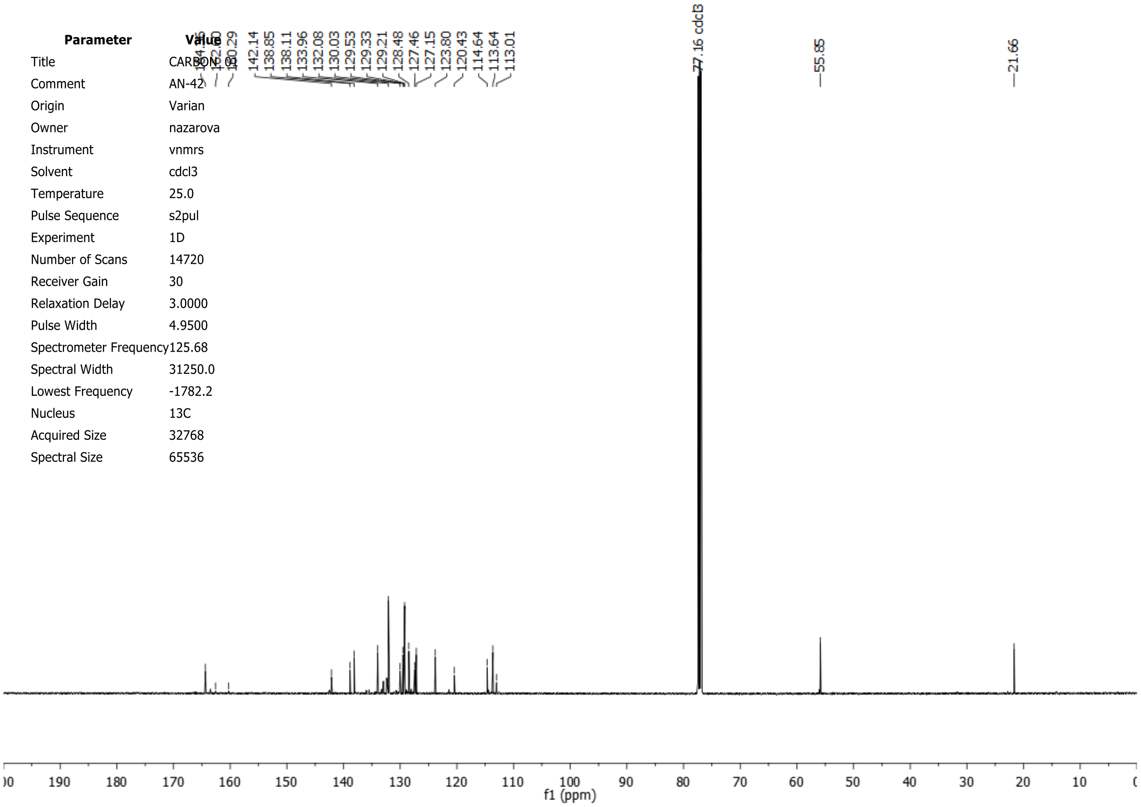

Parameter	Value
Title	PROTON_01
Comment	AN-43
Origin	Varian
Owner	nazarova
Instrument	vnmrs
Solvent	cdcl3
Temperature	25.0
Pulse Sequence	s2pul
Experiment	1D
Number of Scans	64
Receiver Gain	30
Relaxation Delay	5.0000
Pulse Width	6.0000
Spectrometer Frequency	499.76
Spectral Width	5000.0
Lowest Frequency	63.2
Nucleus	1H
Acquired Size	16384
Spectral Size	65536

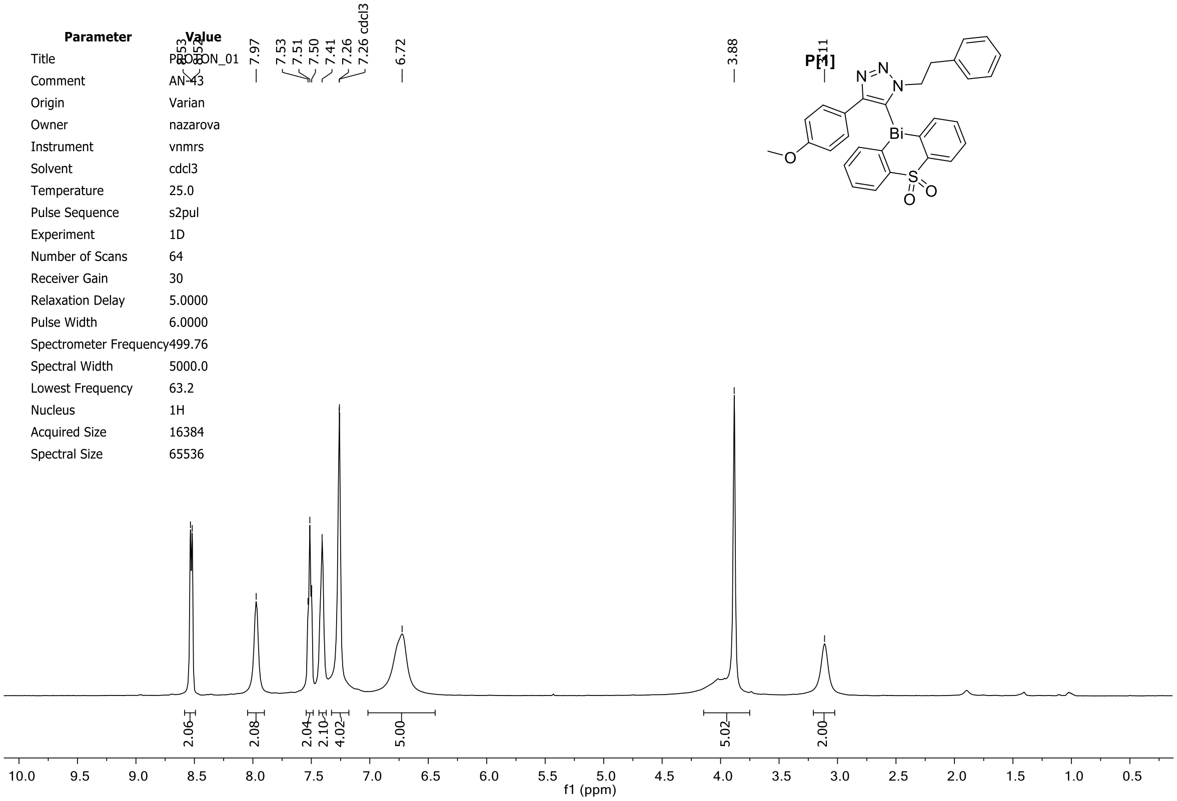

Parameter	Value
Title	CARBON_01
Comment	AN-43
Origin	Varian
Owner	nazarova
Instrument	vnmrs
Solvent	cdcl3
Temperature	25.0
Pulse Sequence	s2pul
Experiment	1D
Number of Scans	11264
Receiver Gain	30
Relaxation Delay	3.0000
Pulse Width	5.3500
Spectrometer Frequency	125.68
Spectral Width	31250.0
Lowest Frequency	-1788.7
Nucleus	13C
Acquired Size	32768
Spectral Size	65536

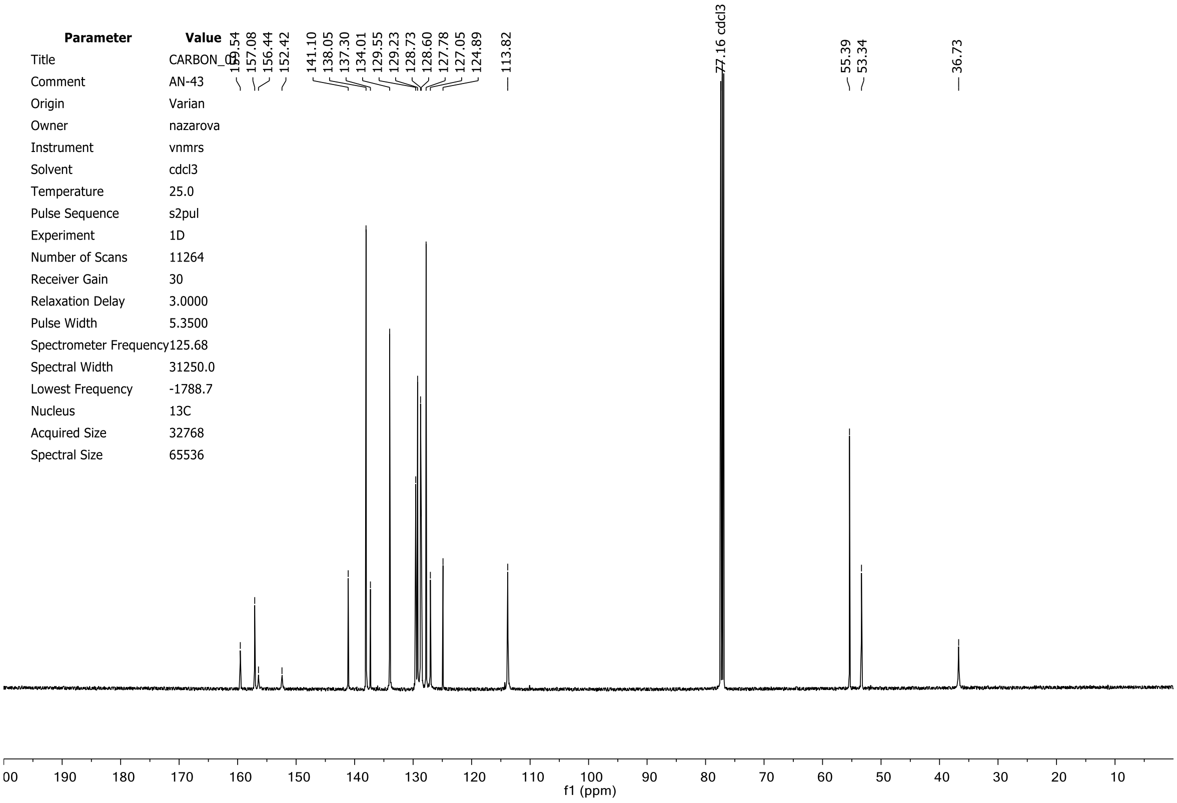

Parameter	Value
Title	PROTON_01
Comment	AN-44
Origin	Varian
Owner	nazarova
Instrument	vnmrs
Solvent	cdcl3
Temperature	25.0
Pulse Sequence	s2pul
Experiment	1D
Number of Scans	32
Receiver Gain	42
Relaxation Delay	5.0000
Pulse Width	6.0000
Spectrometer Frequency	499.77
Spectral Width	6009.6
Lowest Frequency	-12.2
Nucleus	1H
Acquired Size	16384
Spectral Size	65536

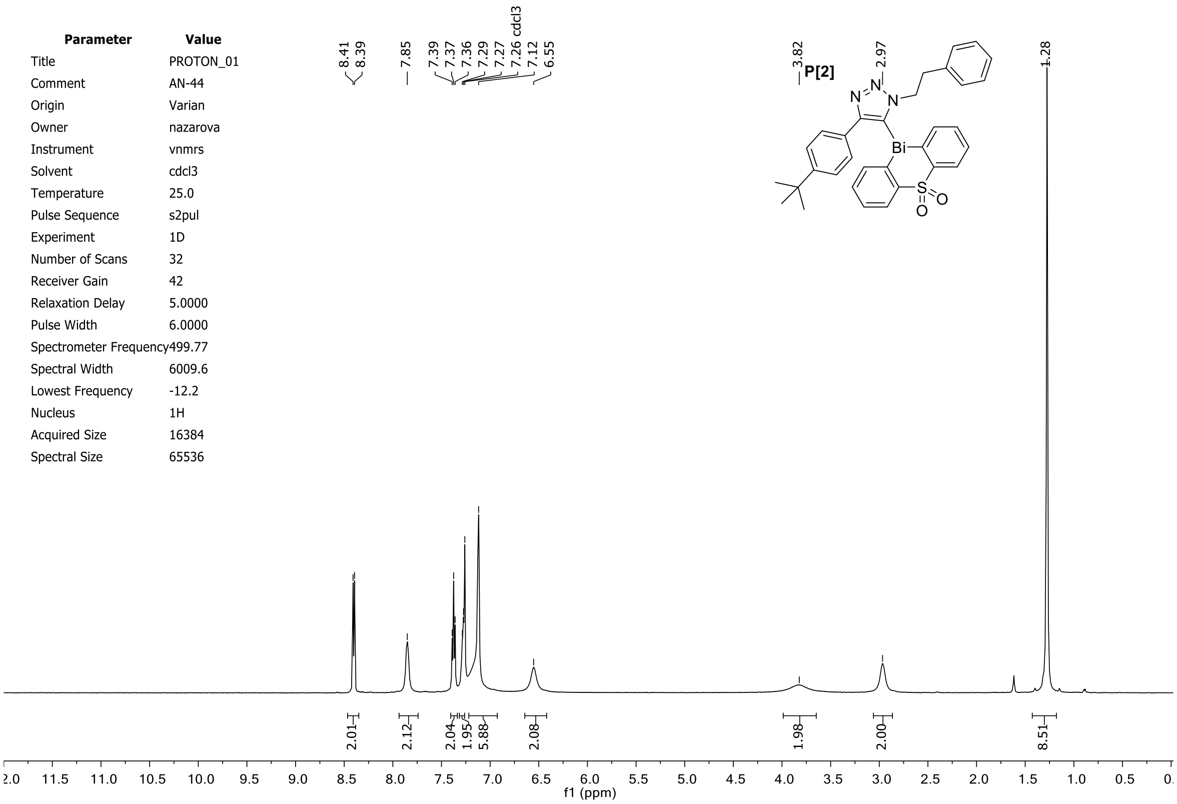

Parameter	Value
Title	CARBON_01
Comment	AN-44
Origin	Varian
Owner	nazarova
Instrument	vnmrs
Solvent	cdcl3
Temperature	25.0
Pulse Sequence	s2pul
Experiment	1D
Number of Scans	1088
Receiver Gain	30
Relaxation Delay	3.0000
Pulse Width	5.3500
Spectrometer Frequency	125.68
Spectral Width	31250.0
Lowest Frequency	-1801.8
Nucleus	13C
Acquired Size	32768
Spectral Size	65536

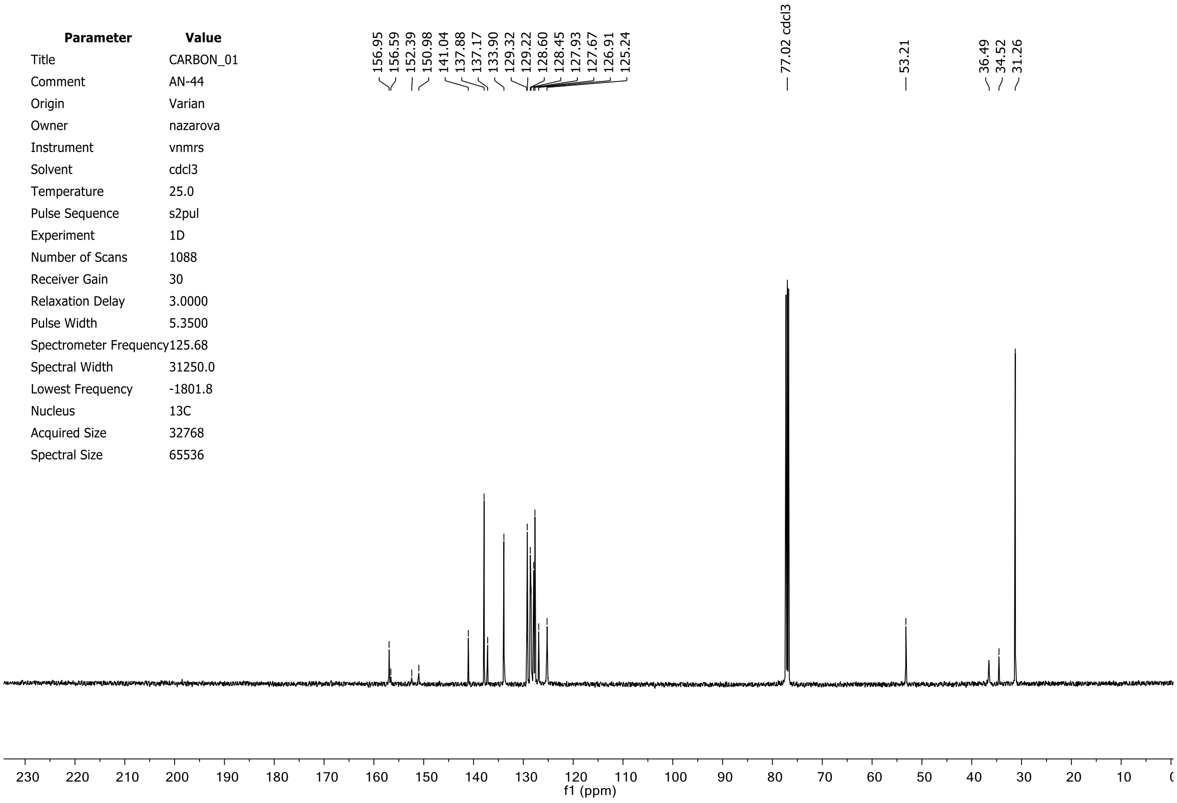

Parameter	Value
Title	PROTON_02
Comment	AN-45
Origin	Varian
Owner	nazarova
Instrument	vnmrs
Solvent	cdcl3
Temperature	25.0
Pulse Sequence	s2pul
Experiment	1D
Number of Scans	64
Receiver Gain	30
Relaxation Delay	5.0000
Pulse Width	6.0000
Spectrometer Frequency	499.77
Spectral Width	6009.6
Lowest Frequency	64.7
Nucleus	1H
Acquired Size	16384
Spectral Size	65536

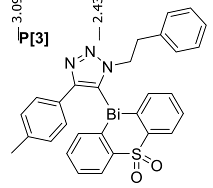

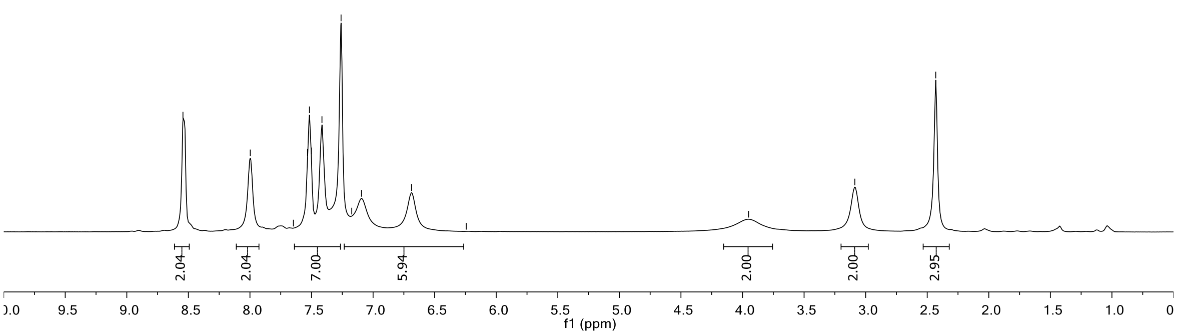

Parameter	Value
Title	CARBON_01
Comment	AN-45
Origin	Varian
Owner	nazarova
Instrument	vnmrs
Solvent	cdcl3
Temperature	25.0
Pulse Sequence	s2pul
Experiment	1D
Number of Scans	8192
Receiver Gain	30
Relaxation Delay	3.0000
Pulse Width	5.3500
Spectrometer Frequency	125.68
Spectral Width	31250.0
Lowest Frequency	-1794.1
Nucleus	13C
Acquired Size	32768
Spectral Size	65536

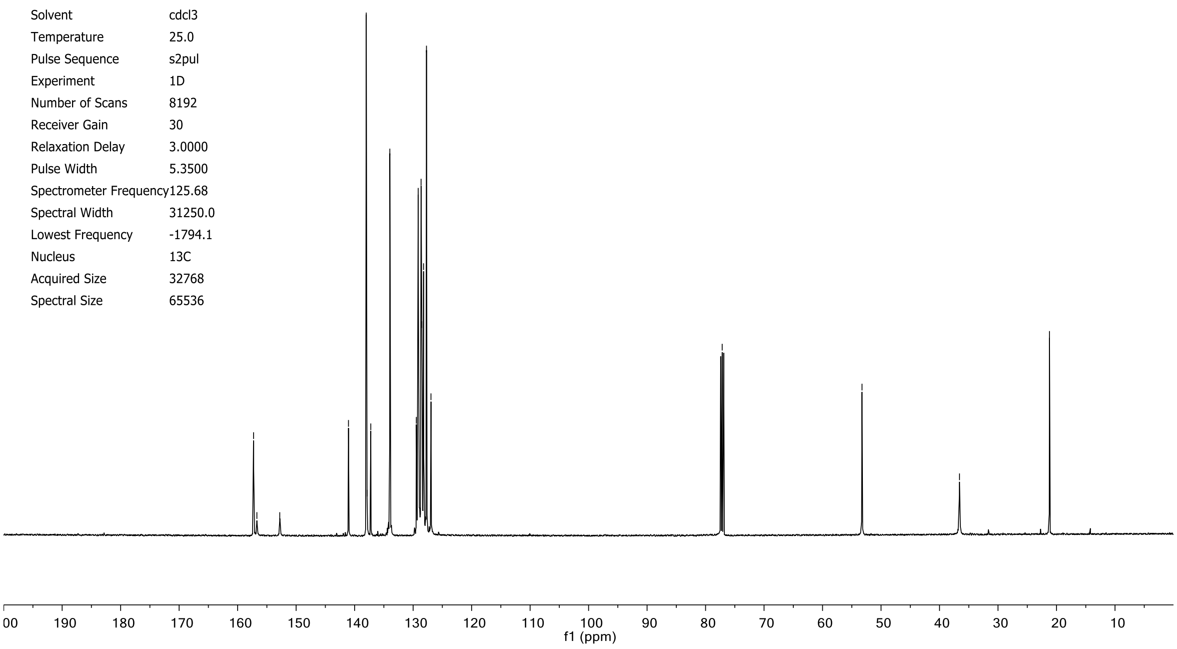

Parameter	Value
Title	PROTON_02
Comment	AN-46
Origin	Varian
Owner	nazarova
Instrument	vnmrs
Solvent	cdcl3
Temperature	25.0
Pulse Sequence	s2pul
Experiment	1D
Number of Scans	64
Receiver Gain	30
Relaxation Delay	5.0000
Pulse Width	6.0000
Spectrometer Frequency	499.77
Spectral Width	6009.6
Lowest Frequency	58.7
Nucleus	1H
Acquired Size	16384
Spectral Size	65536

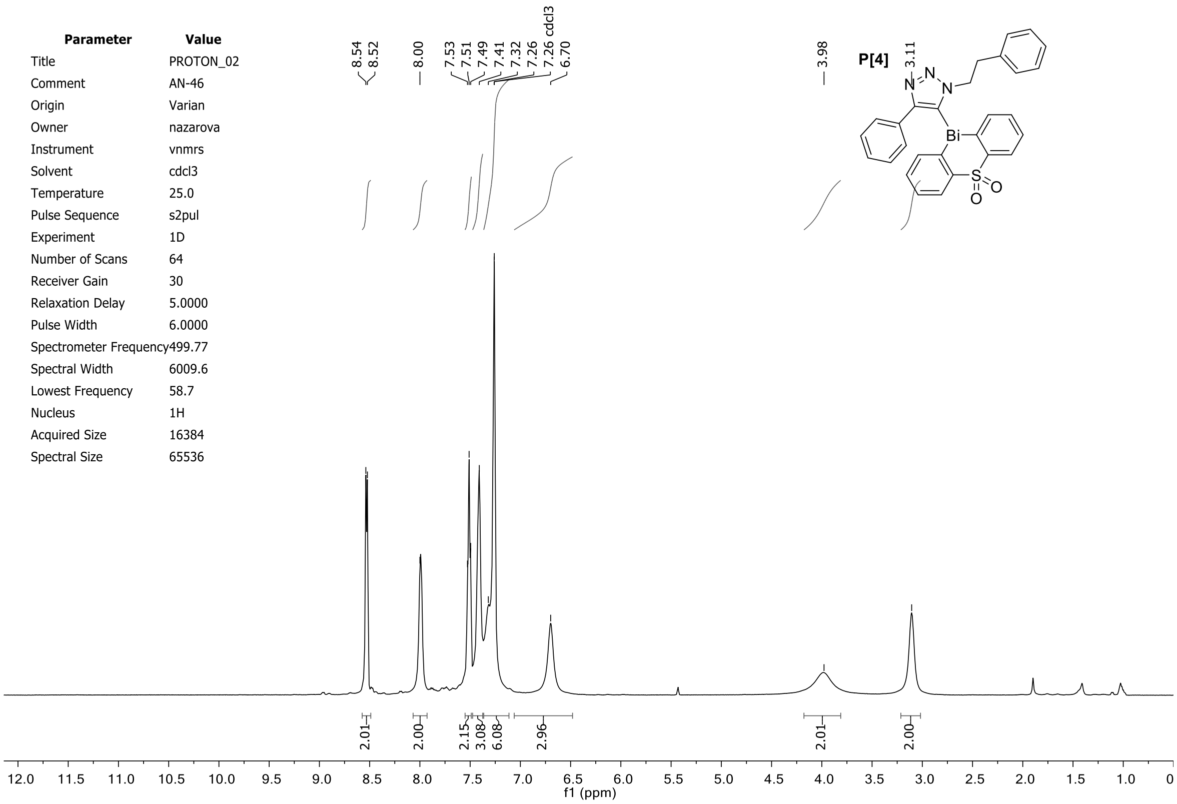

Parameter	Value
Title	CARBON_01
Comment	AN-46
Origin	Varian
Owner	nazarova
Instrument	vnmrs
Solvent	cdcl3
Temperature	25.0
Pulse Sequence	s2pul
Experiment	1D
Number of Scans	14592
Receiver Gain	30
Relaxation Delay	3.0000
Pulse Width	5.3500
Spectrometer Frequency	125.68
Spectral Width	31250.0
Lowest Frequency	-1789.6
Nucleus	13C
Acquired Size	32768
Spectral Size	65536

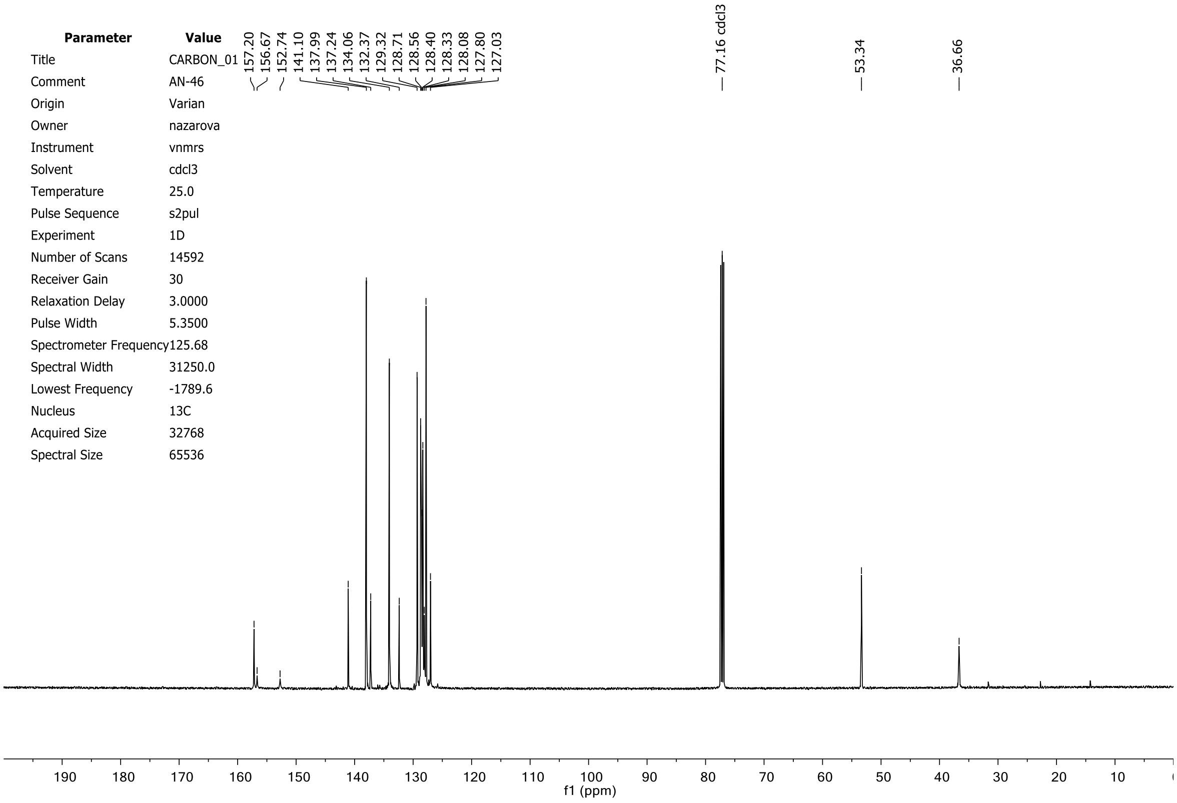

Parameter	Value
Title	PROTON_02
Comment	AN-47
Origin	Varian
Owner	nazarova
Instrument	vnmrs
Solvent	cdcl3
Temperature	25.0
Pulse Sequence	s2pul
Experiment	1D
Number of Scans	64
Receiver Gain	44
Relaxation Delay	5.0000
Pulse Width	6.0000
Spectrometer Frequency	499.76
Spectral Width	5000.0
Lowest Frequency	-8.2
Nucleus	1H
Acquired Size	16384
Spectral Size	65536

P[5]

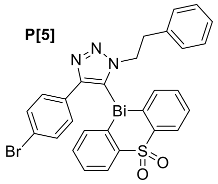

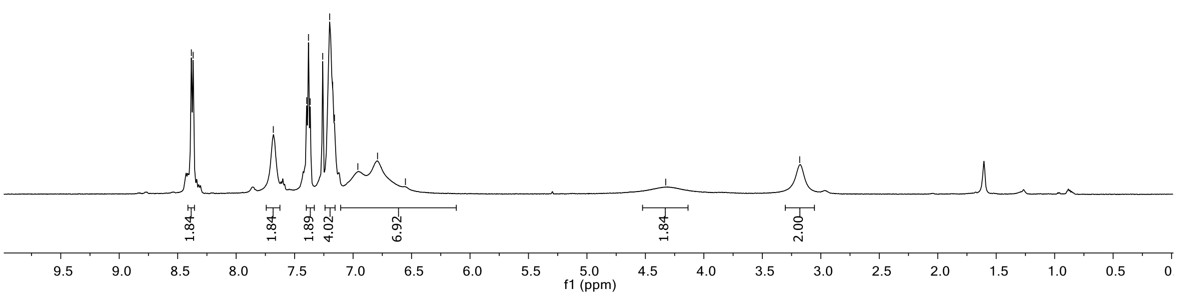

Parameter	Value
Title	CARBON_01
Comment	AN-47
Origin	Varian
Owner	nazarova
Instrument	vnmrs
Solvent	cdcl3
Temperature	25.0
Pulse Sequence	s2pul
Experiment	1D
Number of Scans	11776
Receiver Gain	30
Relaxation Delay	3.0000
Pulse Width	5.3500
Spectrometer Frequency	125.68
Spectral Width	31250.0
Lowest Frequency	-1784.0
Nucleus	13C
Acquired Size	32768
Spectral Size	65536

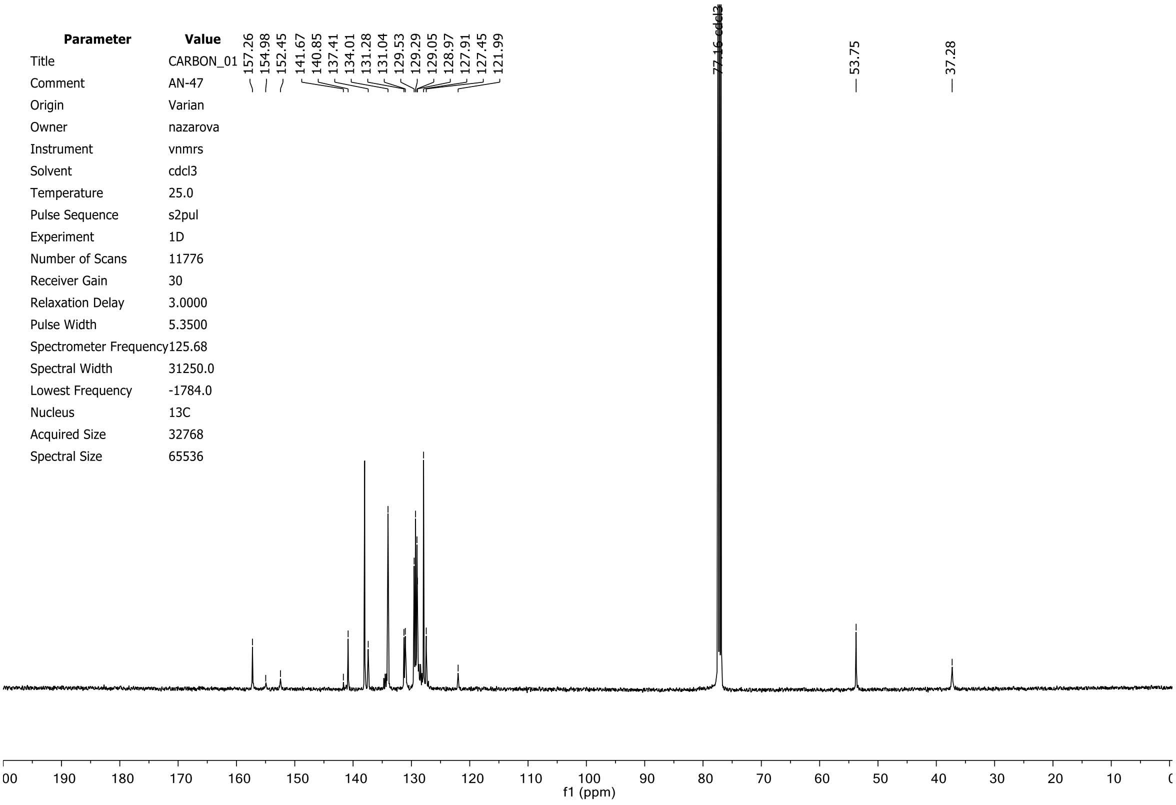

Parameter	Value
Title	PROTON_02
Comment	AN-48
Origin	Varian
Owner	nazarova
Instrument	vnmrs
Solvent	cdcl3
Temperature	25.0
Pulse Sequence	s2pul
Experiment	1D
Number of Scans	8
Receiver Gain	30
Relaxation Delay	1.0000
Pulse Width	4.6500
Spectrometer Frequency	599.80
Spectral Width	9615.4
Lowest Frequency	-1200.5
Nucleus	1H
Acquired Size	16384
Spectral Size	65536

P[6]

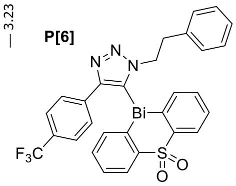

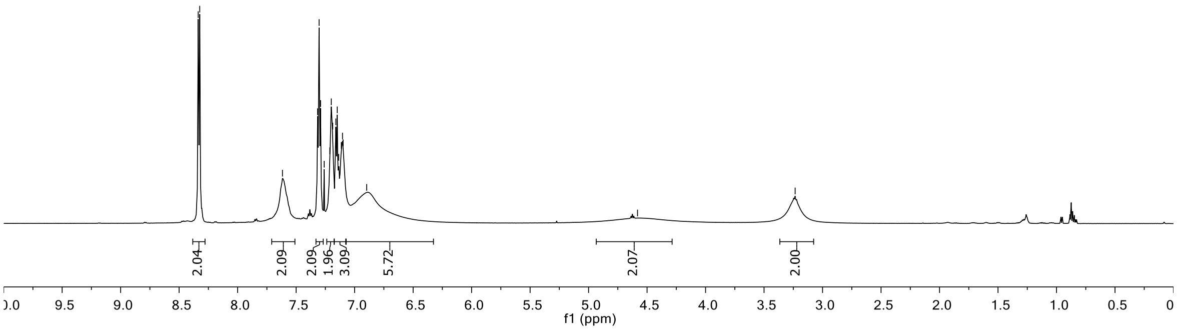

Parameter	Value
Title	CARBON_01
Comment	AN-48
Origin	Varian
Owner	nazarova
Instrument	vnmrs
Solvent	cdcl3
Temperature	25.0
Pulse Sequence	s2pul
Experiment	1D
Number of Scans	4864
Receiver Gain	30
Relaxation Delay	3.0000
Pulse Width	4.7500
Spectrometer Frequency	150.84
Spectral Width	37878.8
Lowest Frequency	-2336.8
Nucleus	13C
Acquired Size	32768
Spectral Size	65536

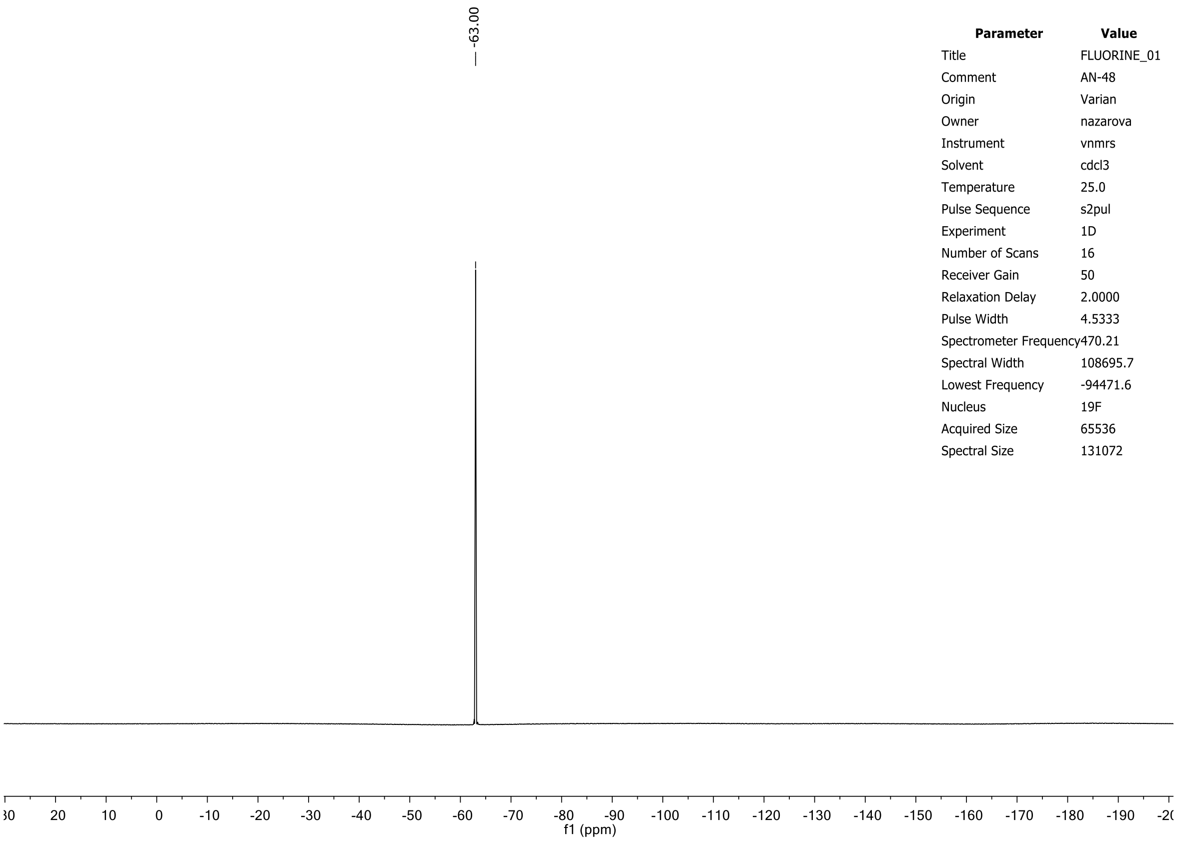

Parameter	Value
Title	FLUORINE_01
Comment	AN-48
Origin	Varian
Owner	nazarova
Instrument	vnmrs
Solvent	cdcl3
Temperature	25.0
Pulse Sequence	s2pul
Experiment	1D
Number of Scans	16
Receiver Gain	50
Relaxation Delay	2.0000
Pulse Width	4.5333
Spectrometer Frequency	470.21
Spectral Width	108695.7
Lowest Frequency	-94471.6
Nucleus	19F
Acquired Size	65536
Spectral Size	131072

6. References

1. Alper, J. S.; Gelb, R. I., Standard errors and confidence intervals in nonlinear regression: comparison of Monte Carlo and parametric statistics. *J. Phys. Chem.* **2002,** *94* (11), 4747-4751.

2. Box, G. E. P.; Muller, M. E., A Note on the Generation of Random Normal Deviates. *Ann. Math. Stat.* **1958,** *29* (2), 610-611.

3. CCDC *Mercury 2020.1*, CCDC: 2020.

4. Kwong, F. Y.; Buchwald, S. L., A General, Efficient, and Inexpensive Catalyst System for the Coupling of Aryl Iodides and Thiols. *Org. Lett.* **2002,** *4* (20), 3517-3520.

5. Bahekar, S. S.; Sarkate, A. P.; Wadhai, V. M.; Wakte, P. S.; Shinde, D. B., CuI catalyzed CS bond formation by using nitroarenes. *Catal. Commun.* **2013,** *41*, 123-125.

6. Sikari, R.; Sinha, S.; Das, S.; Saha, A.; Chakraborty, G.; Mondal, R.; Paul, N. D., Achieving Nickel Catalyzed C–S Cross-Coupling under Mild Conditions Using Metal–Ligand Cooperativity. *J. Org. Chem.* **2019,** *84* (7), 4072-4085.

7. Thomas, J.; Van Hecke, K.; Robeyns, K.; Van Rossom, W.; Sonawane, M. P.; Van Meervelt, L.; Smet, M.; Maes, W.; Dehaen, W., Homothiacalix[4]arenes: Synthetic Exploration and Solid-State Structures. *Chem. Eur. J.* **2011,** *17* (37), 10339-10349.

8. Margraf, N.; Manolikakes, G., One-Pot Synthesis of Aryl Sulfones from Organometallic Reagents and Iodonium Salts. *J. Org. Chem.* **2015,** *80* (5), 2582-2600.

9. Chang, Y.; Lee, H. H.; Kim, S. H.; Jo, T. S.; Bae, C., Scope and Regioselectivity of Iridium-Catalyzed C–H Borylation of Aromatic Main-Chain Polymers. *Macromolecules* **2013,** *46* (5), 1754-1764.

10. Suzuki, H.; Murafuji, T.; Azuma, N., Synthesis and reactions of some new heterocyclic bismuth-(III) and -(V) compounds. 5,10-Dihydrodibenzo[b,e]bismine and related systems. *J. Chem. Soc., Perkin Trans. 1* **1992,** (13).

11. Worrell Brady, T.; Ellery Shelby, P.; Fokin Valery, V., Copper(I)-Catalyzed Cycloaddition of Bismuth(III) Acetylides with Organic Azides: Synthesis of Stable Triazole Anion Equivalents. *Angew. Chem. Int. Ed.* **2013,** *52* (49), 13037-13041.
